# Stereodivergent Access to Trisubstituted Alkenylboronate Esters through Alkene Isomerization

**DOI:** 10.1021/acs.orglett.1c03513

**Published:** 2021-11-12

**Authors:** Lucas Segura, Itai Massad, Masamichi Ogasawara, Ilan Marek

**Affiliations:** ‡ Schulich Faculty of Chemistry, Technion − Israel Institute of Technology, Haifa 3200009, Israel; † Department of Natural Science, Graduate School of Science and Technology, Tokushima University, Tokushima 770-8506, Japan

## Abstract

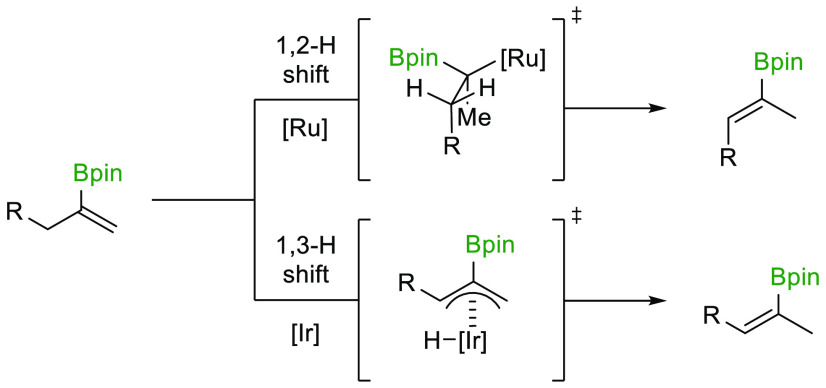

We report an efficient method for the preparation of synthetically valuable trisubstituted alkenylboronate esters through alkene isomerization of their readily available 1,1-disubstituted regioisomeric counterparts. Either stereoisomer of the target alkenylboronate motif can be obtained at will from the same starting material by employing different isomerization catalysts.

The synthetic utility of alkenylboron compounds is widely accepted thanks to their role in various C–C bond forming reactions. The foremost example of such a process is the Suzuki-Miyaura cross-coupling reaction,^1−3^ which has been extensively employed to form highly substituted alkenes and dienes, structures featured in bioactive natural products.^2,4^ The value of this motif is amplified by several transformations that leverage the alkene π-system through electrophile-induced 1,2-boronate rearrangements, affording either new alkene products, as in the Zweifel olefination,^5,6^ or products of net C–C bond addition as in the Morken conjugative cross-coupling reaction.^7−11^ Finally, instead of being directly engaged in C–C bond formation, oxidation of the C–B bond can result in boron enolates, which have proven useful in the realm of stereoselective aldol reactions.^12−14^ The stereospecific nature of the above processes requires complete control over the stereochemistry of the alkenylboron fragment to secure access to stereodefined products. Accordingly, considerable effort has been dedicated to the stereoselective generation of the alkenylboron motif. The pioneered route is the anti-Markovnikov hydroboration of alkynes,^15−22^ which performs admirably for terminal alkynes and affords *E*-alkenylboron products with complete regio- and stereocontrol. Unfortunately, the formation of trisubstituted alkenylboron compounds through this strategy is significantly more challenging. For example, canonical hydroboration of unbiased internal alkynes suffers from substantial regioselectivity issues. Recent efforts directed toward the stereoselective preparation of trisubstituted alkenylboron compounds are depicted in Scheme 1 and range from the Ru-catalyzed formal *trans*-hydroboration reactions systems (Scheme 1a),^19,22−24^ to stereoselective elimination reactions (Scheme 1b)^25^ and boron-Wittig reactions (Scheme 1c).^26^ Alternative approaches utilize alkene isomerization to establish the regio- and stereochemistry of the alkenylboron motif.^27^ Such a strategy has been explored by Suginome in the isomerization of boronate esters derived from the silaboration and diboration of terminal alkynes, where highly substituted alkenylboronate esters were generated from readily available starting materials (Scheme 1d).^28^ In this context, our group has recently demonstrated that ω-ene alkylboronate esters can undergo long-range isomerization in the presence of a Ru–H catalyst to result in stereodefined alkenylboronate esters (Scheme 1e).^29^ In line with our interest in the utilization of alkene isomerization in stereoselective synthesis,^30−36^ we set out to explore the alkene isomerization of readily available 1,1-disubstituted alkenylboronates^37−40^ into either (*E*)- or (*Z*)-trisubstituted alkenylboronate esters (Scheme 1g). Overall, this strategy would offer selective access to both stereoisomers of the target alkenylboronate esters from a single starting material. During the course of our study, Huang and Guo et al. have reported an elegant Fe–H-catalyzed isomerization resulting in trisubstituted (*Z*)-alkenylboronates (Scheme 1f),^41^ leading us to disclose our results herein. For the formation of (*E*)-alkenylboronates, our strategy relies on an Ir-based alkene isomerization catalyst operating through a 1,3-hydride shift mechanism.^35,42−50^ In this mechanistic scenario, the key allyl iridium hydride intermediate prefers a “W-shaped” conformation where the substituents at the termini of the allylic system point away from the bulky iridium center (**A**, Scheme 2a) rather than toward it (**B**). Reinsertion of the hydride would result in the formation of the *E*-alkenylboronate ester. Notably, overisomerization leading to allylboron species should be avoided due to the substitution pattern of the alkenylboronate substrates employed and the reluctance of the 1,3-hydride shift-based catalyst to generate allyliridium hydride intermediates featuring branching at the termini.

**Scheme 1:**
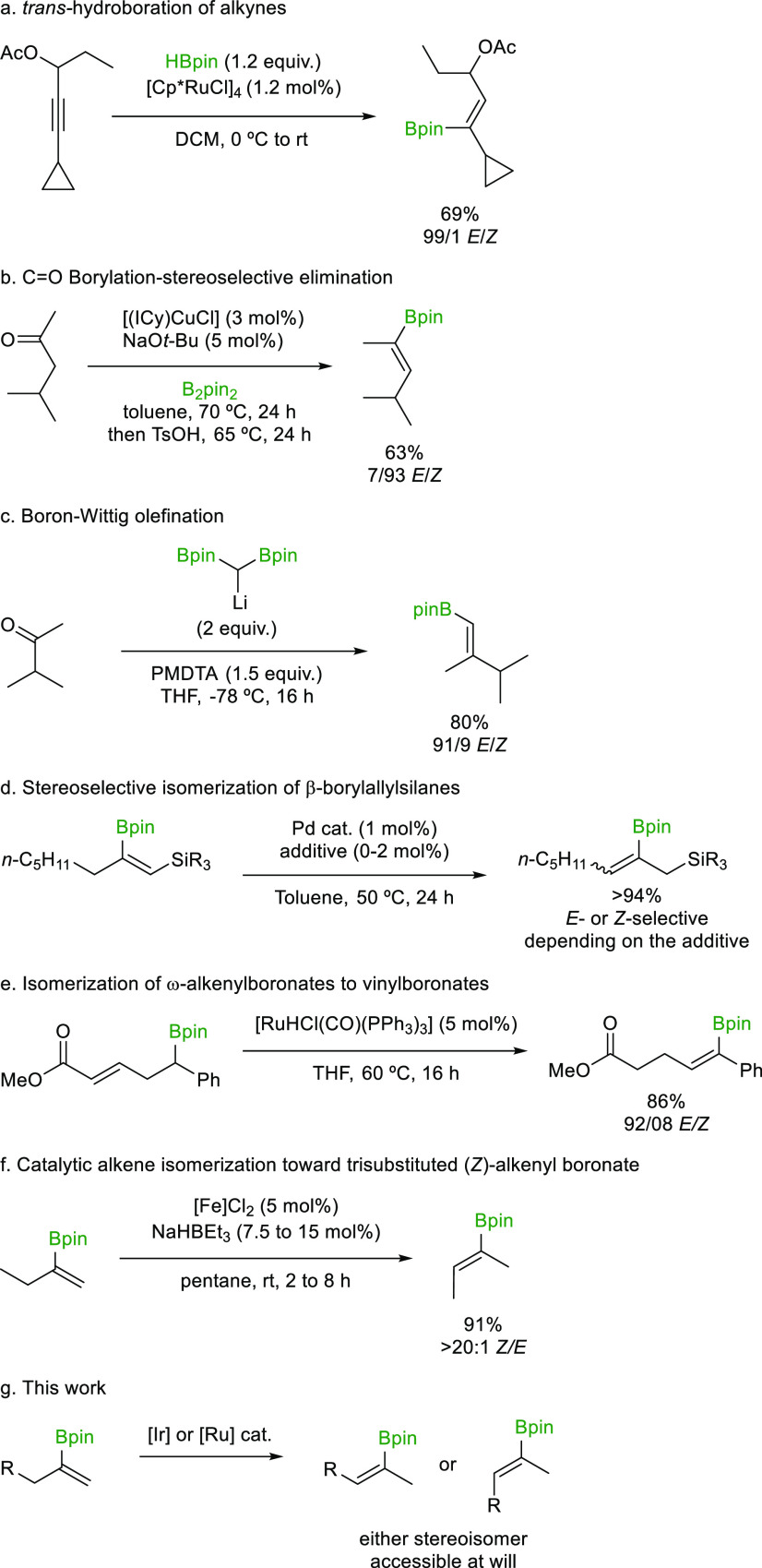
Stereoselective Preparation of Trisubstituted Alkenylboronate Esters

**Scheme 2:**
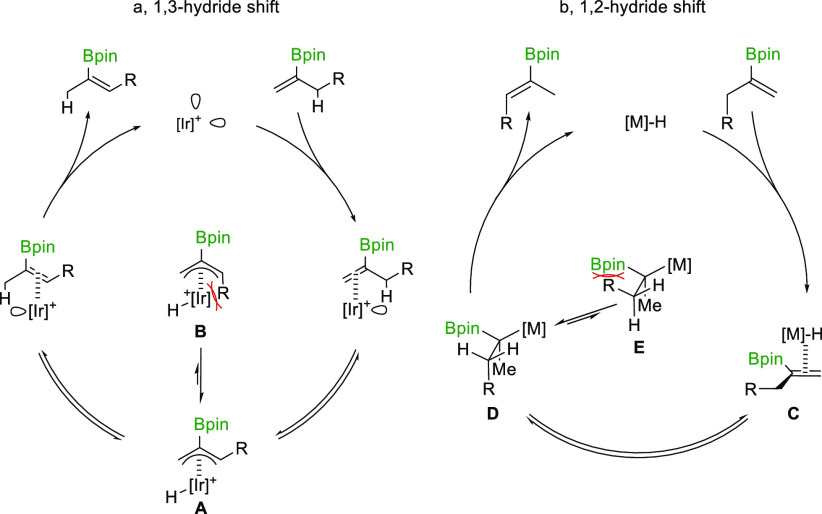
Stereodivergent Isomerization Based on Discrepant Mechanisms

Such an isomerization process that transiently generates reactive allylboronates has been extensively explored by the Murakami group and others,^51−60^ constituting an impressive application of alkene isomerization in stereoselective synthesis. Alternatively, a metal hydride catalyst that operates through a 1,2-hydride shift mechanism should afford the (*Z*)-alkenylboronate derivatives, achieving our goal of stereodivergence (Scheme 2b). Through this mechanism, selectivity would derive from the conformational preferences of the alkylmetal intermediate **D** over **E**. As depicted in Scheme 2b, the alkylmetal intermediate should favor a conformation where the bulky Bpin substituent avoids steric interactions with the R group, resulting in (*Z*)-selectivity.

To challenge the two hypotheses presented above, our model substrate **1a** (R = *n*-Pr), easily synthesized through the Ni-catalyzed hydroalumination-transmetalation sequence of alkynes developed by Hoveyda,^37^ was first submitted to our slightly modified Ir-based isomerization conditions (see Supporting Information).^35,47−49^

Hydrogenative activation of the catalyst to free the iridium of the chelating cyclooctadiene ligand prior to the addition of substrate **1a** proved to be necessary. Although the more sensitive precatalyst [Ir(coe)_2_Cl]_2_ (coe = cyclooctene) could be used to avoid the hydrogenation step, we decided to use the more stable and widely available [Ir(cod)Cl]_2_ as the precatalyst of choice for this study.

Before investigating the substrate scope of this transformation, we probed the functional group tolerance of the Ir-based catalyst by performing the isomerization of **1a** in the presence of various additives (see Supporting Information). With a clearer view of the functional groups tolerated by the Ir-catalyst, we prepared various alkenylboronates to explore the substrate scope of the reaction. We were pleased to observe that the reaction proceeds smoothly in most cases and that steric hindrance has little influence on the stereoselectivity (Scheme 3a, compare (*E*)-**2a** and (*E*)-**2c**), albeit requiring longer reaction times to isomerize sterically encumbered substrates. TBS-protected primary alcohols ((*E*)-**2d** and (*E*)-**2e**) and a primary alkyl chloride ((*E*)-**2f**) were well tolerated. Product (*E*)-**2g**, featuring an indole, was successfully formed with satisfactory yield and stereoselectivity after a slightly extended reaction time (3 h). Allyl-vinylboronate ester (*E*)-**2h** was efficiently and stereoselectively generated from the corresponding alkyne diboration product. Remarkably, (*E*)-**2i** is generated with minimal isomerization of the neighboring trisubstituted alkene into conjugation. Similarly, product (*E*)-**2j** is formed as two energetically degenerate isomers but without any detectable traces of conjugated isomers. Alkenylboronates (*E*)-**2k**–**2m**, featuring aromatic substituents of various electronic characters, were all smoothly prepared. All attempts to isomerize dialkenyl boronate ester **1n** failed, possibly due to chelation of the catalyst by the two alkenes, inhibiting productive isomerization (Scheme 3a). To challenge this hypothesis, substrates **1o** and **1p** were prepared, extending the tether by one and two methylene units, respectively. Their isomerization resulted in the desired products (**2o** and **2p**) in good yield and excellent stereoselectivity, demonstrating the feasibility of isomerization given enough separation between the targeted positions (Scheme 3a).

**Scheme 3:**
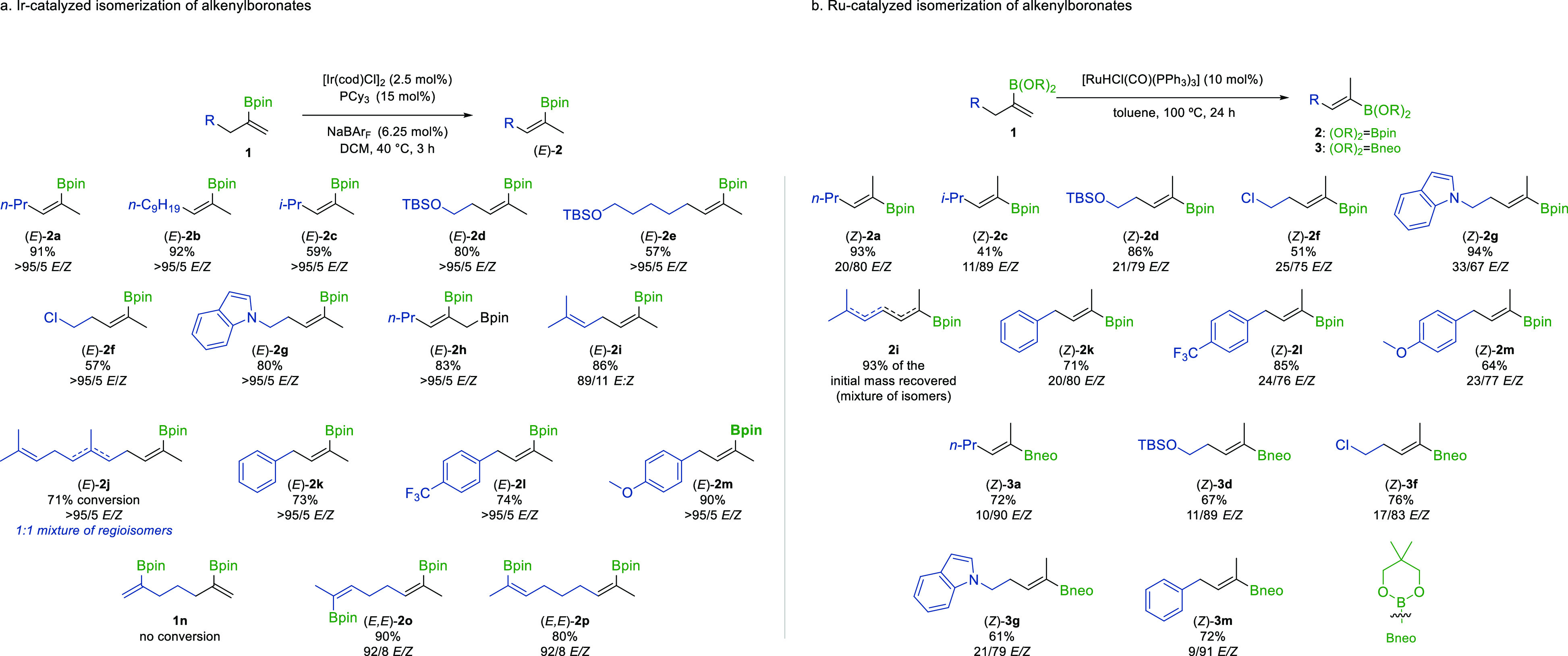
Substrate Scope for Ir- and Ru-Catalyzed Isomerization of Terminal Alkenylboronates

Having established reliable access to (*E*)-configured trisubstituted vinylboronate esters, we wanted to complement this strategy with a route toward the corresponding (*Z*)-isomers.

As discussed previously (Scheme 2b), alkene isomerization through the 1,2-hydride shift mechanism should provide this expected isomer. In this vein, we subjected our model substrate **1a** to the commercially available catalyst [RuHCl(CO)(PPh_3_)_3_], and after a brief optimization of the reaction conditions (solvent, temperature and time, see Supporting Information), we obtained the isomerized product (*Z*)-**2a** with 93% yield and a 20:80 *E*/*Z* ratio (Scheme 3b). A preliminary substrate scope for the Ru-catalyzed isomerization is presented in Scheme 3b. Linear and branched alkyl chains [(*Z*)-**2a** and (*Z*)-**2c**)] do not pose any challenges (see Supporting Information),^35,47−49^ and as anticipated, the stereoselectivity of the reaction increases with the steric demand of the substituent. Protected alcohol-containing product (*Z*)-**2d** can be obtained with minimal formation of the silyl enol ether side product resulting from overisomerization, provided the reaction is closely monitored. The introduction of a chloride maintained an acceptable transformation but unfortunately with a significant loss of selectivity (formation of (*Z*)-**2f**). Pleasingly, indole-containing (*Z*)-**2g** was smoothly generated with the Ru-based catalyst as well as products containing aryl groups of varied electronic nature (Scheme 3b, (*Z*)-**2k**–**m**). As expected, the lack of selectivity inherent to the metal hydride catalyst is manifested in the isomerization of polyenic substrates, as illustrated by the formation of **2i** as a mixture of isomers. A recent study by Aggarwal demonstrates the different steric properties of a range of boronic esters, with the counterintuitive conclusion that the Bneo ester is bulkier than its Bpin counterpart.^61^ Therefore, in an effort to improve the stereoselectivity, the larger Bneo alkenylboronates were synthesized and isomerized. The isomerization of the aforementioned Bneo variants resulted in significantly improved levels of stereoselectivity. However, it should be noted that such boronic esters (Bneo) are of lesser synthetic value compared to their Bpin counterparts, partaking in substantially fewer transformations.

Finally, we demonstrated the synthetic value of this method through a sequential isomerization-Suzuki-Miyaura cross-coupling process. Following Ir-catalyzed alkene isomerization, the crude reaction mixture was filtered, concentrated, and directly subjected to previously established cross-coupling reaction conditions, affording trisubstituted styrene products **4a** and **4b** in excellent yields and as single stereoisomers (Scheme 4). It should be noted that the filtration concentration step can be omitted. The cross-coupling partner and catalytic system can be directly added to the reaction mixture following the isomerization stage, affording product **4b** in 40% yield.

**Scheme 4:**
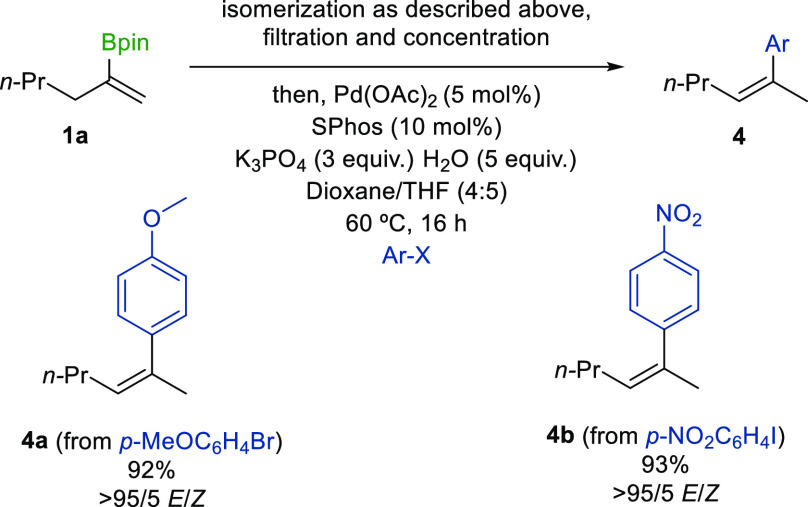
Sequential Isomerization-Suzuki-Miyaura Cross-Coupling

In conclusion, we have developed a stereodivergent strategy toward synthetically valuable trisubstituted alkenylboronate esters by alkene isomerization of their readily available 1,1-disubstituted regioisomers. Using an Ir-based catalytic system operating through a 1,3-hydride shift mechanism, excellent (*E*)-selectivity was obtained. Alternatively, a commercially available Ru–H catalyst provided the (*Z*)-configured alkenylboron compounds with varying degrees of selectivity. The (*E*)-selective Ir-based system complements the (*Z*)-selective Fe–H catalyst recently reported by Huang and Guo et al.^41^ Overall, this method illustrates the potential of alkene isomerization as an entry to highly substituted stereodefined alkenes.

## Supporting Information Available

Experimental procedures and spectral date (PDF)
