# Supplementary material for: Stereodivergent Access to Trisubstituted Alkenylboronate Esters through Alkene Isomerization
Source: Org Lett. 2021 Nov 12;23(23):9194–8. doi: 10.1021/acs.orglett.1c03513 (PMC8650100; doi:10.1021/acs.orglett.1c03513)
Supplement: Supplementary file 1 — ol1c03513_si_001.pdf [file ol1c03513_si_001.pdf]

Stereodivergent Access to Trisubstituted Alkenylboronate Esters Through Alkene Isomerization

Lucas Segura,‡ Itai Massad,‡ Masamichi Ogasawara† and Ilan Marek‡*

‡ Schulich Faculty of Chemistry, Technion – Israel Institute of Technology, Haifa 3200009, Israel.

† Department of Natural Science, Graduate School of Science and Technology. Tokushima University, Tokushima 770-8506, Japan.

Ilan Marek: chilanm@technion.ac.il

Table of Contents

Supplementary information 1

General 2

Starting materials 2

Functional group tolerance of the catalytic system 9

Optimisation of the [Ir]-catalyzed isomerization 8

Product of the [Ir]-catalyzed isomerization 10

Optimization of the isomerization condition using [Ru(HCl)COCl(PPh_3)$_4$] 13

Product isomerized with [Ru(HCl)COCl(PPh_3)$_4$] 15

Cross-coupling reactions 18

Determination of the stereochemistry 19

References 20

NMR spectra 22

General

Unless stated otherwise, chemical reactions were conducted in flame-dried glassware under a positive pressure of argon. Thin-layer chromatography (TLC) was conducted with E. Merck silica gel 60 F254 pre-coated plates, (0.25 mm) and visualized by exposure to UV light (254 nm) or stained with *p*-anisaldehyde stain or potassium permanganate. Column chromatography was performed using Fluka silica gel 60 Å (100-200 mesh) and Fluka Florisil® Adsorbent for Chromatography (40-63 mm, 230- 400 mesh), all boron-containing molecules were purified using silica gel treated with boric acid to mitigate product decomposition.[1] NMR spectra were recorded on Bruker spectrometer (AVIII400) and are reported relative to residual deuterated solvent signals. Chemical shifts are reported in parts per million (ppm) with respect to the residual solvent signal $CDCl_3$ (^{1}H NMR: δ = 7.26; ^{13}C {1H} NMR: δ = 77.00). Peak multiplicities are reported as follows: s = singlet, d = doublet, t = triplet, dd = doublet of doublets, td = triplet of doublets, m = multiplet. High-resolution mass spectra (HRMS) were obtained by the mass spectrometry facility at the Technion using a Q-TOF analyzer. Reactions were monitored by TLC (thin layer chromatography). THF and diethyl ether were purified from stabilizers and dried by distillation and subsequent filtration through flame-dried neutral alumina.[2] Toluene was distilled over sodium/benzophenone before use. All other commercially obtained reagents were used as received. All reactions were performed on 0.5 mmol scale and were carried out in Schlenk tubes heated via a fitted machined aluminum block placed on a heating plate. Larger scale reactions were heated using an oil bath.

Starting materials

General procedure for the preparation of terminal vinylboronate esters via an adapted procedure from Hoveyda's work.[3]

Commercial grade 1,3-bis(diphenylphosphino)propane nickel(II) chloride (Ni(dppp)Cl_2, 16.3 mg, 0.0300 mmol, 0.03 equiv.) is placed in an flame-dried 10 mL round bottom flask equipped with a stir bar and a refluxing condenser. The apparatus is sealed with a septum, flame dried, and kept under a positive pressure of argon at all times. Tetrahydrofuran (THF, 3.0 mL, aim for 0.33 M of starting material) is added through a syringe, followed by dropwise addition of DIBAL–H (232 µL, 1.30 mmol) at 22 °C (gas evolution occurs as DIBAL–H is added). The resulting black solution is cooled to 0 °C (ice bath) before phenylacetylene (110 µL, 1.00 mmol, 1 equiv.) is added slowly over five minutes (reaction is exothermic). The resulting black solution is allowed to warm to 22 °C and stir for an additional two hours. After two hours, the reaction mixture is cooled to 0 ºC and 2-methoxy-4,4,5,5-tetramethyl-1,3,2-dioxaborolane (MeO-Bpin; 492 µL, 3.00 mmol, 3 equiv.) is added dropwise through a syringe into the reaction solution. The resulting solution is refluxed for 24 hours before the reaction is quenched by dropwise addition of water (0.35 equiv.) at 0 °C (ice bath). The mixture is allowed to warm to 22 °C and stir for one additional hour before being filtered through a silica pad. After washing with a 10% diethyl ether solution in petroleum ether, the concentrated solution was purified by column chromatography on silica gel using a boric acid-treated silica.

tert-butyldimethyl(pent-4-yn-1-yloxy)silane

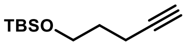

Prepared according to a literature report, NMR analysis being fully consistent with the report.[4] Purified by column chromatography (petroleum ether).

(5.65g, 95% yield, 30 mmol scale, colorless oil)

tert-butyldimethyl(non-8-yn-1-yloxy)silane

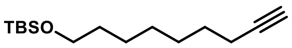

Prepared according to a literature report, NMR analysis being fully consistent with the report.[5] Purified by column chromatography (petroleum ether).

(7.33 g, 96% yield, 30 mmol scale, colorless oil)

1-(pent-4-yn-1-yl)indoline

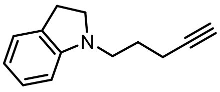

Prepared according to a literature report, NMR analysis being fully consistent with the report.[6] Purified by column chromatography (1/9 ethyl acetate/petroleum ether).

(4.17g, 75% yield, 30 mmol scale, colorless viscous oil)

5-methylhex-4-en-1-yne

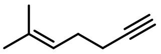

Prepared according to a literature report, NMR analysis being fully consistent with the report.[7] Purified by column chromatography (petroleum ether).

(1.47 g, 52%yield, 30 mmol scale, colorless oil)

(*E*)-5,9-dimethyldeca-4,8-dien-1-yne

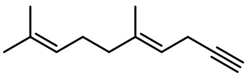

Prepared according to a literature report, NMR analysis being fully consistent with the report.[8] Purified by column chromatography (petroleum ether).

(1.7g, 35% yield, 30 mmol scale, yellow oil)

1-(but-3-yn-1-yl)-4-(trifluoromethyl)benzene

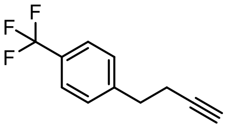

Prepared according to a literature report, NMR analysis being fully consistent with the report.[9] Purified by column chromatography (gradient 0 to 1/9 diethyl ether/petroleum ether).

(2.67 g, 45% yield, 30 mmol scale, yellow oil)

1-(but-3-yn-1-yl)-4-methoxybenzene

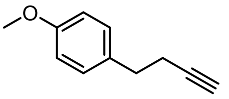

Prepared according to a literature report, NMR analysis being fully consistent with the report.[10] Purified by column chromatography (gradient 1/99 to 1/9 ethyl acetate/petroleum ether).

(2.5 g, 52% yield, 30 mmol scale, colorless viscous oil)

2-(hex-1-en-2-yl)-4,4,5,5-tetramethyl-1,3,2-dioxaborolane (1a)

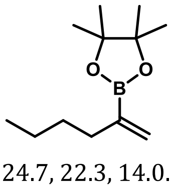

Prepared from 1-hexyne (693-02-7), purchased from Sigma-Aldrich. (2.5 g, 79%, 15 mmol scale, colorless oil). Purified by column chromatography (1/99 diethyl ether/petroleum ether).

^{1}H NMR (400 MHz, Chloroform-*d*) δ 5.74 (d, *J* = 3.5 Hz, 1H), 5.57 (d, *J* = 3.1 Hz, 1H), 2.13 (t, *J* = 7.5 Hz, 2H), 1.41 – 1.35 (m, 2H), 1.30 (t, *J* = 7.2 Hz, 2H), 1.25 (d, *J* = 1.5 Hz, 12H), 1.23 (d, *J* = 2.0 Hz, 2H), 0.90 – 0.85 (m, 3H). ^{13}C NMR (101 MHz, Chloroform-*d*) δ 128.6, 83.2, 35.0, 31.40, 24.8, 24.7, 22.3, 14.0. HRMS (APCI) m/z: [M + H]$^{+}$ calcd for $C_{12}H_{24}BO_2$ 211.1864; Found 211.1862.

2-(dodec-1-en-2-yl)-4,4,5,5-tetramethyl-1,3,2-dioxaborolane (1b)

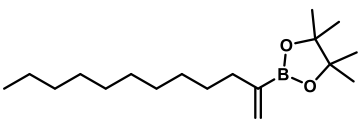

Prepared from 1-dodecyne (765-03-7) purchased from Sigma-Aldrich. (728 mg, 33% yield, 7.5mmol scale, yellow oil). Purified by column chromatography (1/99 diethyl ether/petroleum ether).

^{1}H NMR (400 MHz, chloroform-*d*) δ 5.75 (d, *J* = 3.5 Hz, 1H), 5.63 – 5.53 (m, 1H), 2.13 (t, *J* = 7.4 Hz, 2H), 1.40 (t, *J* = 7.9 Hz, 2H), 1.26 (d, *J* = 4.6 Hz, 36H), 0.87 (t, *J* = 6.6 Hz, 4H). ^{13}C NMR (101 MHz, Chloroform-*d*) δ 128.6, 83.3, 77.2, 35.3, 31.9, 29.7, 29.6, 29.4, 29.3, 29.2, 24.9, 24.8, 24.8, 22.7, 14.1. HRMS (APCI) m/z: [M + H]$^{+}$ calcd for $C_{18}H_{36}BO_2$ 295.2803; Found 295.2813.

4,4,5,5-tetramethyl-2-(4-methylpent-1-en-2-yl)-1,3,2-dioxaborolane (1c)

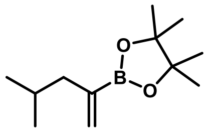

Prepared from 4-methyl-1-pentyne (7154-75-8), purchased from Sigma-Aldrich. (0,375g, 29.5% yield 6.1 mmol scale, yellow oil). Purified by column chromatography (1/99 diethyl ether/petroleum ether).

^{1}H NMR (400 MHz, Chloroform-*d*) δ 5.79 (d, *J* = 3.7 Hz, 1H), 5.55 (d, *J* = 3.5 Hz, 1H), 2.03 (d, *J* = 7.0 Hz, 2H), 1.74 (dq, *J* = 13.5, 6.7 Hz, 1H), 1.26 (s, 12H), 0.85 (d, *J* = 6.7 Hz, 6H). ^{13}C NMR (101 MHz, $CDCl_3$) δ 130.1, 83.2, 44.9, 28.0, 24.7, 22.4. HRMS (APCI) m/z: $[M + H]^+$ calcd for $C_{12}H_{24}BO_2$ 211.1864; Found 211.1876.

tert-butyldimethyl((4-(4,4,5,5-tetramethyl-1,3,2-dioxaborolan-2-yl)pent-4-en-1-yl)oxy)silane (1d)

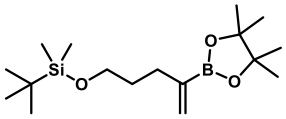

Prepared from tert-butyldimethyl(pent-4-yn-1-yloxy)silane. (2.9g, 46% yield, 19.15mmol scale, colorless oil). Purified by column chromatography (1/99 diethyl ether/petroleum ether).

^{1}H NMR (400 MHz, Chloroform-*d*) δ 5.76 (d, *J* = 3.5 Hz, 1H), 5.65 – 5.56 (m, 1H), 3.60 (t, *J* = 6.7 Hz, 2H), 2.17 (t, *J* = 7.7 Hz, 2H), 1.65 (dq, *J* = 9.5, 6.8 Hz, 2H), 1.26 (s, 12H), 0.89 (s, 9H), 0.04 (d, *J* = 0.7 Hz, 6H). ^{13}C NMR (101 MHz, $CDCl_3$) δ 129.1, 83.3, 77.3, 76.7, 62.9, 32.4, 31.6, 26.0, 24.8, 24.7, 18.3, -5.2. HRMS (APCI) m/z: $[M + H]^+$ calcd for $C_{17}H_{36}BO_3Si$ 327.2529; Found 327.2521.

tert-butyldimethyl((7-(4,4,5,5-tetramethyl-1,3,2-dioxaborolan-2-yl)oct-7-en-1-yl)oxy)silane (1e)

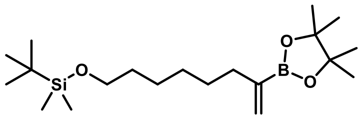

prepared from tert-butyldimethyl(non-8-yn-1-yloxy)silane (800mg, 22% yield, 10 mmol scale, yellow oil). Purified by column chromatography (1/99 diethyl ether/petroleum ether).

^{1}H NMR (400 MHz, Chloroform-*d*) δ 5.75 (d, *J* = 3.5 Hz, 1H), 5.64 – 5.52 (m, 1H), 3.59 (t, *J* = 6.6 Hz, 2H), 2.13 (t, *J* = 7.5 Hz, 2H), 1.49 (q, *J* = 6.7 Hz, 2H), 1.40 (q, *J* = 6.7 Hz, 2H), 1.35 – 1.28 (m, 4H), 1.26 (d, *J* = 1.2 Hz, 12H), 0.89 (d, *J* = 1.2 Hz, 9H), 0.04 (d, *J* = 1.2 Hz, 6H). ^{13}C NMR (101 MHz, $CDCl_3$) δ 128.7, 83.3, 63.3, 35.3, 32.8, 29.2, 29.0, 26.0, 25.7, 24.7, 18.4, -5.3. HRMS (APCI) m/z: $[M + H]^+$ calcd for $C_{20}H_{42}BO_3Si$ 369.2996; Found 369.3027.

2-(5-chloropent-1-en-2-yl)-4,4,5,5-tetramethyl-1,3,2-dioxaborolane (1f)

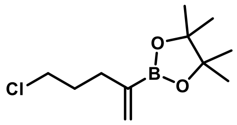

Prepared from 5-Chloro-1-pentyne (14267-92-6) purchased from Sigma-Aldrich. (1.26 g, 27%, 20 mmol scale, yellow oil). Purified by column chromatography (1/99 diethyl ether/petroleum ether).

^{1}H NMR (400 MHz, Chloroform-*d*) δ 5.71 (dd, *J* = 73.0, 2.8 Hz, 1H), 3.49 (t, *J* = 6.8 Hz, 1H), 2.26 (t, *J* = 7.4 Hz, 1H), 1.88 (p, *J* = 6.9 Hz, 1H), 1.23 (d, *J* = 7.6 Hz, 11H), 0.90 (d, *J* = 6.6 Hz, 1H). ^{13}C NMR (101 MHz, $CDCl_3$) δ 130.3, 83.5, 44.6, 32.6, 32.0, 25.3, 24.9, 24.9. HRMS (APCI) m/z: $[M + H]^+$ calcd for $C_{11}H_{21}BClO_2$ 231.1318; Found 231.1303.

1-(4-(4,4,5,5-tetramethyl-1,3,2-dioxaborolan-2-yl)pent-4-en-1-yl)-1H-indole (1g)

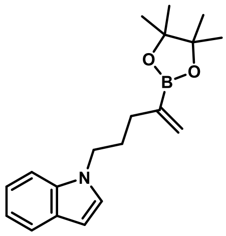

Prepared from 1-(pent-4-yn-1-yl)indoline (see alkyne preparation). (1.8g, 54% yield, 10.6 mmol scale, transparent viscous oil). Purified by column chromatography (1/9 diethyl ether/petroleum ether).

^{1}H NMR (400 MHz, Chloroform-*d*) δ 7.66 – 7.59 (m, 1H), 7.35 (dd, *J* = 8.3, 1.0 Hz, 1H), 7.19 (ddd, *J* = 8.3, 7.0, 1.2 Hz, 1H), 7.12 (d, *J* = 3.1 Hz, 1H), 7.09 (ddd, *J* = 8.0, 6.9, 1.1 Hz, 1H), 6.48 (dd, *J* = 3.2, 0.9 Hz, 1H), 5.84 (d, *J* = 3.3 Hz, 1H), 5.63 (d, *J* = 3.2 Hz, 1H), 4.14 – 4.07 (m, 2H), 2.22 (t, *J* = 7.4 Hz, 2H), 2.05 – 1.95 (m, 2H), 1.27 (s, 12H). ^{13}C NMR (101 MHz, $CDCl_3$) δ 135.9, 130.1, 128.5, 127.8, 121.2, 120.8, 119.1, 109.5, 100.7, 83.5, 45.9, 32.7, 29.4, 24.8, 1.0. HRMS (APCI) m/z: $[M + H]^+$ calcd for $C_{19}H_{27}BNO_2$ 312.2135; Found 312.2162.

(E)-2,2'-(hex-1-ene-1,2-diyl)bis(4,4,5,5-tetramethyl-1,3,2-dioxaborolane) (1h)

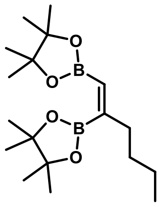

Prepared according to a literature report, NMR analysis being fully consistent with the report.[11] (832 mg, 20% yield, 13.7 mmol scale, yellow oil). Purified by column chromatography (5/95 diethyl ether/petroleum ether).

^{1}H NMR (400 MHz, Chloroform-*d*) δ 5.84 (s, 1H), 2.22 (td, *J* = 7.5, 1.5 Hz, 2H), 1.44 – 1.36 (m, 2H), 1.31 (m, 14H), 1.26 (s, 12H), 0.87 (t, *J* = 7.2 Hz, 3H). ^{13}C NMR (101 MHz, $CDCl_3$) δ 83.6, 83.2, 39.5, 30.7, 24.9, 24.8, 22.4, 13.9. HRMS (APCI) m/z: [M + H]$^+$ calcd for $C_{18}H_{35}B_2O_4$ 337.2721; Found 337.2745.

4,4,5,5-tetramethyl-2-(6-methylhepta-1,5-dien-2-yl)-1,3,2-dioxaborolane (1i)

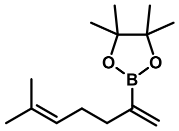

Prepared from 5-methylhex-4-en-1-yne (see alkyne preparation). (920 mg, 26% yield, 15 mmol scale, yellow oil). Purified by column chromatography (1/99 diethyl ether/petroleum ether).

^{1}H NMR (400 MHz, Chloroform-*d*) δ 5.75 (d, *J* = 3.5 Hz, 1H), 5.64 – 5.51 (m, 1H), 5.11 (ddt, *J* = 5.3, 3.9, 1.4 Hz, 1H), 2.20 – 2.12 (m, 2H), 2.08 (q, *J* = 6.8 Hz, 2H), 1.65 (d, *J* = 1.4 Hz, 3H), 1.58 (d, *J* = 1.4 Hz, 3H), 1.25 (s, 12H). ^{13}C NMR (101 MHz, $CDCl_3$) δ 131.3, 129.0, 124.3, 83.3, 35.6, 27.9, 25.7, 24.7, 17.7. HRMS (APCI) m/z: [M + H]$^+$ calcd for $C_{14}H_{26}BO_2$ 237.2020; Found 237.2037.

(*E*)-2-(6,10-dimethylundeca-1,5,9-trien-2-yl)-4,4,5,5-tetramethyl-1,3,2-dioxaborolane (1j)

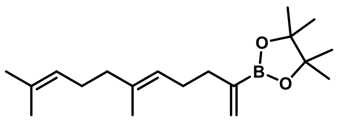

Prepared from (*E*)-5,9-dimethyldeca-4,8-dien-1-yne. (see alkyne preparation, 0,523g, 20% yield, 10 mmol scale, yellow oil). Purified by column chromatography (1/99 diethyl ether/petroleum ether).

^{1}H NMR (400 MHz, Chloroform-*d*) δ 5.75 (d, *J* = 3.5 Hz, 1H), 5.64 – 5.55 (m, 1H), 5.17 – 5.11 (m, 1H), 5.08 (tq, *J* = 5.5, 1.4 Hz, 1H), 2.17 – 2.13 (m, 2H), 2.11 (t, *J* = 7.0 Hz, 2H), 2.04 (t, *J* = 7.1 Hz, 2H), 2.00 – 1.94 (m, 2H), 1.66 (d, *J* = 1.5 Hz, 3H), 1.58 (d, *J* = 1.4 Hz, 6cH), 1.25 (s, 12H). ^{13}C NMR (101 MHz, $CDCl_3$) δ 135.0, 131.2, 129.1, 124.5, 83.3, 82.8, 39.8, 35.6, 27.8, 26.8, 25.7, 25.2, 24.8, 17.7, 16.0. HRMS (APCI) m/z: [M + H]$^+$ calcd for $C_{19}H_{34}BO_2$ 305.2646; Found 305.2645.

4,4,5,5-tetramethyl-2-(4-phenylbut-1-en-2-yl)-1,3,2-dioxaborolane (1k)

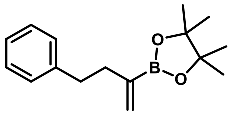

Prepared from but-3-yn-1-ylbenzene (16520-62-0) purchased from Sigma-Aldrich. (1.3 g, 50% yield, 10 mmol scale, yellow oil). Purified by column chromatography (1/99 diethyl ether/petroleum ether).

^{1}H NMR (400 MHz, Chloroform-*d*) δ 7.22 – 7.05 (m, 5H), 5.72 (dt, *J* = 3.5, 1.0 Hz, 1H), 5.53 (dd, *J* = 3.5, 1.9 Hz, 1H), 2.71 – 2.62 (m, 2H), 2.42 – 2.34 (m, 2H), 1.19 (s, 12H), 1.17 (s, 2H), 0.86 (d, *J* = 6.6 Hz, 1H). ^{13}C NMR (101 MHz, Chloroform-*d*) δ 142.6, 129.6, 128.7, 128.3, 125.7, 83.5, 37.4, 35.8, 24.9. HRMS (APCI) m/z: [M + H]$^+$ calcd for $C_{16}H_{24}BO_2$ 259.1869; Found 259.1871.

4,4,5,5-tetramethyl-2-(4-(4-(trifluoromethyl)phenyl)but-1-en-2-yl)-1,3,2-dioxaborolane (1l)

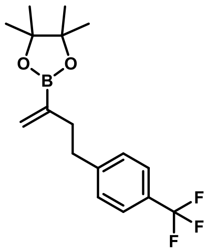

Prepared 1-(but-3-yn-1-yl)-4-(trifluoromethyl)benzene (see alkyne preparation, 1.9g,31.8% yield, 18.3 mmol scale, yellow viscous oil). Purified by column chromatography (5/95 diethyl ether/petroleum ether).

^{1}H NMR (400 MHz, Chloroform-*d*) δ ^{1}H NMR (400 MHz, Chloroform-*d*) δ 7.51 (d, *J* = 7.8 Hz, 3H), 7.28 (d, *J* = 8.0 Hz, 3H), 5.80 (d, *J* = 3.2 Hz, 1H), 5.63 – 5.55 (m, 1H), 2.80 (dd, *J* = 9.1, 6.6 Hz, 2H), 2.52 – 2.42 (m, 2H), 1.26 (s, 12H). ^{13}C NMR (101 MHz, $CDCl_3$) δ 130.0, 128.9, 125.1, 125.1, 83.5, 82.9, 36.9, 35.6, 25.3, 24.9, 24.8. HRMS (APCI) m/z: [M + H]$^+$ calcd for $C_{17}H_{23}BF_3O_2$ 327.1743; Found 327.1756.

2-(4-(4-methoxyphenyl)but-1-en-2-yl)-4,4,5,5-tetramethyl-1,3,2-dioxaborolane (1m)

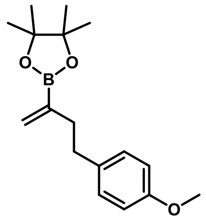

Prepared from 1-(but-3-yn-1-yl)-4-methoxybenzene (see alkyne preparation, 1.86g, 36% yield, 18 mmol scale, transparent viscous oil). Purified by column chromatography (5/95 diethyl ether/petroleum ether).

^{1}H NMR (400 MHz, Chloroform-*d*) δ 7.13 – 7.06 (m, 4H), 6.84 – 6.79 (m, 4H), 5.78 (dt, *J* = 3.4, 0.9 Hz, 1H), 5.61 – 5.56 (m, 1H), 3.79 (s, 2H), 3.78 (s, 1H), 3.78 (s, 3H), 2.71 – 2.65 (m, 2H), 2.45 – 2.39 (m, 2H), 1.27 (s, 12H). ^{13}C NMR (101 MHz, Chloroform-*d*) δ 129.6, 113.9, 113.8, 83.5, 55.4, 37.7, 35.0, 25.0, 24.9. HRMS (APCI) m/z: $[M + H]^+$ calcd for $C_{17}H_{26}BO_3$ 289.1970; Found 289.1993.

4,4,5,5-tetramethyl-2-(6-(3,3,4,4-tetramethylborolan-1-yl)hepta-1,6-dien-2-yl)-1,3,2-dioxaborolane (1n)

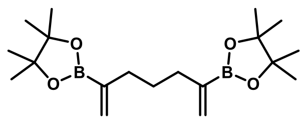

Prepared from hepta-1,6-diyne (2396-63-6), purchased from Sigma-Aldrich. (1.2g, 70% yield, 5 mmol scale, yellow oil). Purified by column chromatography (1/99 diethyl ether/petroleum ether).

^{1}H NMR (400 MHz, Chloroform-*d*) δ 5.75 (d, *J* = 3.6 Hz, 2H), 5.59 (d, *J* = 3.3 Hz, 2H), 2.14 (t, *J* = 7.6 Hz, 4H), 1.61 – 1.49 (m, 2H), 1.25 (s, 24H), 0.90 (dd, *J* = 10.8, 6.7 Hz, 2H). ^{13}C NMR (101 MHz, Chloroform-*d*) δ 129.0, 83.4, 35.2, 28.8, 24.9, 24.7. HRMS (APCI) m/z: $[M + H]^+$ calcd for $C_{19}H_{35}B_2O_4$ 349.2716; Found 349.2732.

2,2'-(octa-1,7-diene-2,7-diyl)bis(4,4,5,5-tetramethyl-1,3,2-dioxaborolane) (1o)

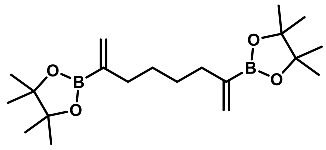

Prepared from octa-1,7-diyne (871-84-1), purchased from Sigma-Aldrich. (5.0 g, 92% yield, 15 mmol scale, colorless viscous oil). Purified by column chromatography (1/99 diethyl ether/petroleum ether).

^{1}H NMR (400 MHz, Chloroform-*d*) δ 5.75 (d, *J* = 3.5 Hz, 2H), 5.58 (d, *J* = 3.6 Hz, 2H), 2.14 (s, 4H), 1.42 (q, *J* = 4.2, 3.8 Hz, 4H), 1.26 (s, 26H), 0.92 (dd, *J* = 6.6, 1.3 Hz, 2H). ^{13}C NMR (101 MHz, $CDCl_3$) δ 128.7, 83.2, 35.2, 28.8, 24.8, 24.7. HRMS (APCI) m/z: $[M + H]^+$ calcd for $C_{20}H_{37}B_2O_4$ 363.2872; Found 363.2906.

4,4,5,5-tetramethyl-2-(6-(3,3,4,4-tetramethylborolan-1-yl)hepta-1,6-dien-2-yl)-1,3,2-dioxaborolane (1p)

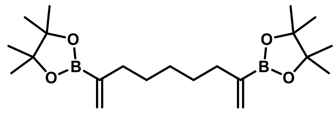

Prepared from hepta-1,6-diyne (2396-63-6), purchased from Sigma-Aldrich. (1.2g, 3.5 mmol on 5 mmol scale, colorless viscous oil). Purified by column chromatography (1/99 diethyl ether/petroleum ether).

^{1}H NMR (400 MHz, Chloroform-*d*) δ 5.74 (d, *J* = 3.5 Hz, 2H), 5.62 – 5.53 (m, 2H), 2.12 (t, *J* = 7.7 Hz, 4H), 1.47 – 1.34 (m, 4H), 1.33 – 1.27 (m, 2H), 1.26 (s, 23H), 1.24 (s, 3H). ^{13}C NMR (101 MHz, $CDCl_3$) δ 128.6, 83.2, 35.3, 29.1, 24.7. HRMS (APCI) m/z: $[M + H]^+$ calcd for $C_{21}H_{39}B_2O_4$ 377.3028; Found 377.3029.

All of the vinyl neopentyl glycol boronates have been prepared according to the methodology reported in the literature.[12] The starting materials used are the alkenyl boronate ester reported above.

2-(hex-1-en-2-yl)-5,5-dimethyl-1,3,2-dioxaborinane

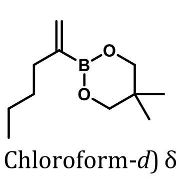

(264mg, 27% yield, 5 mmol scale, yellow oil) Purified by column chromatography (1/99 diethyl ether/petroleum ether).

^{1}H NMR (400 MHz, Chloroform-*d*) δ 5.69 (d, *J* = 3.8 Hz, 1H), 5.55 – 5.44 (m, 1H), 3.63 (s, 4H), 2.18 – 2.05 (m, 2H), 1.40 – 1.22 (m, 4H), 0.96 (s, 6H), 0.92 – 0.88 (m, 3H). ^{13}C NMR (101 MHz, Chloroform-*d*) δ 126.5, 72.2, 34.8, 31.8, 25.5, 22.6, 22.0, 22.0, 14.2. HRMS (APCI) m/z: $[M + H]^+$ calcd for $C_{11}H_{22}BO_2$ 197.1713; Found 197.1706.

tert-butyl((4-(5,5-dimethyl-1,3,2-dioxaborinan-2-yl)pent-4-en-1-yl)oxy)dimethylsilane

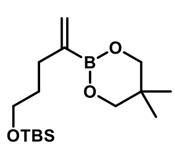

(420 mg, 23% yield, 5.8 mmol scale, yellow oil). Purified by column chromatography (1/99 diethyl ether/petroleum ether).

^{1}H NMR (400 MHz, Chloroform-*d*) δ 5.71 (d, *J* = 3.8 Hz, 1H), 5.58 – 5.45 (m, 1H), 3.62 (d, *J* = 1.5 Hz, 4H), 3.61 – 3.56 (m, 2H), 2.13 (t, *J* = 7.8 Hz, 2H), 1.62 (h, *J* = 7.0 Hz, 2H), 0.95 (s, 6H), 0.89 (s, 9H), 0.04 (s, 6H). ^{13}C NMR (101 MHz, $CDCl_3$) δ 151.3, 126.8, 72.0, 63.1, 62.6, 32.6, 31.6, 26.0, 21.8, 18.3, -5.2. HRMS (APCI) m/z: $[M + H]^+$ calcd for $C_{16}H_{34}BO_3Si$ 313.2370; Found 313.2359.

2-(5-chloropent-1-en-2-yl)-5,5-dimethyl-1,3,2-dioxaborinane

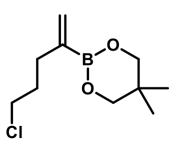

(280 mg, 26% yield, 5 mmol scale, yellow oil). Purified by column chromatography (1/99 diethyl ether/petroleum ether).

^{1}H NMR (400 MHz, Chloroform-*d*) δ 5.77 (d, *J* = 3.6 Hz, 1H), 5.63 – 5.51 (m, 1H), 3.64 (s, 4H), 3.52 (t, *J* = 6.8 Hz, 2H), 2.24 (dd, *J* = 8.2, 6.6 Hz, 2H), 1.89 (dq, *J* = 8.9, 6.9 Hz, 2H), 0.96 (s, 6H). ^{13}C NMR (101 MHz, $CDCl_3$) δ 128.0, 72.1, 44.8, 32.2, 31.6, 25.3, 24.7, 21.8. HRMS (APCI) m/z: $[M + H]^+$ calcd for $C_{10}H_{19}BClO_2$ 217.1167; Found 217.1154.

s**1-(4-(5,5-dimethyl-1,3,2-dioxaborinan-2-yl)pent-4-en-1-yl)-1H-indole**

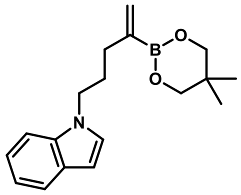

(237 mg, 16% yield, 5 mmol scale, yellow oil). Purified by column chromatography (5/95 diethyl ether/petroleum ether).

^{1}H NMR (400 MHz, Chloroform-*d*) δ 7.62 (d, *J* = 7.8 Hz, 1H), 7.35 (d, *J* = 8.2 Hz, 1H), 7.19 (t, *J* = 7.6 Hz, 1H), 7.14 – 7.05 (m, 2H), 6.48 (d, *J* = 3.1 Hz, 1H), 5.78 (d, *J* = 3.5 Hz, 1H), 5.53 (s, 1H), 4.10 (t, *J* = 7.3 Hz, 2H), 3.63 (d, *J* = 1.2 Hz, 4H), 2.18 (t, *J* = 7.5 Hz, 2H), 1.98 (p, *J* = 7.5 Hz, 2H), 0.96 (d, *J* = 1.3 Hz, 6H). ^{13}C NMR (101 MHz, $CDCl_3$) δ 135.9, 128.5, 127.8, 121.2, 120.8, 119.0, 109.5, 72.1, 65.8, 46.0, 32.3, 31.6, 29.6, 21.8, 15.3, 15.2. HRMS (APCI) m/z: $[M + H]^+$ calcd for $C_{18}H_{25}BNO_2$ 298.1978; Found 298.2007.

5,5-dimethyl-2-(4-phenylbut-1-en-2-yl)-1,3,2-dioxaborinane

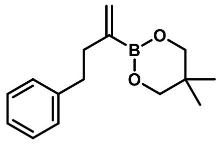

(268.5 mg, 22% yield, 5 mmol scale, yellow oil). Purified by column chromatography (1/99 diethyl ether/petroleum ether).

^{1}H NMR (400 MHz, Chloroform-*d*) δ 7.27 (ddd, *J* = 24.7, 15.0, 7.2 Hz, 5H), 5.81 (d, *J* = 3.6 Hz, 1H), 5.64 – 5.53 (m, 1H), 3.68 (s, 4H), 2.78 (dd, *J* = 9.6, 6.5 Hz, 2H), 2.49 (dd, *J* = 9.7, 6.5 Hz, 2H), 1.01 (s, 6H). ^{13}C NMR (101 MHz, $CDCl_3$) δ 142.7, 128.5, 128.1, 127.1, 125.5, 72.0, 36.9, 36.0, 31.6, 25.3, 21.8, 21.8. HRMS (APCI) m/z: $[M + H]^+$ calcd for $C_{15}H_{22}BO_2$ 245.1713; Found 245.1745.

Optimization of the Ir-catalyzed isomerization

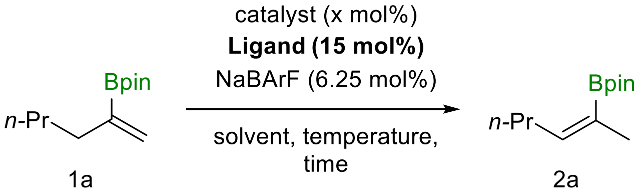

Entry	Conditions	Ligand	Catalyst activation	Yield[a]	*E/Z*[b]
1	$[Ir(cod)Cl]_2$ (2.5 mol%), DCE, 80 °C, 12 h	PCy_3	[H][c]	91%	93/7
2	$[Ir(cod)Cl]_2$ (2.5 mol%), DCE, 80 °C, 12 h	PCy_3	None	15%	> 95/5
3	$[Ir(coe)_2Cl_2]$ (5 mol%), DCE, 80 °C, 12 h	PCy_3	None	92%	93/7
4	$[Ir(cod)_2(BF_4)]$ (5 mol%), DCE, 80 °C, 12 h	PCy_3	[H][c]	54%	> 95/5
5	$[Ir(cod)Cl]_2$ (2.5 mol%), DCE, 80 °C, 12 h	PMe_3	[H][c]	52%	31/69
6	$[Ir(cod)Cl]_2$ (2.5 mol%), DCE, 80 °C, 12 h	P(*n*-Bu)$_3$	[H][c]	92%	74/26
7	$[Ir(cod)Cl]_2$ (2.5 mol%), PhCl, 132 °C, 12 h	PCy_3	[H][c]	75%	95/5
**8**	**$[Ir(cod)Cl]_2$ (2.5 mol%), DCM, 40 °C, 3 h**	**PCy_3**	**[H][c]**	**91%**	**> 95/5**
9	$[Ir(cod)Cl]_2$ (1 mol%),[d] DCM, 40 °C, 1 h	PCy_3	[H][c]	90%	> 95/5

[a]Yields determined by ^1H NMR of the crude reaction mixture. [b]Ratio determined by ^1H NMR analysis of the crude reaction mixture. [c]Hydrogenative activation of the catalytic system before the addition of the substrate. [d] 6 mol% PCy_3, 2.6 mol% $NaBAr_F$.

The reaction proceeded smoothly with the initial conditions: the activation of the catalyst appeared mandatory as a significant reduction of the activity is expected otherwise (entry 2). Using $[Ir(coe)_2Cl_2]$ as a catalyst avoided the need for activation while retaining performance. The addition of $NaBAr_F$ in the reaction mixture is necessary to abstract the chloride anion from the iridium and free one of the necessary coordination sites enabling the 1,3-

hydride mechanism. In entry 4, [Ir(cod)$_2$BF$_4$] is employed, this catalyst does not need any anion abstractor as the BF_4^- anion coordinates the iridium to a weaker extent, therefore allowing catalysis. Yet the two aforementioned catalysts suffer air stability shortcomings and should be handled in a glove box or using Shlenk technique. As explained in the introduction, it is accepted that the preferable conformer for the allyl intermediate is one having the substituents pointing away from the metal. A reduction in the ligand steric parameter (PMe_3, entry 5) resolves in the preferred generation of the opposite *Z* isomer, albeit with poor stereoselectivity. The increase in the steric bulk achieved by preferring P(*n*-Bu)$_3$ as a ligand is sufficient to generate the *E*-isomer as the major product with a low selectivity as compared with the PCy_3 based system, supporting the hypothesis discussed above. The system sustains reactivity and selectivity at higher temperatures, even for 12 h (entry 7). Such an increase in temperature is not necessary to shorten the reaction time as the reaction can proceed to completion within 1 h at 40 ºC with only 1 mol% of the iridium dimer. This reduction in temperature allowed the use of DCM as a solvent instead of 1,2-DCE. The optimal conditions chosen for the scope of this reaction settles for 3 h reaction time and the usual 5 mol% of iridium. This ensures reliable conversion of the substrates within the expected time frame regardless of functional groups present on the substrate.

Functional group tolerance of the catalytic system

Before investigating the substrate scope of the reaction, we decided to first probe the functional group tolerance of the Ir-based catalyst by performing the isomerization of **1a** in the presence of various additives. Water is well tolerated, even in relatively large amounts, possibly due to its low miscibility with DCM. Similarly, alcohols of various substitution patterns do not strongly interfere with isomerization. While acetone is also compatible with the Ir-catalyst, cyclohexanone, surprisingly, obstructs isomerization to a considerable extent (only 17% conversion). Other carbonyl compounds have variable effects on the catalytic system, depending on both their steric and electronic properties. Esters and carbonates are well tolerated, while the more coordinating amides, ureas and carboxylic acids are not. Protected carbonyls, namely aldehyde-derived acetals, do not inhibit the catalyst. Unsurprisingly, Lewis-basic tertiary amines and nitriles completely preclude isomerization, as do terminal and internal alkynes

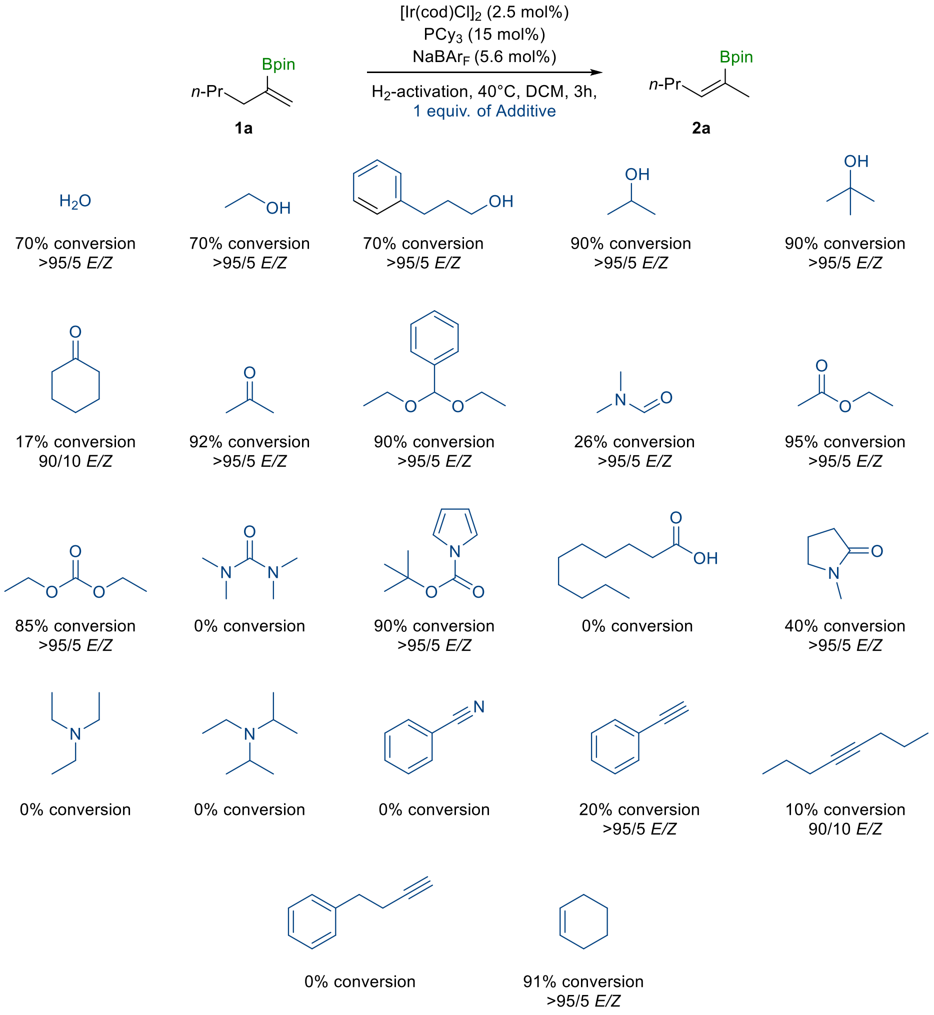

Products of the Ir-catalyzed isomerization

General procedure for the Iridium catalyzed alkenylboronic ester mono-isomerization

Sodium tetrakis[3,5-bis(trifluoromethyl)phenyl]borate (10 mg, 0.011 mmol, 5.6 mol%) is placed in a flame-dried Schlenk tube under argon flow. PCy_3 (8.41 mg, 0.03 mmol, 15 mol%) and $[Ir(cod)Cl]_2$ (3.36 mg, 0.005 mmol, 2.5 mol%) are subsequently added under a flow of argon and the solids stirred for 1 minute at room temperature before DCM (1 mL) is added. Upon complete dissolution of the solids (resulting in an orange-reddish solution), hydrogen gas is bubbled through a needle for 1 minute, adding DCM at the end of this hydrogenation step can be necessary to conserve the desired volume of solvent. Subsequently, the reaction is degassed by performing 3 freeze-pump-thaw cycles. The substrate (0.2 mmol) is added neat and the resulting mixture is allowed to stir at 40 ºC. Upon consumption of the starting material (usually 1 hour, conversion was monitored by TLC: petroleum ether/diethyl ether eluent system (20/1), visualized with anisaldehyde. The isomerized boronic esters are typically 0.05 units higher in Rf than the starting material). The mixture is then allowed to cool down to room temperature, DCM is removed in vacuo, and the resulting crude product is purified by column chromatography (Et2O/petroleum ether).

Note: the quality of the Ir-precatalyst often affects the reaction rate, with the best reactivity obtained with $[Ir(cod)Cl]_2$ purchased from Strem.

(*E*)-2,2'-(hex-1-ene-1,2-diyl)bis(4,4,5,5-tetramethyl-1,3,2-dioxaborolane) ((*E*)-2a)

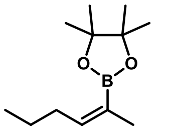

(91% isolated yield, 95.5 mg, *E/Z*>5/95, 0.5mmol scale, colorless oil). Purified by column chromatography (1/99 diethyl ether/petroleum ether).

^{1}H NMR (400 MHz, Chloroform-*d*) δ 5.81 (t, *J* = 1.5 Hz, 1H), 2.24 – 2.13 (m, 2H), 1.41 – 1.33 (m, 3H), 1.28 (s, 12H), 1.23 (s, 12H), 0.87 – 0.80 (m, 6H). ^{13}C NMR (101 MHz, Chloroform-*d*) δ 147.2, 82.9, 33.2, 25.0, 23.2, 22.5, 13.8. HRMS (APCI) m/z: [M + H]$^+$ calcd for $C_{12}H_{24}BO_2$ 211.1864; Found 211.1873.

(*E*)-2-(dodec-2-en-2-yl)-4,4,5,5-tetramethyl-1,3,2-dioxaborolane ((*E*)-2b)

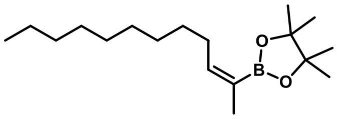

(51mg, *E/Z*>95/5, 92% yield on 0.2 mmol, yellow oil). Purified by column chromatography (1/99 diethyl ether/petroleum ether).

^{1}H NMR (400 MHz, Chloroform-*d*) δ 6.10 – 5.99 (m, 1H), 2.33 – 2.24 (m, 2H), 1.74 (d, *J* = 1.4 Hz, 3H), 1.26 (m, 26H), 0.89 – 0.85 (m, 3H). ^{13}C NMR (101 MHz, Chloroform-*d*) δ 147.6, 82.9, 32.1, 31.2, 30.1, 29.8, 29.7, 29.5, 29.3, 25.0, 22.8, 22.5, 14.2. HRMS (APCI) m/z: [M + H]$^+$ calcd for $C_{18}H_{36}BO_2$ 295.2808; Found 295.2808.

(*E*)-4,4,5,5-tetramethyl-2-(4-methylpent-2-en-2-yl)-1,3,2-dioxaborolane ((*E*)-2c)

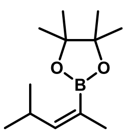

(24.8mg, 59% yield, *E/*Z>95:5, 0.2mmol scale, colorless oil). Purified by column chromatography (1/99 diethyl ether/petroleum ether).

Larger scale isomerization: 106.0 mg isolated, 50% yield, 1 mmol scale (petroleum ether).

^{1}H NMR (400 MHz, Chloroform-*d*) δ 6.10 – 5.99 (m, 1H), 2.33 – 2.24 (m, 2H), 1.74 (d, *J* = 1.4 Hz, 3H), 1.26 (s, 28H), 0.89 – 0.85 (m, 4H). ^{13}C NMR (101 MHz, Chloroform-*d*) δ 154.7, 82.9, 29.8, 24.9, 24.8, 23.6, 22.4. HRMS (APCI) m/z: [M + H]$^+$ calcd for $C_{12}H_{24}BO_2$ 211.1864; Found 211.1872.

(*E*)-tert-butyldimethyl((4-(4,4,5,5-tetramethyl-1,3,2-dioxaborolan-2-yl)pent-3-en-1-yl)oxy)silane ((*E*)-2d)

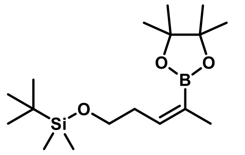

(61 mg recovered, 80%yield, *E/*Z>95:5, 0.2mmol scale, colorless oil). Purified by column chromatography (1/99 diethyl ether/petroleum ether).

^{1}H NMR (400 MHz, Chloroform-*d*) δ 6.07 (t, *J* = 7.6 Hz, 1H), 3.61 (td, *J* = 7.1, 1.3 Hz, 2H), 2.56 (q, *J* = 7.2 Hz, 2H), 1.76 (s, 3H), 1.26 (s, 13H), 0.89 (s, 10H), 0.05 (d, *J* = 1.3 Hz, 6H). ^{13}C NMR (101 MHz, $CDCl_3$) δ 143.1, 82.8, 63.5, 34.9, 26.0, 24.8, 22.4, 18.4, -5.2. HRMS (APCI) m/z: [M + H]$^+$ calcd for $C_{17}H_{36}BO_3Si$ 327.2521; Found 327.2536.

(*E*)-tert-butyldimethyl((7-(4,4,5,5-tetramethyl-1,3,2-dioxaborolan-2-yl)oct-6-en-1-yl)oxy)silane ((*E*)-2e)

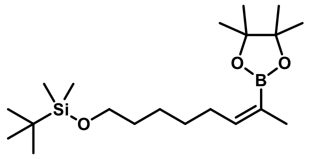

(57% yield, 105 mg, *E/Z*>95:5, 0.5 mmol scale, colorless oil). Purified by column chromatography (1/99 diethyl ether/petroleum ether).

^{1}H NMR (400 MHz, Chloroform-*d*) δ 6.05 (td, *J* = 7.5, 4.1 Hz, 1H), 3.59 (t, *J* = 6.6 Hz, 2H), 2.39 – 2.24 (m, 2H), 1.74 (d, *J* = 1.4 Hz, 3H), 1.52 (p, *J* = 6.7 Hz, 2H), 1.38 – 1.28 (m, 4H), 1.26 (s, 12H), 0.89 (s, 9H), 0.04 (s, 6H). ^{13}C NMR (101 MHz, Chloroform-*d*) δ 147.4, 82.9, 63.4, 32.9, 31.2, 29.9, 26.1, 25.5, 25.0, 22.5, 18.5, -5.1. HRMS (APCI): m/z calculated for [M+H]$^+$:; found. HRMS (APCI) m/z: [M + H]$^+$ calcd for $C_{20}H_{42}BO_3Si$ 369.2991; Found 369.3013.

(*E*)-2-(5-chloropent-2-en-2-yl)-4,4,5,5-tetramethyl-1,3,2-dioxaborolane ((*E*)-2f)

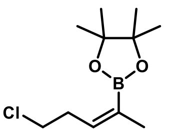

(36 mg recovered, 57% *E/Z*>95/5, 0.2 mmol scale, yellow oil). Purified by column chromatography (1/99 diethyl ether/petroleum ether).

^{1}H NMR (400 MHz, Chloroform-*d*) δ 6.08 (t, *J* = 7.4 Hz, 1H), 3.53 (t, *J* = 7.0 Hz, 2H), 2.79 (qd, *J* = 7.1, 1.2 Hz, 2H), 1.79 (d, *J* = 1.4 Hz, 3H), 1.27 (s, 12H). ^{13}C NMR (101 MHz, $CDCl_3$) δ 141.8, 83.1, 44.9, 34.1, 29.7, 24.8, 22.4. HRMS (APCI) m/z: [M + H]$^+$ calcd for $C_{11}H_{21}BClO_2$ 231.1323; Found 231.1314.

(*E*)-1-(4-(4,4,5,5-tetramethyl-1,3,2-dioxaborolan-2-yl)pent-3-en-1-yl)-1H-indole ((*E*)-2g)

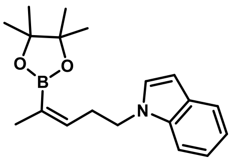

(54 mg recovered, 80%yield, E/Z>95:5, 0.2mmol scale, yellow viscous oil). Purified by column chromatography (5/95 diethyl ether/petroleum ether).

^{1}H NMR (400 MHz, Chloroform-*d*) δ 7.64 (dt, *J* = 7.8, 1.1 Hz, 1H), 7.49 (dd, *J* = 8.2, 1.1 Hz, 1H), 7.22 (ddd, *J* = 8.2, 7.0, 1.2 Hz, 1H), 7.16 – 7.08 (m, 2H), 6.49 (dd, *J* = 3.1, 0.9 Hz, 1H), 6.16 – 6.08 (m, 1H), 4.18 – 4.11 (m, 2H), 2.93 – 2.82 (m, 2H), 1.80 (s, 3H), 1.27 (s, 12H). ^{13}C NMR (101 MHz, Chloroform-*d*) δ 142.4, 128.0, 121.2, 120.9, 119.2, 109.9, 100.8, 83.2, 46.9, 32.3, 25.0, 22.6. HRMS (APCI) m/z: [M + H]$^+$ calcd for $C_{19}H_{27}BNO_2$ 312.2135; Found 312.2135.

(*E*)-2,2'-(hex-2-ene-1,2-diyl)bis(4,4,5,5-tetramethyl-1,3,2-dioxaborolane) ((*E*)-2h)

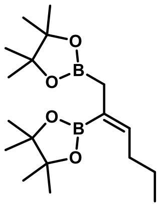

(59 mg recovered, 83%yield, *E/Z*>95:5, 0.2 mmol scale, yellow oil). Purified by column chromatography (1/99 diethyl ether/petroleum ether).

^{1}H NMR (400 MHz, Chloroform-*d*) δ 5.96 (t, *J* = 7.6 Hz, 1H), 2.28 (q, *J* = 7.4 Hz, 2H), 1.72 (s, 2H), 1.38 – 1.32 (m, 2H), 1.25 (s, 12H), 1.21 (s, 12H), 0.86 (t, *J* = 7.4 Hz, 3H). ^{13}C NMR (101 MHz, Chloroform-*d*) δ 145.9, 83.0, 82.9, 33.2, 25.0, 25.0, 25.0, 24.9, 23.3, 13.8. HRMS (APCI) m/z: [M + H]$^+$ calcd for $C_{18}H_{35}B_2O_4$ 337.2721; Found 337.2756.

(*E*)-4,4,5,5-tetramethyl-2-(6-methylhepta-2,5-dien-2-yl)-1,3,2-dioxaborolane ((*E*)-2i)

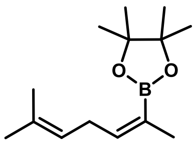

(46 mg recovered, 86% yield, *E/Z*>95:5, 0.2 mmol scale, yellow oil). Purified by column chromatography (1/99 diethyl ether/petroleum ether).

^{1}H NMR (400 MHz, Chloroform-*d*) δ 6.03 – 5.96 (m, 1H), 5.19 – 5.06 (m, 1H), 3.09 – 2.97 (m, 2H), 1.75 (d, *J* = 1.4 Hz, 3H), 1.69 (d, *J* = 1.4 Hz, 3H), 1.65 (d, *J* = 1.3 Hz, 3H), 1.27 (s, 12H). ^{13}C NMR (101 MHz, $CDCl_3$) δ 145.5, 131.7, 122.9, 82.8, 30.6, 25.8, 24.8, 24.8, 24.8, 24.8, 22.3, 17.7. HRMS (APCI) m/z: [M + H]$^+$ calcd for $C_{14}H_{26}BO_2$ 237.2026; Found 237.2048.

(*E*)-2-(6,10-dimethylundeca-2,9-dien-2-yl)-4,4,5,5-tetramethyl-1,3,2-dioxaborolane ((*E*)-2j)

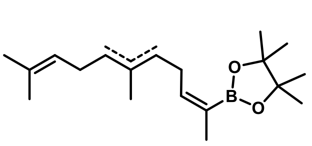

(60 mg recovered, 71% yield, mixture of isomers, *E/Z*>95:5, 0.2 mmol scale, yellow oil). Purified by column chromatography (1/99 diethyl ether/petroleum ether).

^{1}H NMR (400 MHz, Chloroform-*d*) δ 5.97 – 5.86 (m, 1H), 5.11 – 4.95 (m, 3H), 2.98 (t, *J* = 7.5 Hz, 2H), 2.37 – 2.30 (m, 1H), 2.02 – 1.96 (m, 3H), 1.91 (dd, *J* = 9.3, 5.8 Hz, 3H), 1.68 (s, 3H), 1.61 (d, *J* = 1.5 Hz, 3H), 1.57 (s, 3H), 1.52 (s,3H), 1.20 (s, 12H). ^{13}C NMR (101 MHz, Chloroform-*d*) δ 145.7, 135.4, 124.5, 122.9, 83.0, 82.9, 39.9, 30.6, 26.8, 25.8, 25.4, 25.0, 24.97, 24.95, 24.9, 22.5, 17.8, 16.2. HRMS (APCI) m/z: [M + H]$^+$ calcd for $C_{19}H_{34}BO_2$ 305.2646; Found 305.2662.

(*E*)-4,4,5,5-tetramethyl-2-(4-phenylbut-2-en-2-yl)-1,3,2-dioxaborolane ((*E*)-2k)

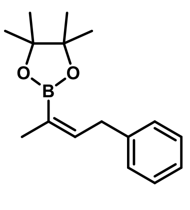

(44 mg, 73% yield, *E/Z*>5:95 on 0.2mmol scale, colorless oil) . Purified by column chromatography (1/99 diethyl ether/petroleum ether).

^{1}H NMR (400 MHz, Chloroform-*d*) δ 7.29 (d, *J* = 7.4 Hz, 1H), 7.24 – 7.15 (m, 3H), 6.19 (t, *J* = 7.6 Hz, 1H), 3.70 (d, *J* = 7.7 Hz, 2H), 1.79 (d, *J* = 1.4 Hz, 3H), 1.30 (s, 12H). ^{13}C NMR (101 MHz, $CDCl_3$) δ 145.2, 141.5, 128.6, 128.3, 125.7, 83.0, 37.6, 24.9, 22.3. HRMS (APCI) m/z: [M + H]$^+$ calcd for $C_{16}H_{24}BO_2$ 259.1869; Found 259.1848.

(*E*)-4,4,5,5-tetramethyl-2-(4-(4-(trifluoromethyl)phenyl)but-2-en-2-yl)-1,3,2-dioxaborolane ((*E*)-2l)

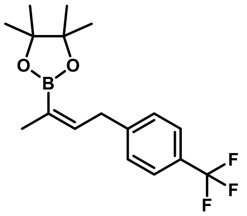

(48.7 mg, 74% yield, *E/Z*>95:5 on 0,2mmol scale, yellow oil). Purified by column chromatography (5/95 diethyl ether/petroleum ether).

^{1}H NMR (400 MHz, Chloroform-*d*) δ 7.52 (d, *J* = 7.9 Hz, 2H), 7.32 (d, *J* = 7.9 Hz, 2H), 6.23 – 6.07 (m, 1H), 3.74 (d, *J* = 7.7 Hz, 2H), 1.80 (d, *J* = 1.4 Hz, 3H), 1.30 (s, 12H). ^{13}C NMR (101 MHz, Chloroform-*d*) δ 145.7, 143.8, 128.8, 125.2 (q, *J* = 3.7 Hz), 83.1, 37.3, 24.8, 22.3. HRMS (APCI) m/z: [M + H]$^+$ calcd for $C_{17}H_{23}BF_3O_2$ 327.1738; Found 327.1718.
ss

(*E*)-2-(4-(4-methoxyphenyl)but-2-en-2-yl)-4,4,5,5-tetramethyl-1,3,2-dioxaborolane ((*E*)-2m)

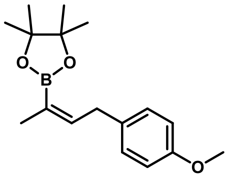

(57mg, 90% yield, *E/Z*>95:5, 0.2mmol scale, colorless oil). Purified by column chromatography (5/95 diethyl ether/petroleum ether).

^{1}H NMR (400 MHz, Chloroform-*d*) δ 7.08 – 7.03 (m, 2H), 6.77 – 6.72 (m, 3H), 6.12 – 6.05 (m, 1H), 3.70 (s, 4H), 3.55 (dd, *J* = 7.7, 1.3 Hz, 2H), 1.70 (q, *J* = 1.3 Hz, f3H), 1.22 (s, 12H). ^{13}C NMR (101 MHz, Chloroform-*d*) δ 157.9, 145.8, 133.8, 129.6, 114.0, 113.9, 83.1, 55.4, 36.8, 25.0, 24.98, 24.95, 22.4. HRMS (APCI) m/z: [M + H]$^+$ calcd for $C_{17}H_{26}BO_3$ 289.1975; Found 289.1962.

2,2'-((2E,6E)-octa-2,6-diene-2,7-diyl)bis(4,4,5,5-tetramethyl-1,3,2-dioxaborolane) ((*E,E*)-2o

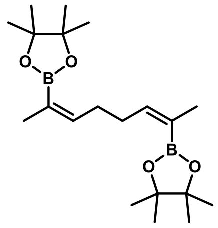

Reaction for 72 h in DCE at 80 ºC (65mg, 90% yield, *E/Z* 92:8, 0.2mmol scale, yellow viscous oil). Purified by column chromatography (5/95 diethyl ether/petroleum ether).

^{1}H NMR (400 MHz, Chloroform-*d*) δ 6.05 (s, 2H), 2.42 – 2.34 (m, 3H), 1.74 (d, *J* = 1.5 Hz, 6H), 1.26 (s, 24H). ^{13}C NMR (101 MHz, $CDCl_3$) δ 147.0, 82.7, 31.3, 24.8, 22.4. HRMS (APCI) m/z: [M + H]$^+$ calcd for $C_{20}H_{37}B_2O_4$ 363.2906; Found 363.2872.

2,2'-((2E,7E)-nona-2,7-diene-2,8-diyl)bis(4,4,5,5-tetramethyl-1,3,2-dioxaborolane) ((*E,E*)-2p

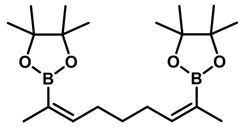

Reaction for 72 h in DCE at 80 ºC (60mg, 80% yield, *E/Z* 92:8, 0.2mmol scale, yellow viscous oil). Purified by column chromatography (5/95 diethyl ether/petroleum ether).

^{1}H NMR (400 MHz, Chloroform-*d*) δ 6.07 (t, *J* = 7.9 Hz, 2H), 2.35 – 2.26 (m, 4H), 1.75 (d, *J* = 1.4 Hz, 6H), 1.43 – 1.36 (m, 2H), 1.26 (s, 24H). ^{13}C NMR (101 MHz, $CDCl_3$) δ 147.35, 82.73, 30.62, 24.82, 22.36. HRMS (APCI) m/z: [M + H]$^+$ calcd for $C_{21}H_{39}B_2O_4$ 377.3029; Found 377.3020.

Optimization of the isomerization conditions using [Ru(HCl)COCl(PPh$_3$)$_4$]

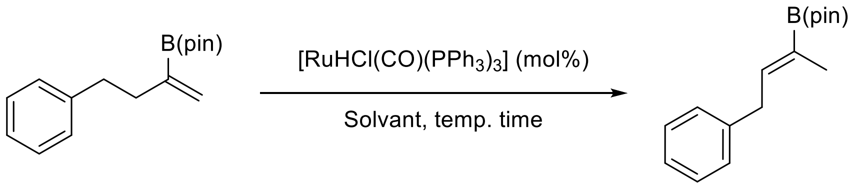

Entry	Catalyst loading	Solvent	Temp.	Time	Yield[a]	*E/Z*[b]
1	10 mol%	THF	90 °C[c]	24 h	33%	15/85
2	10 mol%	Pinacolone	90 °C[c]	24 h	14%	20/80
3	10 mol%	MeCN	90 °C[c]	24 h	3%	50/50
4	10 mol%	Benzene	90 °C[c]	24 h	76%	21/79
5	10 mol%	Toluene	110 °C	24 h	62%	27/73
6	10 mol%	Mesitylene	110 °C	24 h	73%	33/66
7	10 mol%	Toluene	50 °C	24 h	0%	-
8	10 mol%	Toluene	80°C	24 h	60%	18/82
9	10 mol%	Toluene	100 °C	24 h	78%	22/78
10	10 mol%	Toluene	100 °C	1 h	51%	15/85
11	10 mol%	Toluene	100 °C	6 h	54%	22/78
12	5 mol%	Toluene	100 °C	24 h	78%	27/73
13	1 mol%	Toluene	100 °C	24 h	20%	34/66

[a] yield of the isolated *E/Z* mixture. [b] Ratio determined by ^{1}H NMR analysis of the *E/Z* mixture. [c] reaction performed in a sealed tube flushed with argon.

A rapid solvent screening showed better conversion and selectivity in an aromatic solvent, toluene was eventually preferred for its high boiling point and availability. The optimal temperature for the transformation is displayed in entry 9, lower temperature offers a marginal increase in

selectivity at the price of substantially longer reaction time (entries 7 and 8). Performing the reaction at reflux temperature of toluene marginally increases conversion and worsens the selectivity (entry 5). Longer reaction times are necessary to achieve yields exceeding 70% (entries 10 and 11 demonstrate that most of the conversion occurs rapidly, but abruptly slows down). Lowering the catalyst loading is not recommended as conversion rate and selectivity are both reduced (entries 12 and 13).

Product isomerized with [Ru(HCl)COCl(PPh_3)$_4$]

General procedure for the (Z)-selective, [Ru] catalyzed isomerization of terminal alkenylboronate ester

Under argon atmosphere, RuHCl(CO)(PPh_3)$_3$ (19 mg, 0.02mmol, 10 mol%) was added to a flame-dried Schlenk tube equipped with a magnetic stir bar. The tube was put under vacuum and backfilled with argon (3 times). A solution of the substrate (0.2 mmol, 1.0 equiv) in toluene (1 mL) was added by syringe and stirred at 100 °C. After 24 h, the mixture was cooled to room temperature and quenched by evaporation of the solvent. The crude residue was purified by flash column chromatography on boric acid-treated silica gel (petroleum ether/diethyl ether = 20:0 to 10:1) to give the resulting (Z)-mono-isomerized product.

(*Z*)-2-(hex-2-en-2-yl)-4,4,5,5-tetramethyl-1,3,2-dioxaborolane ((*Z*)-2a)

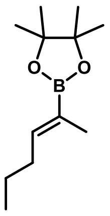

(39 mg recovered, 93% yield, 20/80 *E/Z*, 0.2 mmol scale, colorless oil). Purified by column chromatography (1/99 diethyl ether/petroleum ether).

^{1}H NMR (400 MHz, Chloroform-*d*) δ 6.32 (ddd, *J* = 8.9, 6.1, 1.9 Hz, 1H), 2.14 – 2.04 (m, 2H), 1.71 – 1.63 (m, 3H), 1.42 (h, *J* = 7.4 Hz, 2H), 1.26 (s, 12H), 0.91 (t, *J* = 7.4 Hz, 3H). ^{13}C NMR (101 MHz, $CDCl_3$) δ 146.5, 83.0, 30.8, 24.8, 22.0, 14.0, 13.9. HRMS (APCI) m/z: $[M + H]^+$ calcd for $C_{12}H_{24}BO_2$ 211.1869; Found 211.1866.

(*Z*)-4,4,5,5-tetramethyl-2-(4-methylpent-2-en-2-yl)-1,3,2-dioxaborolane ((*Z*)-2c)

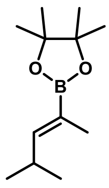

(30 mg recovered, 42% yield, 11/89 *E/Z*, 0.2 mmol scale, colorless oil). Purified by column chromatography (1/99 diethyl ether/petroleum ether).

^{1}H NMR (400 MHz, Chloroform-*d*) δ 6.12 (dd, *J* = 9.1, 1.8 Hz, 1H), 2.68 (dp, *J* = 9.1, 6.7 Hz, 1H), 1.68 (d, *J* = 1.7 Hz, 3H), 1.26 (s, 12H), 0.96 (d, *J* = 6.7 Hz, 6H). ^{13}C NMR (101 MHz, $CDCl_3$) δ 153.3, 130.07, 83.0, 44.8, 28.0, 27.4, 24.8, 22.2, 13.7. HRMS (APCI) m/z: $[M + H]^+$ calcd for $C_{12}H_{24}BO_2$ 211.1869; Found 211.1882. HRMS (APCI) m/z: $[M + H]^+$ calcd for $C_{12}H_{24}BO_2$ 211.1869; Found 211.1882. HRMS (APCI) m/z: $[M + H]^+$ calcd for $C_{12}H_{24}BO_2$ 211.1869; Found 211.1882.

(*Z*)-tert-butyldimethyl((4-(4,4,5,5-tetramethyl-1,3,2-dioxaborolan-2-yl)pent-3-en-1-yl)oxy)silane ((*Z*)-2d)

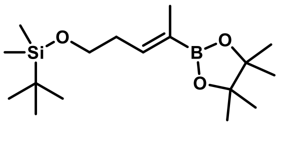

(63.7 mg recovered,86% yield, 21/79 *E/Z,* 0.2 mmol scale, colorless oil). Purified by column chromatography (1/99 diethyl ether/petroleum ether).

Larger scale isomerization: 227 mg isolated, 69% yield, 1 mmol scale (1/99 diethyl ether/petroleum ether).

^{1}H NMR (400 MHz, Chloroform-*d*) δ 6.34 – 6.24 (m, 1H), 3.65 (t, *J* = 7.3 Hz, 2H), 2.37 (qd, *J* = 7.2, 1.1 Hz, 2H), 1.69 (d, *J* = 0.9 Hz, 3H), 1.25 (s, 12H), 0.88 (d, *J* = 2.4 Hz, 9H), 0.04 (s, 6H). ^{13}C NMR (101 MHz, $CDCl_3$) δ 141.9, 83.1, 62.3,

32.5, 25.98, 25.95, 24.8, 24.7, 18.4, 14.0, -5.3. HRMS (APCI) m/z: $[M + H]^+$ calcd for $C_{17}H_{36}BO_3Si$ 327.2521; Found 327.2544.

(Z)-2-(5-chloropent-2-en-2-yl)-4,4,5,5-tetramethyl-1,3,2-dioxaborolane ((*Z*)-2f)

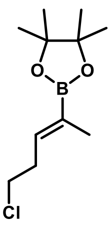

(38 mg recovered, 51% yield, 25/75 *E/Z,* 0.2 mmol scale, yellow oil). Purified by column chromatography (1/99 diethyl ether/petroleum ether).

^{1}H NMR (400 MHz, Chloroform-*d*) δ 6.27 (ddd, *J* = 8.7, 6.0, 1.8 Hz, 1H), 3.55 (t, *J* = 7.2 Hz, 2H), 2.61 (qd, *J* = 7.1, 1.0 Hz, 2H), 1.73 – 1.67 (m, 3H), 1.26 (s, 15H). ^{13}C NMR (101 MHz, $CDCl_3$) δ 140.5, 83.3, 43.3, 32.1, 24.8, 24.7, 14.1. HRMS (APCI) m/z: $[M + H]^+$ calcd for $C_{11}H_{21}BClO_2$ 231.1323; Found 231.1335.

(*Z*)-1-(4-(4,4,5,5-tetramethyl-1,3,2-dioxaborolan-2-yl)pent-3-en-1-yl)-1H-indole ((*Z*)-2g)

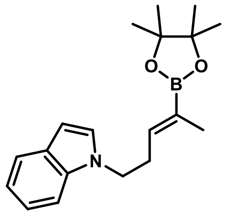

(62 mg recovered, 94% yield, 33/67 *E/Z,* 0.2mmol scale, colorless viscous oil). Purified by column chromatography (5/95 diethyl ether/petroleum ether).

^{1}H NMR (400 MHz, Chloroform-*d*) δ 7.68 – 7.60 (m, 1H), 7.37 (dd, *J* = 8.2, 1.0 Hz, 1H), 7.22 (ddd, *J* = 8.3, 7.0, 1.2 Hz, 1H), 7.17 – 7.07 (m, 2H), 6.50 (dd, *J* = 3.2, 0.9 Hz, 1H), 6.38 (ddt, *J* = 7.1, 5.3, 1.8 Hz, 1H), 4.24 – 4.17 (m, 2H), 2.75 – 2.61 (m, 2H), 1.69 – 1.62 (m, 3H), 1.29 (s, 12H). ^{13}C NMR (101 MHz, $CDCl_3$) δ 140.8, 135.8, 128.6, 127.5, 121.3, 120.9, 119.2, 109.2, 101.1, 83.3, 45.4, 29.7, 24.7, 13.9. HRMS (APCI) m/z: $[M + H]^+$ calcd for $C_{19}H_{27}BNO_2$ 312.2129; Found 312.2154.

(*Z*)-4,4,5,5-tetramethyl-2-(4-phenylbut-2-en-2-yl)-1,3,2-dioxaborolane ((*Z*)-2k

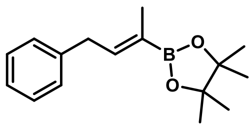

(51 mg recovered, 71% yield, 20/80 *E/Z,* 0.2 mmol scale, colorless oil). Purified by column chromatography (1/99 diethyl ether/petroleum ether).

^{1}H NMR (400 MHz, Chloroform-*d*) δ 7.29 (d, *J* = 7.6 Hz, 1H), 7.23 – 7.15 (m, 4H), 6.51 – 6.42 (m, 1H), 3.48 (d, *J* = 7.1 Hz, 2H), 1.84 – 1.73 (m, 4H), 1.25 (s, 12H). ^{13}C NMR (101 MHz, $CDCl_3$) δ 144.2, 140.5, 128.6, 128.4, 125.9, 83.2, 35.0, 24.86, 24.79, 24.76, 14.0. HRMS (APCI) m/z: $[M + H]^+$ calcd for $C_{16}H_{24}BO_2$ 259.1864; Found 259.1877.

(*Z*)-4,4,5,5-tetramethyl-2-(4-(4-(trifluoromethyl)phenyl)but-2-en-2-yl)-1,3,2-dioxaborolane ((*Z*)-2l)

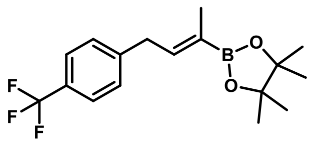

(59 mg recovered, 85% yield, 24/76 *E/Z,* 0.2 mmol scale, yellow oil). Purified by column chromatography (5/95 diethyl ether/petroleum ether).

^{1}H NMR (400 MHz, Chloroform-*d*) δ 7.53 (d, *J* = 8.0 Hz, 2H), 7.30 (d, *J* = 8.0 Hz, 2H), 6.42 (td, *J* = 7.1, 1.8 Hz, 1H), 3.53 (d, *J* = 7.1 Hz, 2H), 1.87 – 1.74 (m, 3H), 1.26 (s, 12H). ^{13}C NMR (101 MHz, $CDCl_3$) δ 142.7, 128.9, 125.3, 125.3, 83.3, 34.8, 24.9, 24.8, 14.1. HRMS (APCI) m/z: $[M + H]^+$ calcd for $C_{17}H_{23}BF_3O_2$ 327.1738; Found 327.1744.

(*Z*)-2-(4-(4-methoxyphenyl)but-2-en-2-yl)-4,4,5,5-tetramethyl-1,3,2-dioxaborolane ((*Z*)-2m)

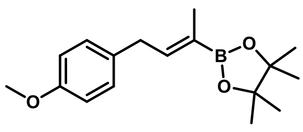

(44.5 mg recovered, 64% yield, 23/77 *E/Z,* 0.2 mmol scale, yellow oil). Purified by column chromatography (5/95 diethyl ether/petroleum ether).

^{1}H NMR (400 MHz, Chloroform-*d*) δ 7.10 (d, *J* = 8.6 Hz, 2H), 6.86 – 6.79 (m, 2H), 6.45 (td, *J* = 7.1, 1.8 Hz, 1H), 3.78 (s, 3H), 3.41 (d, *J* = 7.1 Hz, 2H), 1.83 – 1.75 (m, 3H), 1.25 (s, 12H). ^{13}C NMR (101 MHz, $CDCl_3$) δ 157.8, 144.7, 132.6, 129.5, 113.8, 83.2, 55.3, 34.1, 24.8, 14.0. HRMS (APCI) m/z: $[M + H]^+$ calcd for $C_{17}H_{26}BO_3$ 289.1975; Found 289.1986.

(*Z*)-2-(hex-2-en-2-yl)-5,5-dimethyl-1,3,2-dioxaborinane ((*Z*)-3a)

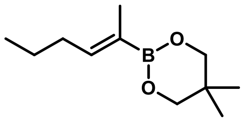

(28.3mg, 72% yield, 10/90 *E/Z*, 0.2 mmol scale, yellow oil). Purified by column chromatography (1/99 diethyl ether/petroleum ether).

^{1}H NMR (400 MHz, Chloroform-*d*) δ 6.24 – 6.15 (m, 1H), 3.57 (s, 4H), 2.02 (d, *J* = 7.3 Hz, 2H), 1.56 (d, *J* = 1.8 Hz, 3H), 1.35 (q, *J* = 7.4 Hz, 2H), 0.89 (d, *J* = 1.2 Hz, 6H), 0.84 (q, *J* = 3.3 Hz, 3H). ^{13}C NMR (101 MHz, $CDCl_3$) δ 143.7, 72.1, 31.6, 30.7, 22.2, 21.9, 21.9, 14.0, 13.4. HRMS (APCI) m/z: [M + H]$^+$ calcd for $C_{11}H_{22}BO_2$ 197.1713; Found 197.1703.

(*Z*)-tert-butyl((4-(5,5-dimethyl-1,3,2-dioxaborinan-2-yl)pent-3-en-1-yl)oxy)dimethylsilane ((*Z*)-3d)

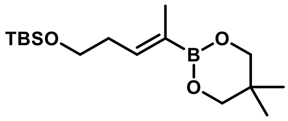

(41.8 mg, 67% yield, 11/89 *E/Z,* 0.2 mmol scale, yellow oil). Purified by column chromatography (1/99 diethyl ether/petroleum ether).

^{1}H NMR (400 MHz, Chloroform-*d*) δ 6.26 – 6.18 (m, 1H), 3.63 (s, 4H), 2.37 (q, *J* = 7.3 Hz, 2H), 1.65 (s, 3H), 0.97 – 0.93 (m, 8H), 0.89 (s, 10H), 0.05 (s, 6H). ^{13}C NMR (101 MHz, $CDCl_3$) δ 139.0, 72.1, 72.0, 71.9, 62.5, 32.5, 31.6, 26.0, 25.7, 21.8, 18.4, 13.6, -5.2. HRMS (APCI) m/z: [M + H]$^+$ calcd for $C_{16}H_{34}BO_3Si$ 313.2370; Found 313.2375.

(*Z*)-2-(5-chloropent-2-en-2-yl)-5,5-dimethyl-1,3,2-dioxaborinane ((*Z*)-3f)

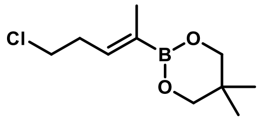

(32.9mg, 76% yield, 17/83 *E/Z* 0.2 mmol scale, yellow oil). Purified by column chromatography (1/99 diethyl ether/petroleum ether).
Exact Mass:

^{1}H NMR (400 MHz, Chloroform-*d*) δ 6.27 – 6.14 (m, 1H), 3.64 (s, 4H), 3.55 (t, *J* = 7.1 Hz, 2H), 2.60 (q, *J* = 7.1 Hz, 2H), 1.66 (s, 3H), 0.96 (s, 6H). ^{13}C NMR (101 MHz, $CDCl_3$) δ 137.9, 133.8, 128.4, 72.1, 43.6, 32.0, 25.4, 21.8, 13.7. HRMS (APCI) m/z: [M + H]$^+$ calcd for $C_{10}H_{19}BClO_2$ 217.1167; Found 217.1155.

(*Z*)-1-(4-(5,5-dimethyl-1,3,2-dioxaborinan-2-yl)pent-3-en-1-yl)-1H-indole ((*Z*)-3g)

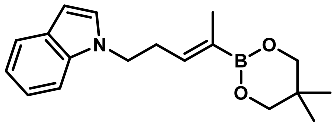

(36.3 mg, 61% yield, 21/79 *E/Z,* 0.2 mmol scale, colorless viscous oil). Purified by column chromatography (5/95 diethyl ether/petroleum ether).

^{1}H NMR (400 MHz, Chloroform-*d*) δ 7.70 – 7.58 (m, 1H), 7.37 (d, *J* = 8.1 Hz, 1H), 7.20 (dt, *J* = 7.0, 1.3 Hz, 1H), 7.14 – 7.05 (m, 2H), 6.49 (d, *J* = 3.1 Hz, 1H), 6.31 (td, *J* = 6.4, 6.0, 3.2 Hz, 1H), 4.18 (dd, *J* = 8.4, 6.9 Hz, 2H), 3.65 (s, 4H), 2.67 (q, *J* = 7.4 Hz, 2H), 1.59 (d, *J* = 1.7 Hz, 3H), 0.97 (d, *J* = 1.1 Hz, 6H). ^{13}C NMR (101 MHz, $CDCl_3$) δ 138.11, 127.60, 121.31, 120.90, 119.18, 109.27, 100.99, 72.14, 45.60, 31.63, 29.58, 21.82, 13.49. HRMS (APCI) m/z: [M + H]$^+$ calcd for $C_{18}H_{25}BNO_2$ 298.1978; Found 298.1966.

(*Z*)-5,5-dimethyl-2-(4-phenylbut-2-en-2-yl)-1,3,2-dioxaborinane ((*Z*)-3k)

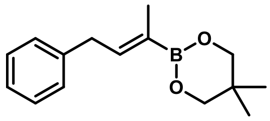

(35.1 mg, 72% yield, 9/91 *E/Z* , 0.2 mmol scale, colorless oil). Purified by column chromatography (1/99 diethyl ether/petroleum ether).

^{1}H NMR (400 MHz, Chloroform-*d*) δ 7.33 – 7.26 (m, 3H), 7.24 – 7.16 (m, 4H), 6.45 (tt, *J* = 7.4, 1.8 Hz, 1H), 3.64 (s, 4H), 3.50 (d, *J* = 7.1 Hz, 2H), 1.80 – 1.75 (m, 3H), 0.97 (s, 6H). ^{13}C NMR (101 MHz, $CDCl_3$) δ 141.6, 128.6, 128.4, 125.7, 72.1, 35.0, 31.6, 21.9, 21.8, 13.6. HRMS (APCI) m/z: [M + H]$^+$ calcd for $C_{15}H_{22}BO_2$ 245.1713; Found 245.1712.

Cross-coupling reactions

The cross-coupling of the (*E*)-**2a** with *o*-bromotoluene and *p*-nitro iodobenzene was performed using a reported procedure.[12]

(*Z*)-1-(hex-2-en-2-yl)-4-methoxybenzene (4a)

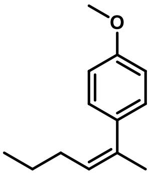

(35 mg,92% yield, 5/95 *E/Z*, 0.2 mmol scale, colorless viscous oil). Prepared according to a literature report, NMR analysis being fully consistent with the report.[13] Purified by column chromatography (1/99 diethyl ether/petroleum ether).

(*Z*)-1-(hex-2-en-2-yl)-4-nitrobenzene (4b)

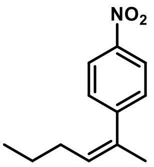

(38.2 mg, 93% yield, 5/95 *E/Z*, 0.2 mmol scale, yellow viscous oil). Purified by column chromatography (5/95 diethyl ether/petroleum ether).

^{1}H NMR (400 MHz, Chloroform-*d*) δ 8.25 – 8.15 (m, 2H), 7.33 (d, *J* = 8.8 Hz, 2H), 5.59 (tq, *J* = 7.5, 1.5 Hz, 1H), 2.05 (d, *J* = 1.4 Hz, 3H), 1.92 (qd, *J* = 7.4, 1.3 Hz, 2H), 1.36 (h, *J* = 7.4 Hz, 2H), 0.84 (t, *J* = 7.4 Hz, 3H). ^{13}C NMR (101 MHz, $CDCl_3$) δ 149.4, 138.6, 134.2, 130.2, 128.9, 124.8, 123.4, 31.2, 25.0, 23.0, 13.7. HRMS (APCI) m/z: $[M + H]^+$ calcd for $C_{12}H_{16}NO_2$ 206.1176; Found 206.1159.

Procedure for the one-pot isomerization and cross-coupling

Sodium tetrakis[3,5-bis(trifluoromethyl)phenyl]borate (10 mg, 0.011 mmol, 5.6 mol%) is placed in a flame-dried Schlenk tube under argon flow. PCy_3 (8.41 mg, 0.03 mmol, 15 mol%) and $[Ir(cod)Cl]_2$ (3.36 mg, 0.005 mmol, 2.5 mol%) are subsequently added under a flow of argon and the solids stirred for 1 minute at room temperature before DCM (1 mL) is added. Upon complete dissolution of the solids (resulting in an orange-reddish solution), hydrogen gas is bubbled through a needle for 1 minute, adding DCM at the end of this hydrogenation step can be necessary to conserve the desired volume of solvent. Subsequently, the reaction is degassed by performing 3 freeze, pump, thaw cycles. The substrate (0.2 mmol, 1 equiv.) is added neat and the resulting mixture is allowed to stir at 40 ºC. Upon consumption of the starting material (usually 1 hour, conversion was monitored by TLC: petroleum ether/diethyl ether eluent system (20/1), visualized with anisaldehyde, the isomerized boronic ester are typically 0.05 units higher in Rf than the starting material). To the reaction mixture, $Pd(OAc)_2$ (5 mol%), Sphos (10 mol%) and K_3PO_4 (3.0 equiv) were added, followed by 1.5 equiv. of the aryl bromide or iodide of choice together with 5 equiv. of water. The reaction mixture is stirred at 80 ºC until completion (petroleum ether/diethyl ether eluent system (20/1)). The reaction is then hydrolyzed by the addition of water, extracted twice with diethyl ether, the organic phase was dried over sodium sulfate and filtered. After concentrating the crude reaction mixture, the purification was done by column chromatography with a solvent fitting the polarity of the obtained styrenyl compound.

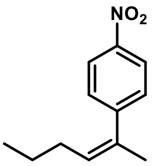

(11 mg, 40% yield, 5/95 *E/Z*, 0.2 mmol scale, yellow viscous oil)

^{1}H NMR (400 MHz, Chloroform-*d*) δ 8.25 – 8.15 (m, 2H), 7.33 (d, *J* = 8.8 Hz, 2H), 5.59 (tq, *J* = 7.5, 1.5 Hz, 1H), 2.05 (d, *J* = 1.4 Hz, 3H), 1.92 (qd, *J* = 7.4, 1.3 Hz, 2H), 1.36 (h, *J* = 7.4 Hz, 2H), 0.84 (t, *J* = 7.4 Hz, 3H). ^{13}C NMR (101 MHz, $CDCl_3$) δ 149.37, 138.65, 134.23, 130.24, 128.88, 124.84, 123.41, 31.19, 25.03, 23.03, 13.73. HRMS (APCI) m/z: $[M + H]^+$ calcd for $C_{12}H_{16}NO_2$ 206.1176; Found 206.1159.

Determination of the stereochemistry

The determination of the *E/Z* ratio was performed by analysis of the crude reaction mixture by NMR integration. The *E* and *Z* isomers were identified by comparison with a report in the literature when existent,[14–21] by analogy with other NMR shifts and the change in the multiplicity of the Csp^2 bound H (the *E*-isomers generally present a signal as triplet while the *Z* presents a characteristic doublet of triplet). Alternatively, NOESY could be used (special correlation spots are in green/blue color). With this technique, the difference between the two isomers appeared clearly.

List of literature precedent

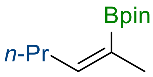

(*E*)-**2a**
ref. 13
(*Z*)-**2a**
ref. 14

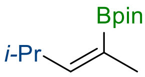

(*E*)-**2c**
ref. 16
(*Z*)-**2c**
ref. 15

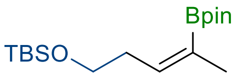

(*Z*)-**2d**
ref. 20

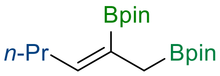

(*E*)-**2h**
ref. 17

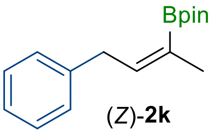

(*Z*)-**2k**
ref. 18

Illustrative example for the NOESY determination of E/Z ratio

This example is the mixture of the two isomers obtained by reaction of **1a** with the [Ru] catalyst. The major (*Z*)-isomer presents a correlation (represented and circled in blue) with the *n*-propyl chain CH_2 protons but not with the methyl ones. Whereas the minor *E*-isomer is characterized by a strong correlation with the methyl hydrogens (in red).

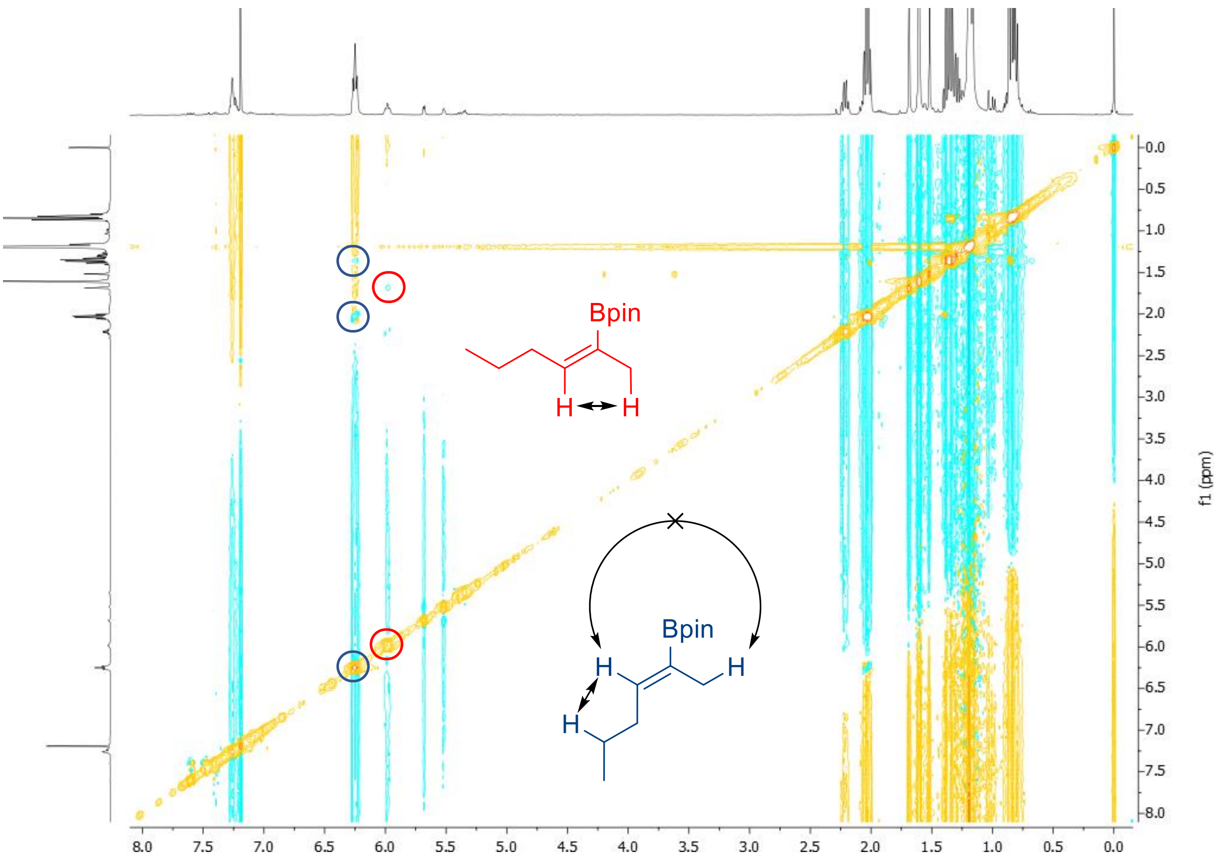

Typical different signals in ¹H NMR

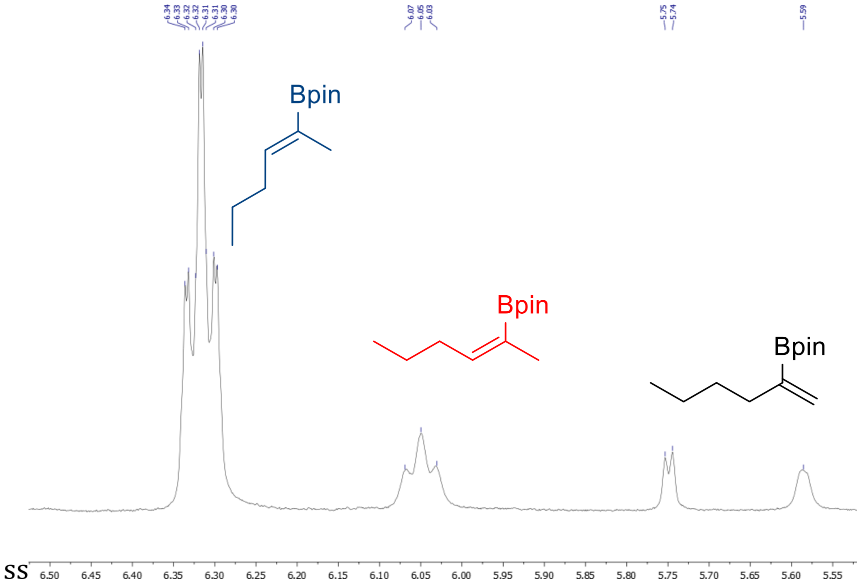

References

(1) Hitosugi, S.; Tanimoto, D.; Nakanishi, W.; Isobe, H. A Facile Chromatographic Method for Purification of Pinacol Boronic Esters. *Chem. Lett.* **2012**, *41*, 972–973.

(2) Williams, D. B. G.; Lawton, M. Drying of Organic Solvents: Quantitative Evaluation of the Efficiency of Several Desiccants. *J. Org. Chem.* **2010**, *75*, 8351–8354.

(3) Gao, F.; Hoveyda, A. H. α-Selective Ni-Catalyzed Hydroalumination of Aryl-and Alkyl-Substituted Terminal Alkynes: Practical Syntheses of Internal Vinyl Aluminums, Halides, or Boronates. *J. Am. Chem. Soc.* **2010**, *132*, 10961–10963.

(4) Marshall, J. A.; DeHoff, B. S. The Synthesis of Cembranolide Precursors via Addition of Allylstannanes to Conjugated Aldehydes. *J. Org. Chem.* **1986**, *51*, 863–872.

(5) Li, Z.; Zhang, L.; Nishiura, M.; Luo, G.; Luo, Y.; Hou, Z. Enantioselective Cyanoborylation of Allenes by N-Heterocyclic Carbene-Copper Catalysts. *ACS Catal.* **2020**, *10*, 11685–11692.

(6) Wang, Z.; Chen, L.; Yao, Y.; Liu, Z.; Gao, J.-M.; She, X.; Zheng, H. Dearomatization of Indole via Intramolecular [3+ 2] Cycloaddition: Access to the Pentacyclic Skeleton of Strychons Alkaloids. *Org. Lett.* **2018**, *20*, 4439–4443.

(7) Anastasia, L.; Dumond, Y. R.; Negishi, E. Stereoselective Synthesis of Exocyclic Alkenes by Cu-Catalyzed Allylmagnesiation, Pd-Catalyzed Alkylation, and Ru-Catalyzed Ring-Closing Metathesis: Highly Stereoselective Synthesis of (*Z*)-and (*E*)-γ-Bisabolenes. *European J. Org. Chem.* **2001**, *2001*, 3039–3043.

(8) Hooz, J.; Cabezas, J.; Musmanni, S.; Calzada, J. Propargylation of Alkyl Halides:(*E*)-6, 10-dimethyl-5, 9-undecadien-1-yne and (*E*)-7, 11-dimethyl-6, 10-dodecadien-2-yn-1-ol: 5, 9-Undecadien-1-yne, 6, 10-dimethyl-,(*E*)-and 6, 10-Dodecadien-2-yn-1-ol, 7, 11-dimethyl-,(*E*)-. *Org. Synth.* **2003**, *69*, 120.

(9) Nguyen, J.; Duncan, N.; Lalic, G. Direct Β-Selective Cross-Coupling of Alkenyl Gold Complexes with Alkyl Electrophiles. *European J. Org. Chem.* **2016**, *2016*, 5803–5806.

(10) Satoh, T.; Hayashi, Y.; Yamakawa, K. Fritsch–Wiechell Rearrangement of 1-Chlorovinyl Sulfoxides: A New Method for Synthesizing Acetylenes from Aldehydes with One-Carbon Homologation. *Bull. Chem. Soc. Jpn.* **1993**, *66*, 1866–1869.

(11) Ramírez, J.; Fernandez, E. Convenient Synthesis of α, α-Difluorinated Carbonyl Compounds from Alkynes through a Fluoro-Deboronation Process. *Synthesis.* **2005**, *2005*, 1698–1700.

(12) Ho, G.-M.; Segura, L.; Marek, I. Ru-Catalyzed Isomerization of ω-Alkenylboronates towards Stereoselective Synthesis of Vinylboronates with Subsequent: In Situ Functionalization. *Chem. Sci.* **2020**, *11*.

(13) James, B. G.; Pattenden, G. Dichotomous Ylide Formation from an Alkyl (Benzyl) Phosphonium Salt Leading to Stereoselective Alkene Syntheses. *J. Chem. Soc. Perkin Trans. 1* **1976**, *13*, 1476–1479.

(14) Godeau, J.; Pintaric, C.; Olivero, S.; Duñach, E. Electrochemical Preparation of Pinacol Allylboronic Esters. *Electrochim. Acta* **2009**, *54*, 5116–5119.

(15) Kim, H. R.; Yun, J. Highly Regio-and Stereoselective Synthesis of Alkenylboronic Esters by Copper-Catalyzed Boron Additions to Disubstituted Alkynes. *Chem. Commun.* **2011**, *47*, 2943–2945.

(16) Bismuto, A.; Thomas, S. P.; Cowley, M. J. Aluminum Hydride Catalyzed Hydroboration of Alkynes. *Angew. Chem. Int. Ed.* **2016**, *55*, 15356–15359.

(17) Longobardi, L. E.; Fürstner, A. Trans-Hydroboration of Propargyl Alcohol Derivatives and Related Substrates. *Chem. Eur. J.* **2019**, *25*, 10063–10068.

(18) Peck, C. L.; Nekvinda, J.; Santos, W. L. Diboration of 3-Substituted Propargylic Alcohols

Using a Bimetallic Catalyst System: Access to (*Z*)-Allyl, Vinyldiboronates. *Chem. Commun.* **2020**, *56*, 10313–10316.

(19) Guan, W.; Michael, A. K.; McIntosh, M. L.; Koren-Selfridge, L.; Scott, J. P.; Clark, T. B. Stereoselective Formation of Trisubstituted Vinyl Boronate Esters by the Acid-Mediated Elimination of α-Hydroxyboronate Esters. *J. Org. Chem.* **2014**, *79*, 7199–7204.

(20) Xu, S.; Geng, P.; Li, Y.; Liu, G.; Zhang, L.; Guo, Y.; Huang, Z. Pincer Iron Hydride Complexes for Alkene Isomerization: Catalytic Approach to Trisubstituted (*Z*)-Alkenyl Boronates. *ACS Catal.* **2021**, *11*, 10138–10147.

(21) Hesse, M. J.; Butts, C. P.; Willis, C. L.; Aggarwal, V. K. Diastereodivergent Synthesis of Trisubstituted Alkenes through Protodeboronation of Allylic Boronic Esters: Application to the Synthesis of the Californian Red Scale Beetle Pheromone. *Angew. Chem. Int. Ed.* **2012**, *51*, 12444–12448.

NMR spectra

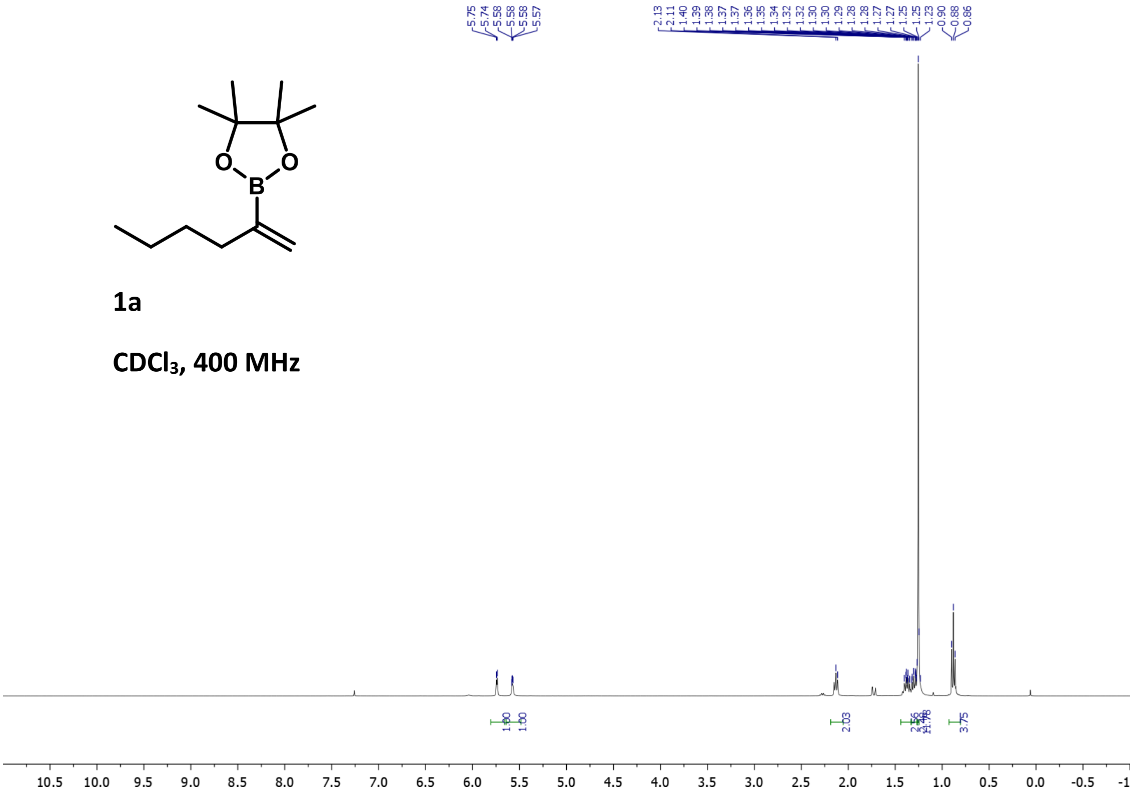

1a

$CDCl_3$, 400 MHz

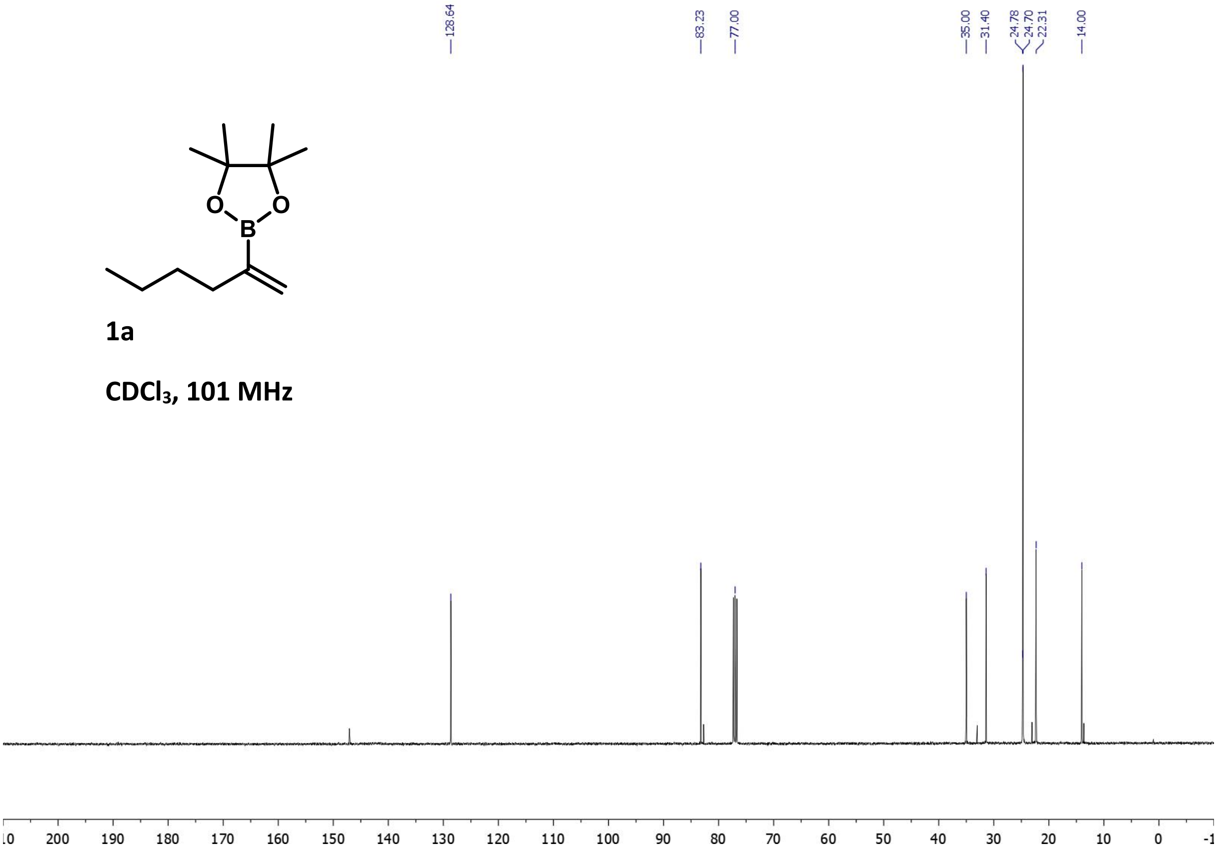
1a
CDCl3, 101 MHz
128.64
83.23
77.00
35.00
31.40
24.78
24.70
22.31
14.00

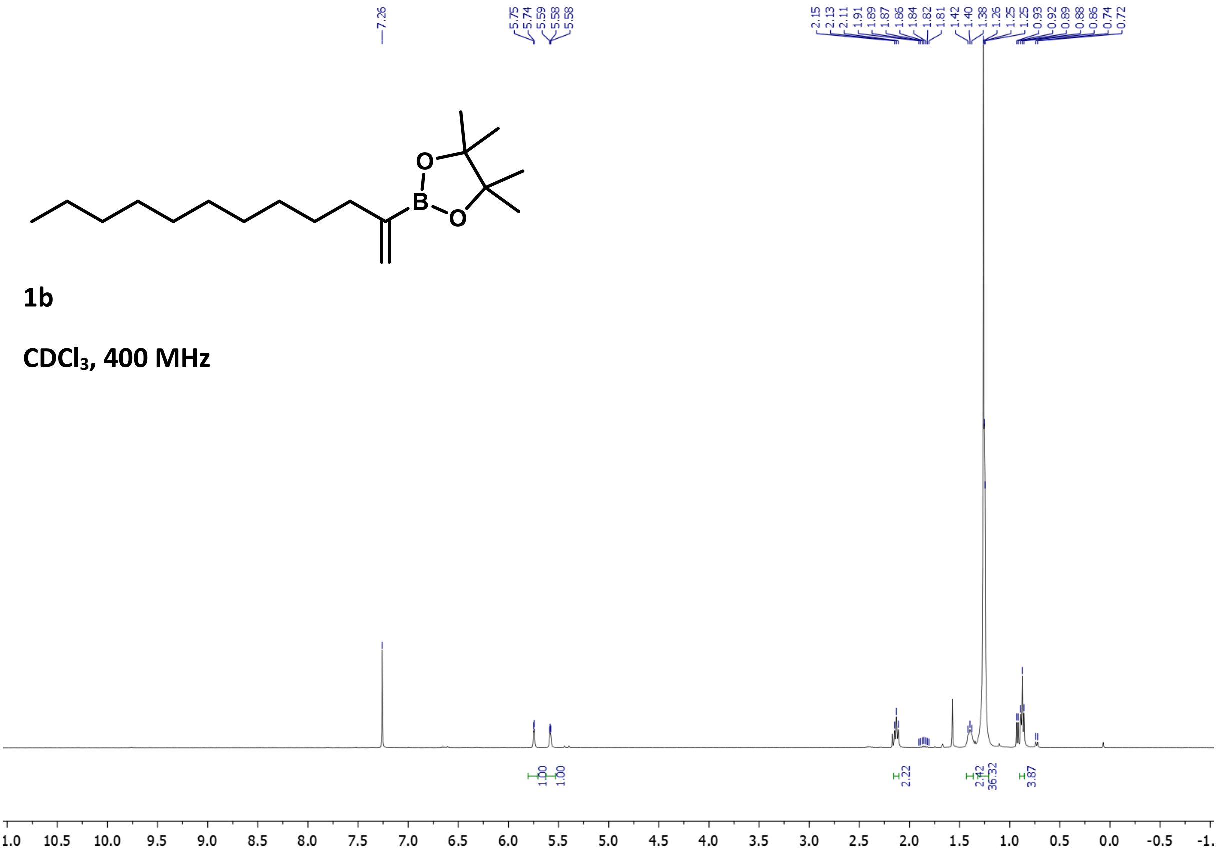
1b
CDCl3, 400 MHz
7.26
5.75
5.74
5.59
5.58
5.58
2.15
2.13
2.11
1.91
1.89
1.87
1.86
1.84
1.82
1.81
1.42
1.40
1.38
1.26
1.25
1.25
0.93
0.92
0.89
0.88
0.86
0.74
0.72
1.00
1.00
2.22
2.12
36.32
3.87

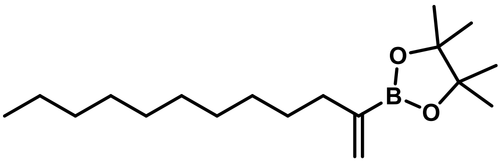

1b

$CDCl_3$, 101 MHz

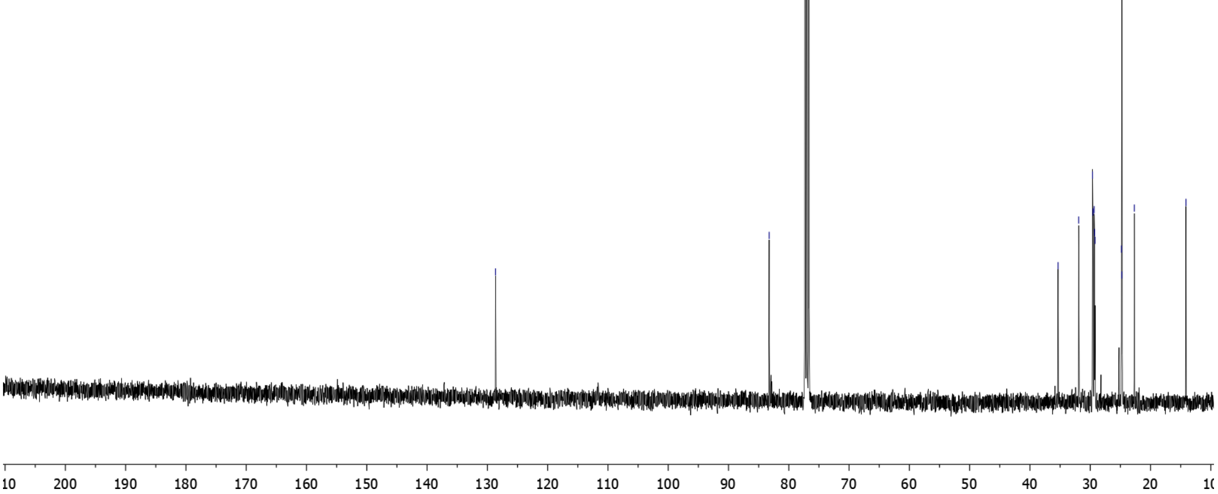

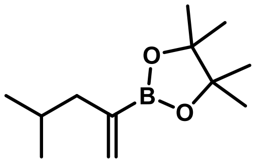

1c

$CDCl_3$, 400 MHz

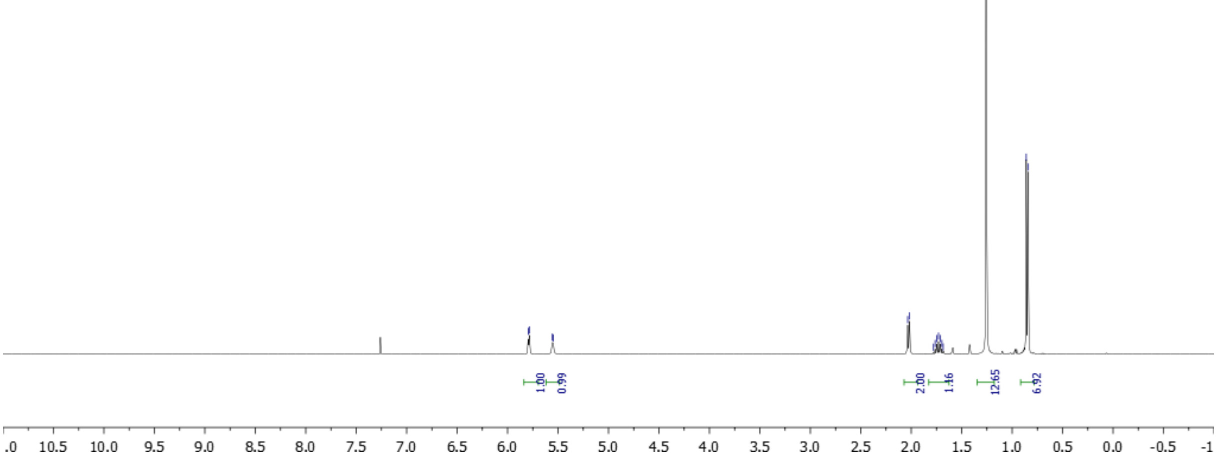

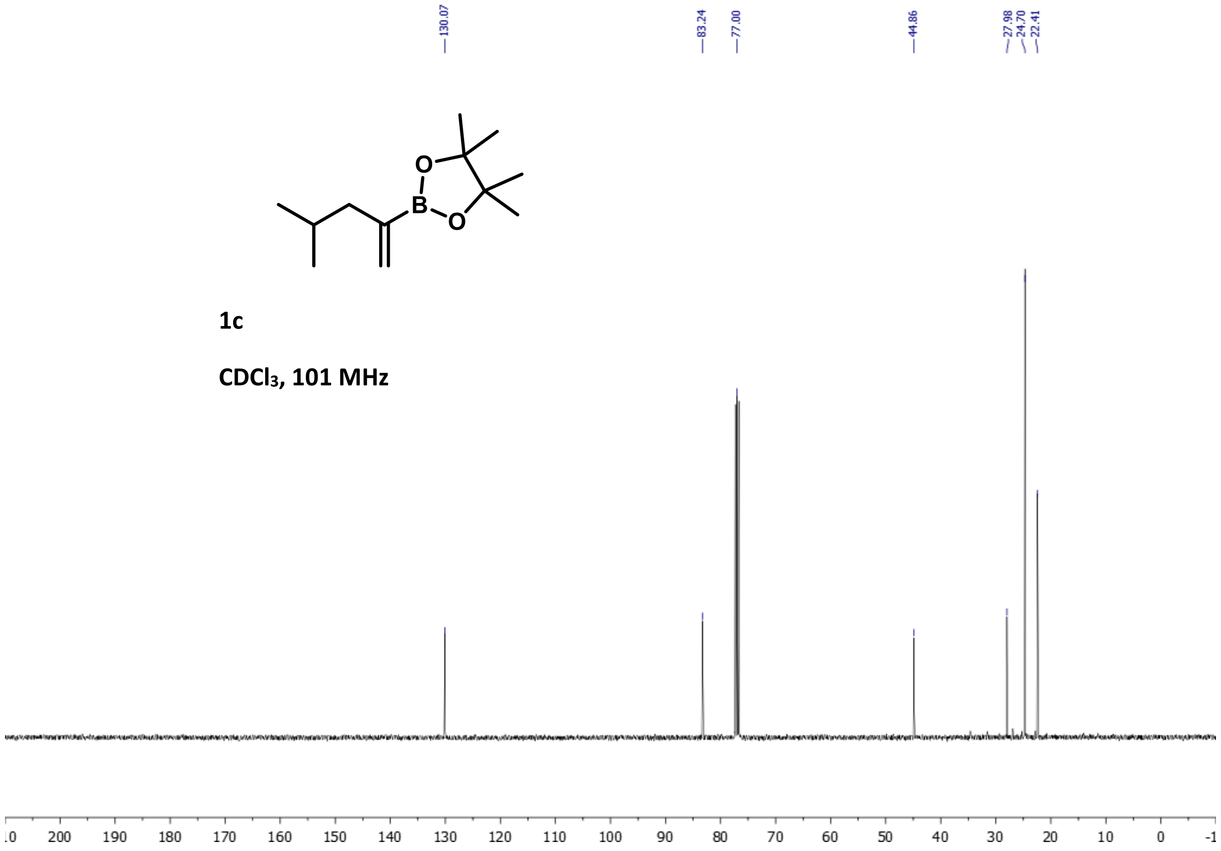
130.07
83.24
77.00
44.86
27.98
24.70
22.41
1c
CDCl₃, 101 MHz

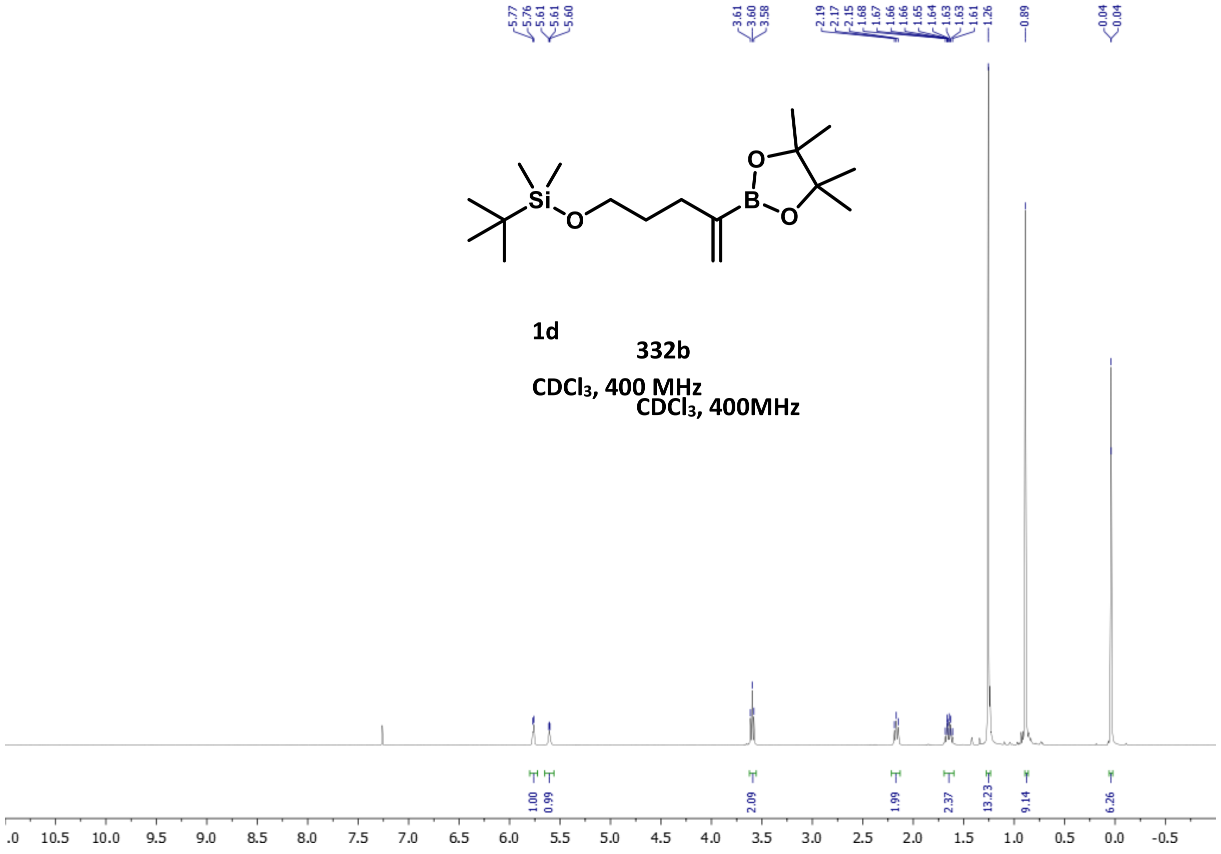
5.77
5.76
5.61
5.61
5.60
3.61
3.60
3.58
2.19
2.17
2.15
1.68
1.67
1.66
1.66
1.65
1.64
1.63
1.63
1.61
1.26
0.89
0.04
0.04
1d
332b
CDCl₃, 400 MHz
CDCl₃, 400MHz
1.00
0.99
2.09
1.99
2.37
13.23
9.14
6.26

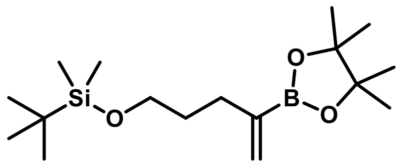

1d

$CDCl_3$, 101 MHz

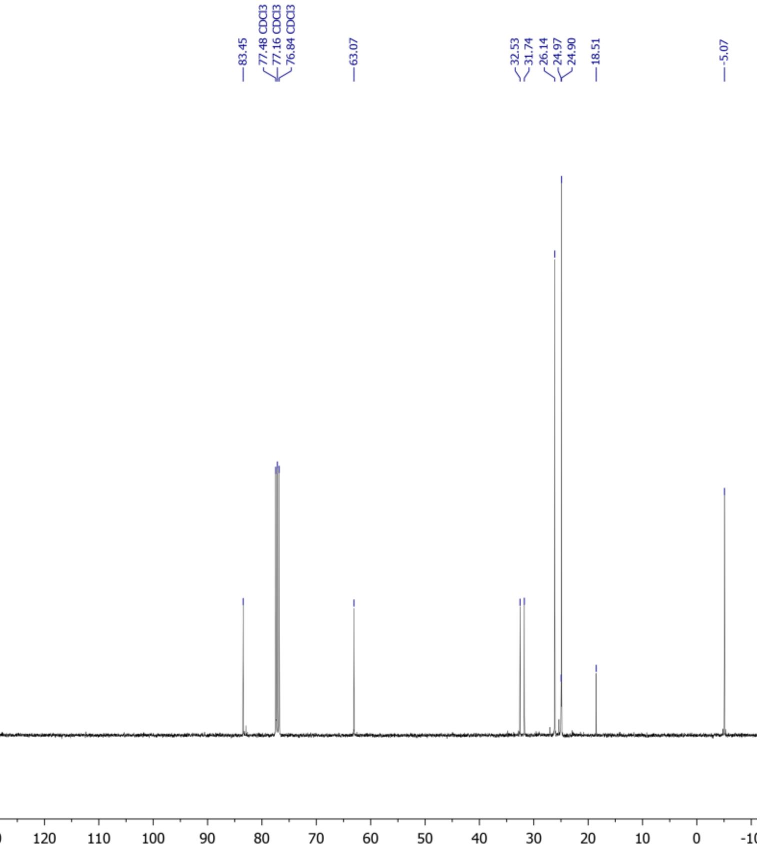

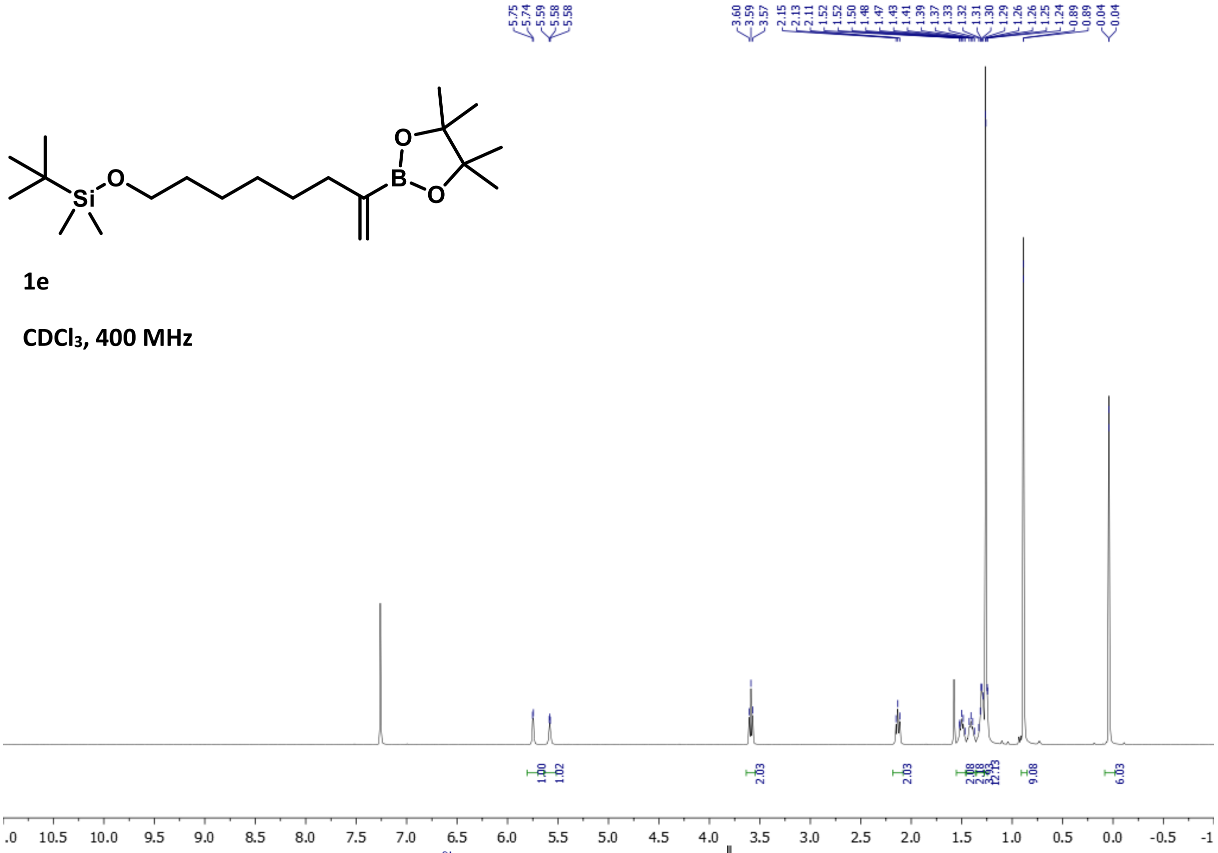

5.75
5.74
5.59
5.58
5.58
3.60
3.59
3.57
2.15
2.13
2.11
1.52
1.52
1.50
1.48
1.47
1.43
1.41
1.39
1.37
1.33
1.32
1.31
1.30
1.29
1.26
1.26
1.25
1.24
0.89
0.89
0.04
0.04
1e
CDCl$_3$, 400 MHz
1.00
1.02
2.03
2.03
2.08
2.18
3.43
12.13
9.08
6.03

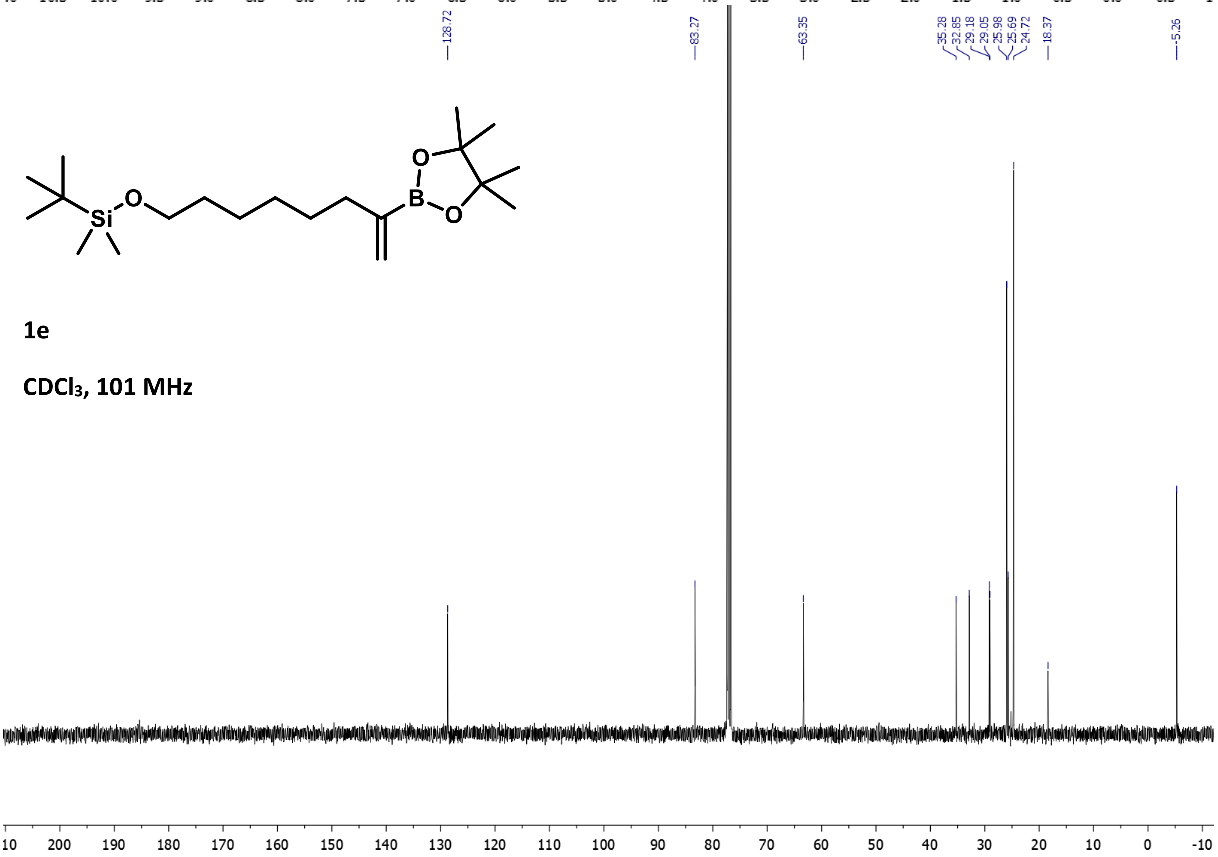

128.72
83.27
63.35
35.28
32.85
29.18
29.05
25.98
25.69
24.72
18.37
-5.26
1e
CDCl$_3$, 101 MHz

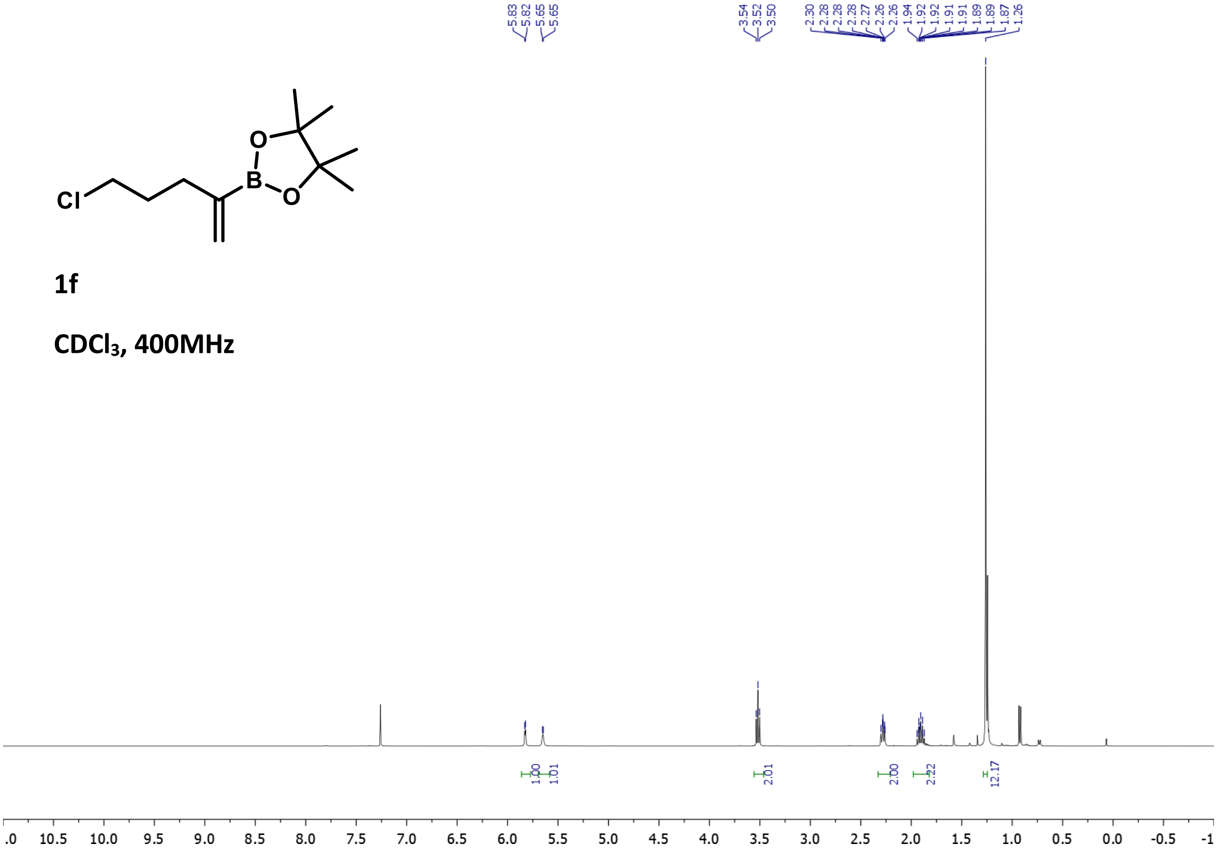
5.83
5.82
5.65
5.65
3.54
3.52
3.50
2.30
2.28
2.28
2.28
2.27
2.26
2.26
1.94
1.92
1.92
1.91
1.91
1.89
1.89
1.87
1.26
Cl
B
O
O
1f
CDCl3, 400MHz
1.00
1.01
2.01
2.00
2.22
12.17

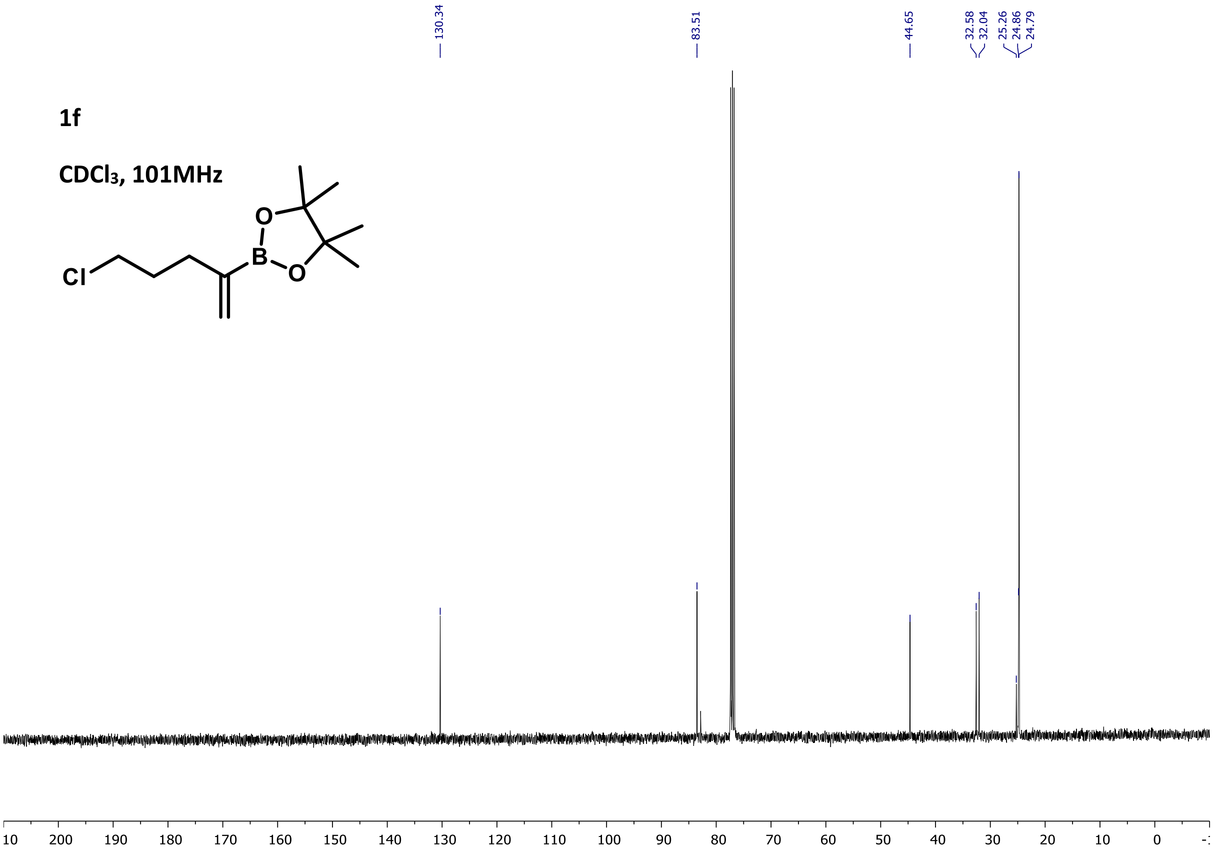
130.34
83.51
44.65
32.58
32.04
25.26
24.86
24.79
1f
CDCl3, 101MHz
Cl
B
O
O

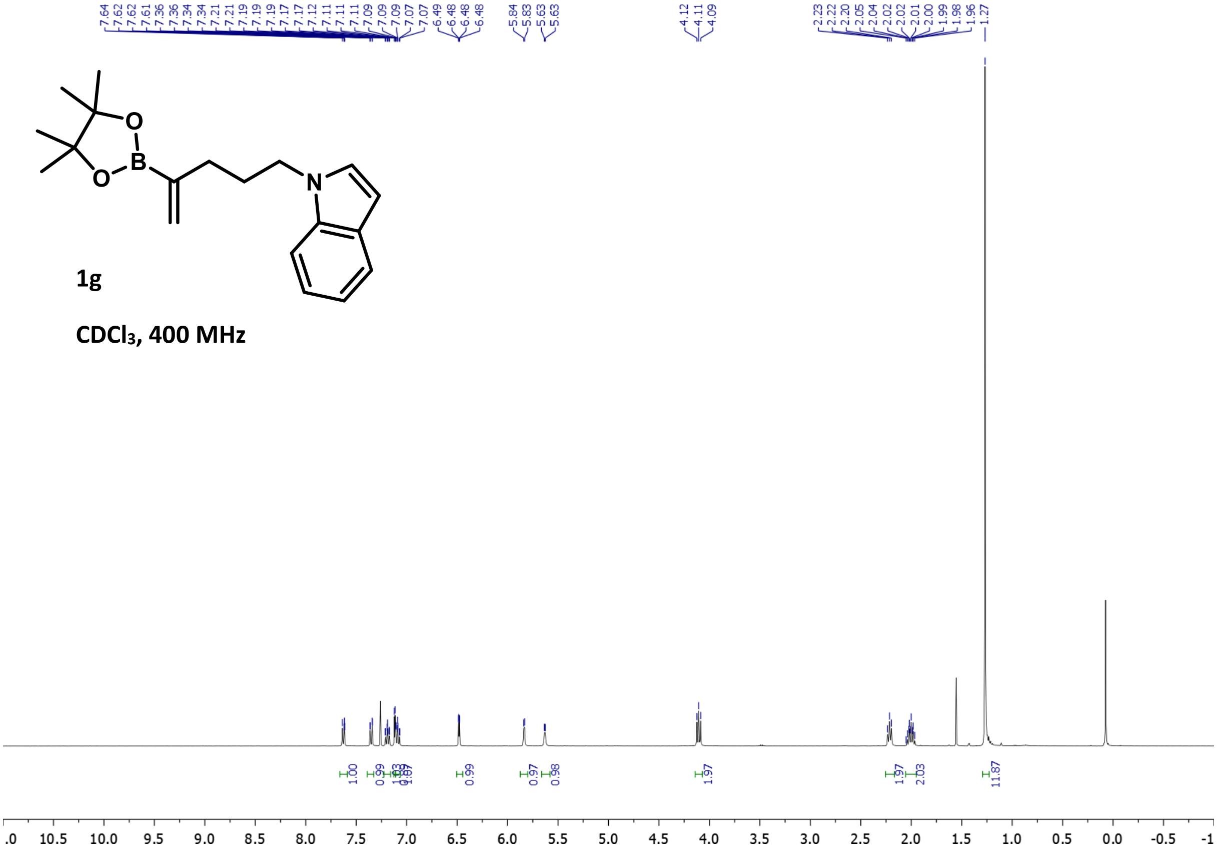

1g

CDCl₃, 400 MHz

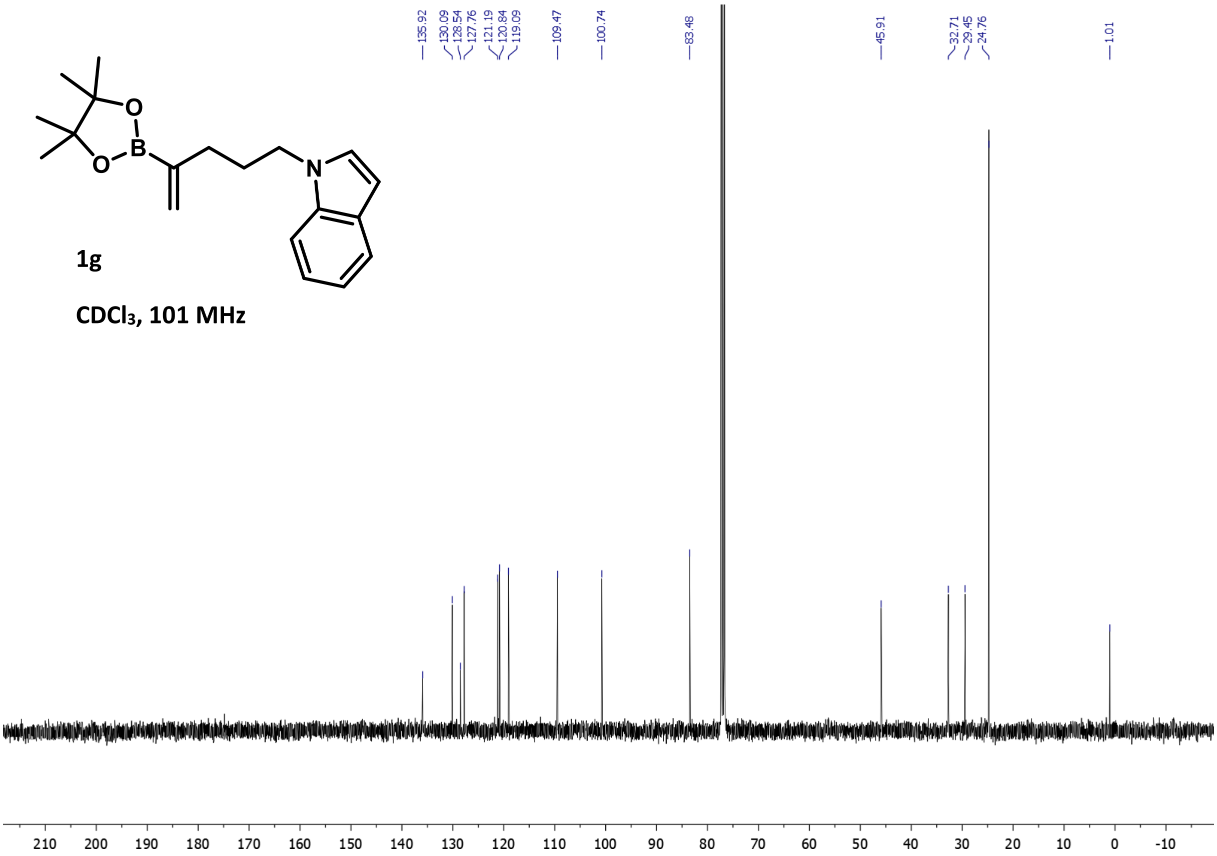

1g

CDCl₃, 101 MHz

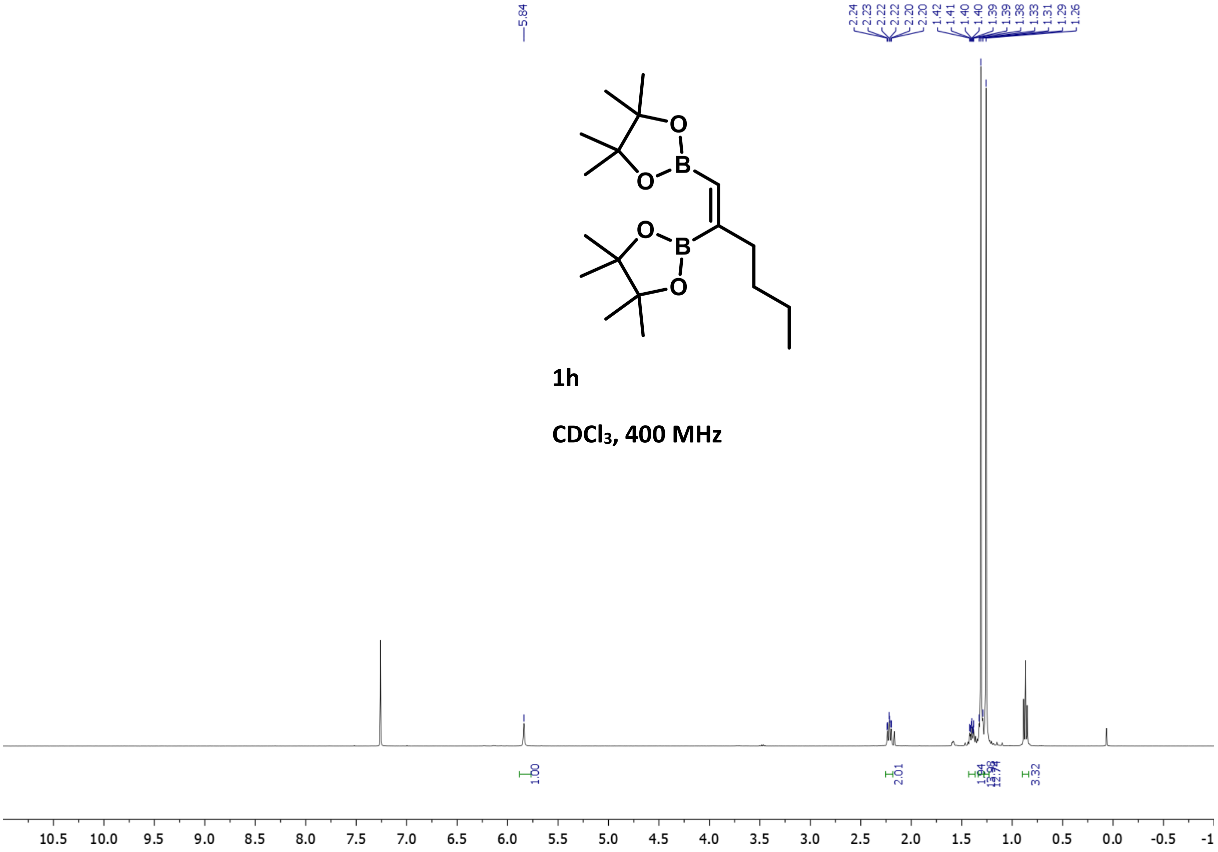
1h
CDCl3, 400 MHz
5.84
2.24
2.23
2.22
2.22
2.20
2.20
1.42
1.41
1.40
1.40
1.39
1.39
1.38
1.33
1.31
1.29
1.26
1.00
2.01
3.32

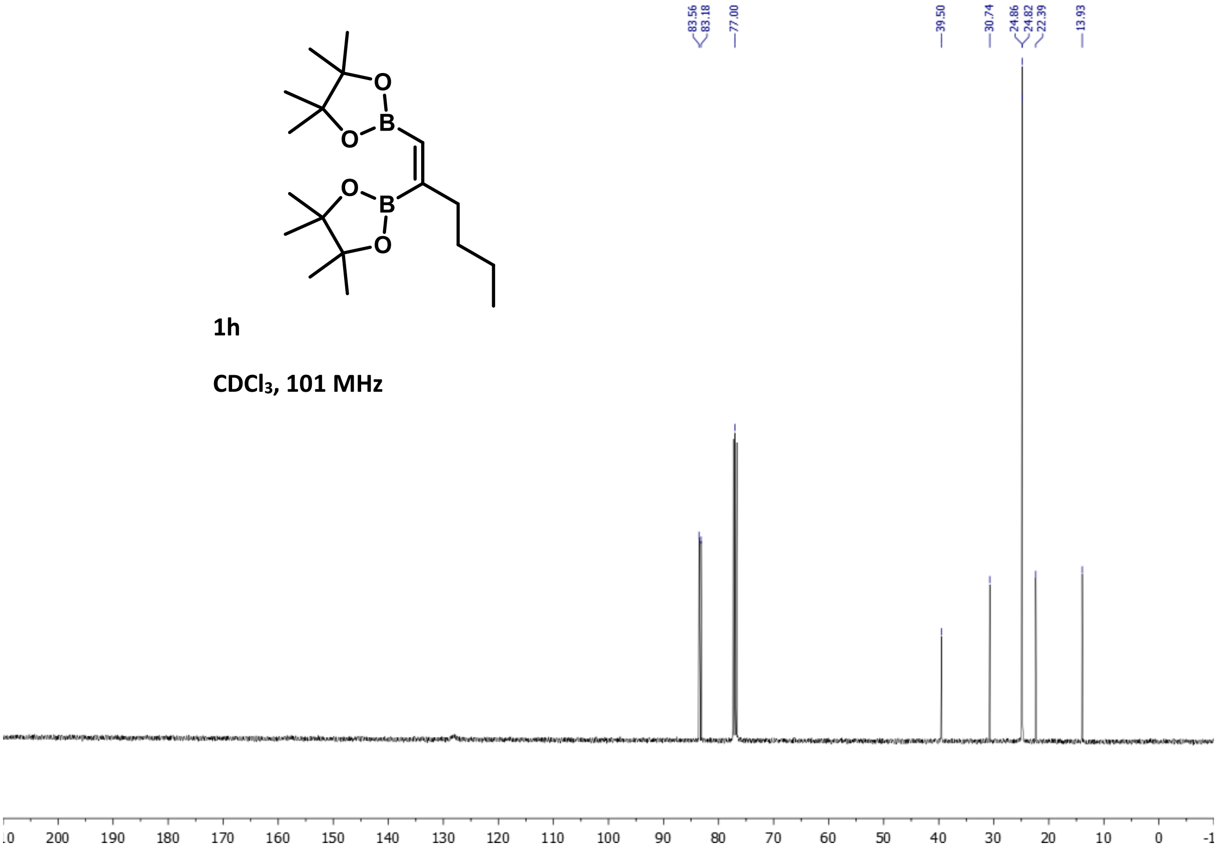
1h
CDCl3, 101 MHz
83.56
83.18
77.00
39.50
30.74
24.86
24.82
22.39
13.93

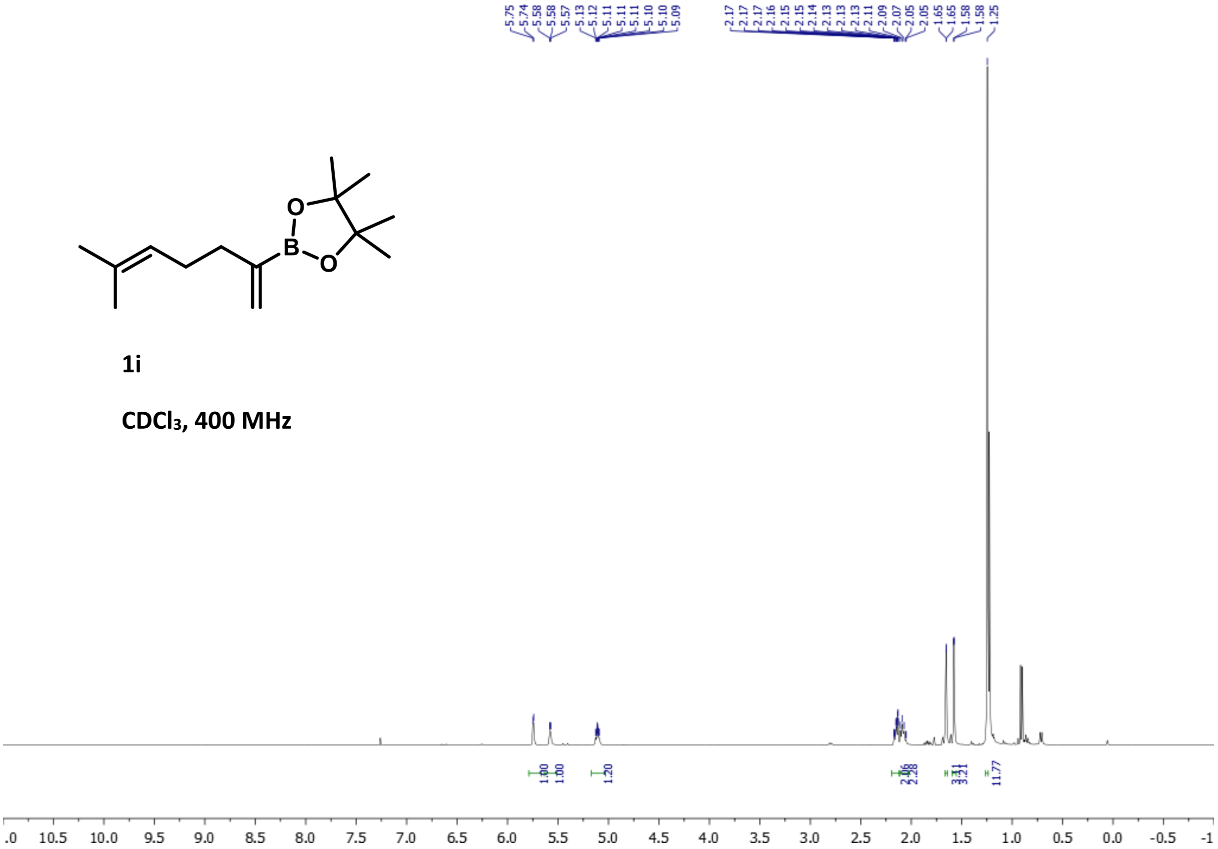

1i

$CDCl_3$, 400 MHz

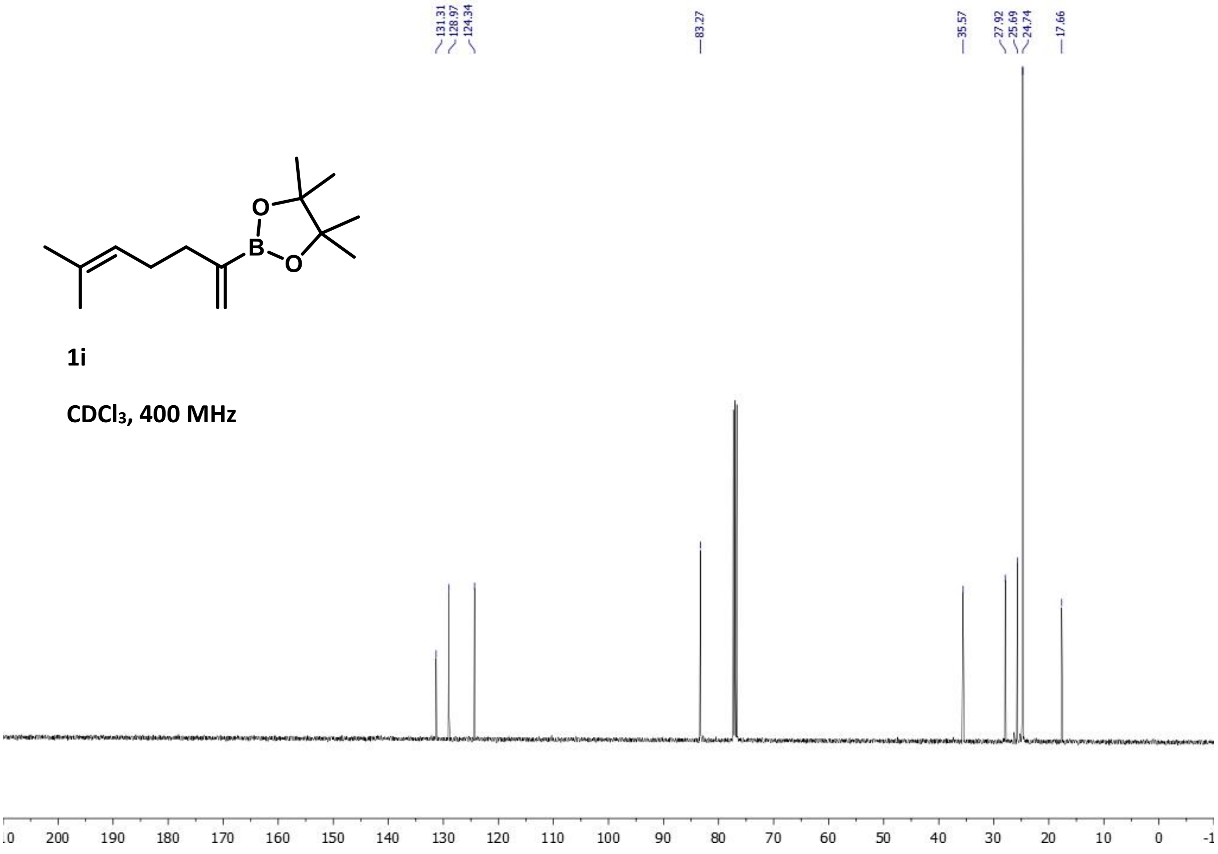

1i

$CDCl_3$, 400 MHz

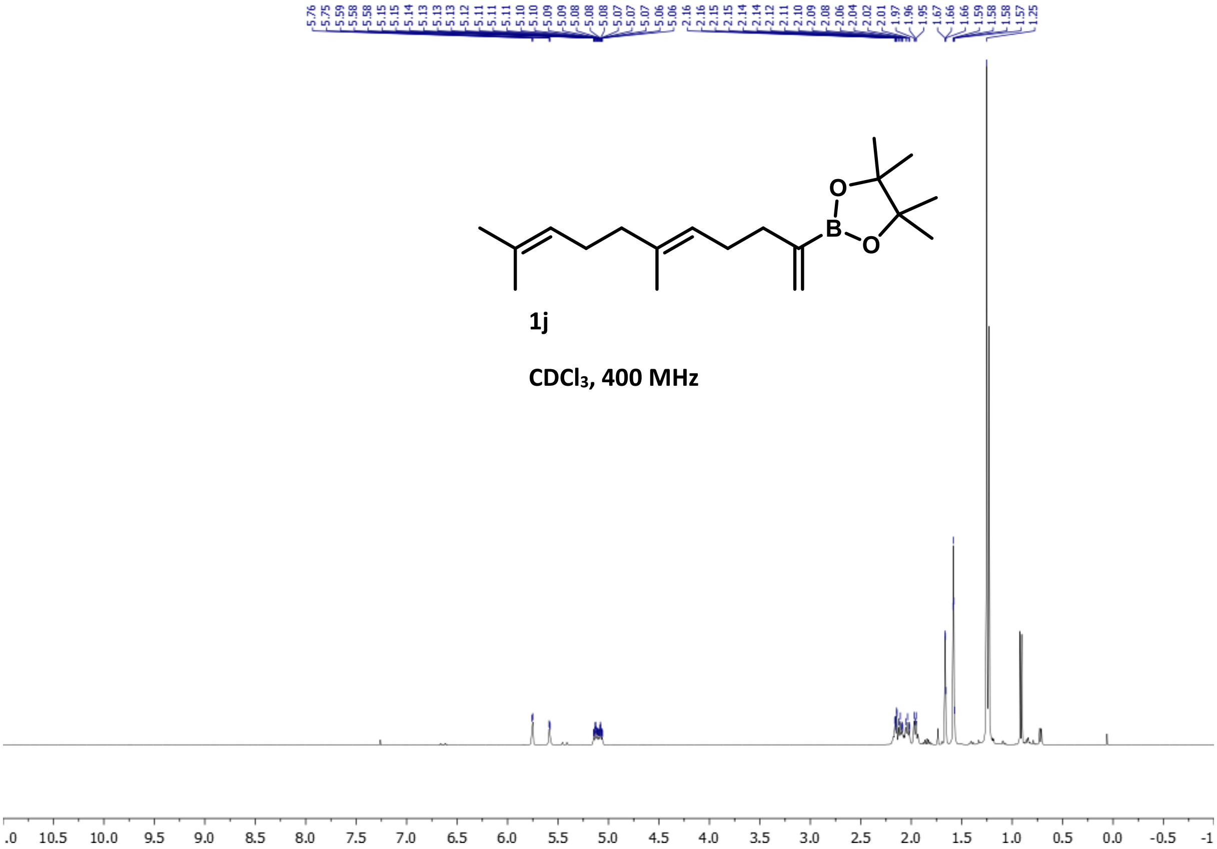

1j

CDCl₃, 400 MHz

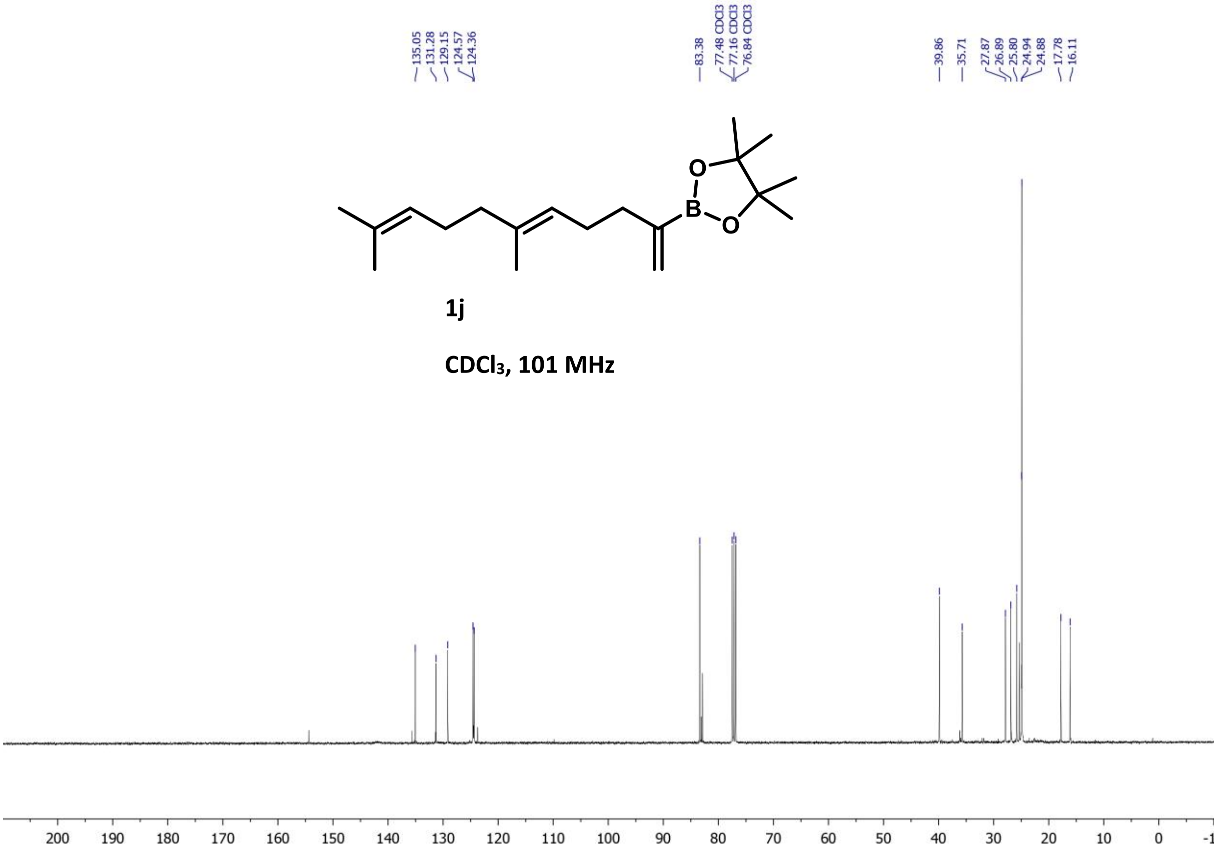

1j

CDCl₃, 101 MHz

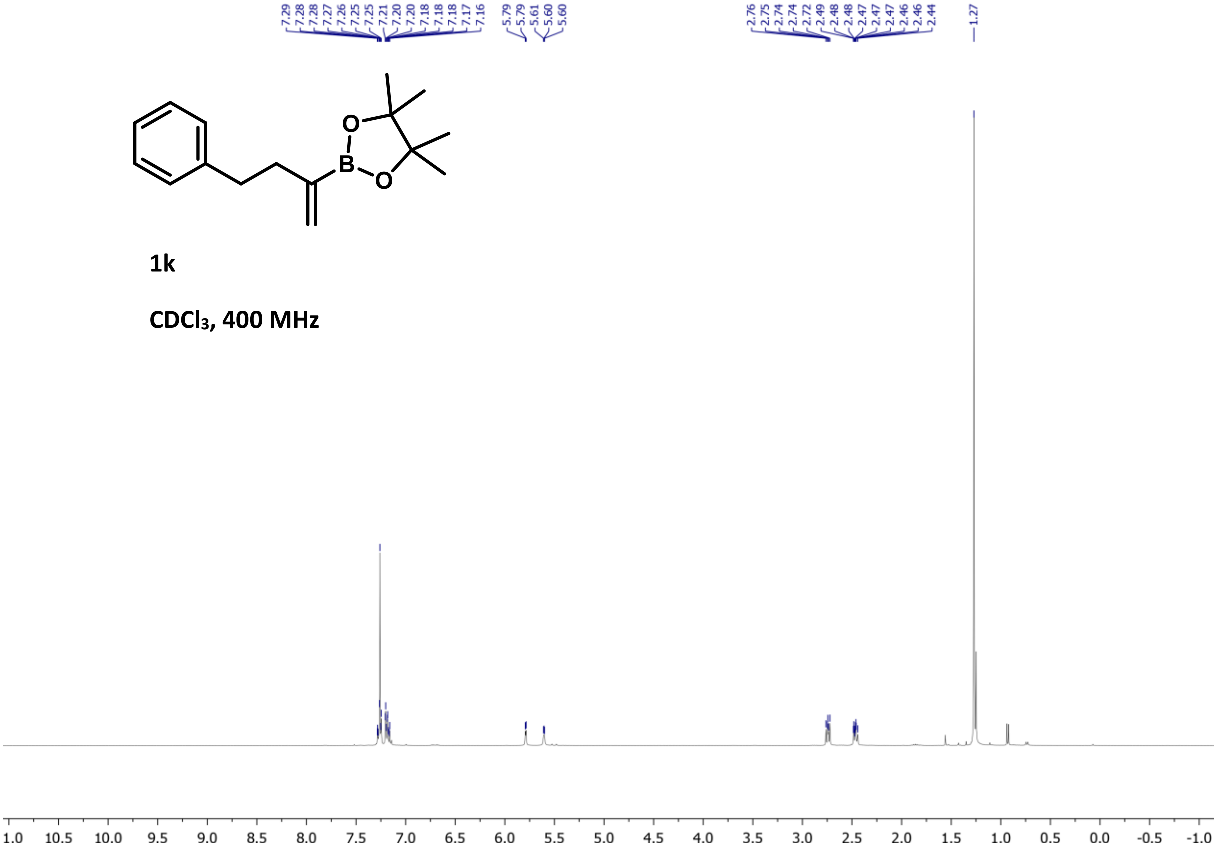
7.29
7.28
7.28
7.27
7.26
7.25
7.25
7.21
7.20
7.20
7.18
7.18
7.18
7.17
7.16
5.79
5.79
5.61
5.60
5.60
2.76
2.75
2.74
2.74
2.72
2.49
2.48
2.48
2.47
2.47
2.47
2.46
2.46
2.44
1.27
O
B
O
1k
CDCl₃, 400 MHz
1.0
10.5
10.0
9.5
9.0
8.5
8.0
7.5
7.0
6.5
6.0
5.5
5.0
4.5
4.0
3.5
3.0
2.5
2.0
1.5
1.0
0.5
0.0
-0.5
-1.0

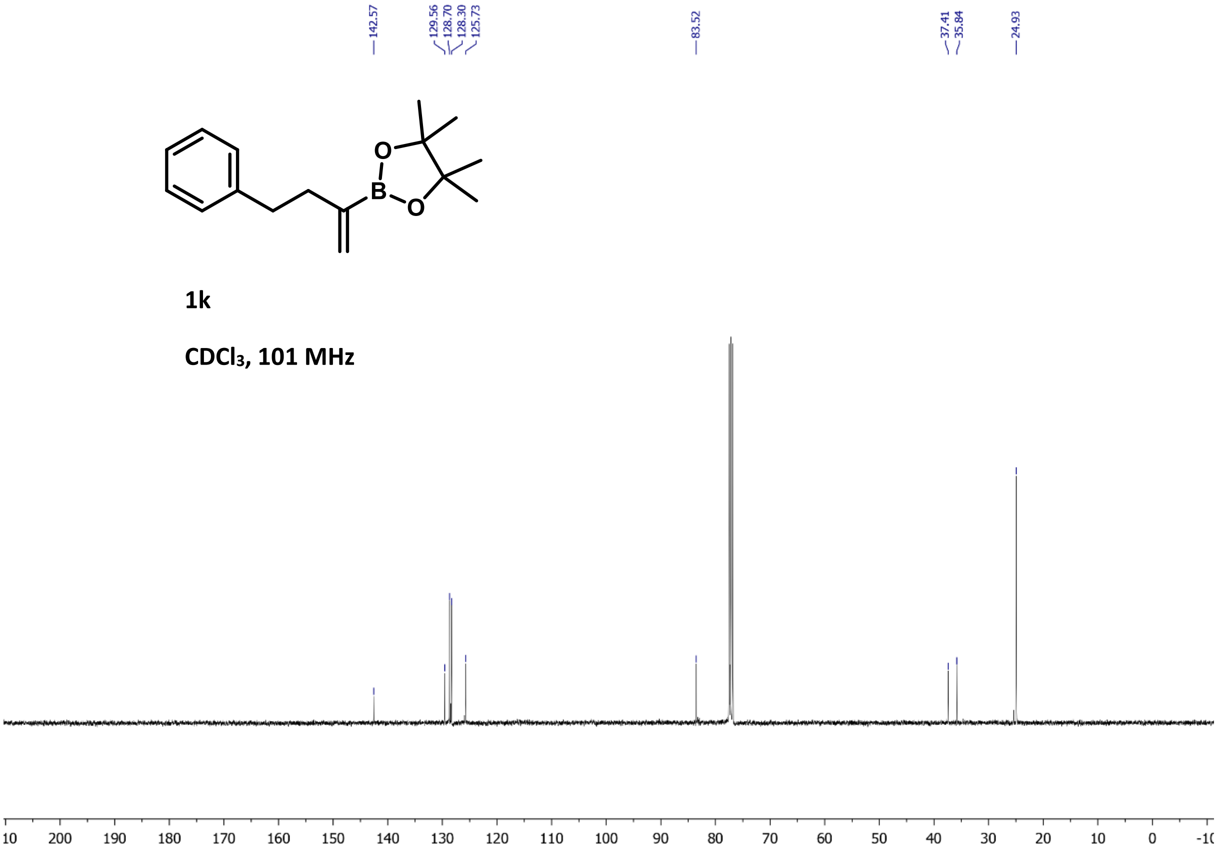
142.57
129.56
128.70
128.30
125.73
83.52
37.41
35.84
24.93
O
B
O
1k
CDCl₃, 101 MHz
10
200
190
180
170
160
150
140
130
120
110
100
90
80
70
60
50
40
30
20
10
0
-1(

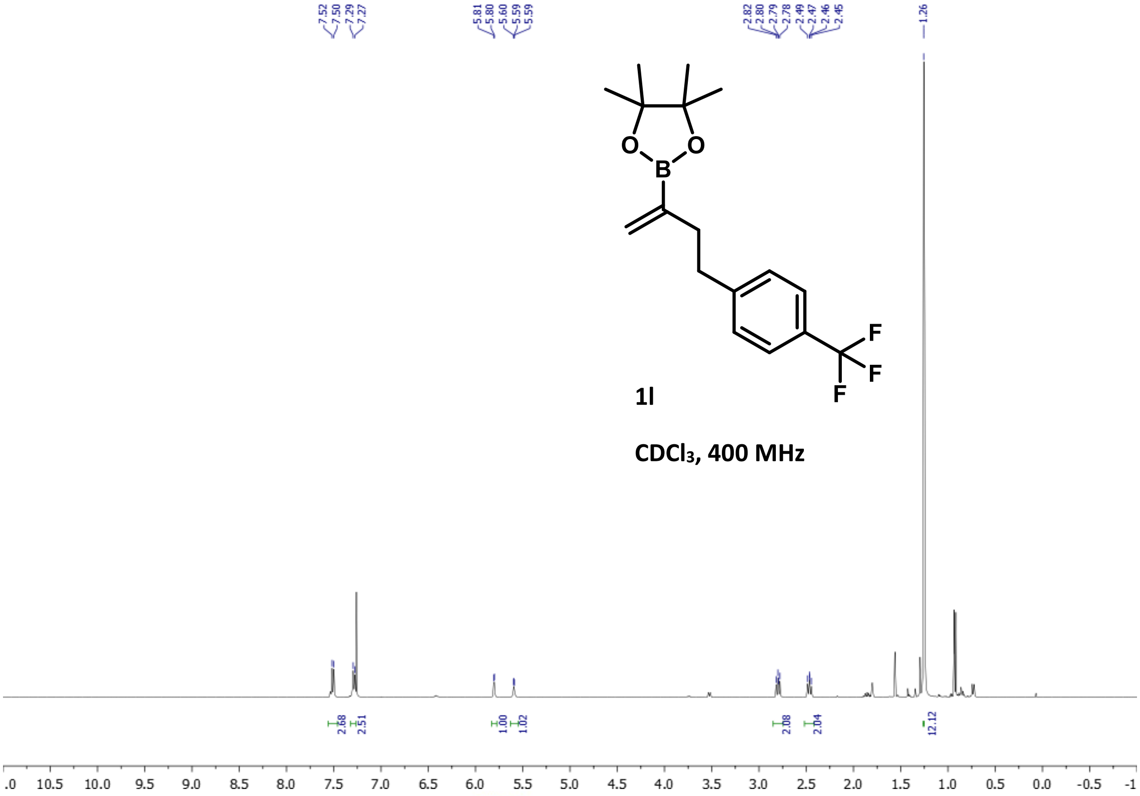
7.52
7.50
7.29
7.27
5.81
5.80
5.60
5.59
5.59
2.82
2.80
2.79
2.78
2.49
2.47
2.46
2.45
1.26
O
B
O
F
F
F
1l
CDCl3, 400 MHz
2.68
2.51
1.00
1.02
2.08
2.04
12.12
.0 10.5 10.0 9.5 9.0 8.5 8.0 7.5 7.0 6.5 6.0 5.5 5.0 4.5 4.0 3.5 3.0 2.5 2.0 1.5 1.0 0.5 0.0 -0.5 -1

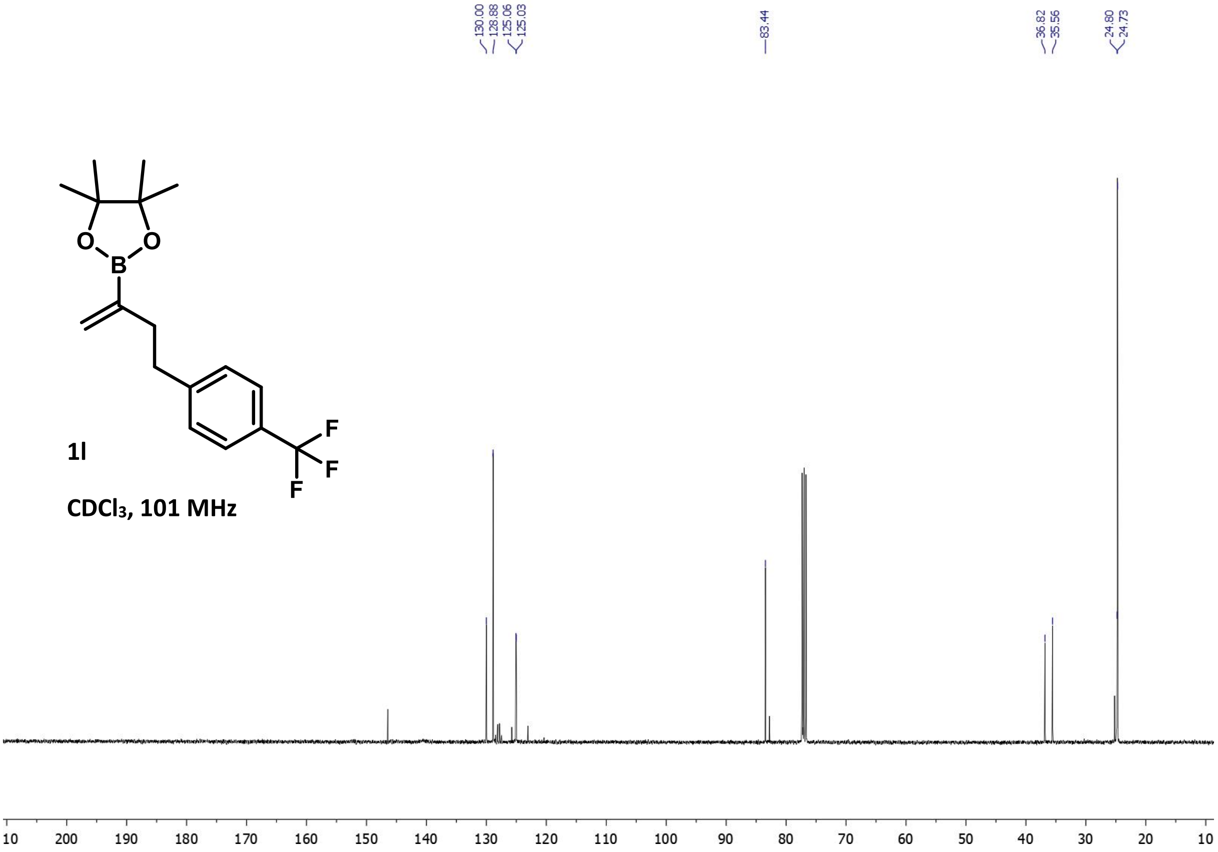
130.00
128.88
125.06
125.03
83.44
36.82
35.56
24.80
24.73
O
B
O
F
F
F
1l
CDCl3, 101 MHz
10 200 190 180 170 160 150 140 130 120 110 100 90 80 70 60 50 40 30 20 10

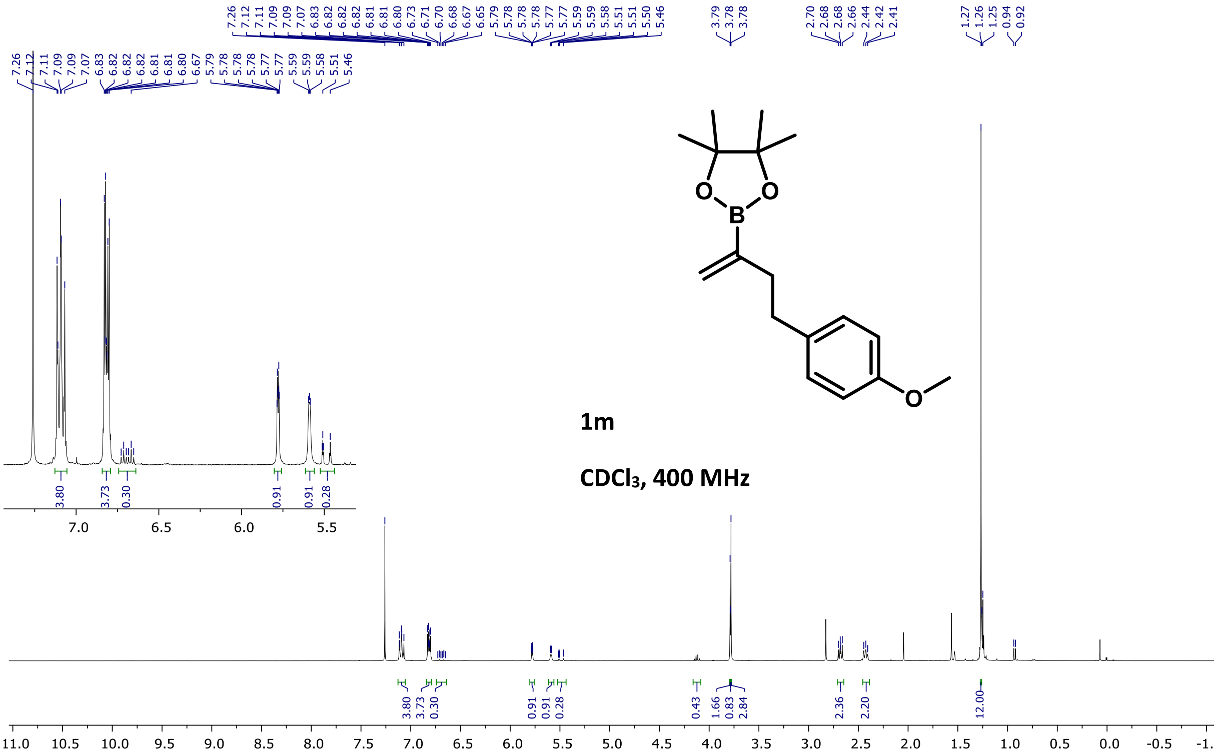

1m

CDCl₃, 400 MHz

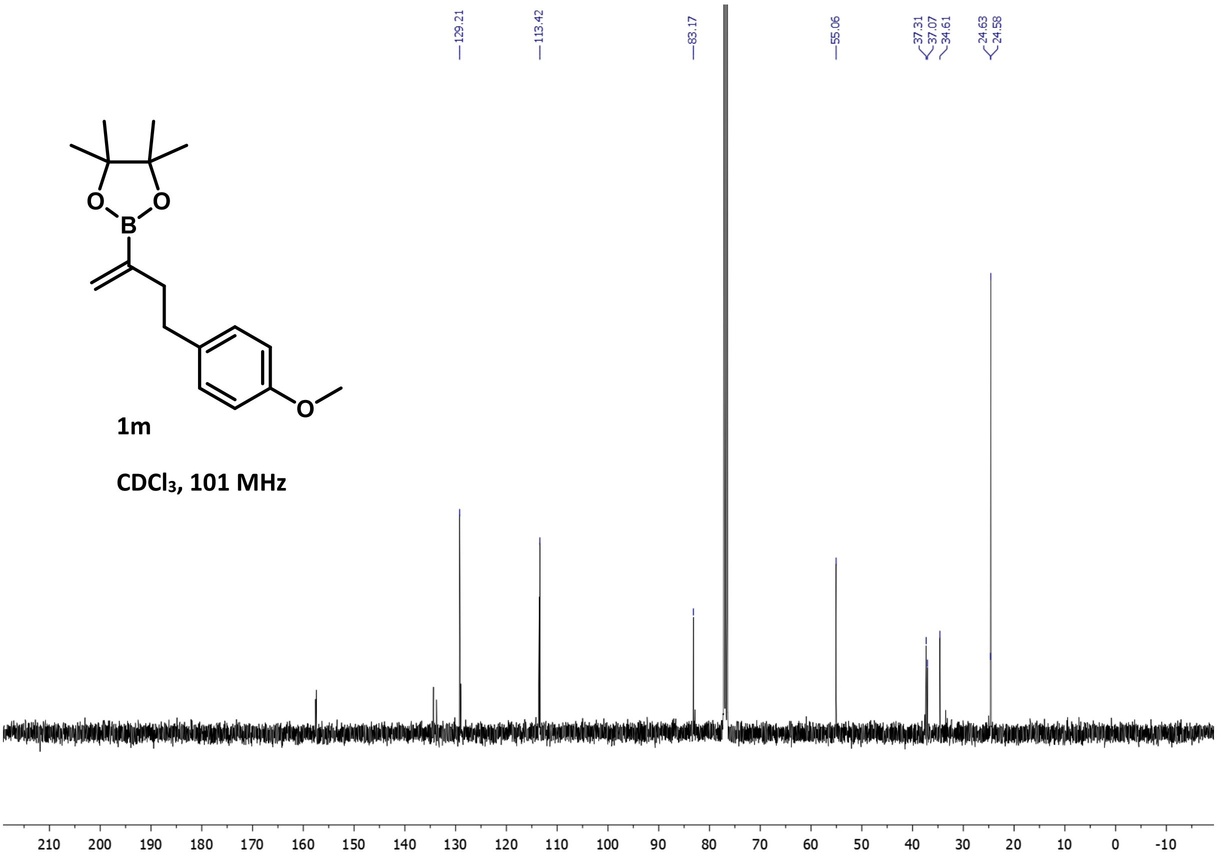

1m

CDCl₃, 101 MHz

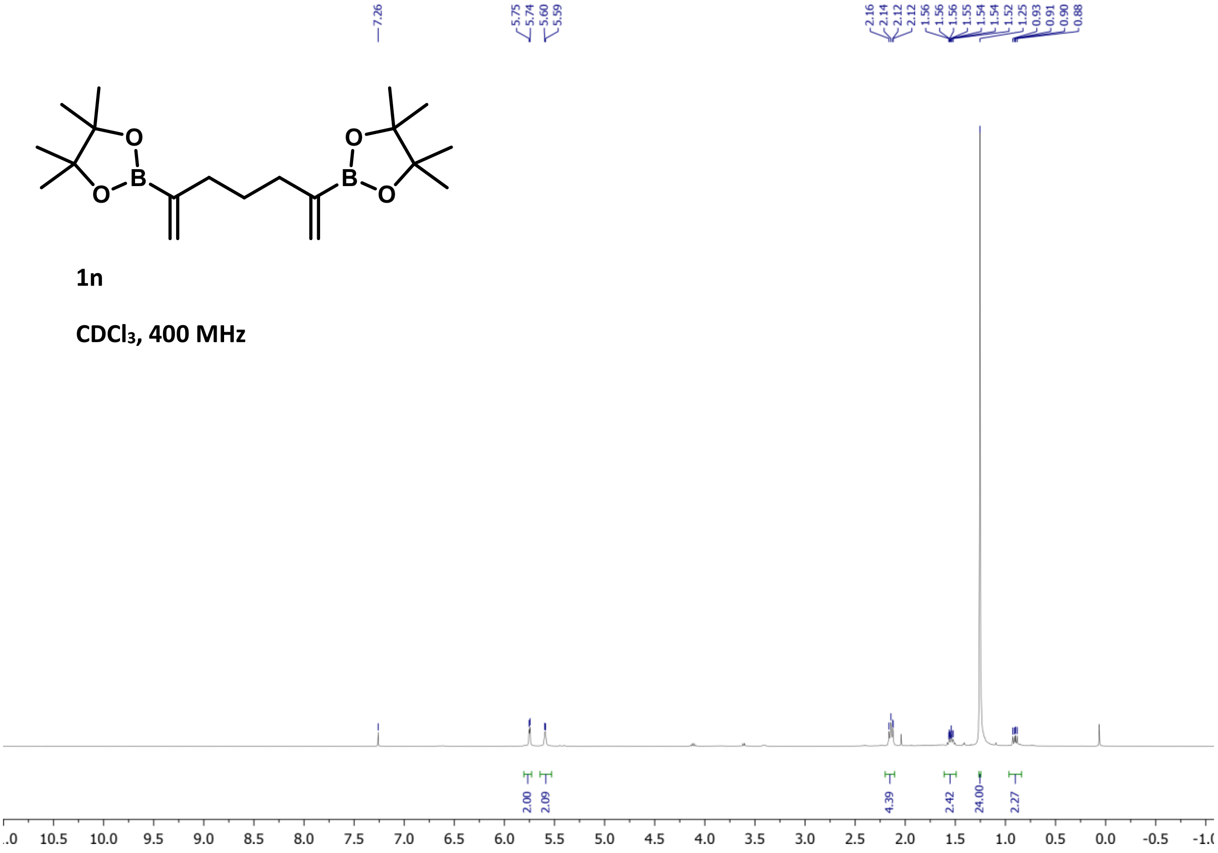
1n
CDCl3, 400 MHz
7.26
5.75 5.74 5.60 5.59
2.16 2.14 2.12 2.12 1.56 1.56 1.56 1.55 1.54 1.54 1.54 1.52 1.25 0.93 0.91 0.90 0.88
2.00 2.09 4.39 2.42 24.00 2.27

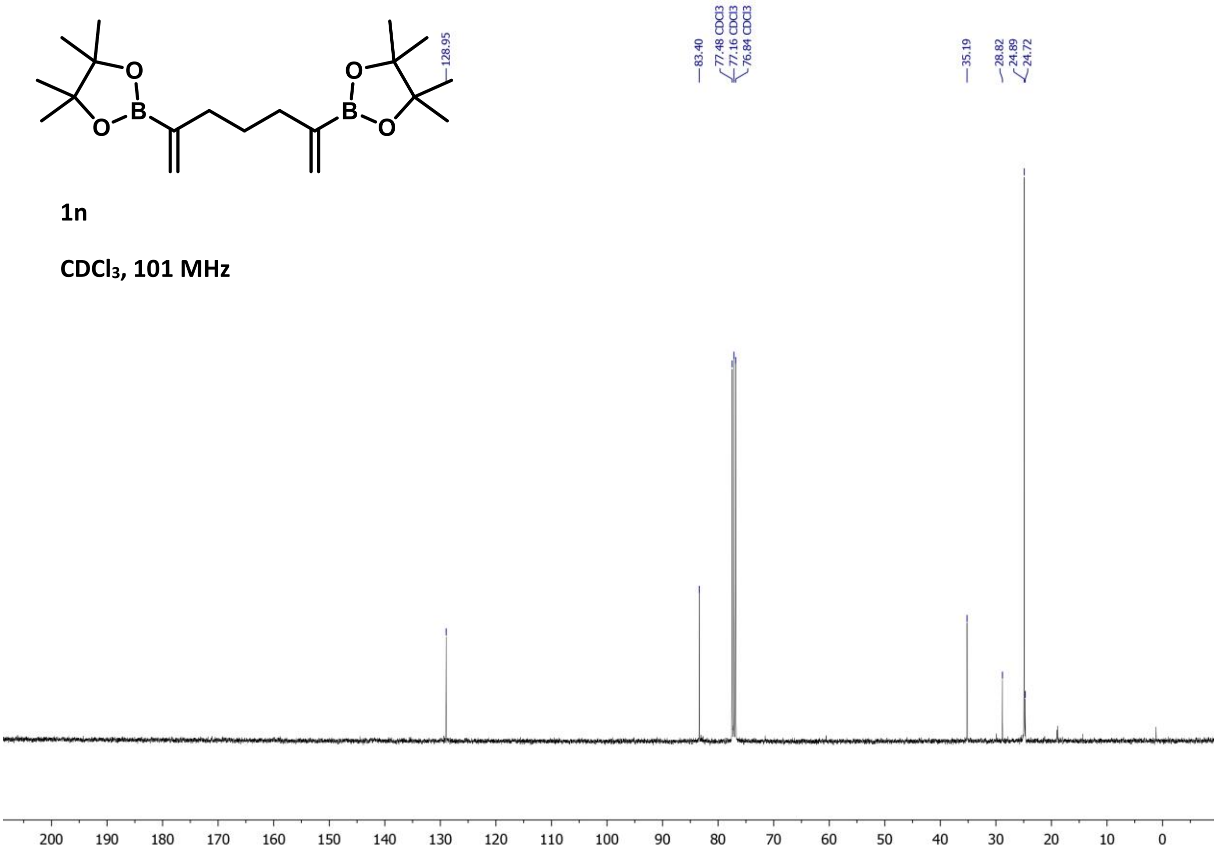
1n
CDCl3, 101 MHz
128.95
83.40
77.48 CDCl3
77.16 CDCl3
76.84 CDCl3
35.19
28.82 24.89 24.72

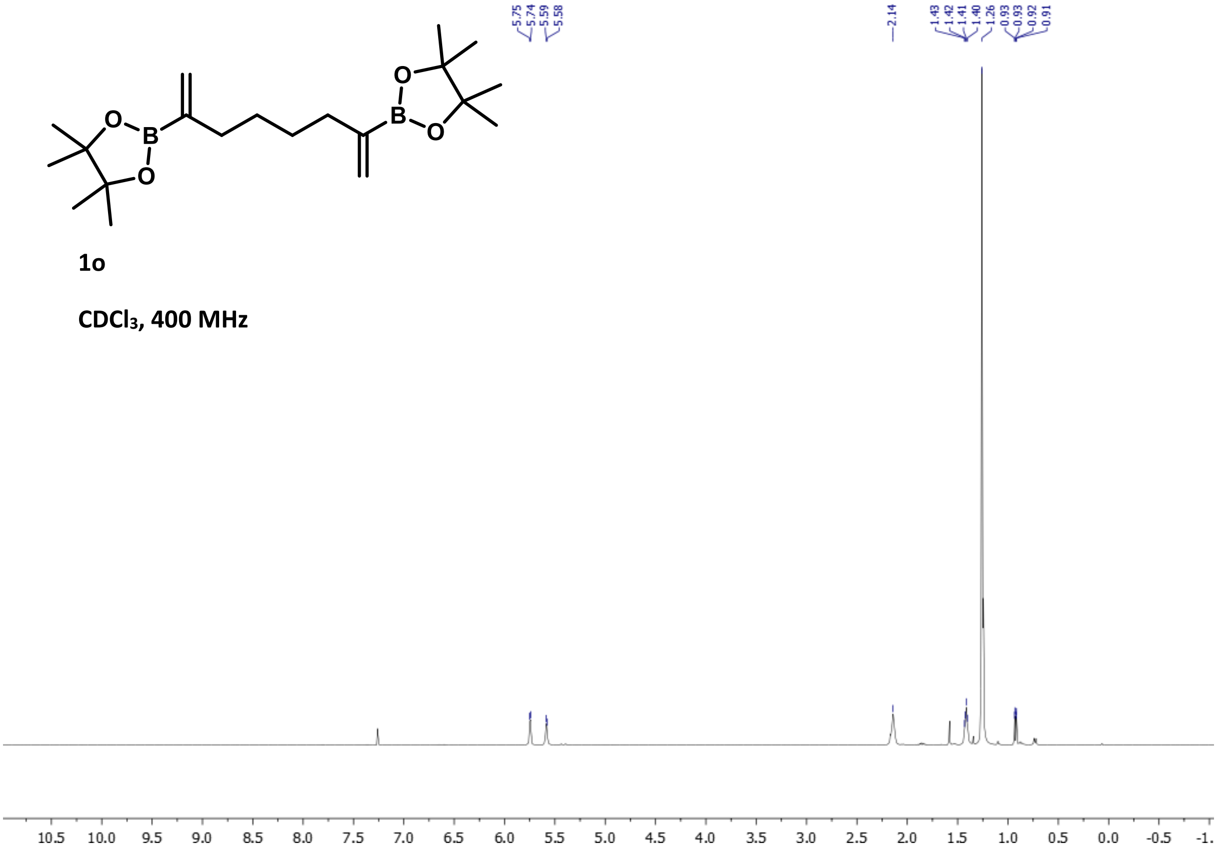

1o

$CDCl_3$, 400 MHz

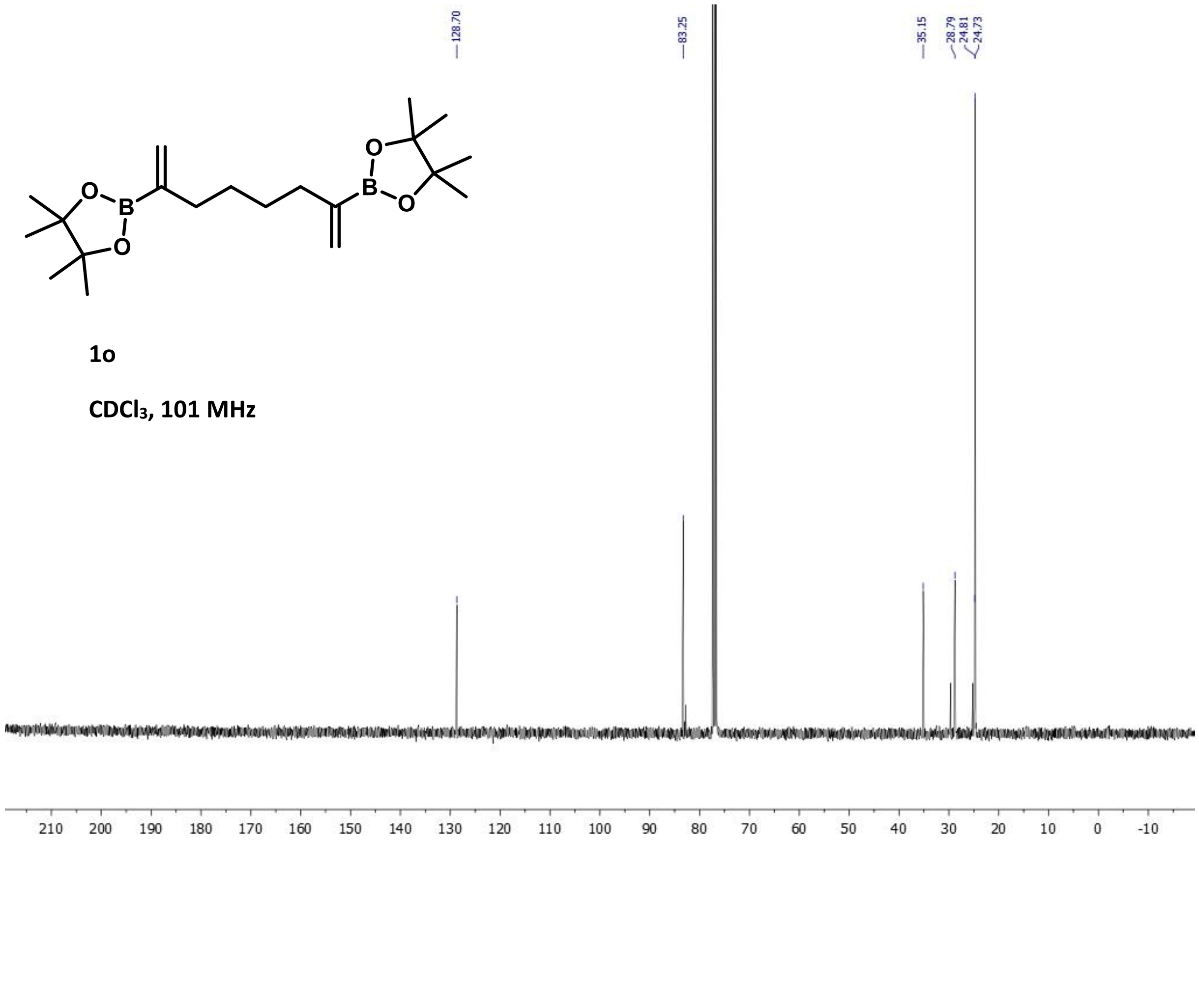

1o

$CDCl_3$, 101 MHz

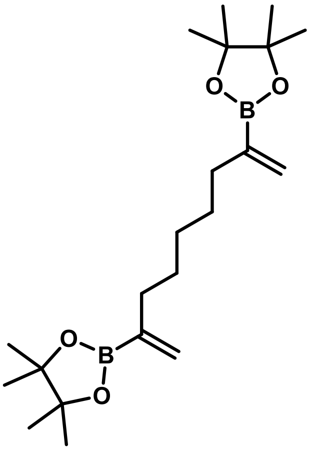

1p

$CDCl_3$, 400 MHz

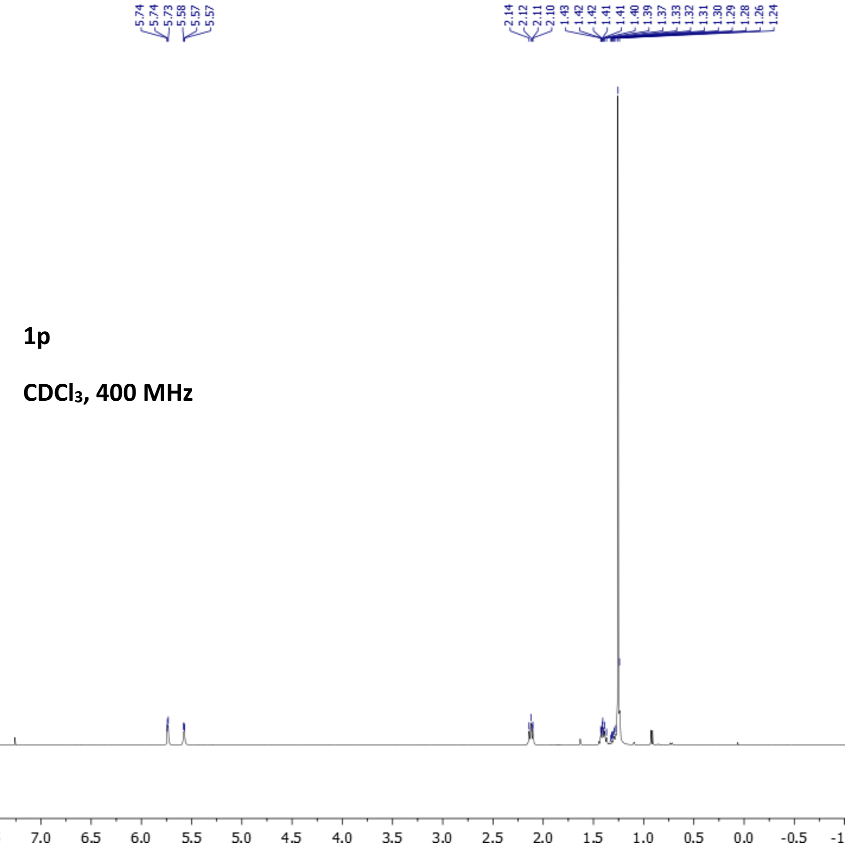

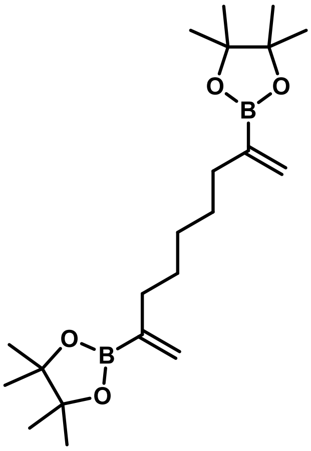

1p

$CDCl_3$, 101 MHz

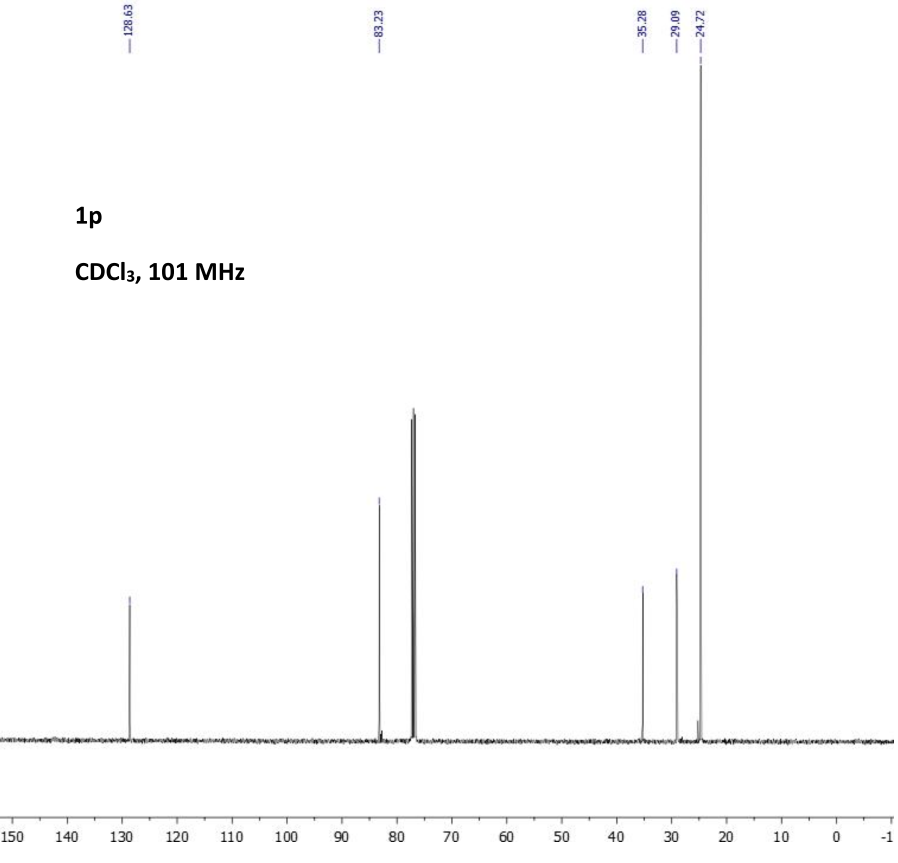

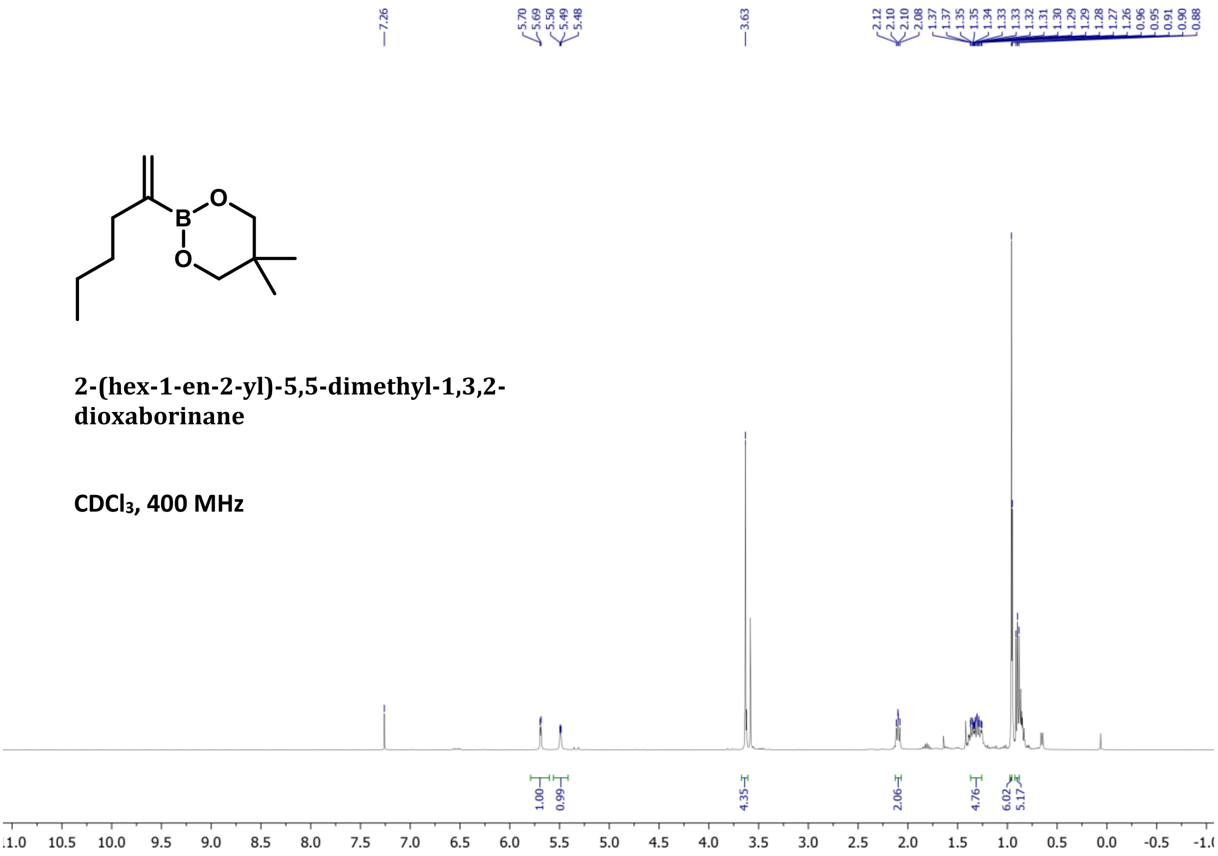

2-(hex-1-en-2-yl)-5,5-dimethyl-1,3,2-dioxaborinane

CDCl₃, 400 MHz

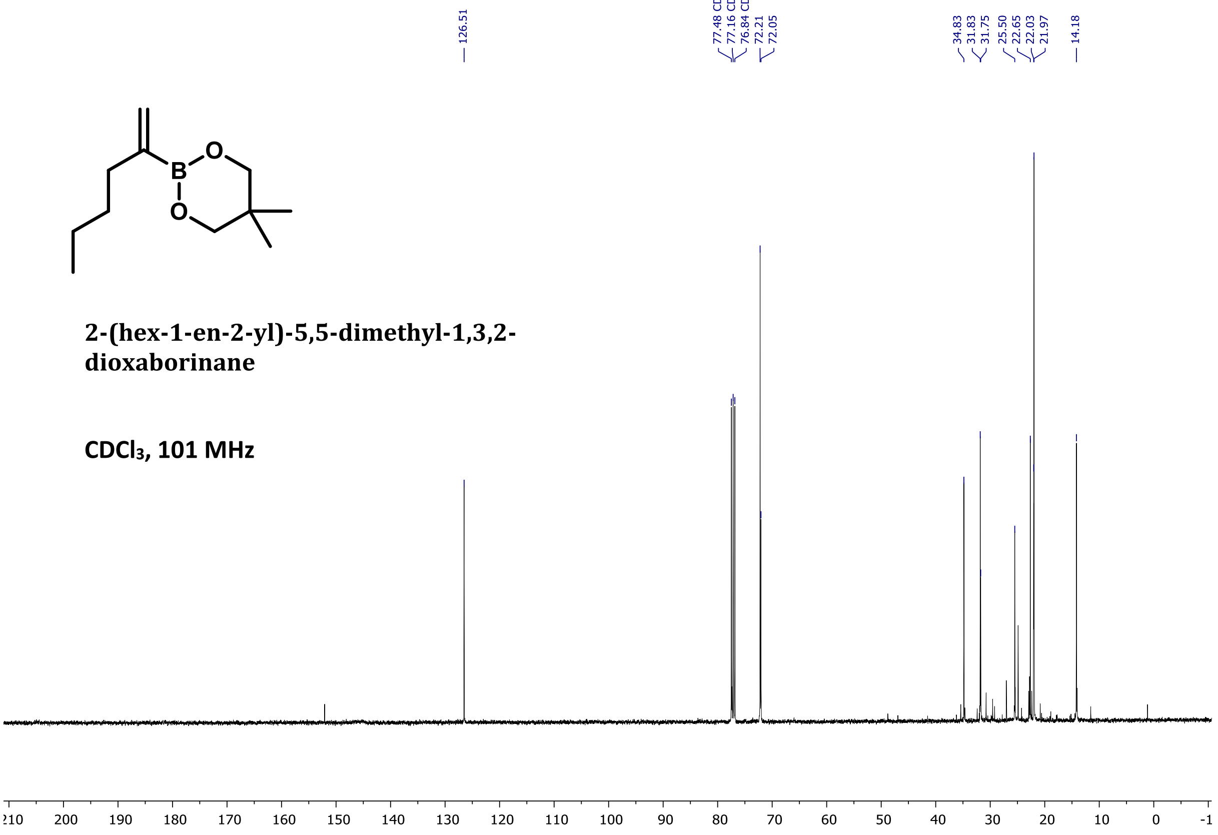

2-(hex-1-en-2-yl)-5,5-dimethyl-1,3,2-dioxaborinane

CDCl₃, 101 MHz

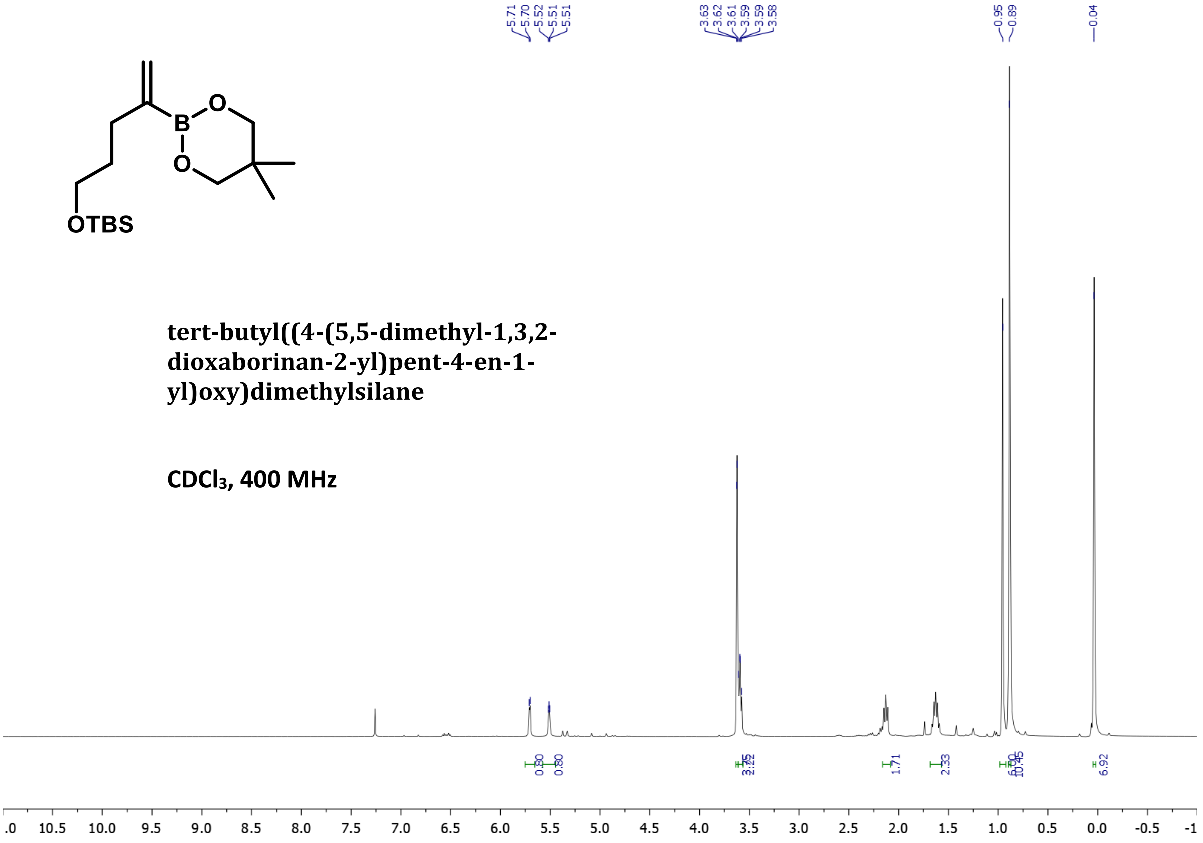
5.71
5.70
5.52
5.51
5.51
3.63
3.62
3.61
3.59
3.59
3.58
0.95
0.89
0.04
OTBS
tert-butyl((4-(5,5-dimethyl-1,3,2-dioxaborinan-2-yl)pent-4-en-1-yl)oxy)dimethylsilane
CDCl₃, 400 MHz
1.71
2.33
10.45
6.92

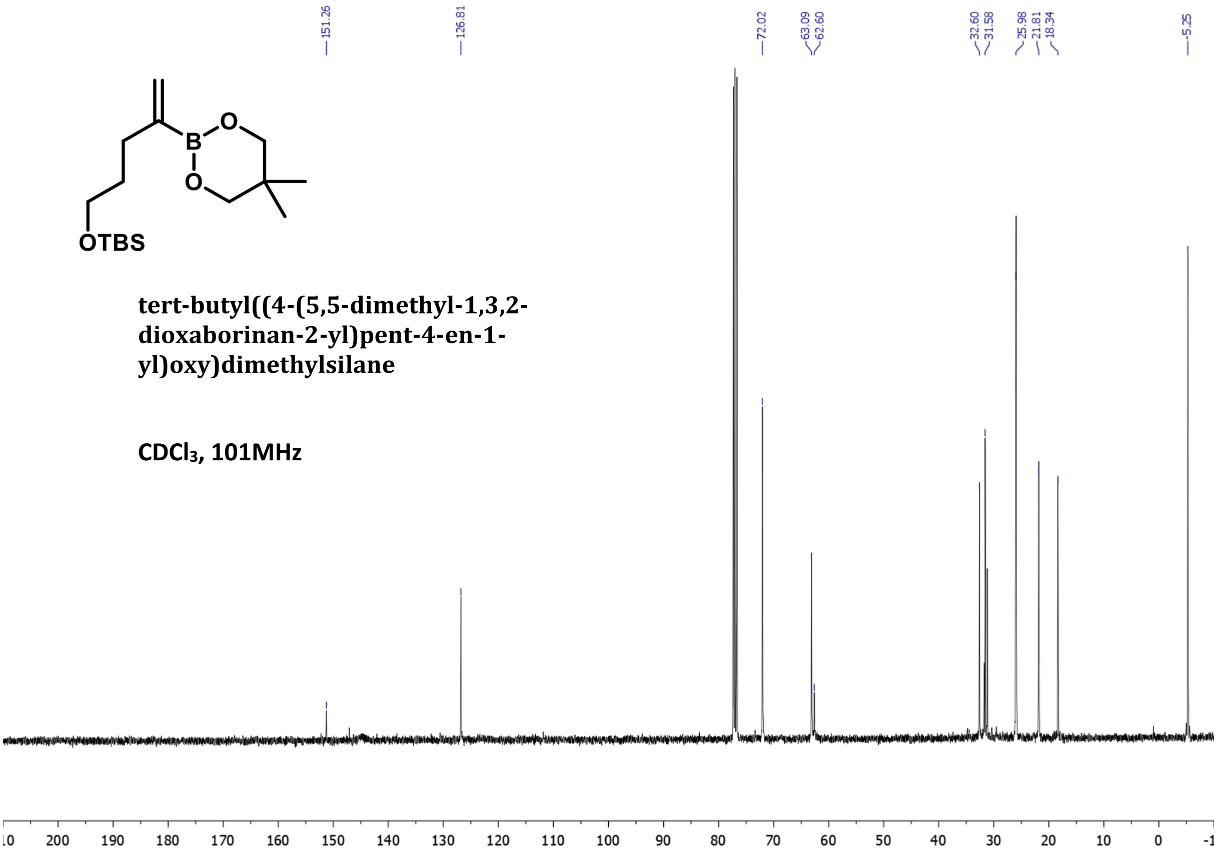
151.26
126.81
72.02
63.09
62.60
32.60
31.58
25.98
21.81
18.34
-5.25
OTBS
tert-butyl((4-(5,5-dimethyl-1,3,2-dioxaborinan-2-yl)pent-4-en-1-yl)oxy)dimethylsilane
CDCl₃, 101MHz

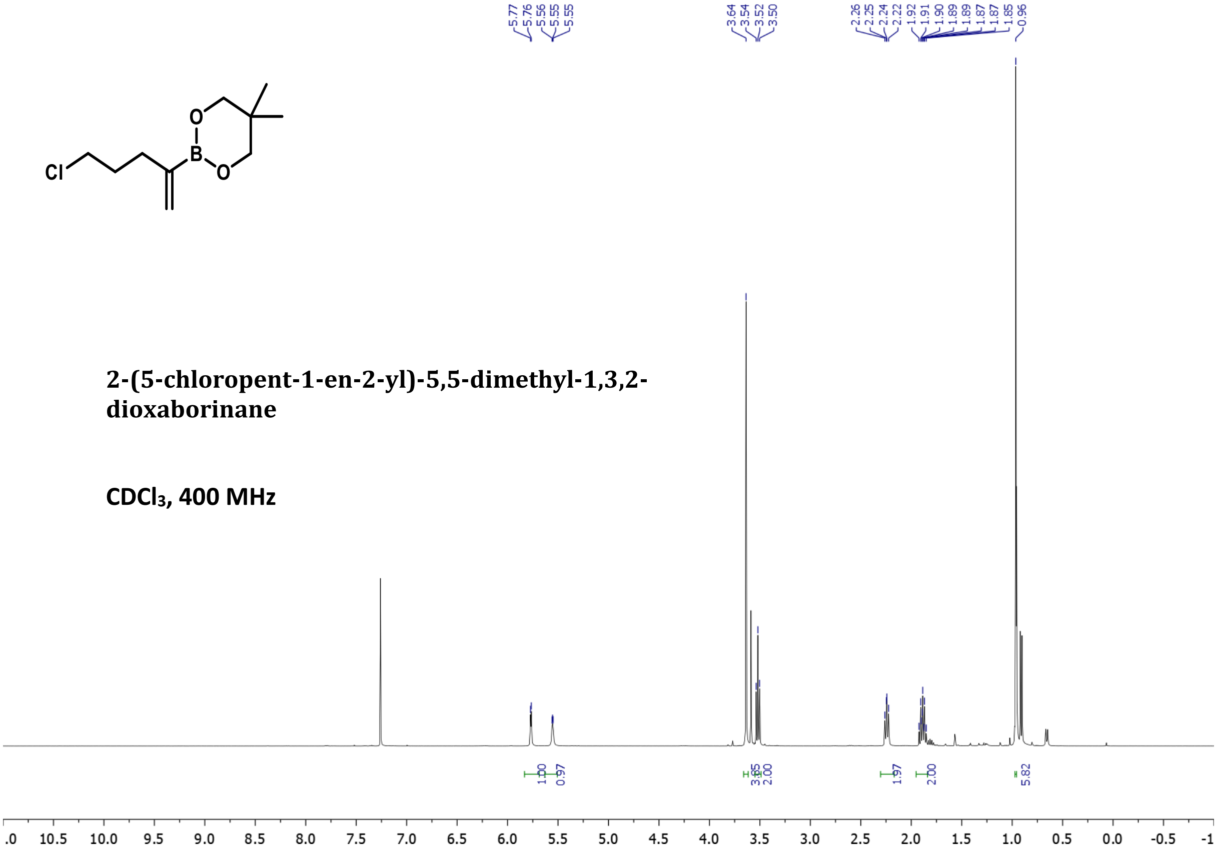

2-(5-chloropent-1-en-2-yl)-5,5-dimethyl-1,3,2-dioxaborinane

CDCl$_3$, 400 MHz

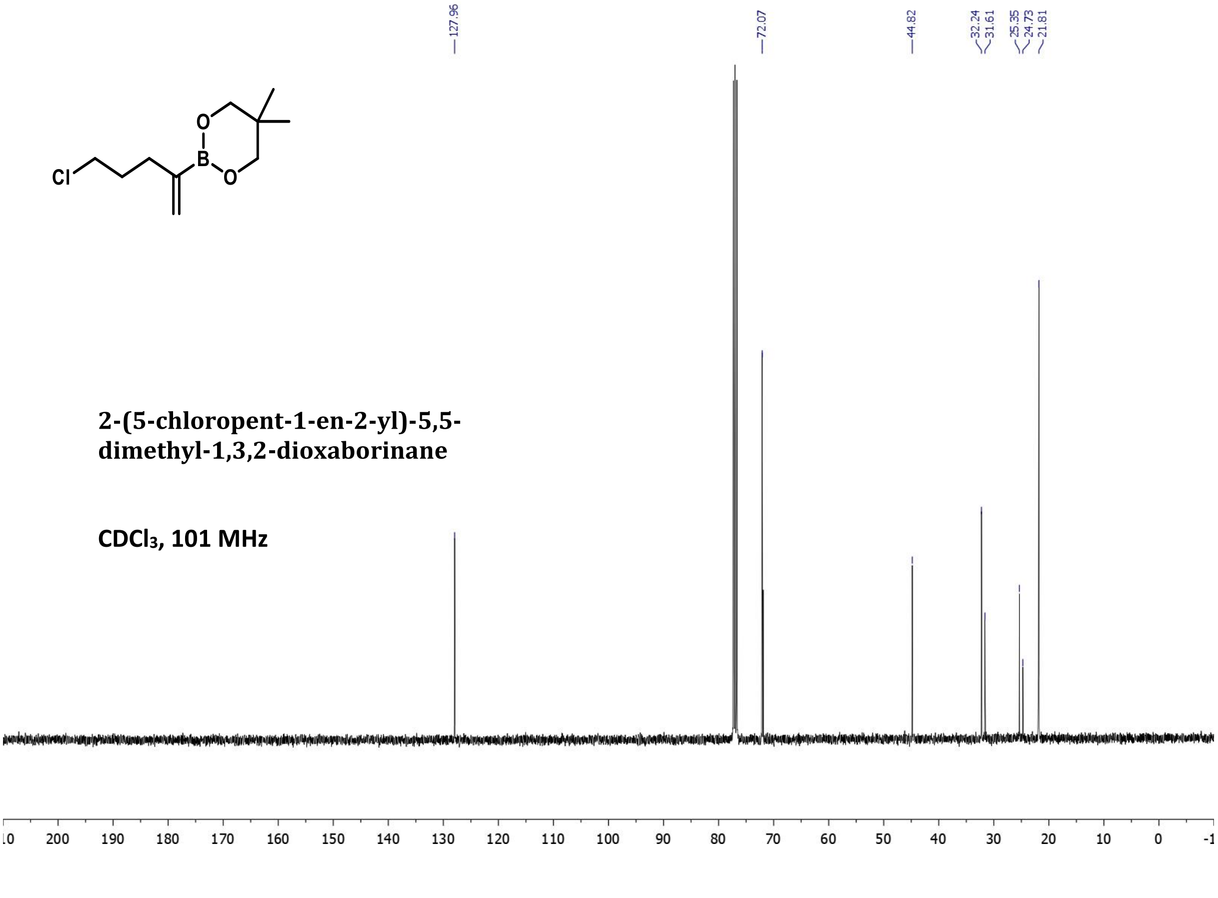

2-(5-chloropent-1-en-2-yl)-5,5-dimethyl-1,3,2-dioxaborinane

CDCl$_3$, 101 MHz

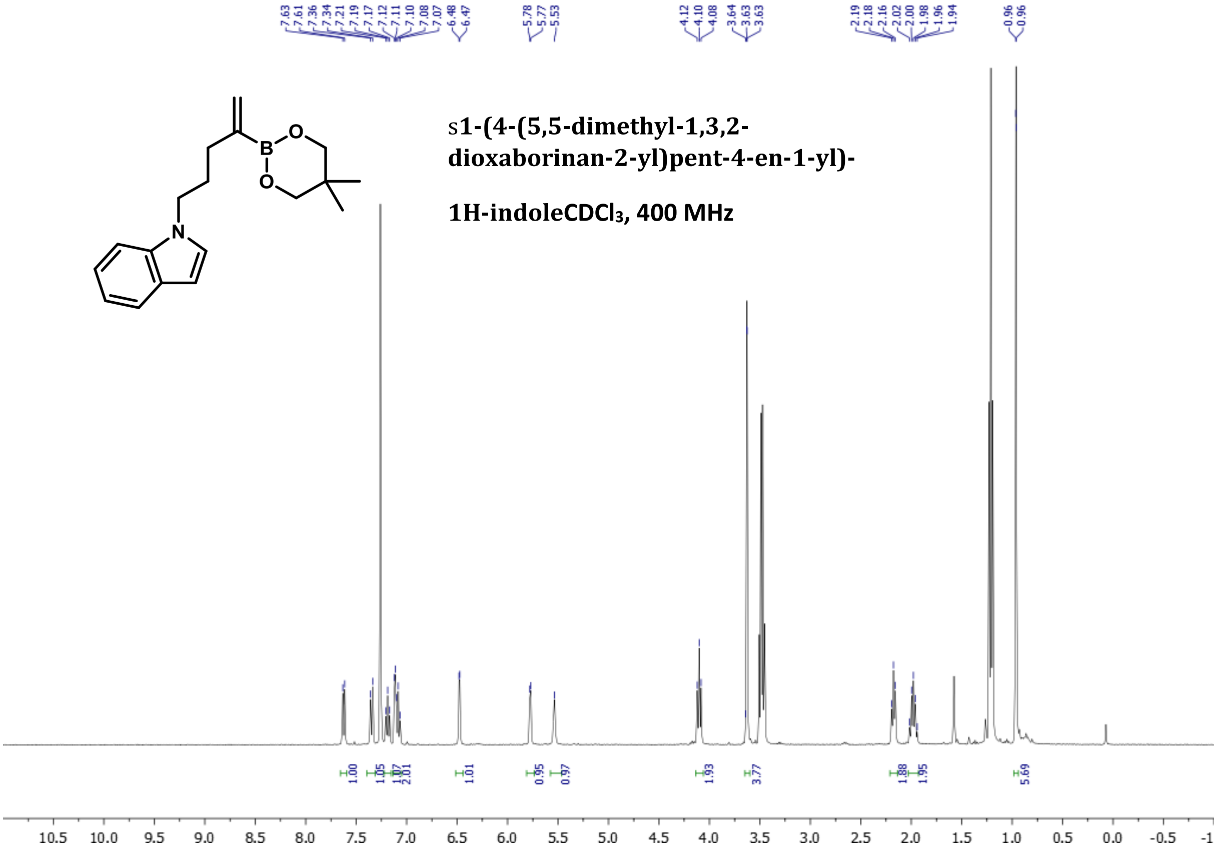

s1-(4-(5,5-dimethyl-1,3,2-dioxaborinan-2-yl)pent-4-en-1-yl)-1H-indoleCDCl₃, 400 MHz

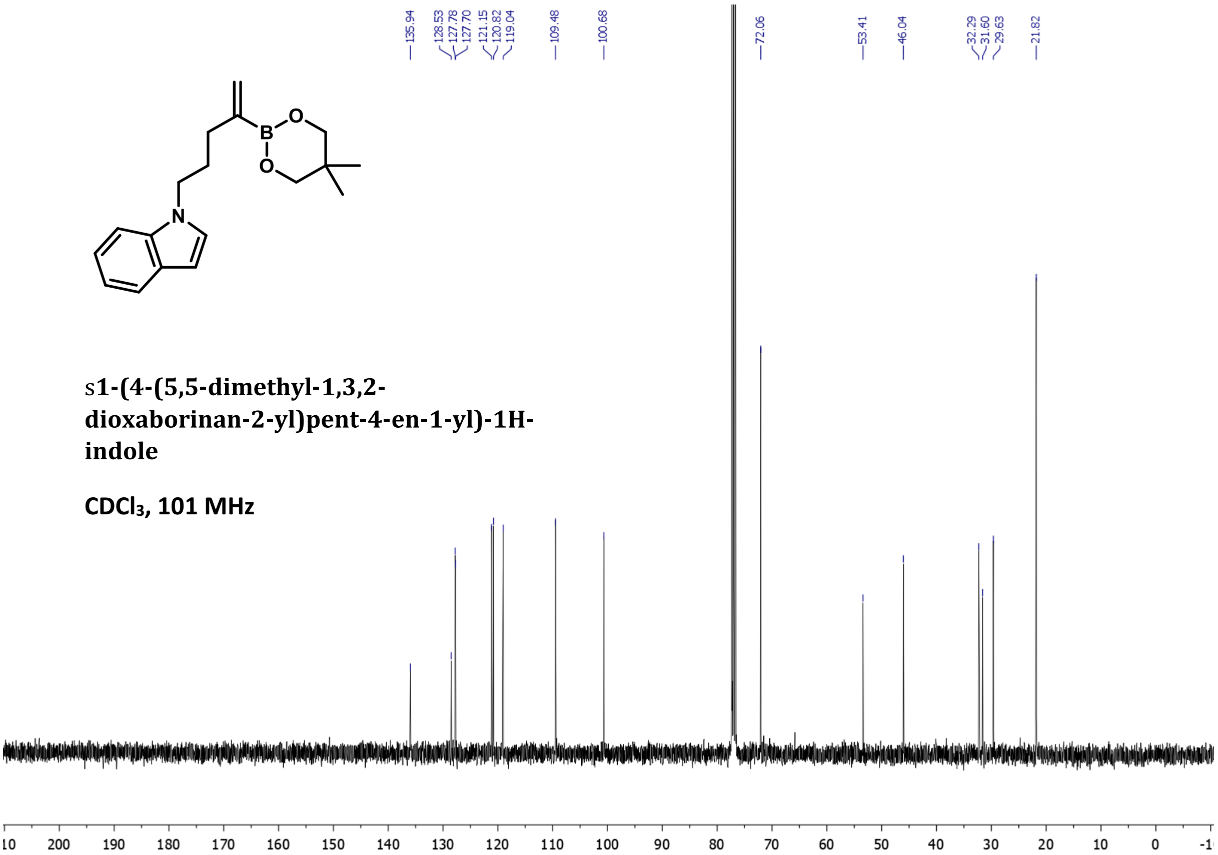

s1-(4-(5,5-dimethyl-1,3,2-dioxaborinan-2-yl)pent-4-en-1-yl)-1H-indole

$CDCl_3$, 101 MHz

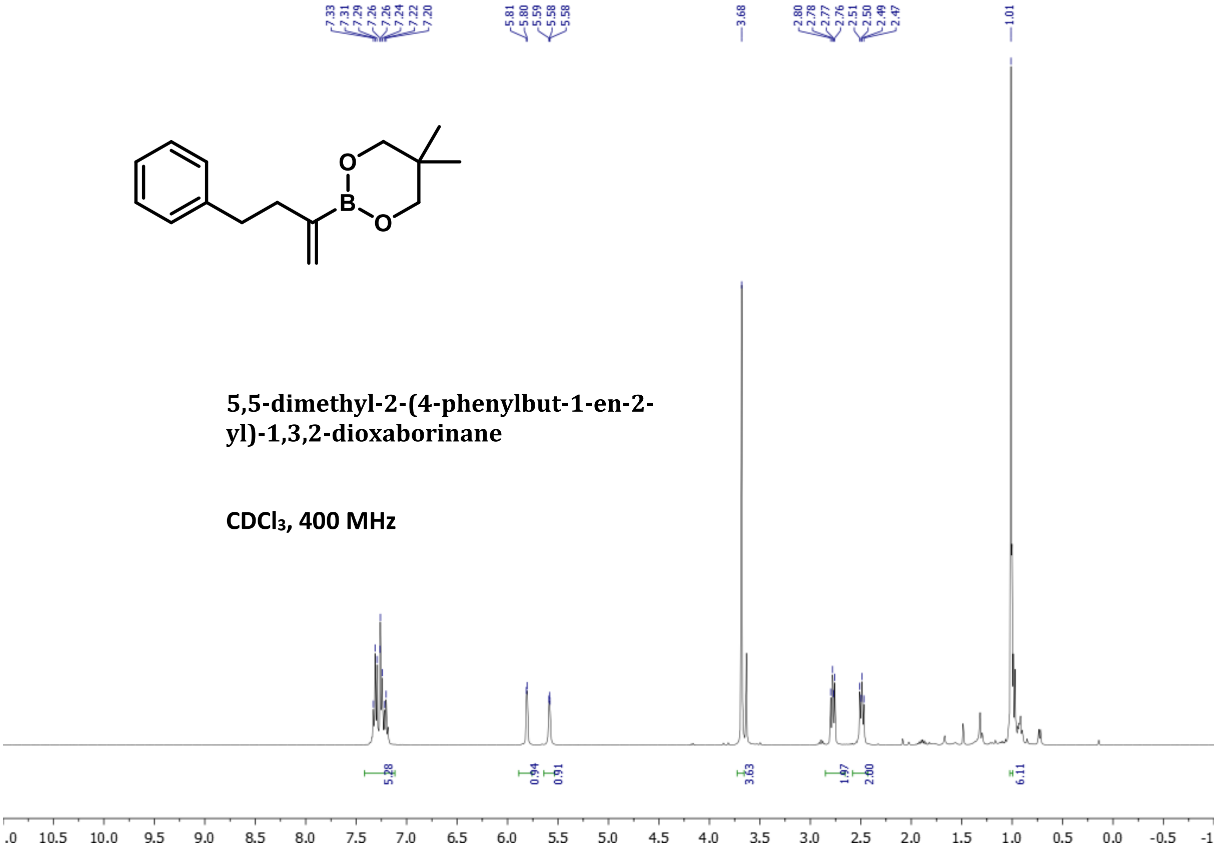
5,5-dimethyl-2-(4-phenylbut-1-en-2-yl)-1,3,2-dioxaborinane
CDCl₃, 400 MHz

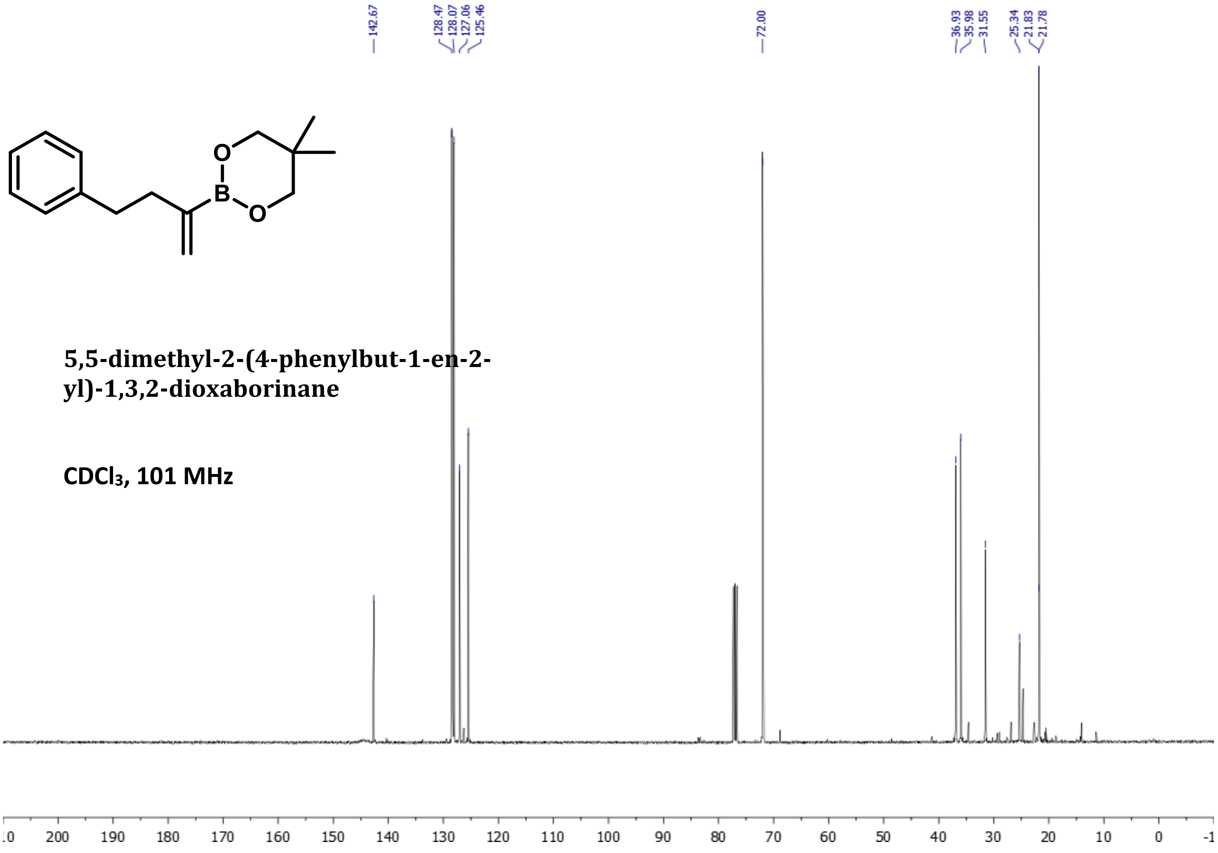
5,5-dimethyl-2-(4-phenylbut-1-en-2-yl)-1,3,2-dioxaborinane
CDCl₃, 101 MHz

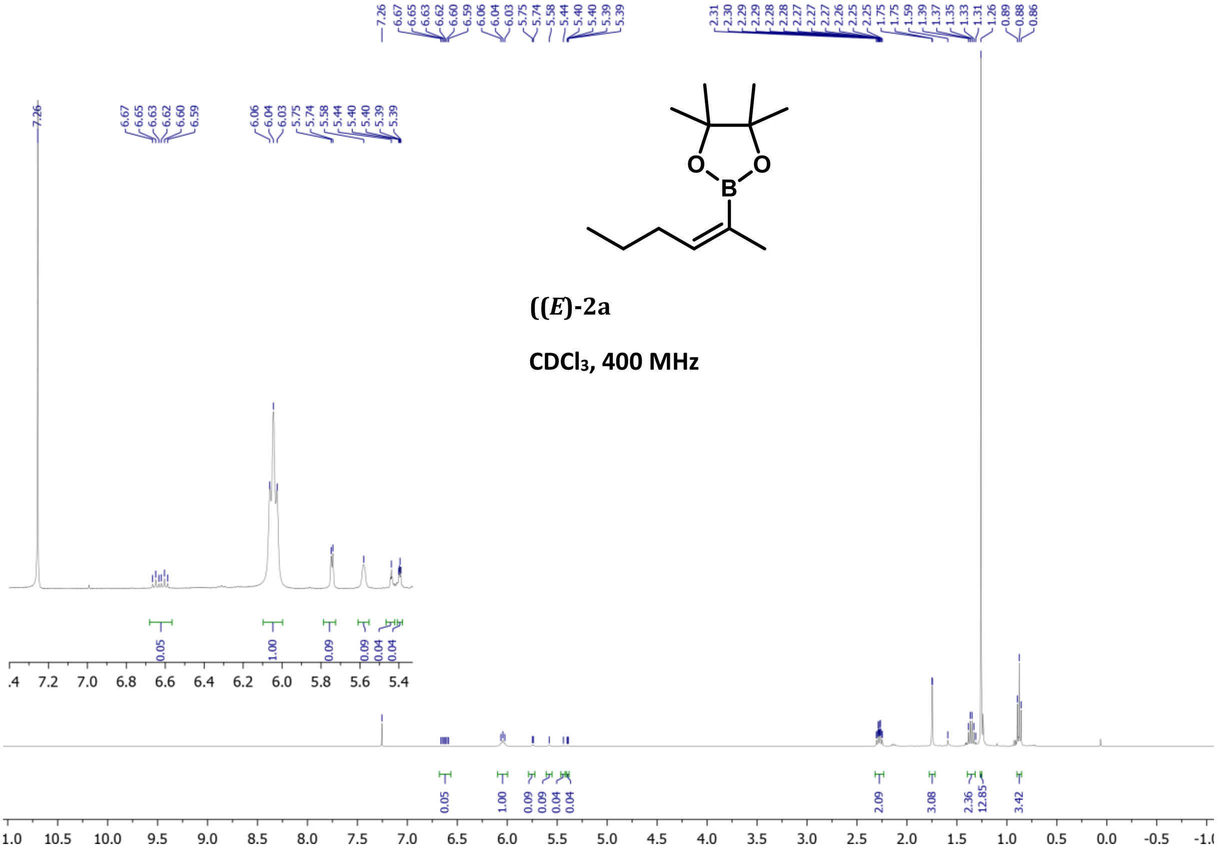
((*E*)-2a)
CDCl₃, 400 MHz
7.26
6.67 6.65 6.63 6.62 6.60 6.59
6.06 6.04 6.03
5.75 5.74
5.58
5.44 5.40 5.40 5.39 5.39
2.31 2.30 2.29 2.29 2.28 2.28 2.27 2.27 2.26 2.25 2.25
1.75 1.75 1.59 1.39 1.37 1.35 1.33 1.31 1.26
0.89 0.88 0.86
0.05 1.00 0.09 0.09 0.04 0.04
2.09 3.08 2.36 12.85 3.42
O B O

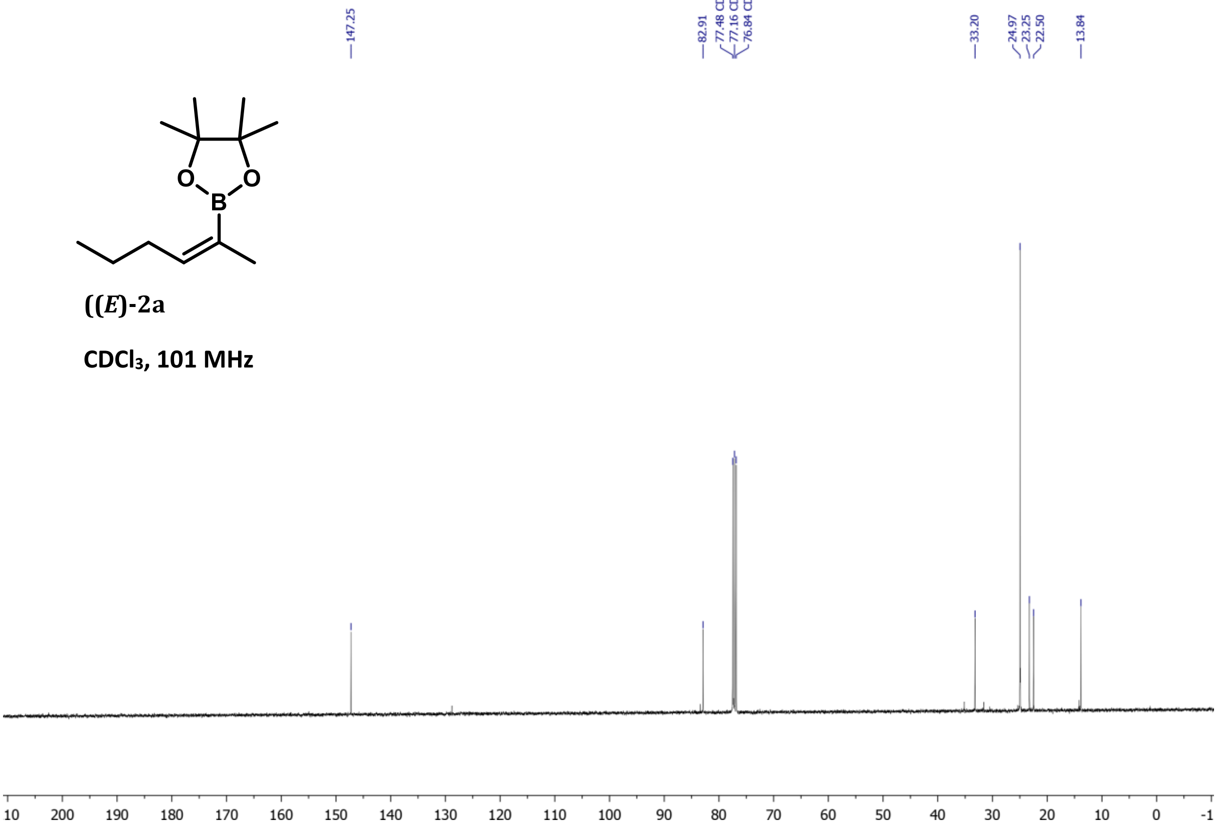
((*E*)-2a)
CDCl₃, 101 MHz
147.25
82.91
77.48 CDCl3
77.16 CDCl3
76.84 CDCl3
33.20
24.97 23.25 22.50
13.84
O B O

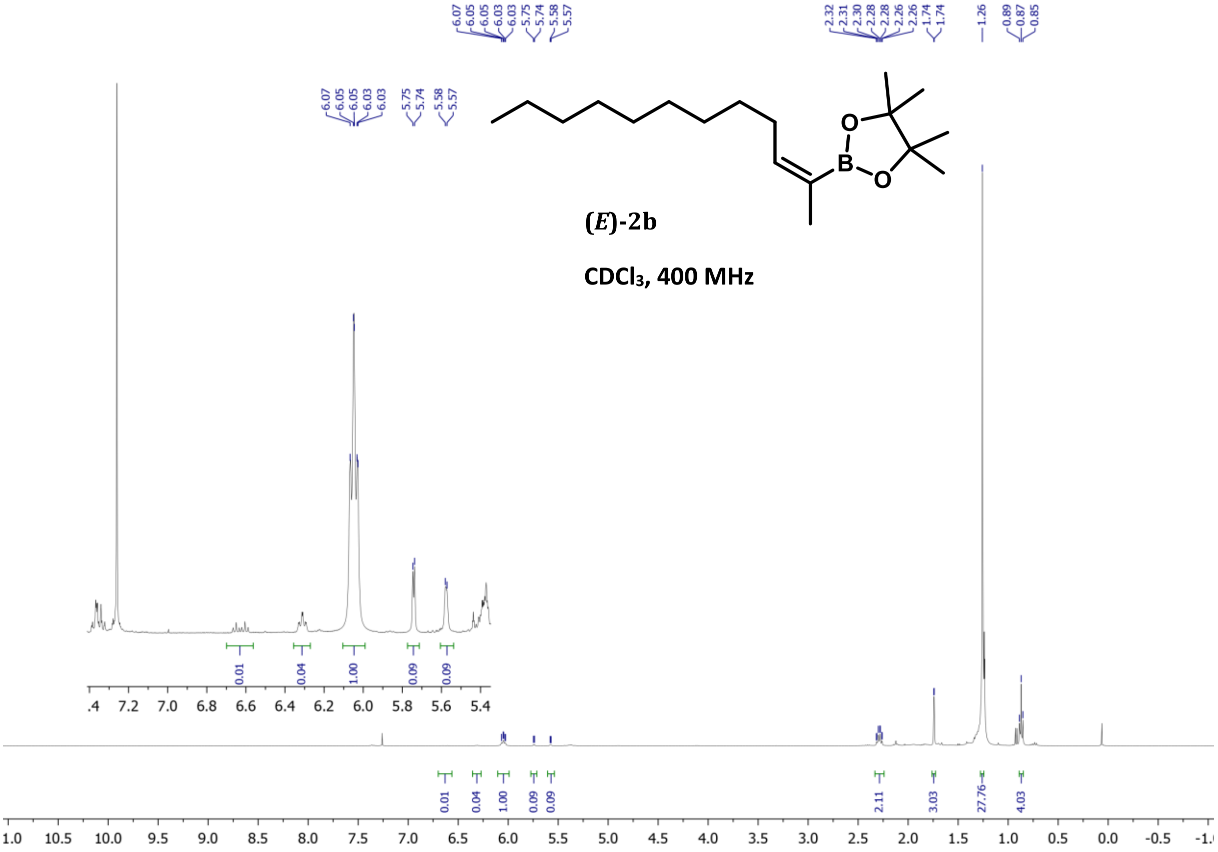
(*E*)-2b
CDCl₃, 400 MHz
6.07 6.05 6.05 6.03 6.03 6.03 5.75 5.74 5.58 5.57
2.32 2.31 2.30 2.28 2.28 2.26 2.26 1.74 1.74 1.26 0.89 0.87 0.85
0.01 0.04 1.00 0.09 0.09
2.11 3.03 27.76 4.03

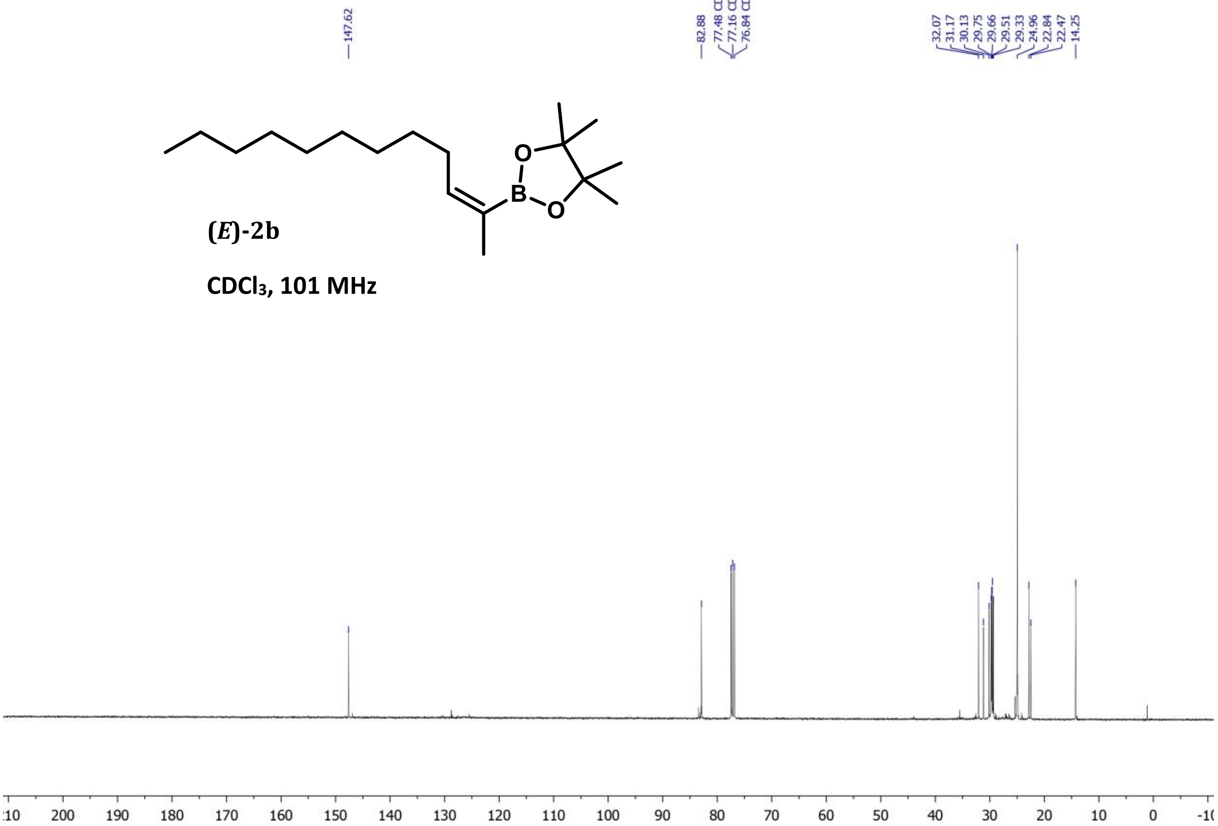
(*E*)-2b
CDCl₃, 101 MHz
147.62
82.88 77.48 CDCl3 77.16 CDCl3 76.84 CDCl3
32.07 31.17 30.13 29.75 29.66 29.51 29.33 24.96 22.84 22.47 14.25

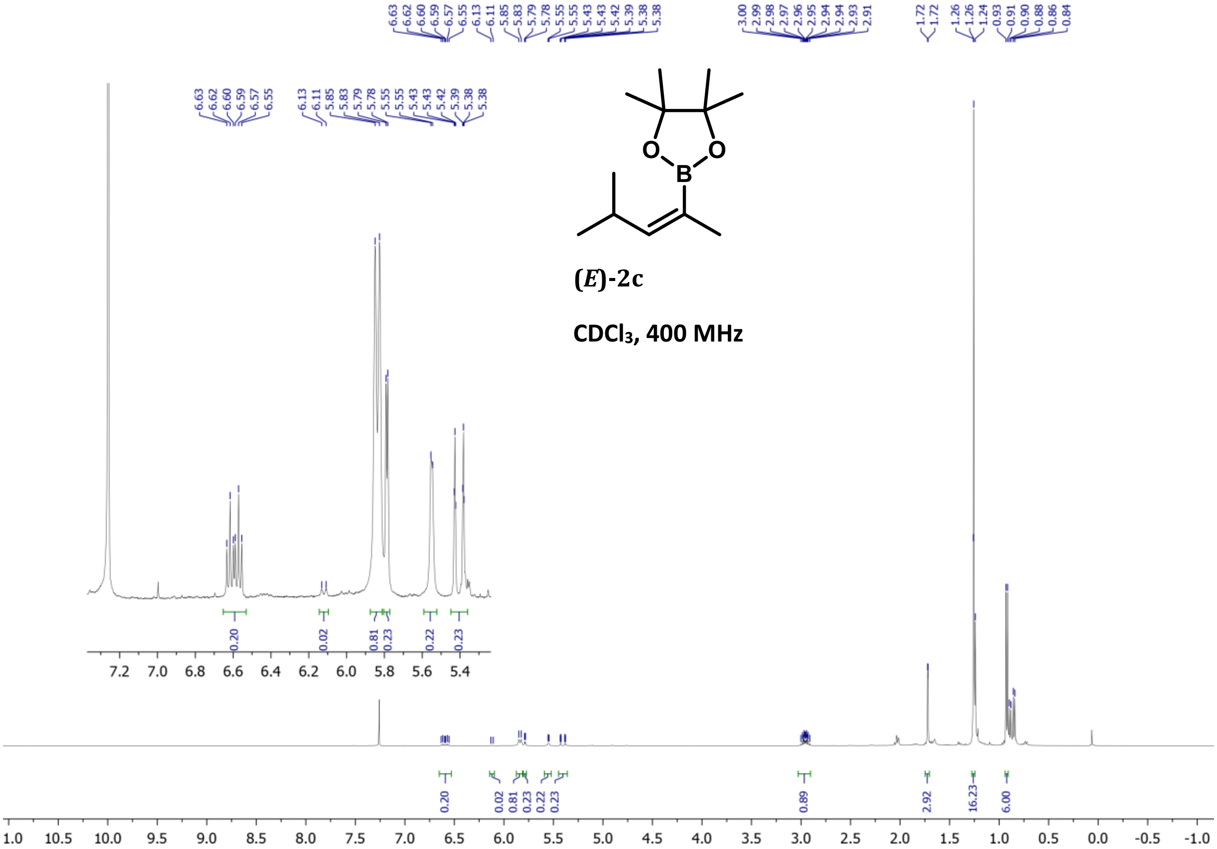
(E)-2c
CDCl3, 400 MHz

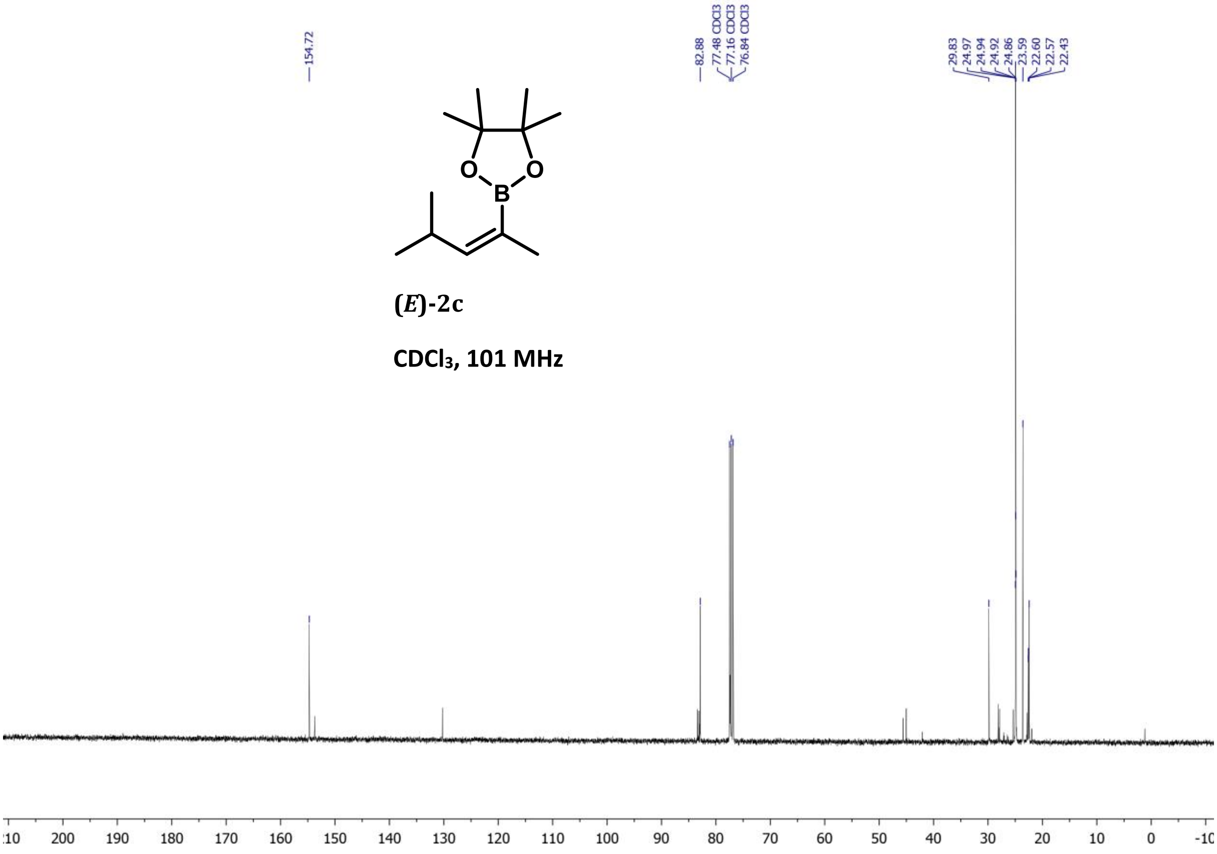
(E)-2c
CDCl3, 101 MHz

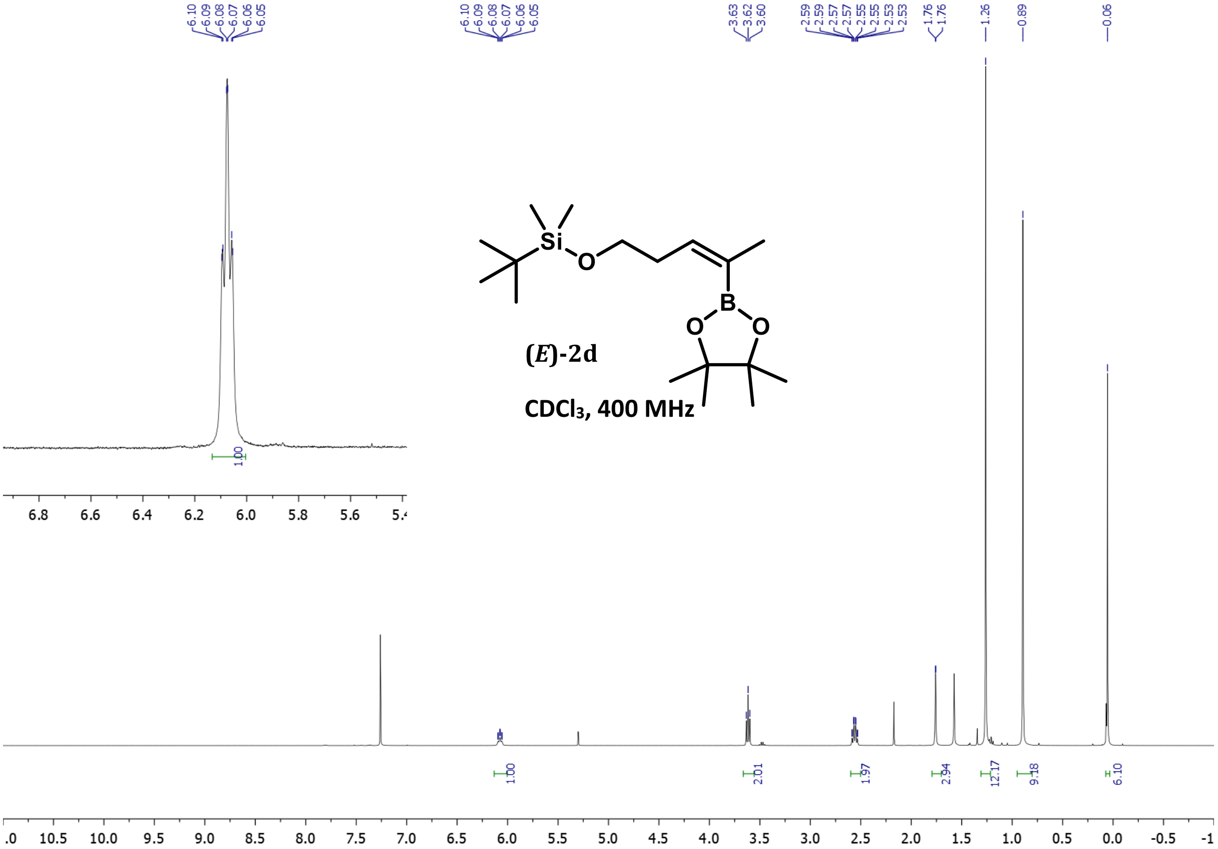
(E)-2d
CDCl3, 400 MHz

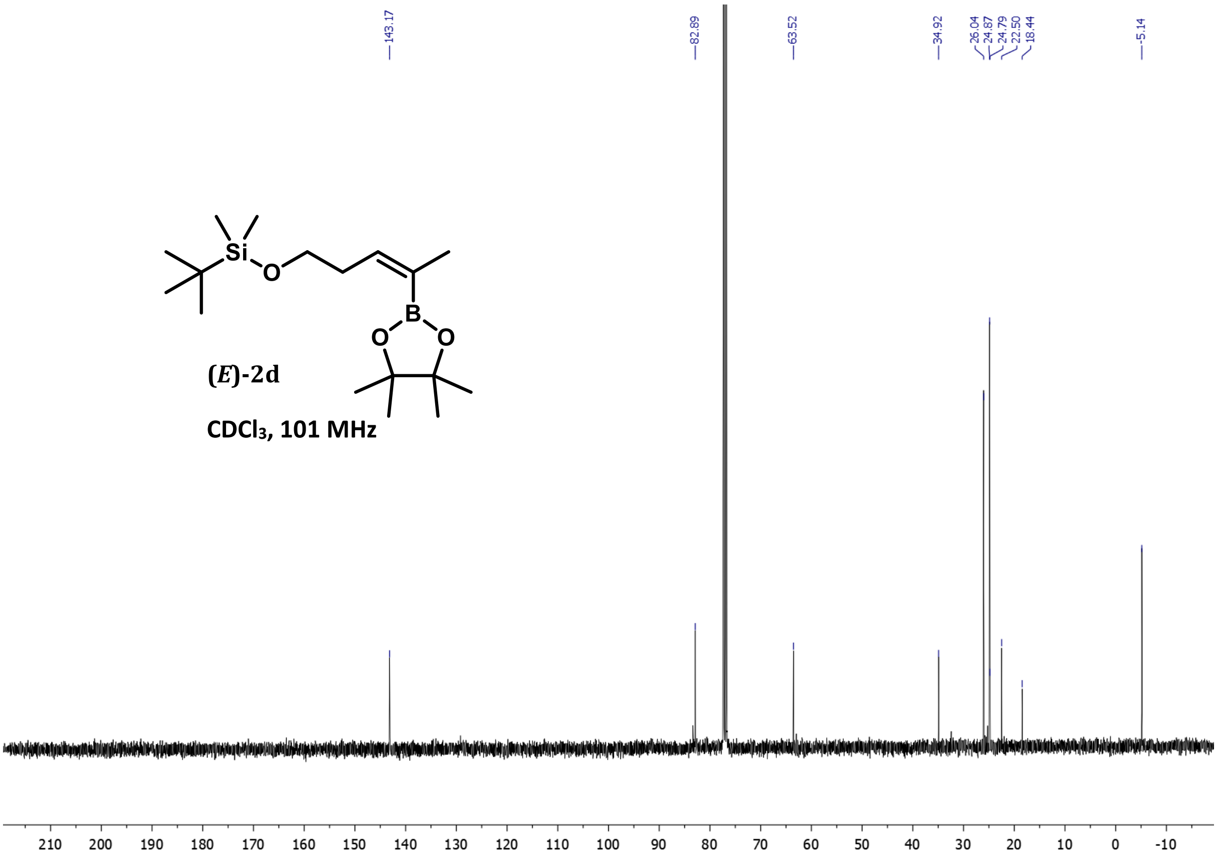
(E)-2d
CDCl3, 101 MHz

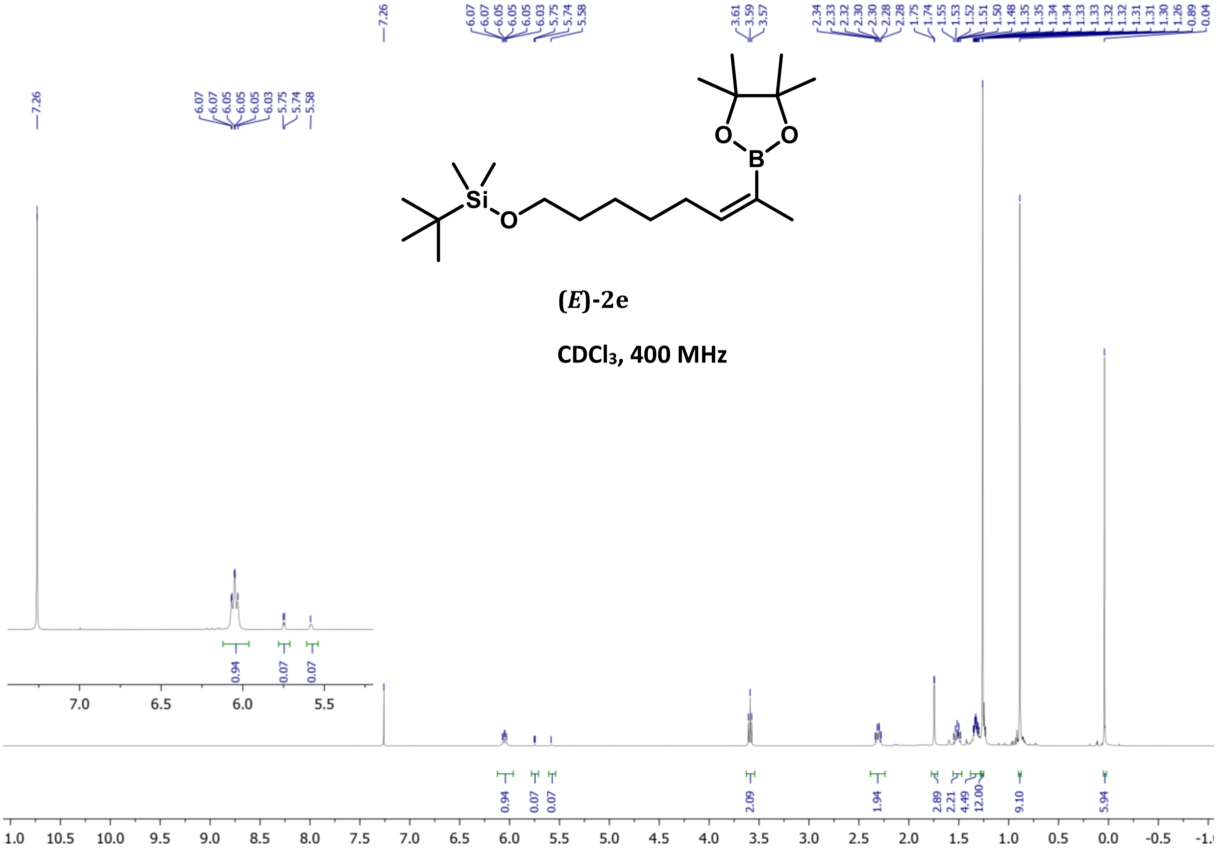
(E)-2e
CDCl3, 400 MHz

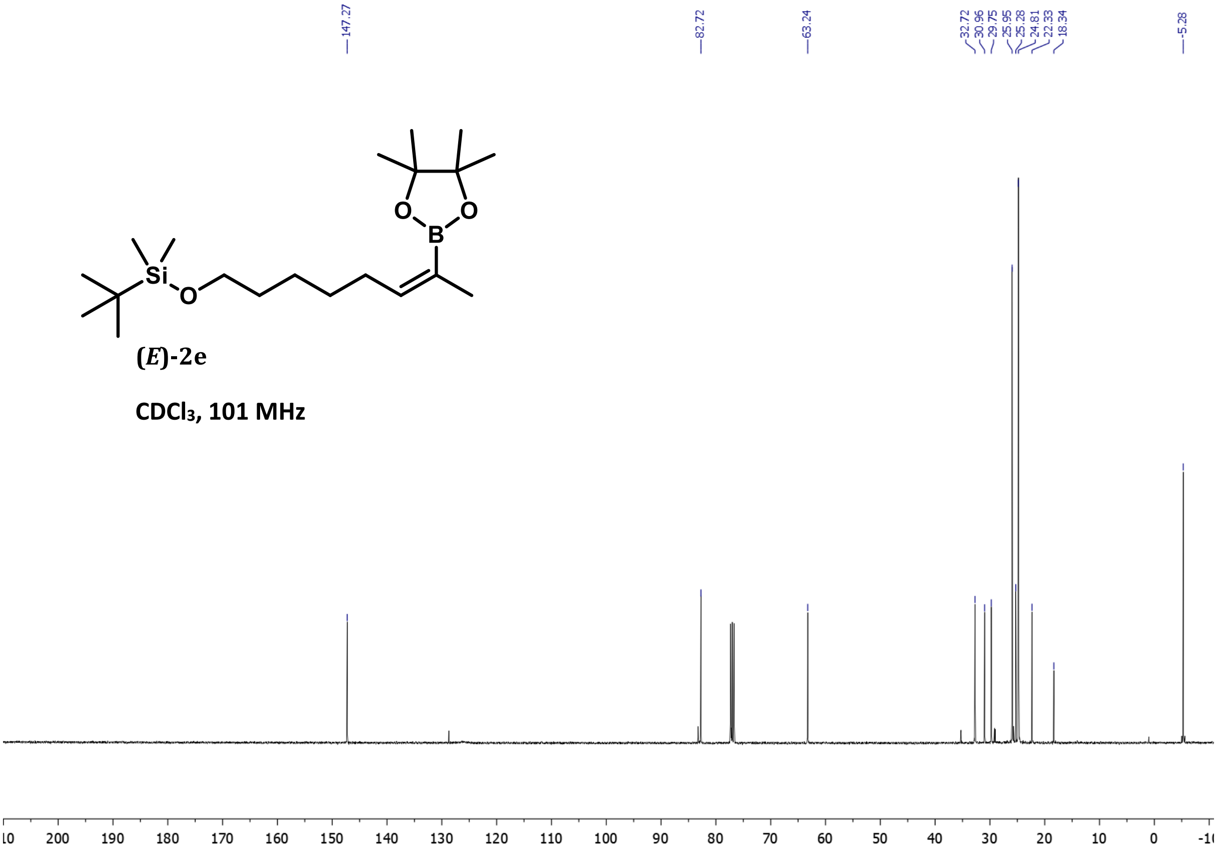
147.27
82.72
63.24
32.72
30.96
29.75
25.95
25.28
24.81
22.33
18.34
-5.28
(E)-2e
CDCl3, 101 MHz

(*E*)-2f

CDCl$_3$, 400 MHz

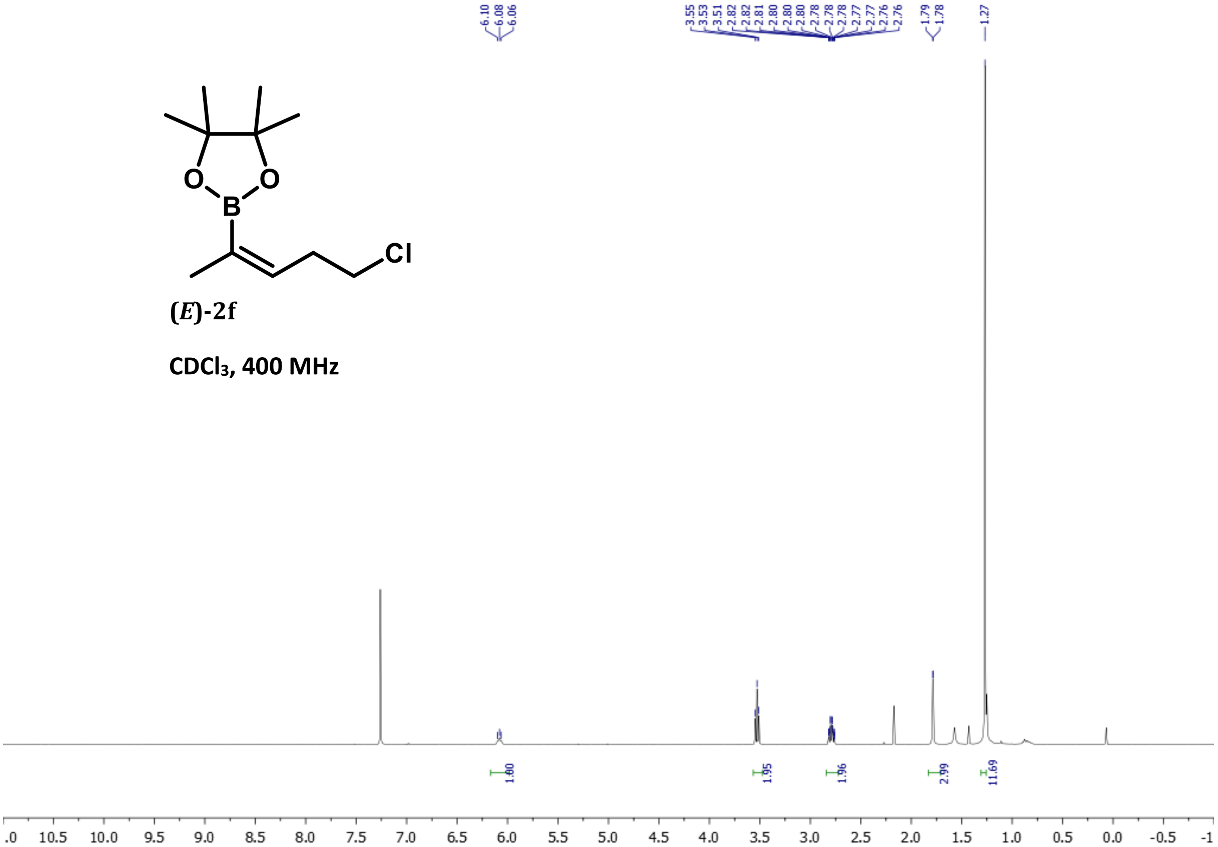

(*E*)-2f

CDCl$_3$, 101 MHz

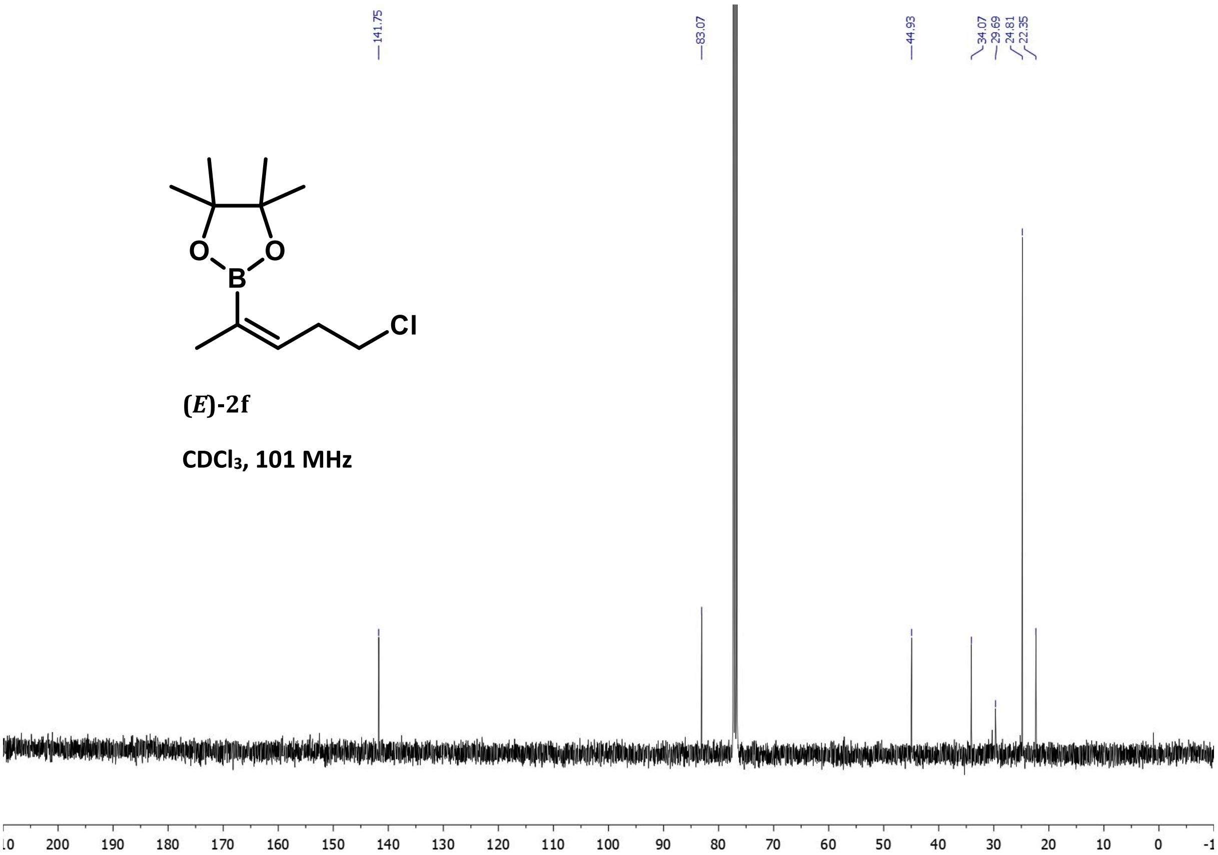

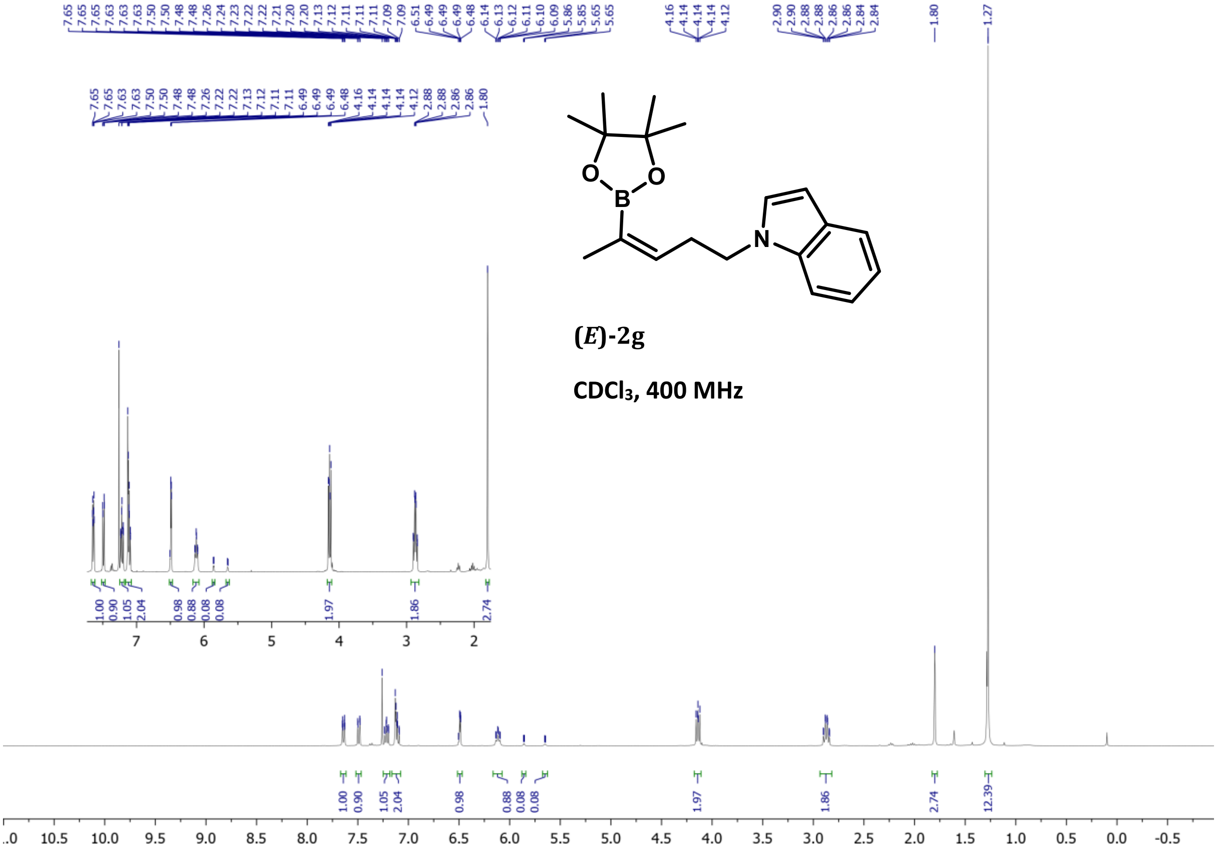
(E)-2g
CDCl3, 400 MHz
N
O
B
O

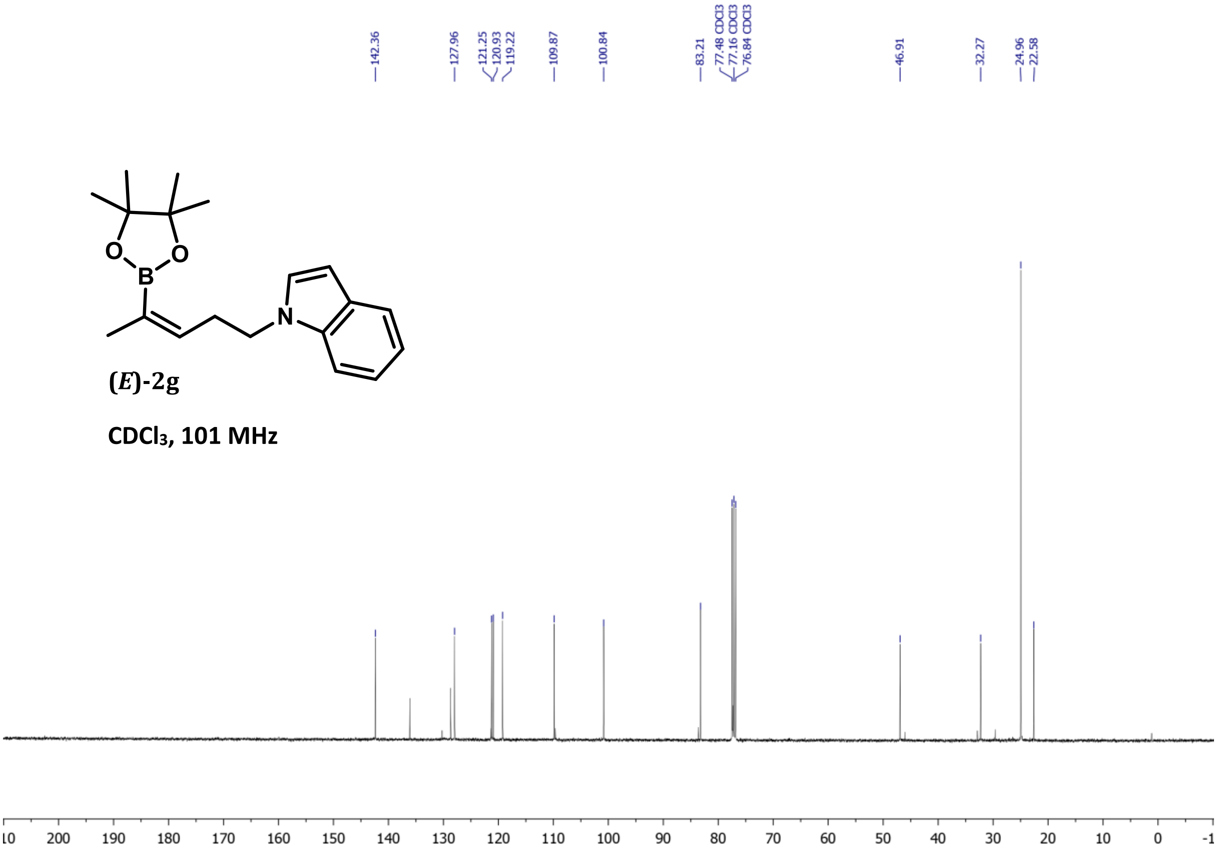
142.36
127.96
121.25
120.93
119.22
109.87
100.84
83.21
77.48 CDCl3
77.16 CDCl3
76.84 CDCl3
46.91
32.27
24.96
22.58
(E)-2g
CDCl3, 101 MHz
N
O
B
O

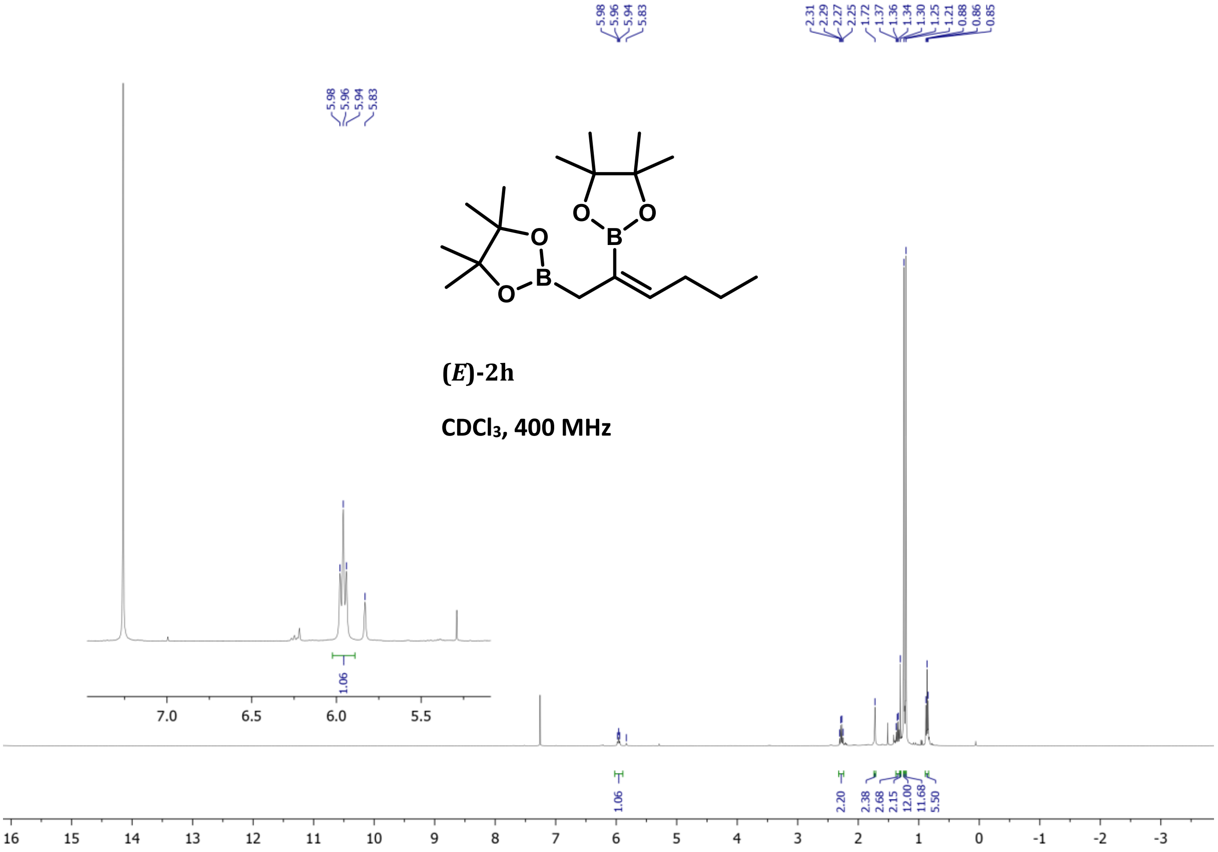
5.98
5.96
5.94
5.83
2.31
2.29
2.27
2.25
1.72
1.37
1.36
1.34
1.30
1.25
1.21
0.88
0.86
0.85
(*E*)-2h
CDCl₃, 400 MHz
1.06
2.20
2.38
2.68
2.15
12.00
11.68
5.50

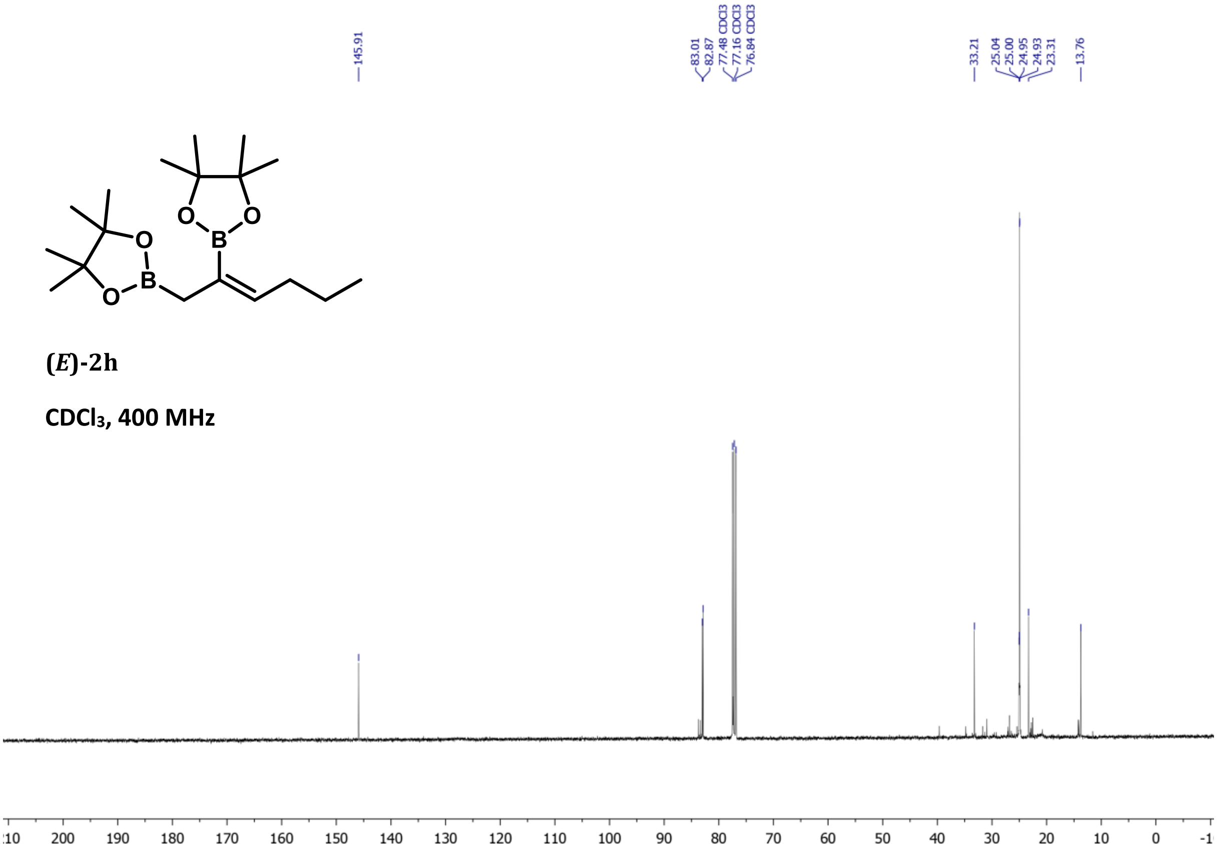
145.91
83.01
82.87
77.48 CDCl3
77.16 CDCl3
76.84 CDCl3
33.21
25.04
25.00
24.95
24.93
23.31
13.76
(*E*)-2h
CDCl₃, 400 MHz

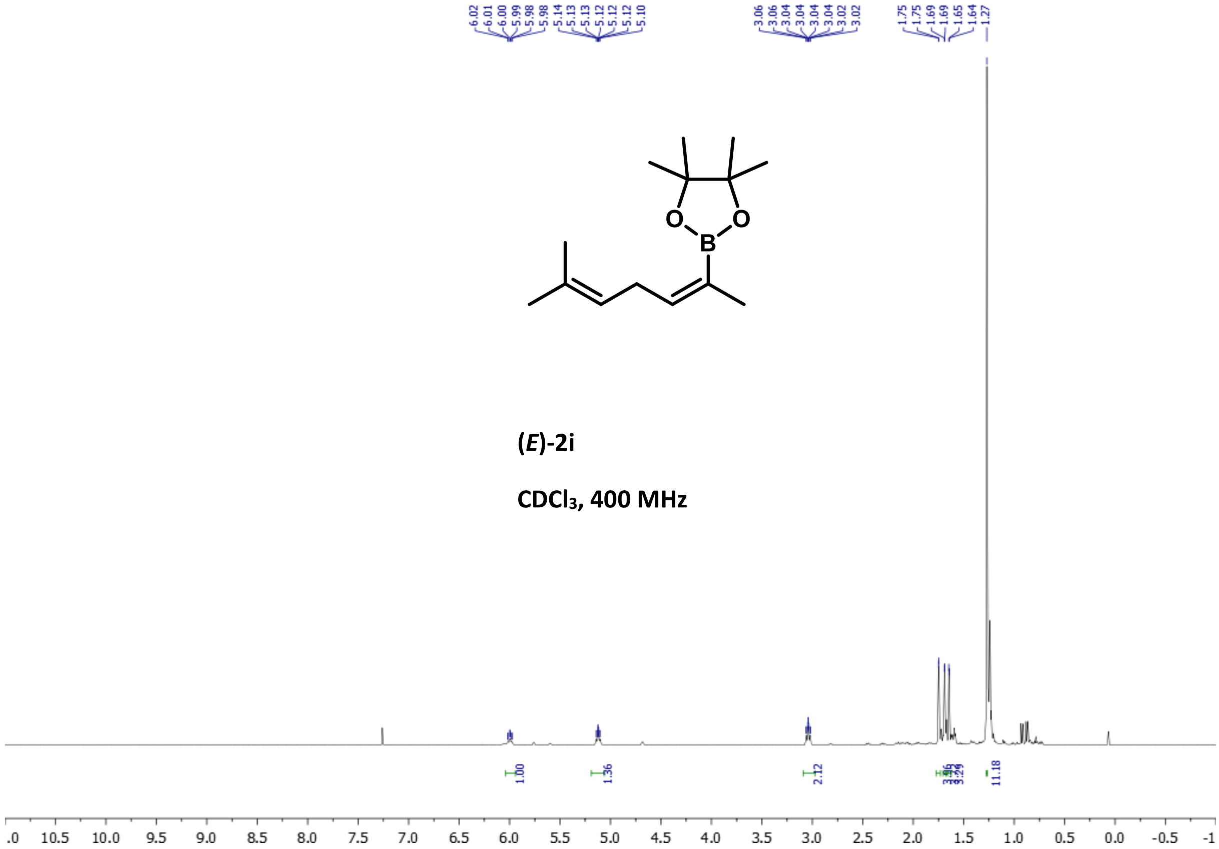
6.02 6.01 6.00 5.99 5.98 5.98 5.14 5.13 5.13 5.12 5.12 5.12 5.10
3.06 3.06 3.04 3.04 3.04 3.04 3.02 3.02
1.75 1.75 1.69 1.69 1.65 1.64 1.27
(E)-2i
CDCl3, 400 MHz
1.00 1.36 2.12 11.18

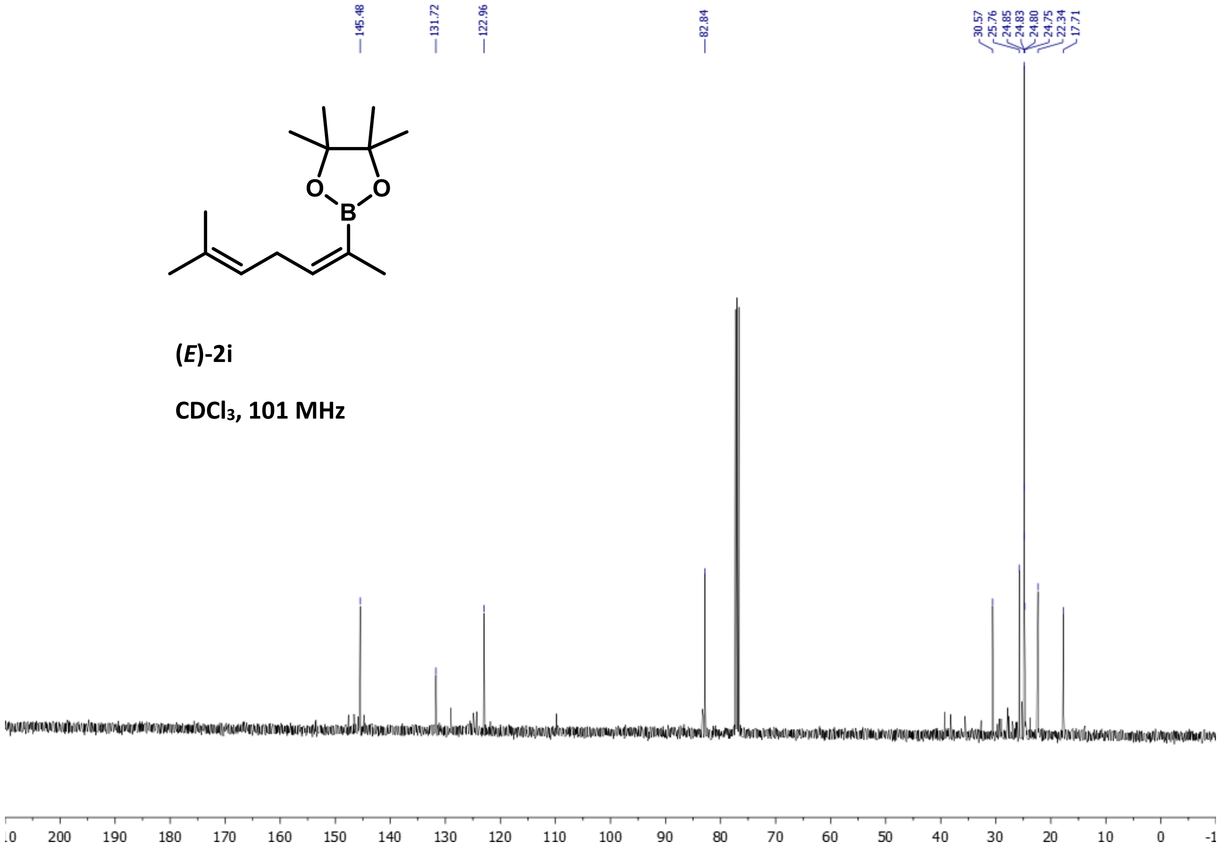
145.48 131.72 122.96 82.84
30.57 25.76 24.85 24.83 24.80 24.75 22.34 17.71
(E)-2i
CDCl3, 101 MHz

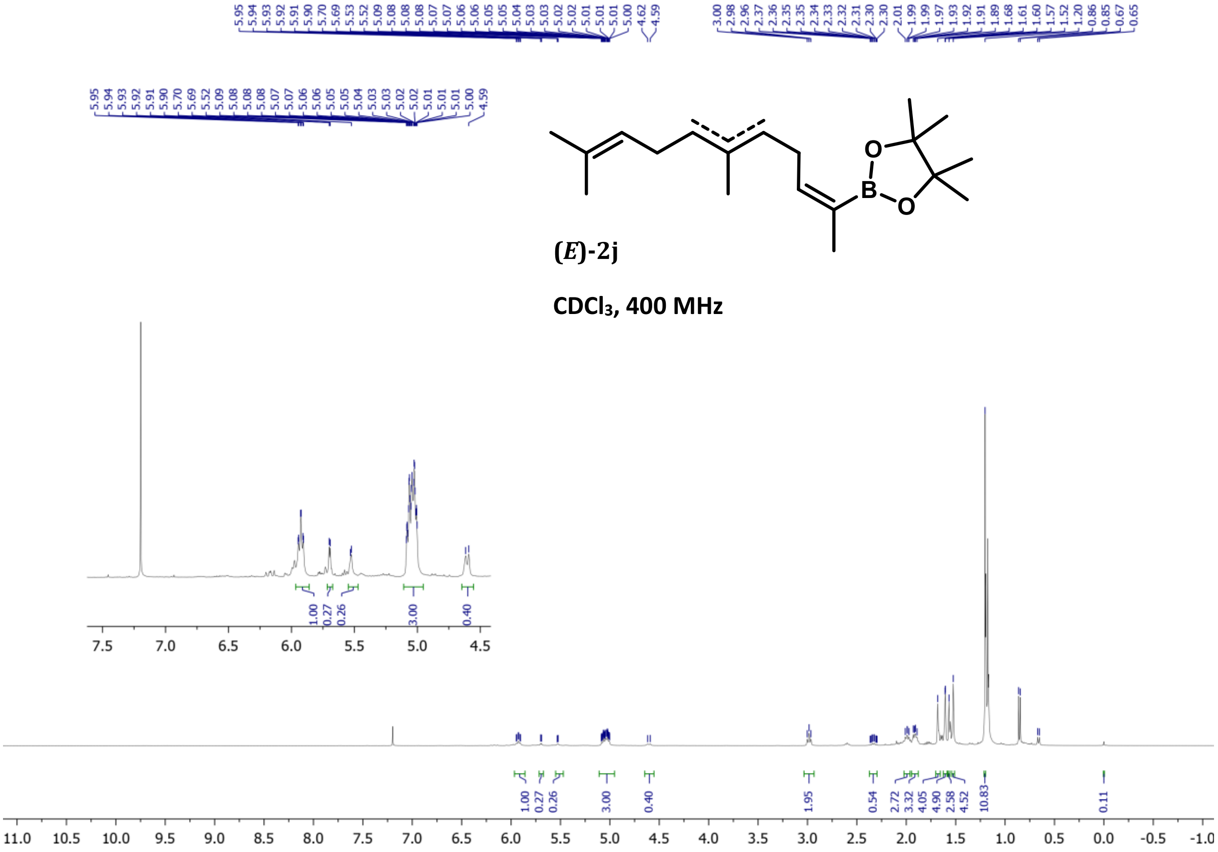

(*E*)-2j

$CDCl_3$, 400 MHz

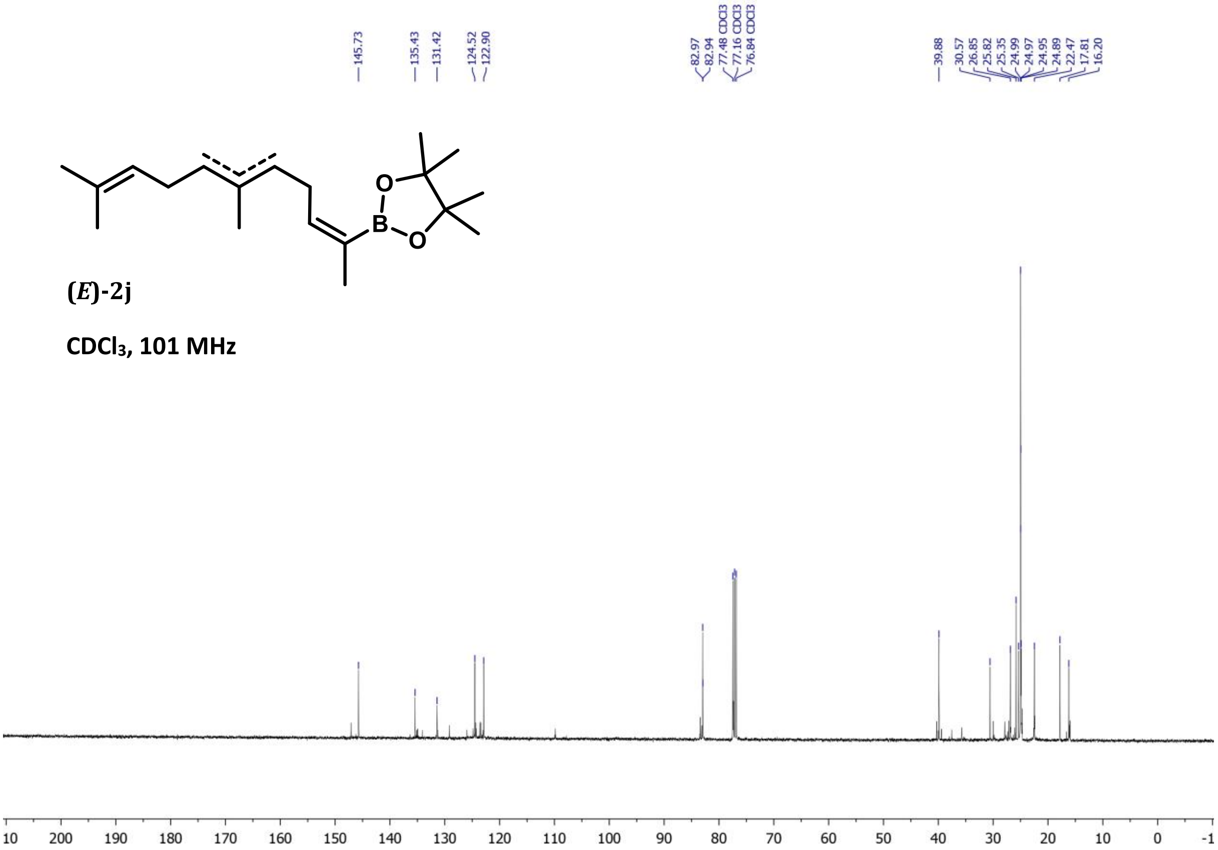

(*E*)-2j

$CDCl_3$, 101 MHz

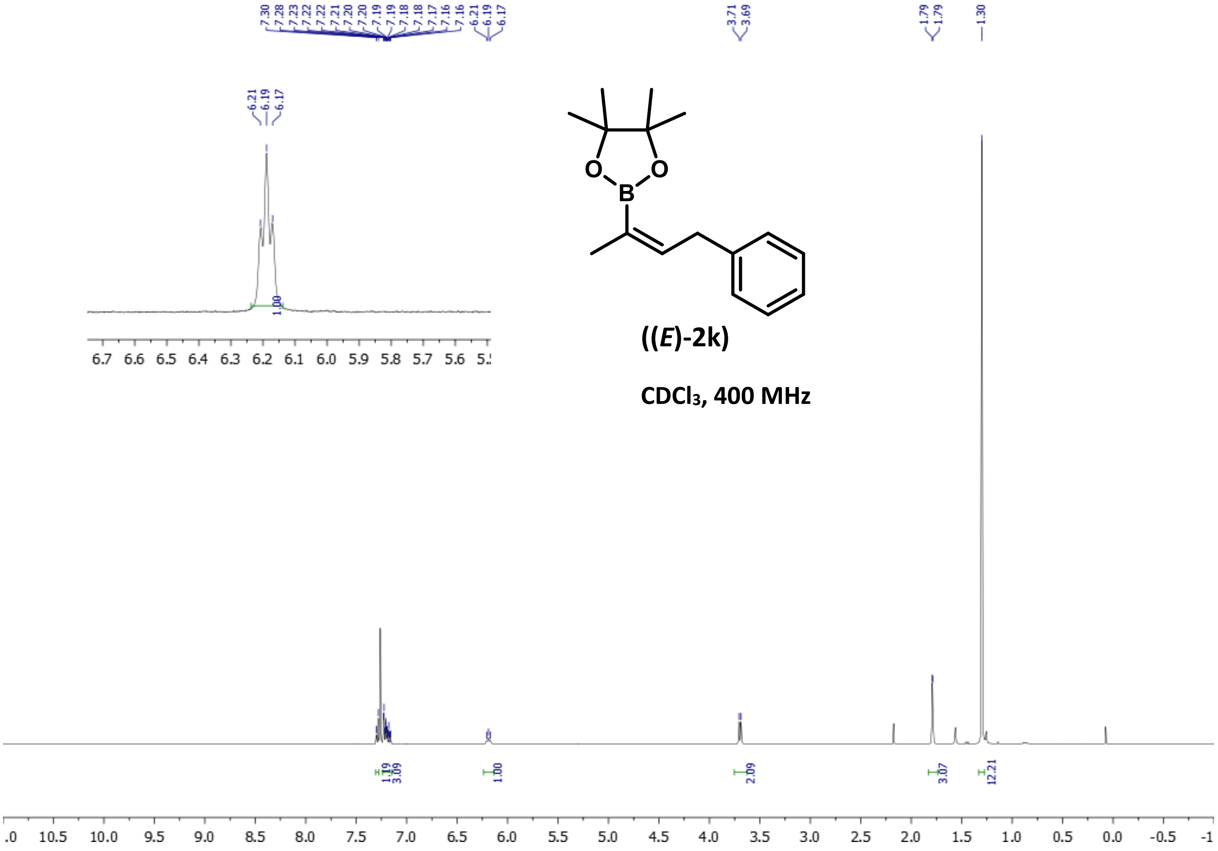
7.30 7.28 7.23 7.22 7.22 7.21 7.20 7.20 7.19 7.19 7.19 7.18 7.18 7.17 7.16 6.21 6.19 6.17
3.71 3.69
1.79 1.79
1.30
6.21 6.19 6.17
((E)-2k)
CDCl3, 400 MHz
1.19 3.09
1.00
2.09
3.07
12.21

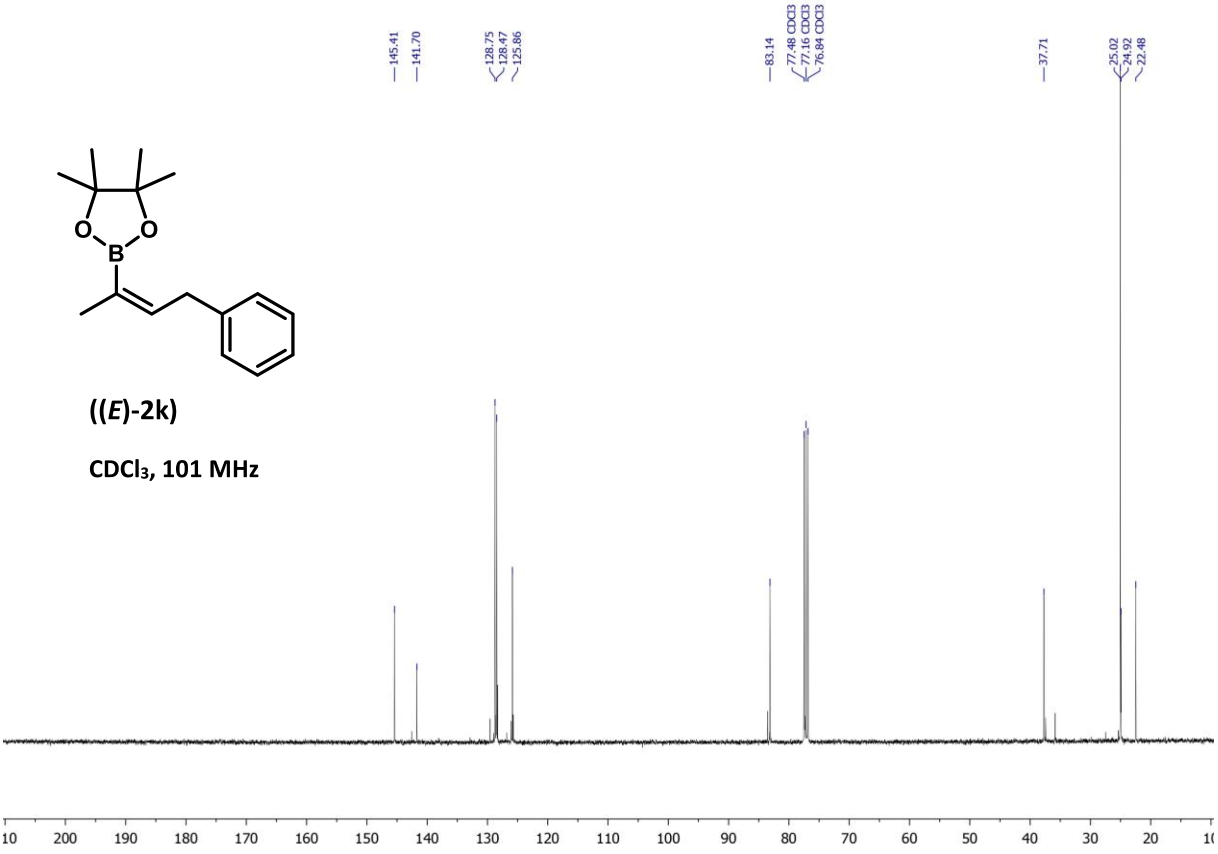
145.41
141.70
128.75 128.47 125.86
83.14
77.48 CDCl3 77.16 CDCl3 76.84 CDCl3
37.71
25.02 24.92 22.48
((E)-2k)
CDCl3, 101 MHz

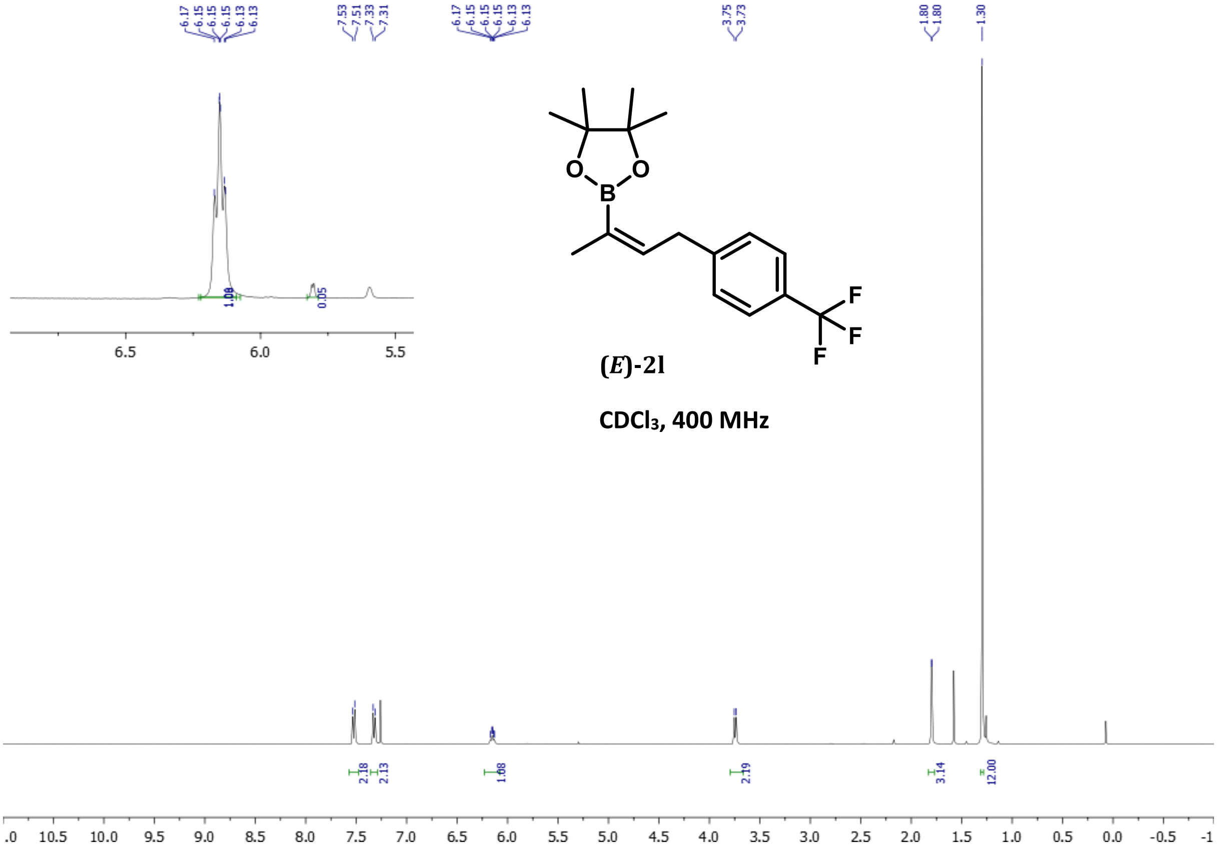
(E)-2l
CDCl₃, 400 MHz
F
F
F
O
B
O

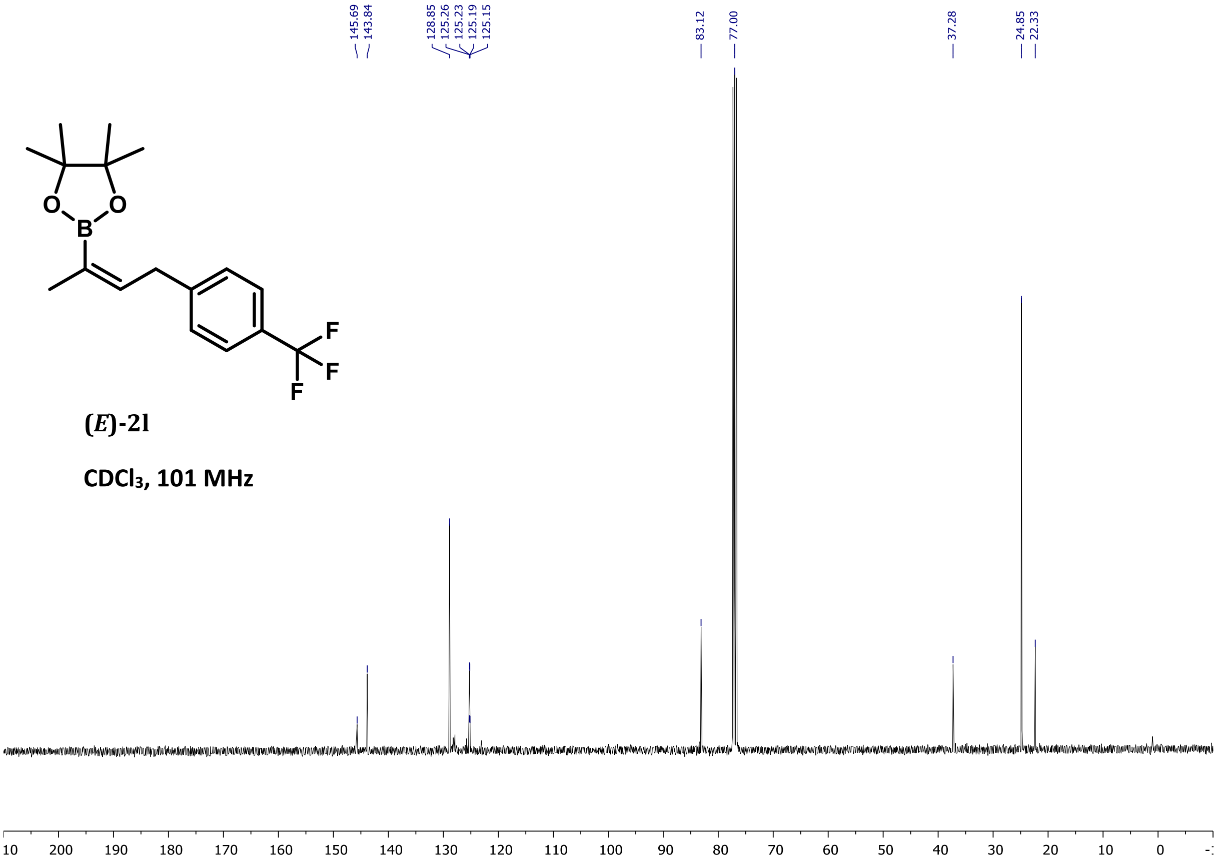
(E)-2l
CDCl₃, 101 MHz
F
F
F
O
B
O

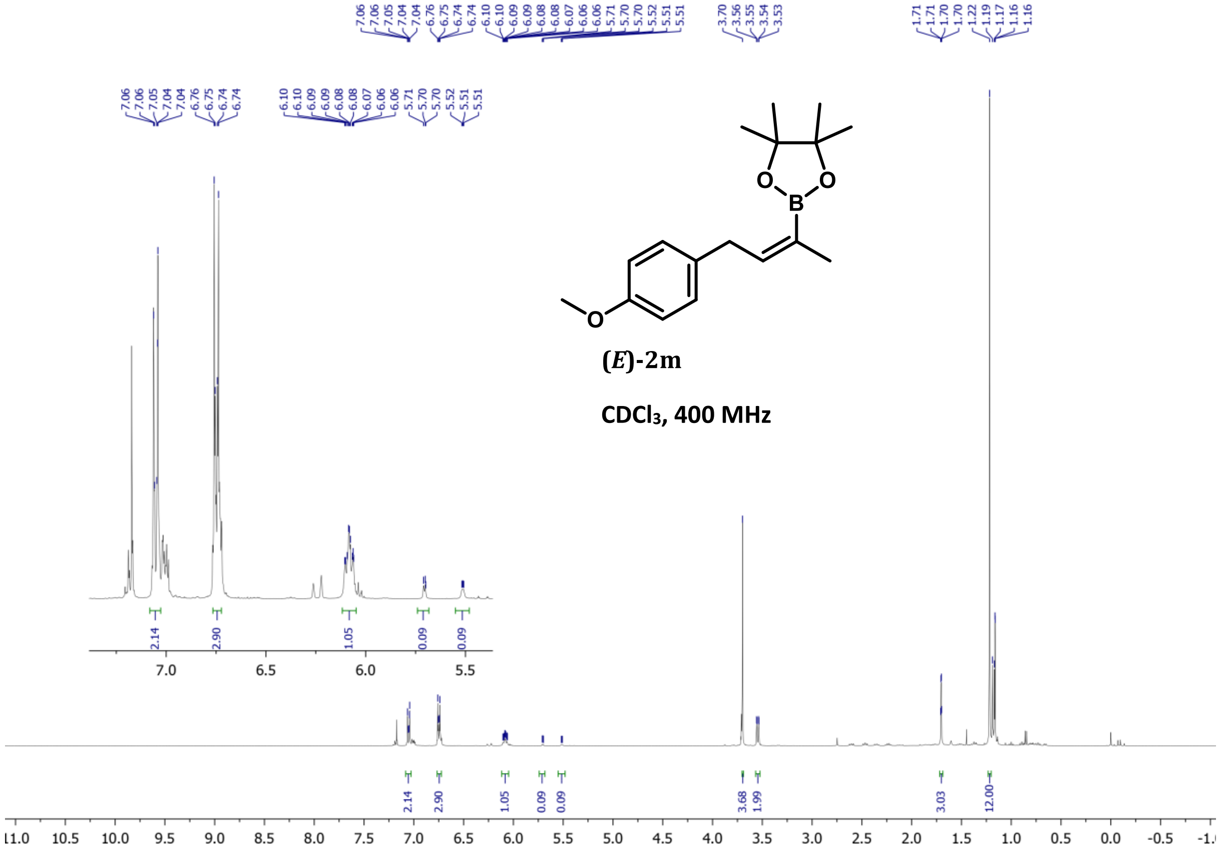
(*E*)-2m
CDCl₃, 400 MHz

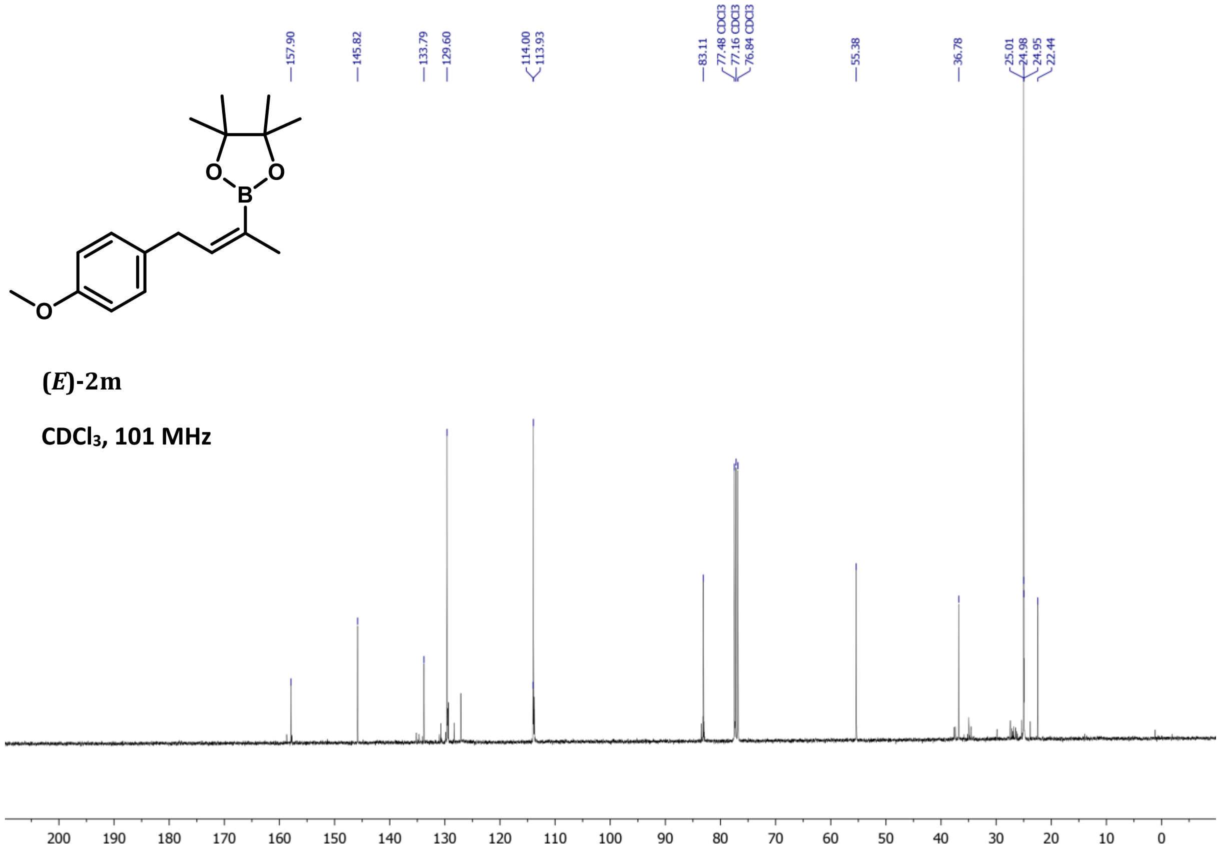
(*E*)-2m
CDCl₃, 101 MHz

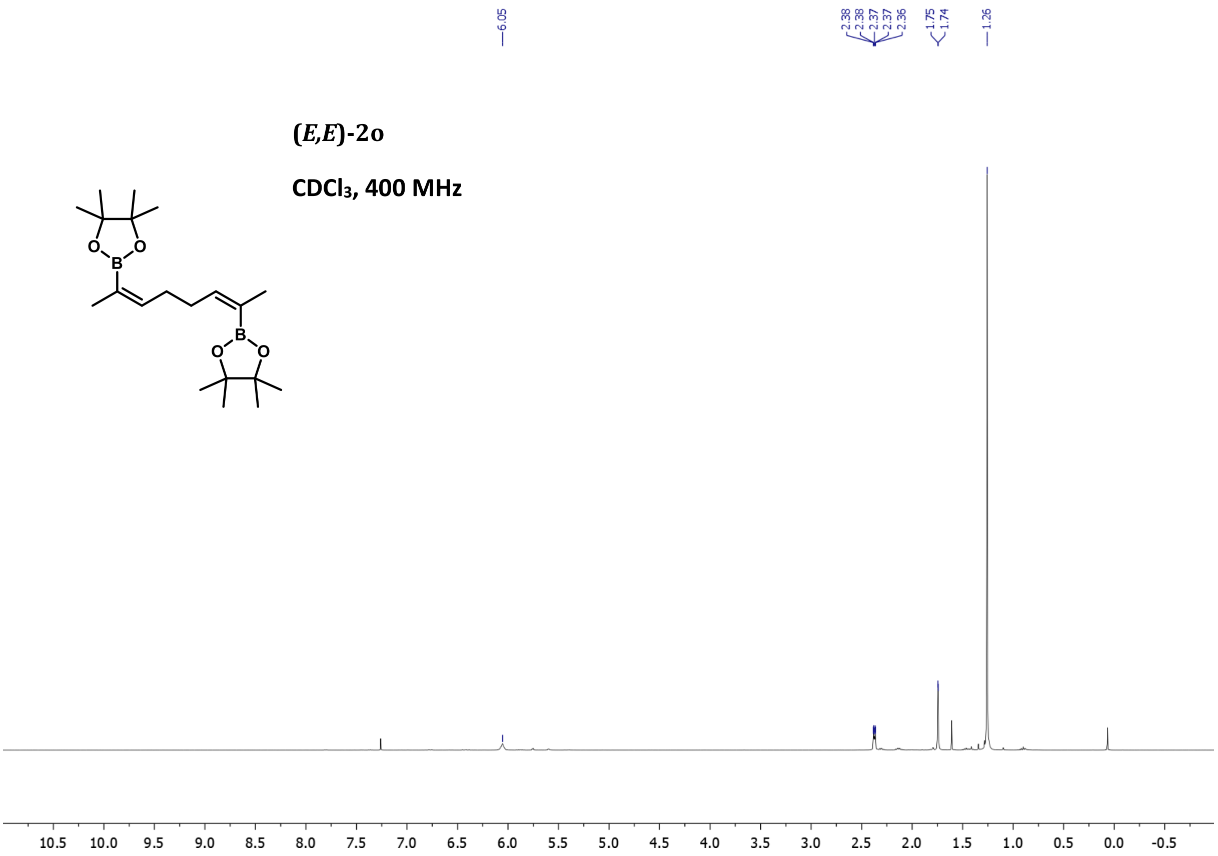
6.05
2.38
2.38
2.37
2.37
2.36
1.75
1.74
1.26
(E,E)-2o
CDCl3, 400 MHz
10.5 10.0 9.5 9.0 8.5 8.0 7.5 7.0 6.5 6.0 5.5 5.0 4.5 4.0 3.5 3.0 2.5 2.0 1.5 1.0 0.5 0.0 -0.5

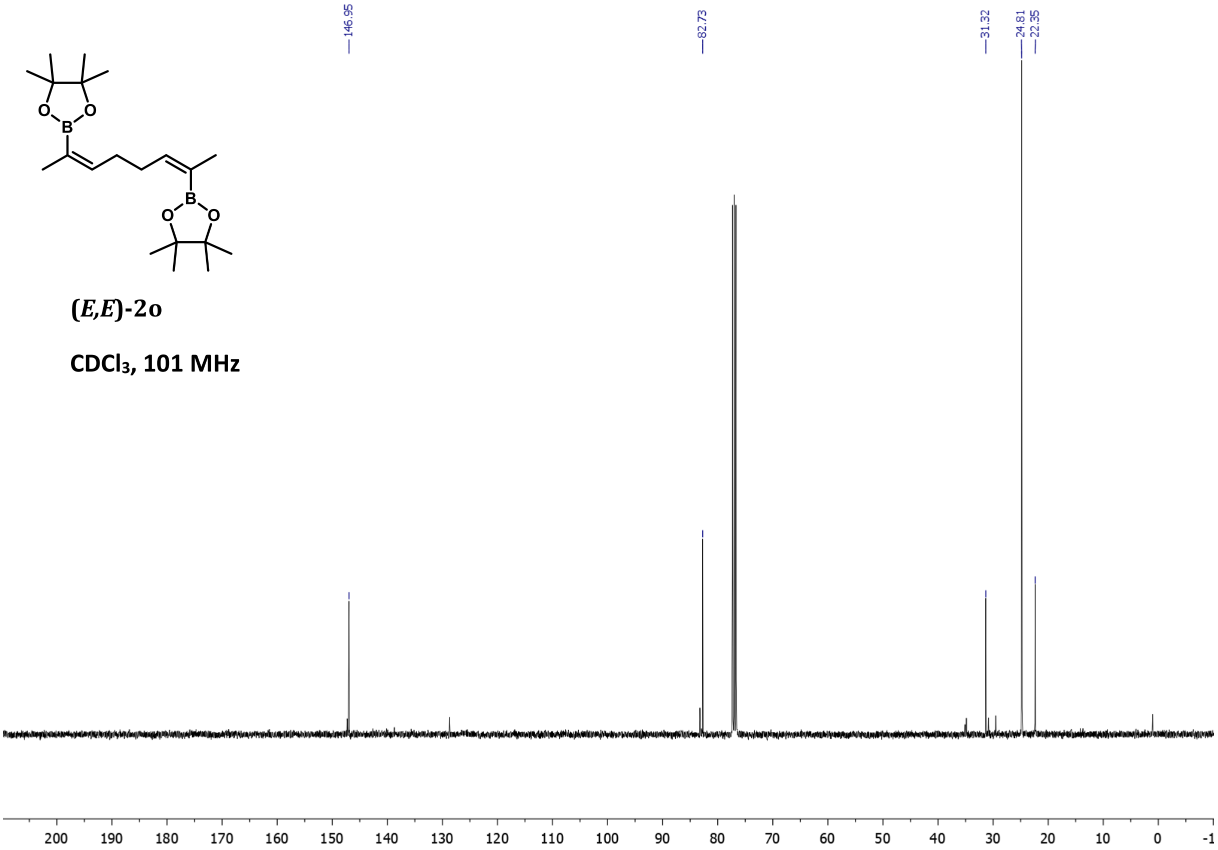
146.95
82.73
31.32
24.81
22.35
(E,E)-2o
CDCl3, 101 MHz
200 190 180 170 160 150 140 130 120 110 100 90 80 70 60 50 40 30 20 10 0 -1

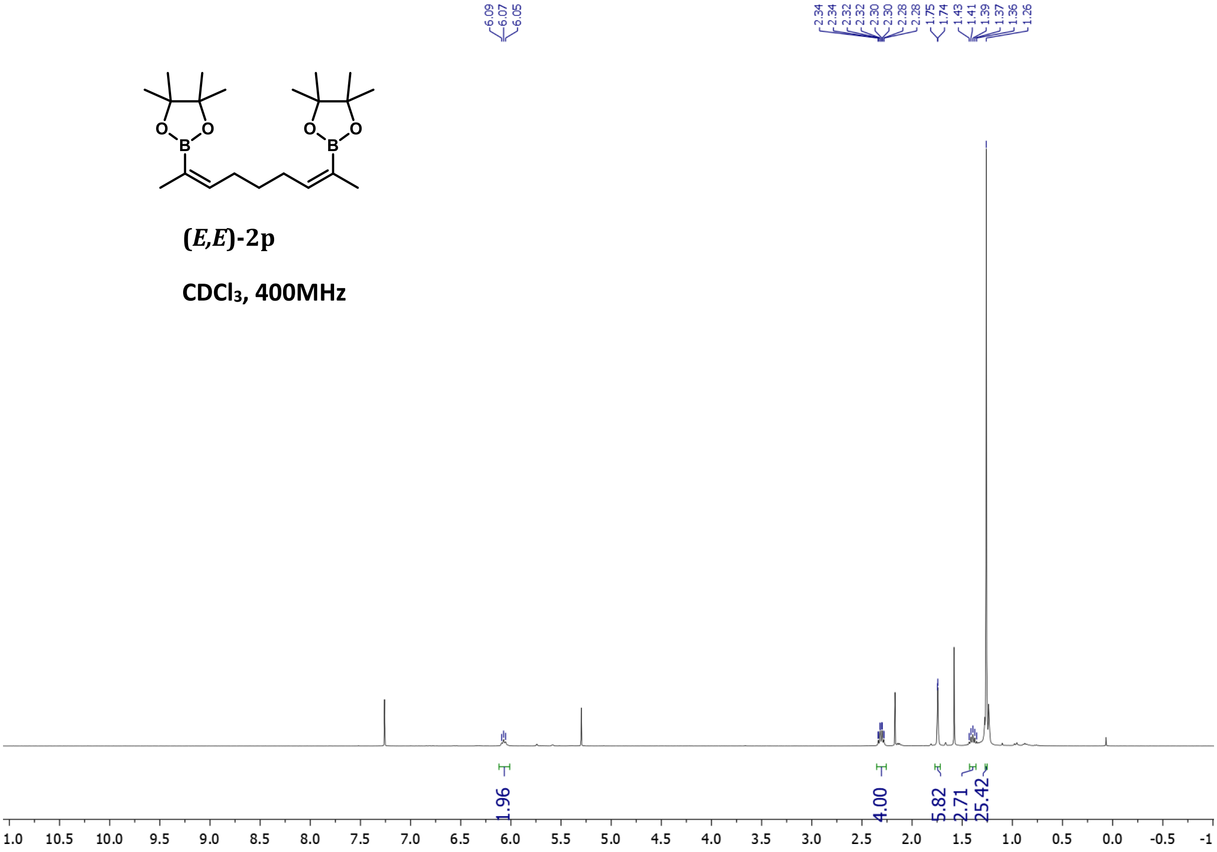

(*E,E*)-2p

$CDCl_3$, 400MHz

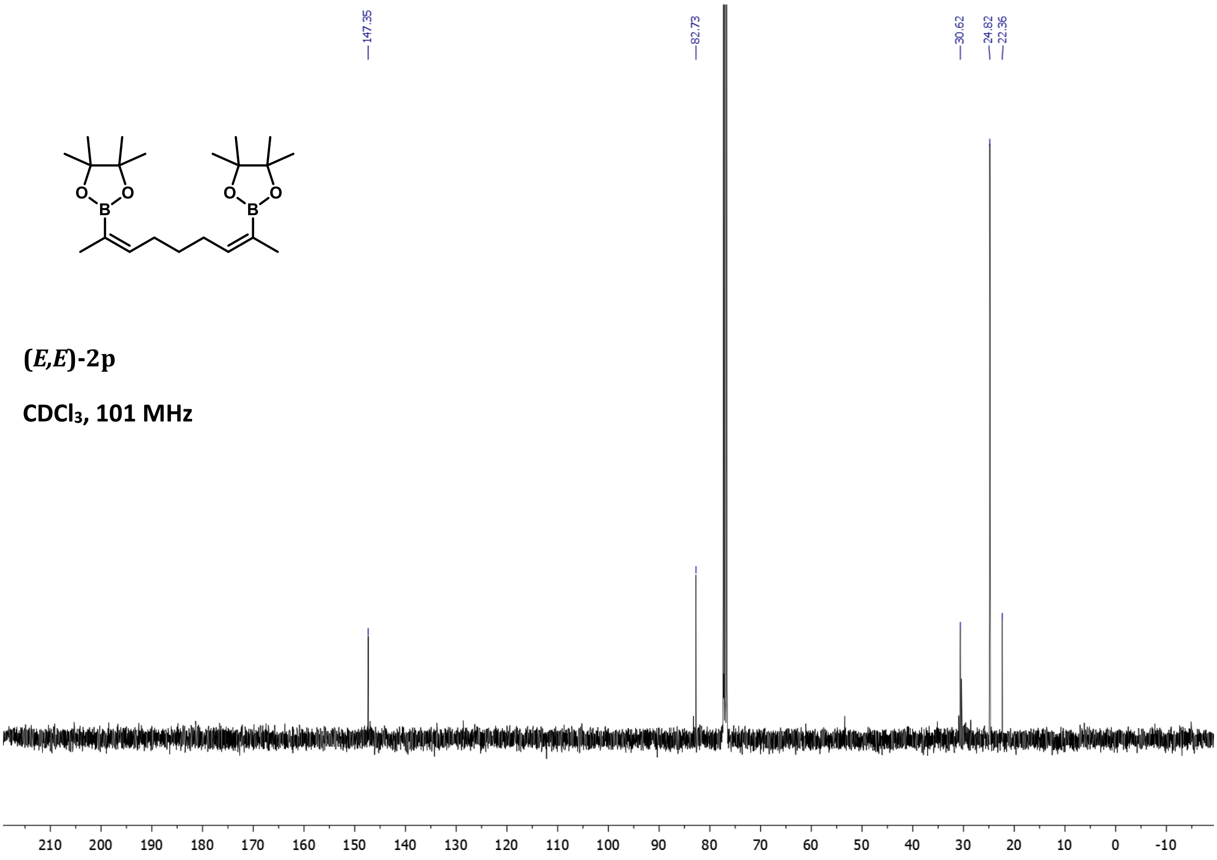

(*E,E*)-2p

$CDCl_3$, 101 MHz

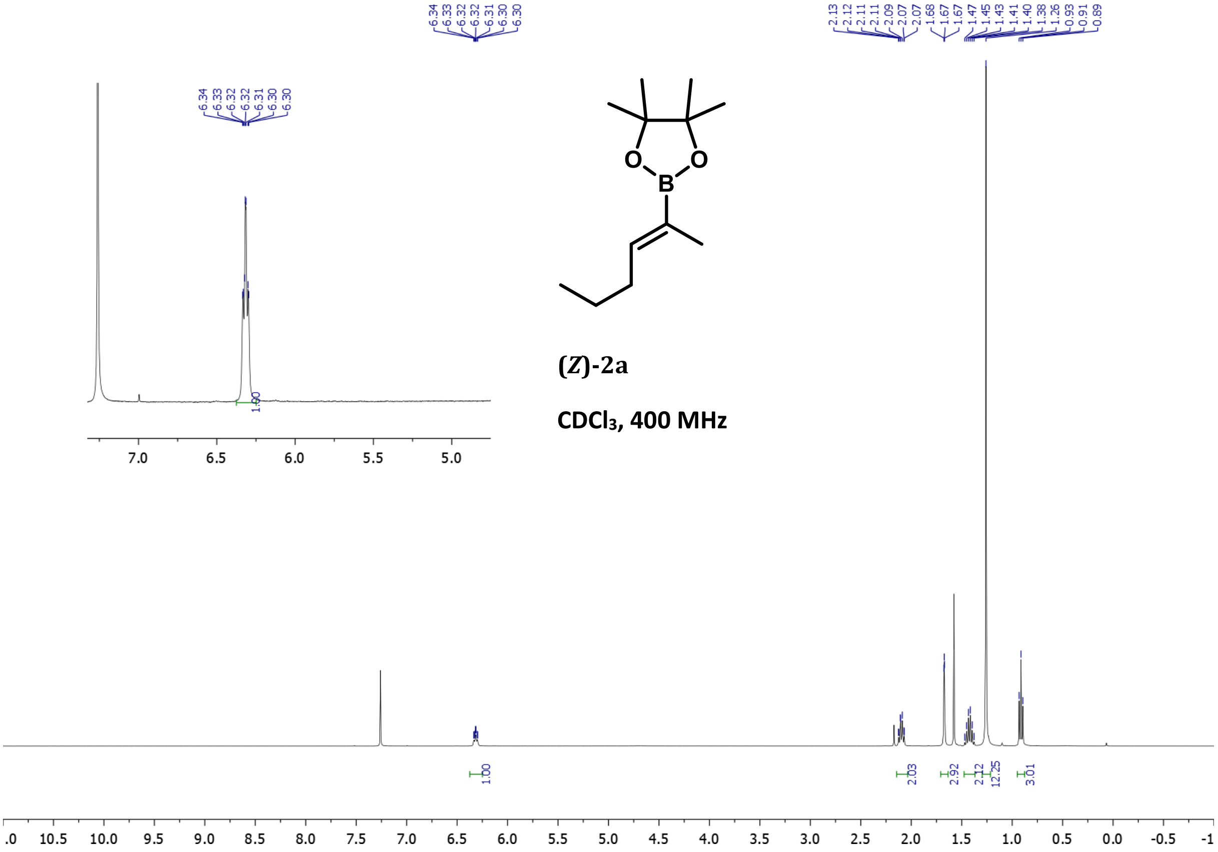
(Z)-2a
CDCl3, 400 MHz

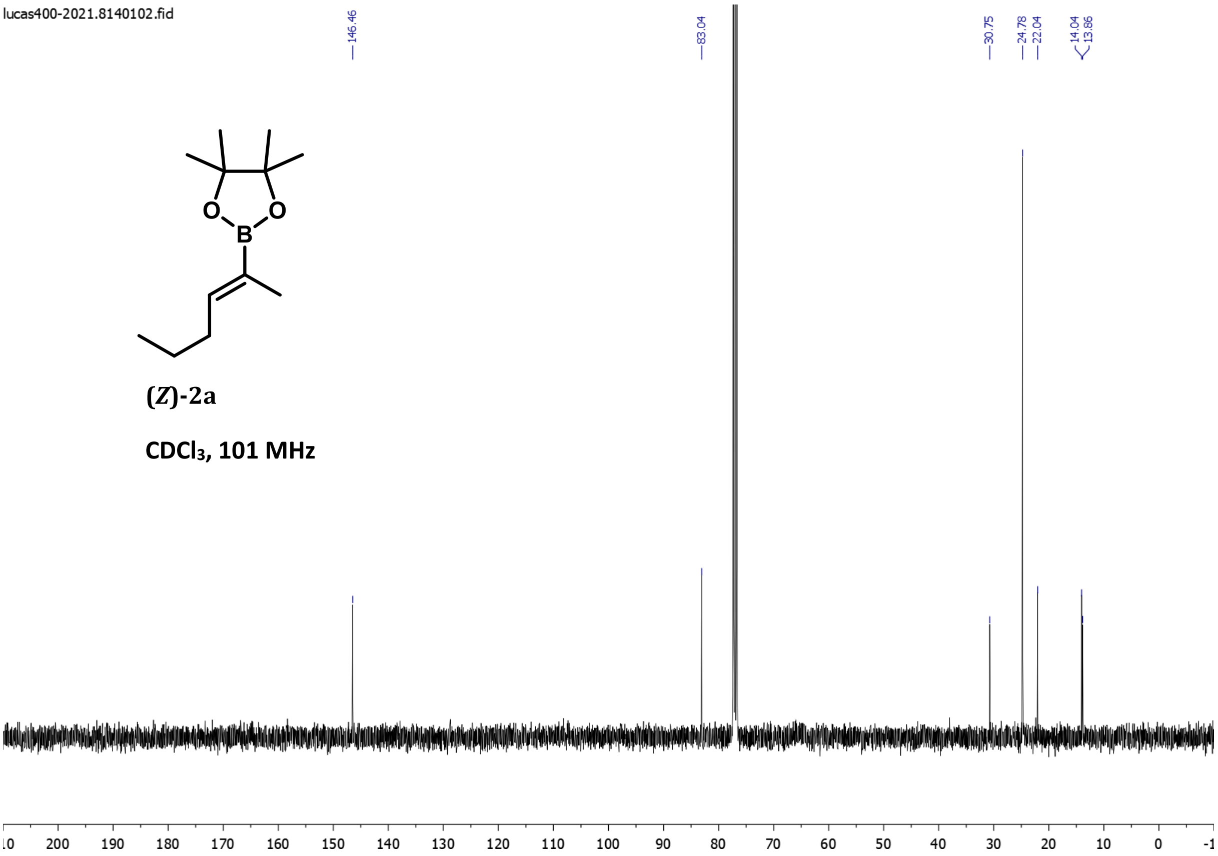
lucas400-2021.8140102.fid
(Z)-2a
CDCl3, 101 MHz

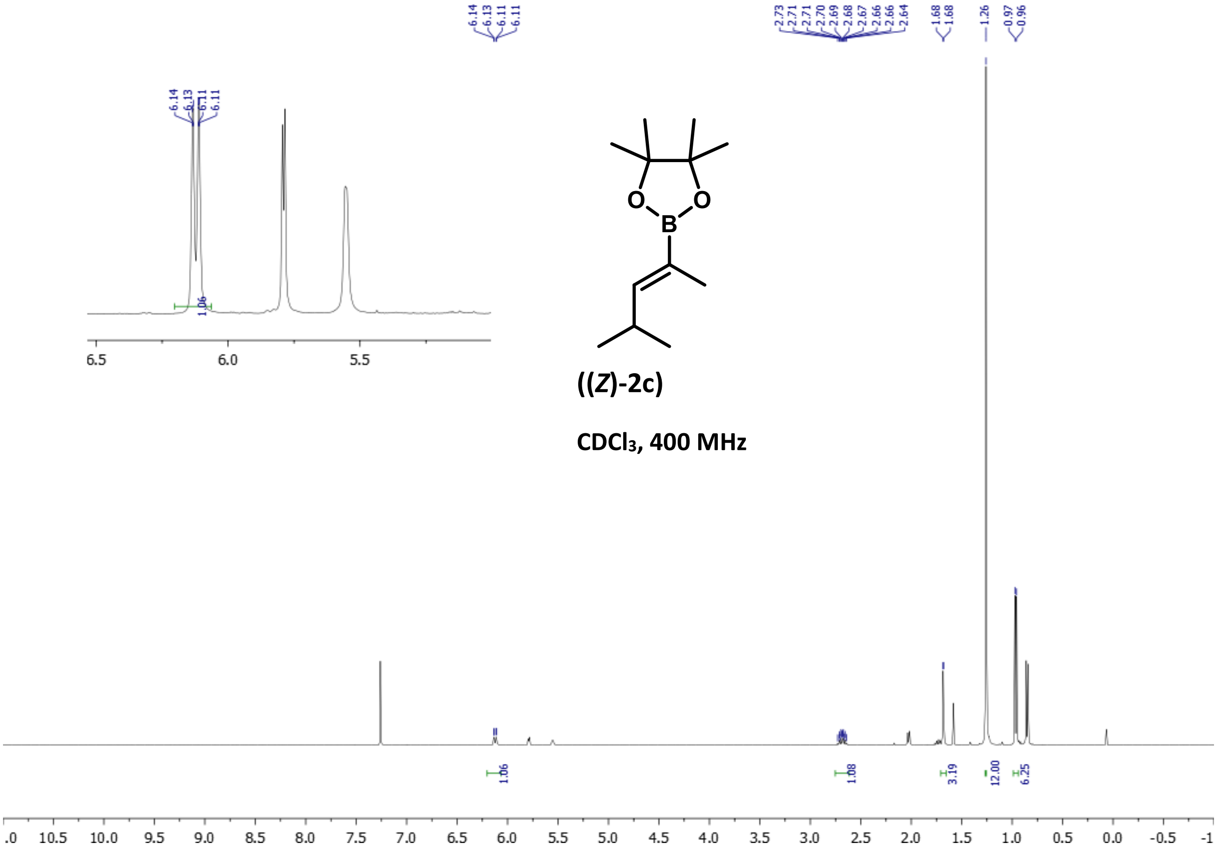
((Z)-2c)
CDCl₃, 400 MHz
6.14 6.13 6.11 6.11
2.73 2.71 2.71 2.70 2.69 2.68 2.67 2.66 2.66 2.64
1.68 1.68
1.26
0.97 0.96
1.06
1.08
3.19
12.00
6.25

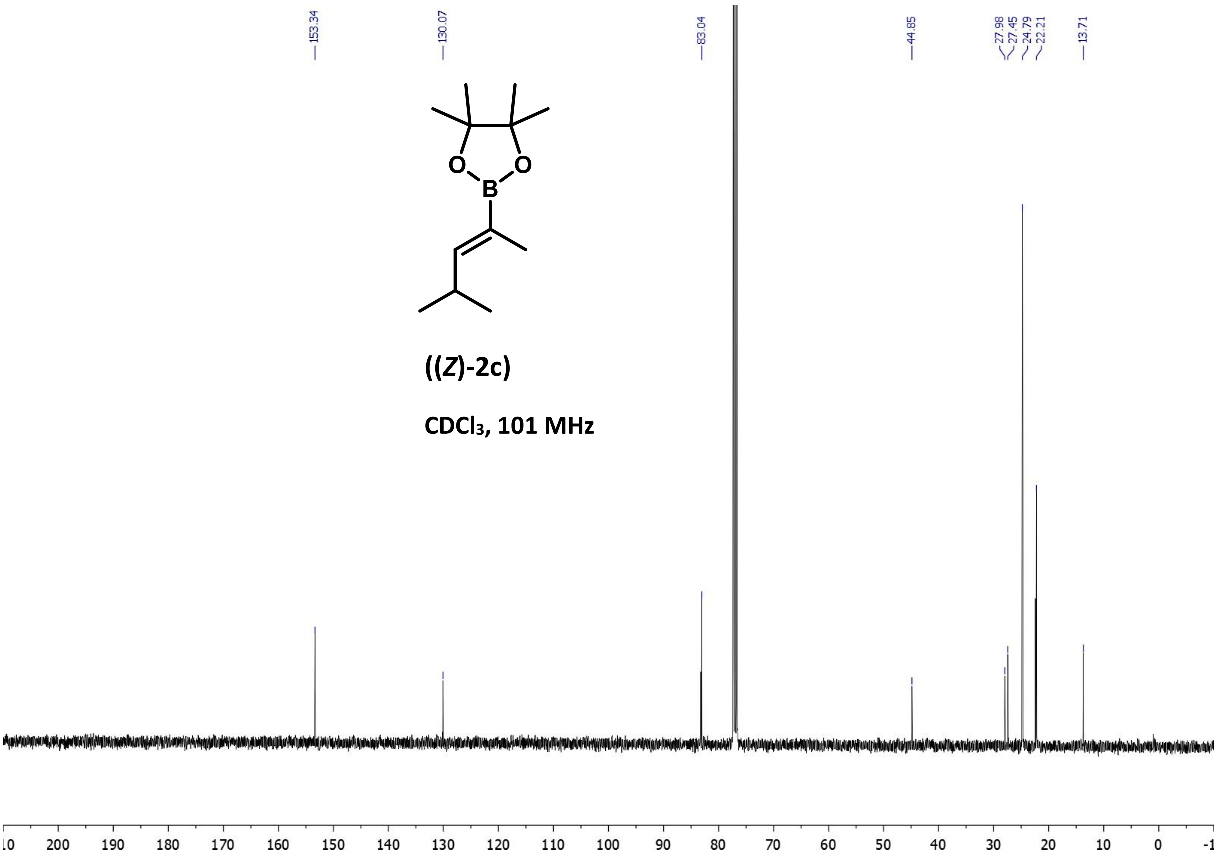
((Z)-2c)
CDCl₃, 101 MHz
153.34
130.07
83.04
44.85
27.98 27.45 24.79 22.21
13.71

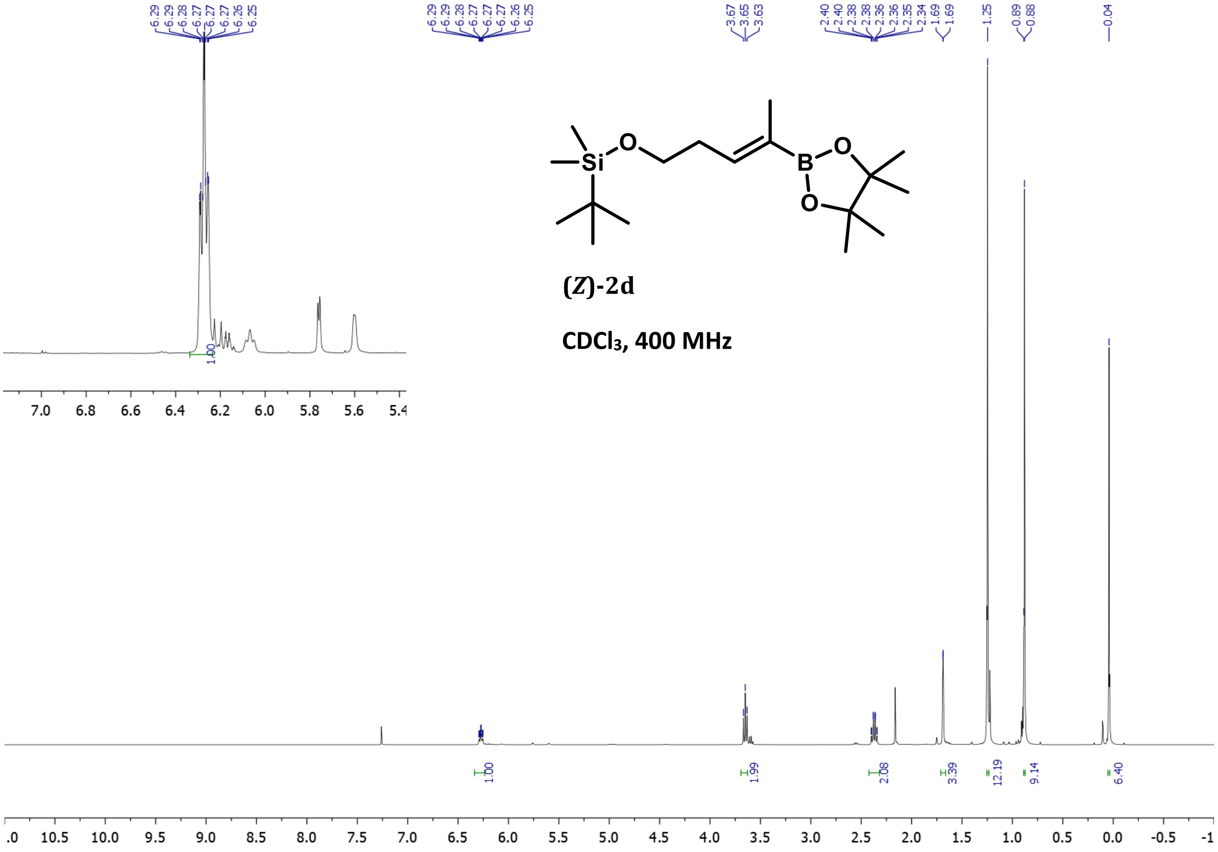

(Z)-2d

$CDCl_3$, 400 MHz

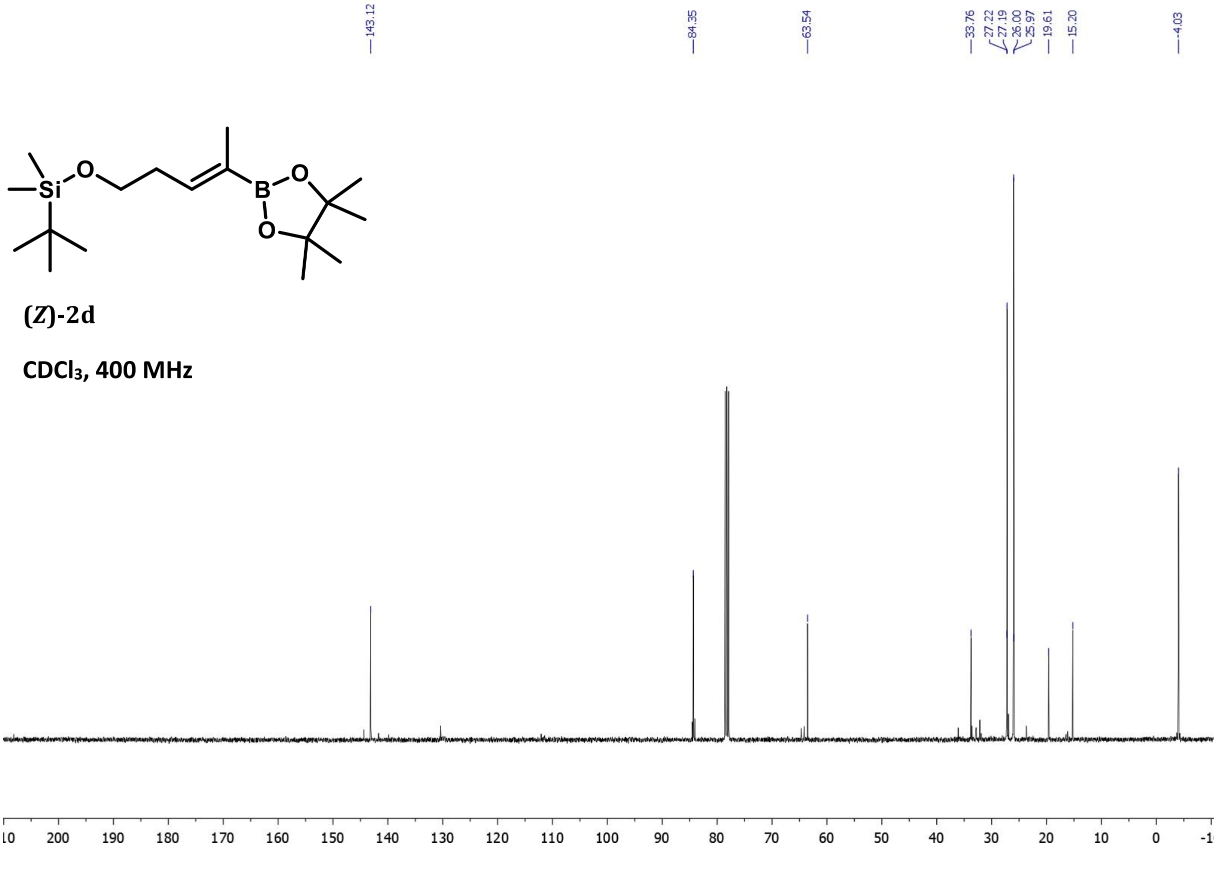

(Z)-2d

$CDCl_3$, 400 MHz

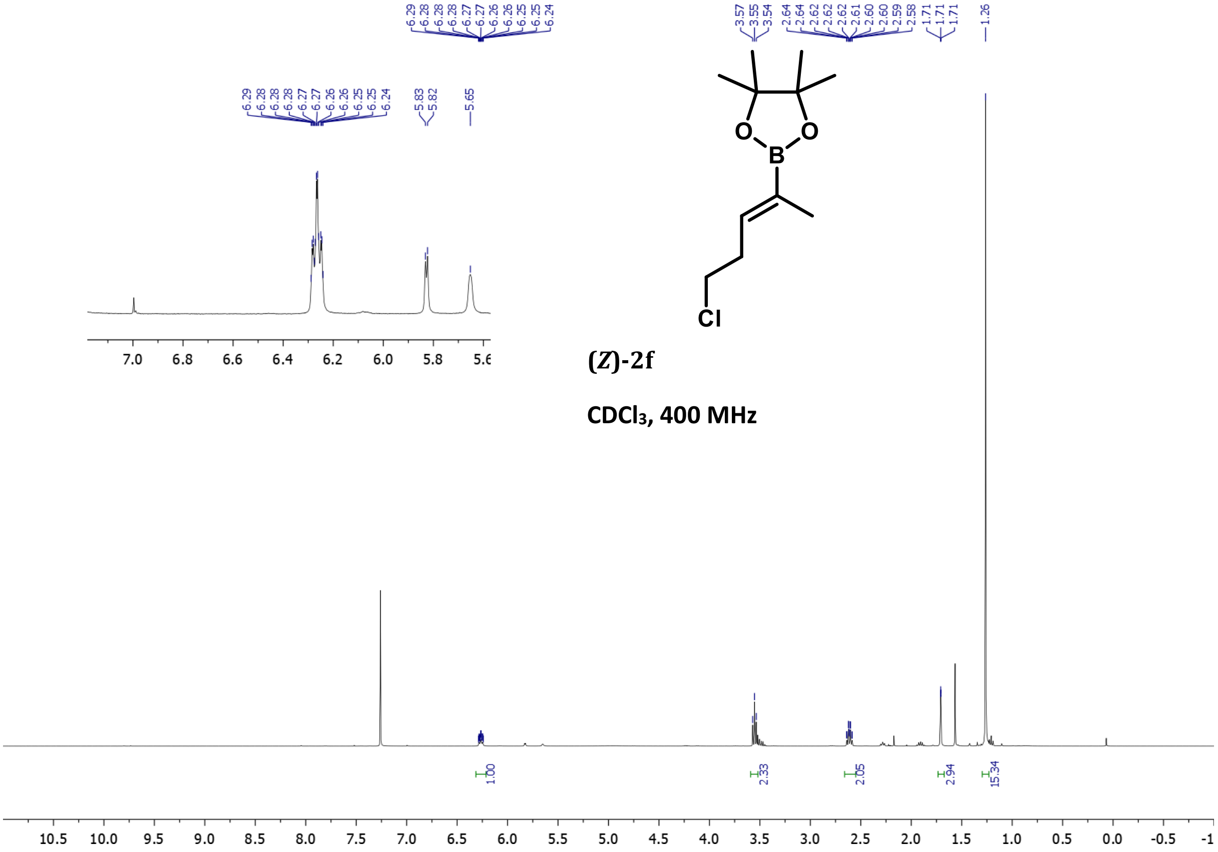
6.29 6.28 6.28 6.28 6.27 6.27 6.26 6.26 6.25 6.25 6.24
3.57 3.55 3.54 2.64 2.64 2.62 2.62 2.62 2.61 2.60 2.60 2.59 2.58 1.71 1.71 1.71
1.26
5.83 5.82
5.65
O
B
Cl
(Z)-2f
CDCl3, 400 MHz
1.00
2.33
2.05
2.94
15.34

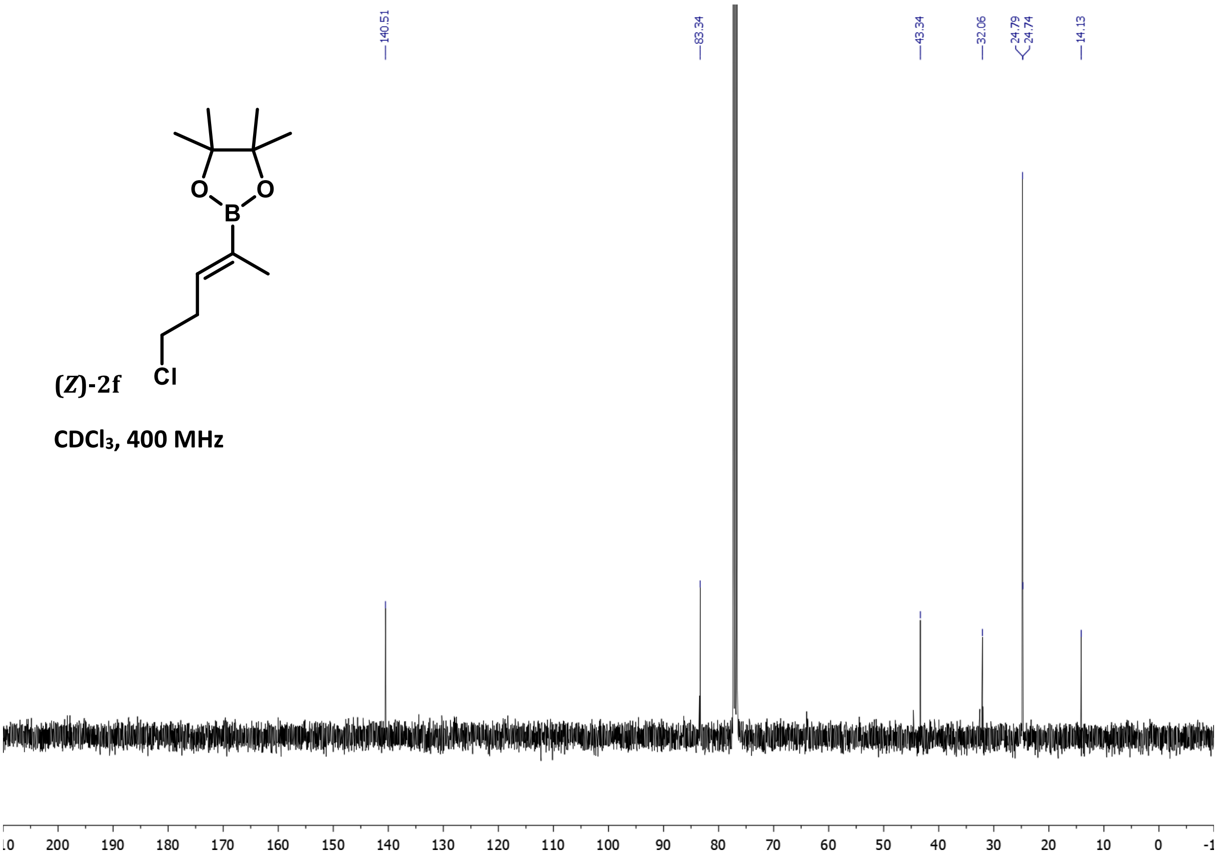
140.51
83.34
43.34
32.06
24.79 24.74
14.13
O
B
Cl
(Z)-2f
CDCl3, 400 MHz

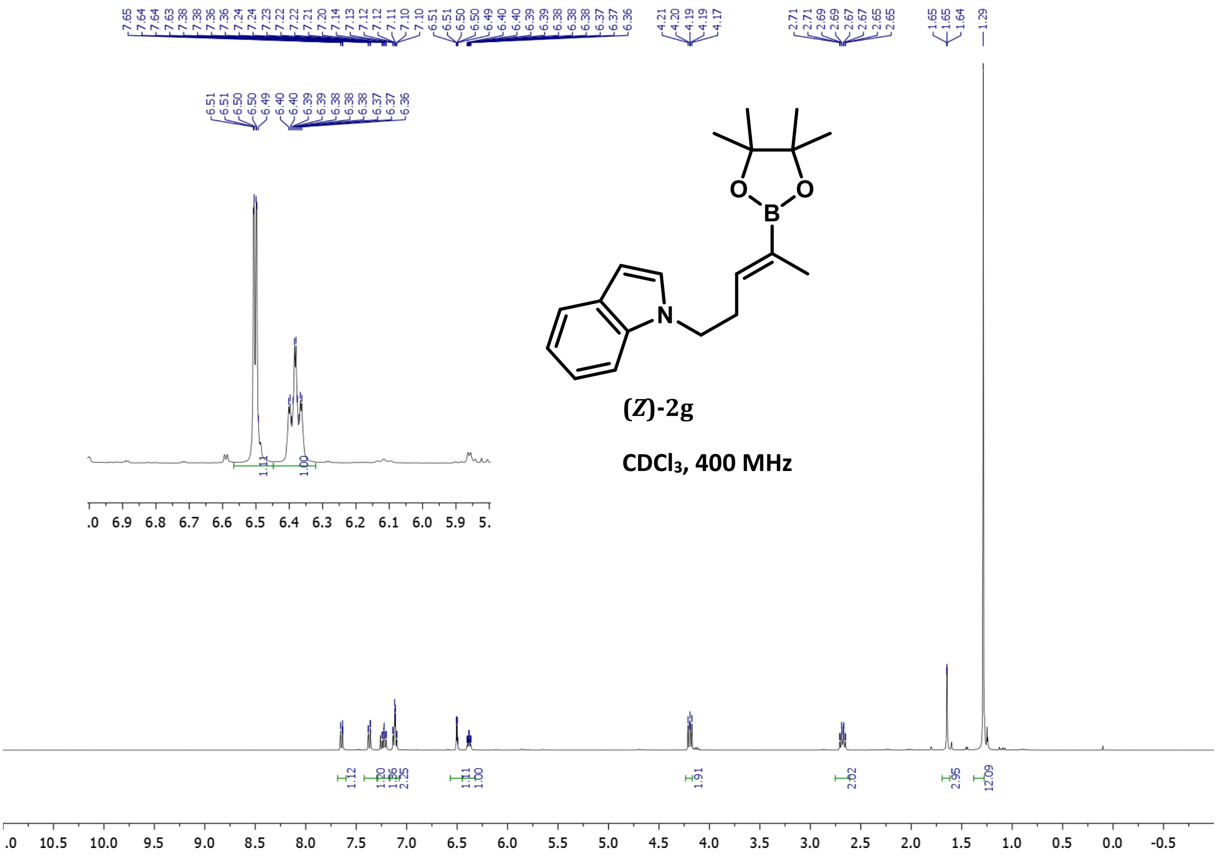
7.65 7.64 7.63 7.38 7.38 7.36 7.36 7.24 7.24 7.23 7.22 7.22 7.21 7.20 7.14 7.13 7.12 7.12 7.11 7.10 7.10 6.51 6.51 6.50 6.50 6.49 6.40 6.40 6.39 6.39 6.38 6.38 6.38 6.37 6.37 6.36 4.21 4.20 4.19 4.19 4.17 2.71 2.71 2.69 2.69 2.67 2.67 2.65 1.65 1.65 1.64 1.29
6.51 6.51 6.50 6.50 6.49 6.40 6.40 6.39 6.39 6.38 6.38 6.38 6.37 6.37 6.36
O
B
O
N
(Z)-2g
CDCl3, 400 MHz
1.11 1.00
.0 6.9 6.8 6.7 6.6 6.5 6.4 6.3 6.2 6.1 6.0 5.9 5.
1.12 1.20 1.16 2.25 1.11 1.00 1.91 2.02 2.95 12.09
.0 10.5 10.0 9.5 9.0 8.5 8.0 7.5 7.0 6.5 6.0 5.5 5.0 4.5 4.0 3.5 3.0 2.5 2.0 1.5 1.0 0.5 0.0 -0.5

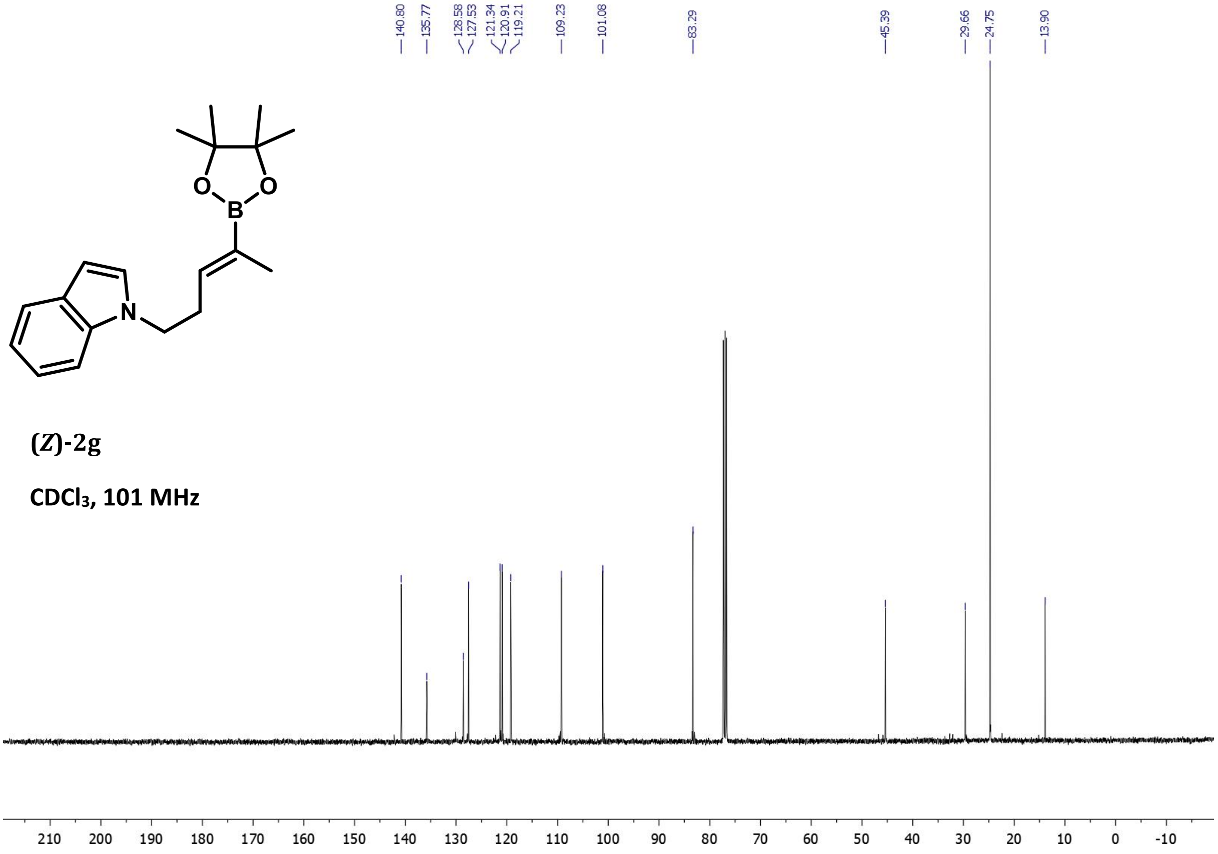
140.80 135.77 128.58 127.53 121.34 120.91 119.21 109.23 101.08 83.29 45.39 29.66 24.75 13.90
O
B
O
N
(Z)-2g
CDCl3, 101 MHz
210 200 190 180 170 160 150 140 130 120 110 100 90 80 70 60 50 40 30 20 10 0 -10

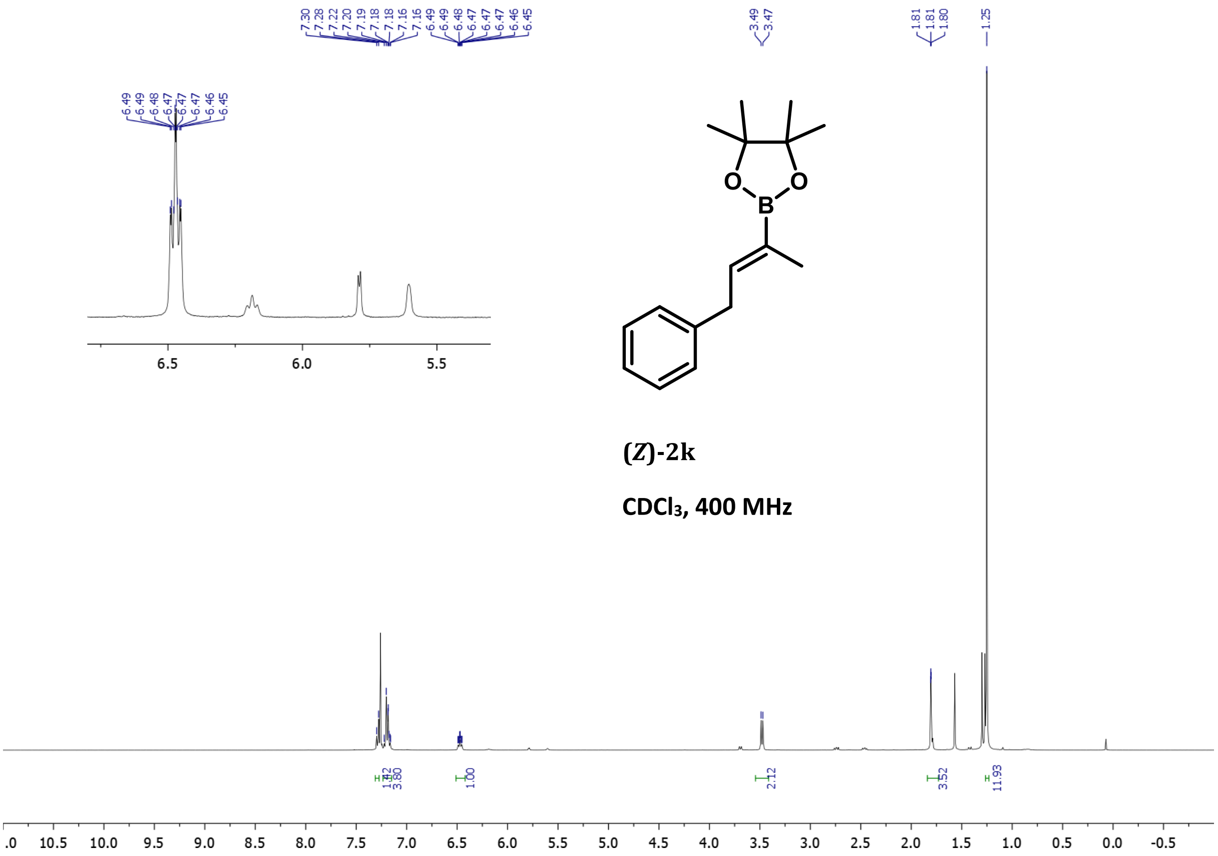
(Z)-2k
CDCl₃, 400 MHz
7.30 7.28 7.22 7.20 7.19 7.18 7.18 7.16 6.49 6.49 6.48 6.47 6.47 6.47 6.46 6.45
3.49 3.47
1.81 1.81 1.80
1.25
1.12 3.80 1.00 2.12 3.52 11.93

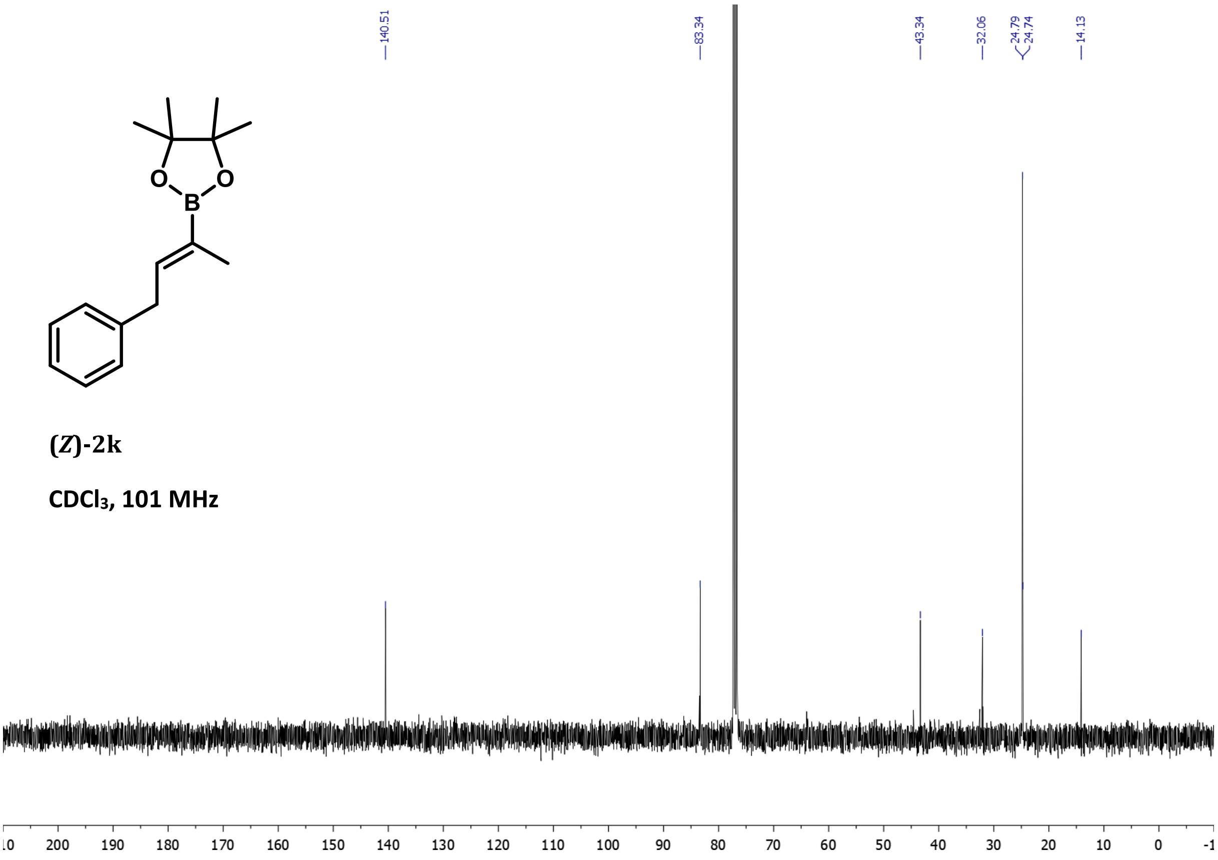
(Z)-2k
CDCl₃, 101 MHz
140.51
83.34
43.34
32.06
24.79 24.74
14.13

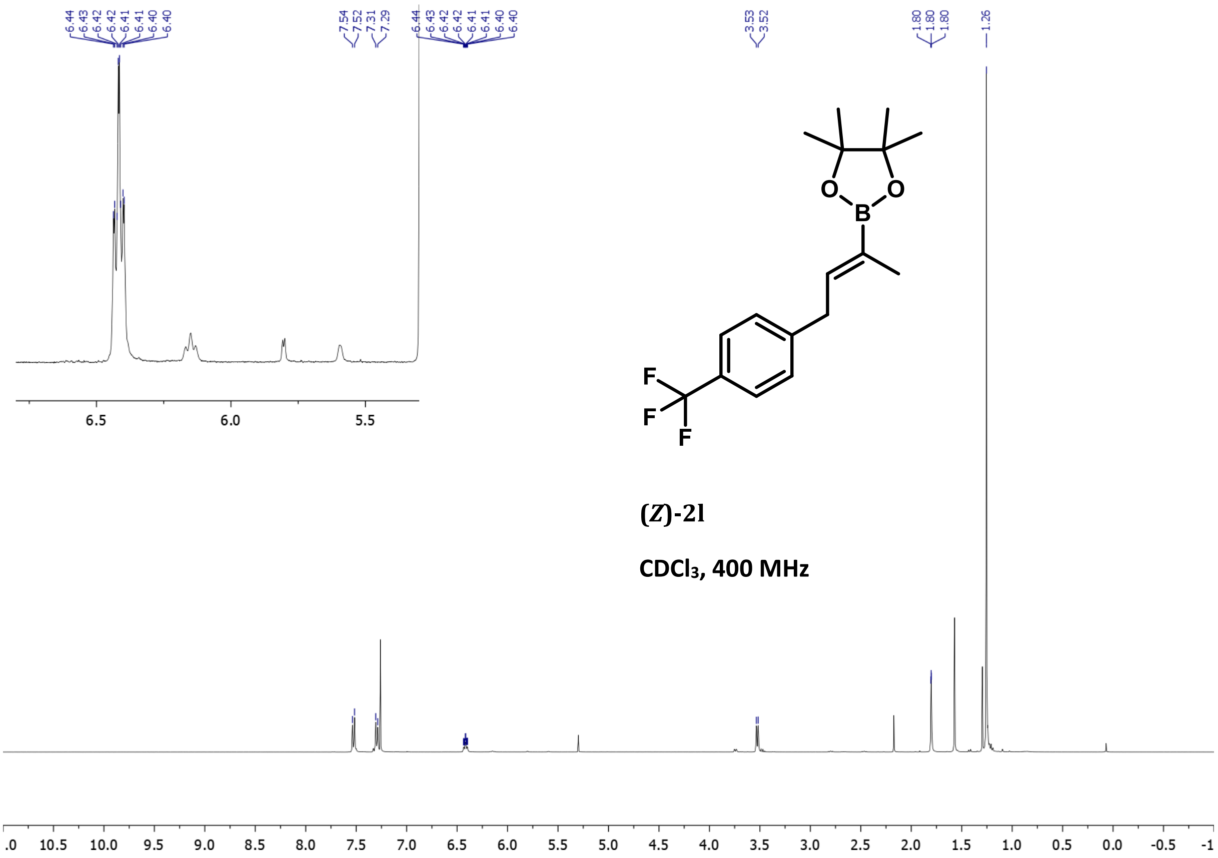
(Z)-2l
CDCl₃, 400 MHz
6.44
6.43
6.42
6.41
6.41
6.40
6.40
7.54
7.52
7.31
7.29
6.44
6.43
6.42
6.42
6.41
6.41
6.40
6.40
3.53
3.52
1.80
1.80
1.80
1.26

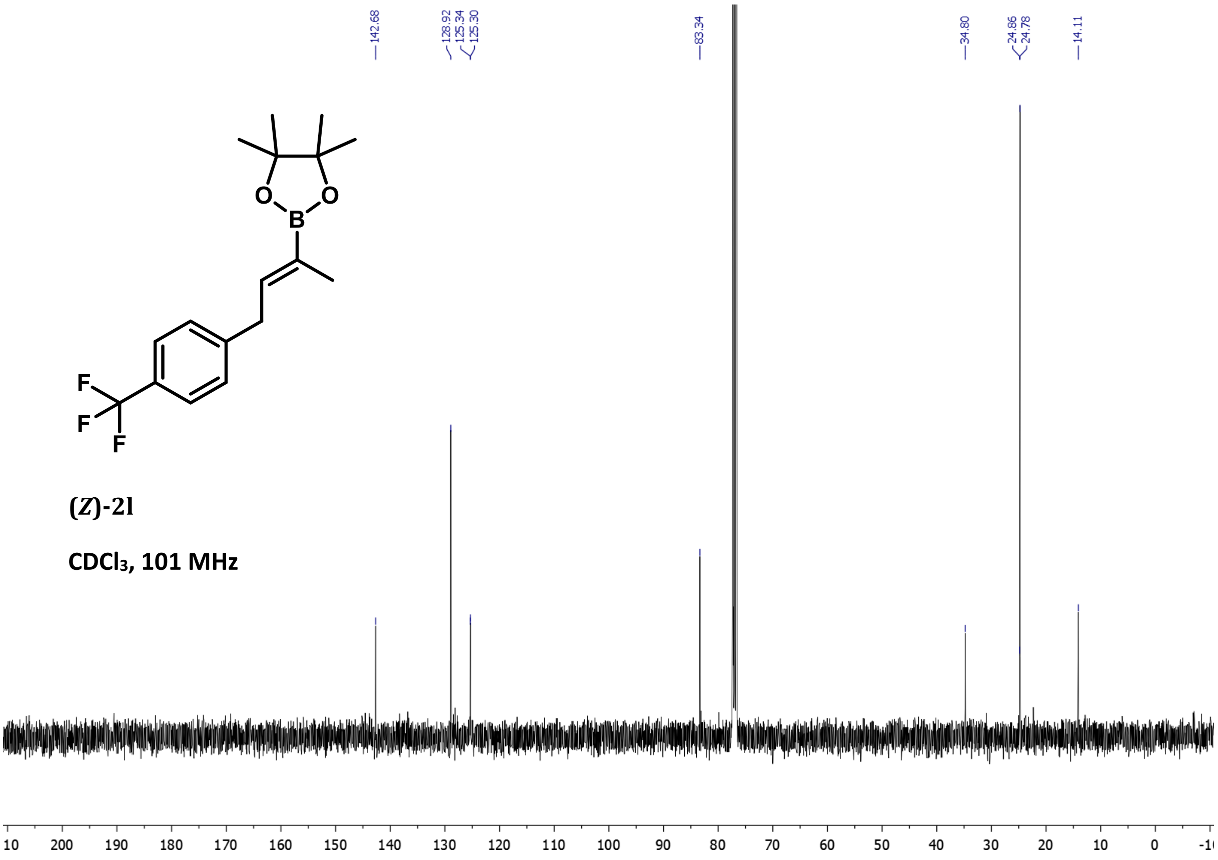
(Z)-2l
CDCl₃, 101 MHz
142.68
128.92
125.34
125.30
83.34
34.80
24.86
24.78
14.11

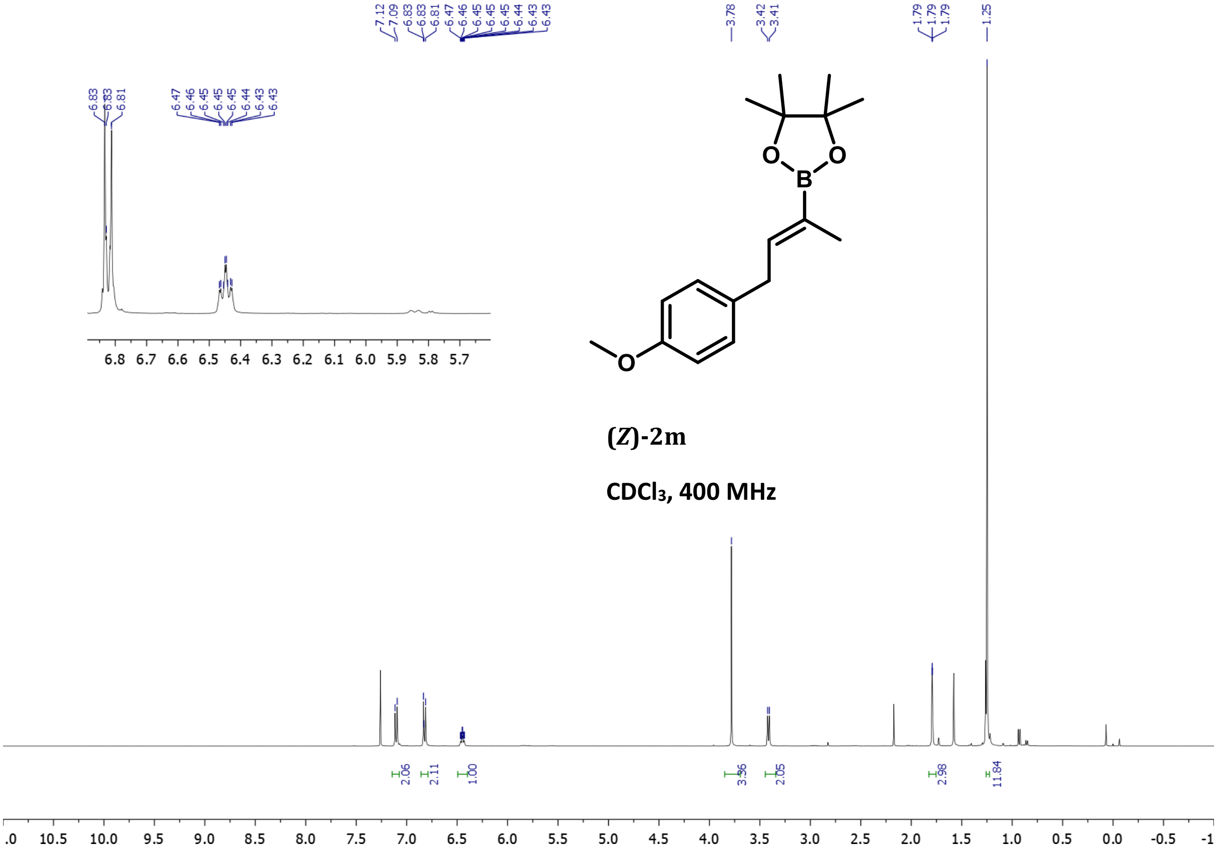
(Z)-2m
CDCl3, 400 MHz
7.12
7.09
6.83
6.83
6.81
6.47
6.46
6.45
6.45
6.45
6.44
6.43
6.43
3.78
3.42
3.41
1.79
1.79
1.79
1.25
2.06
2.11
1.00
3.36
2.05
2.98
11.84
O
B
O
O

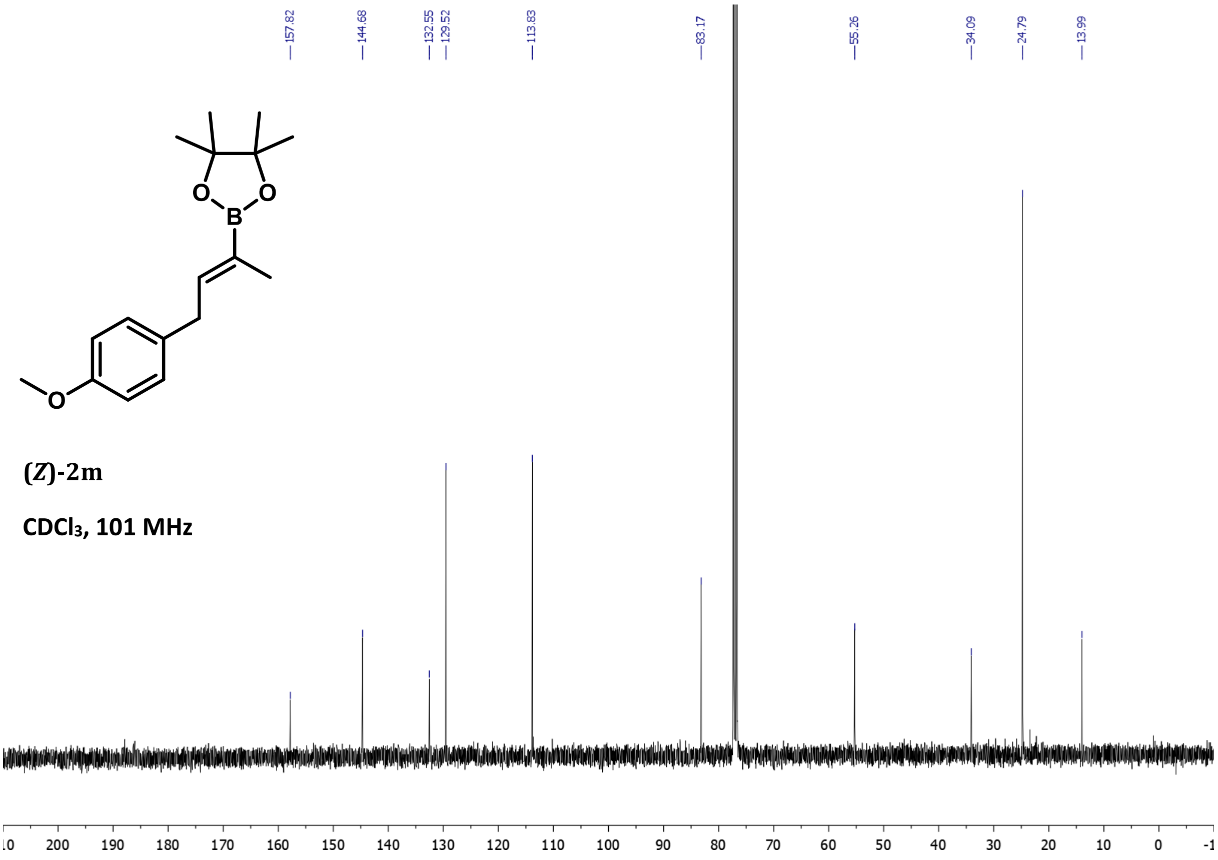
(Z)-2m
CDCl3, 101 MHz
157.82
144.68
132.55
129.52
113.83
83.17
55.26
34.09
24.79
13.99
O
B
O
O

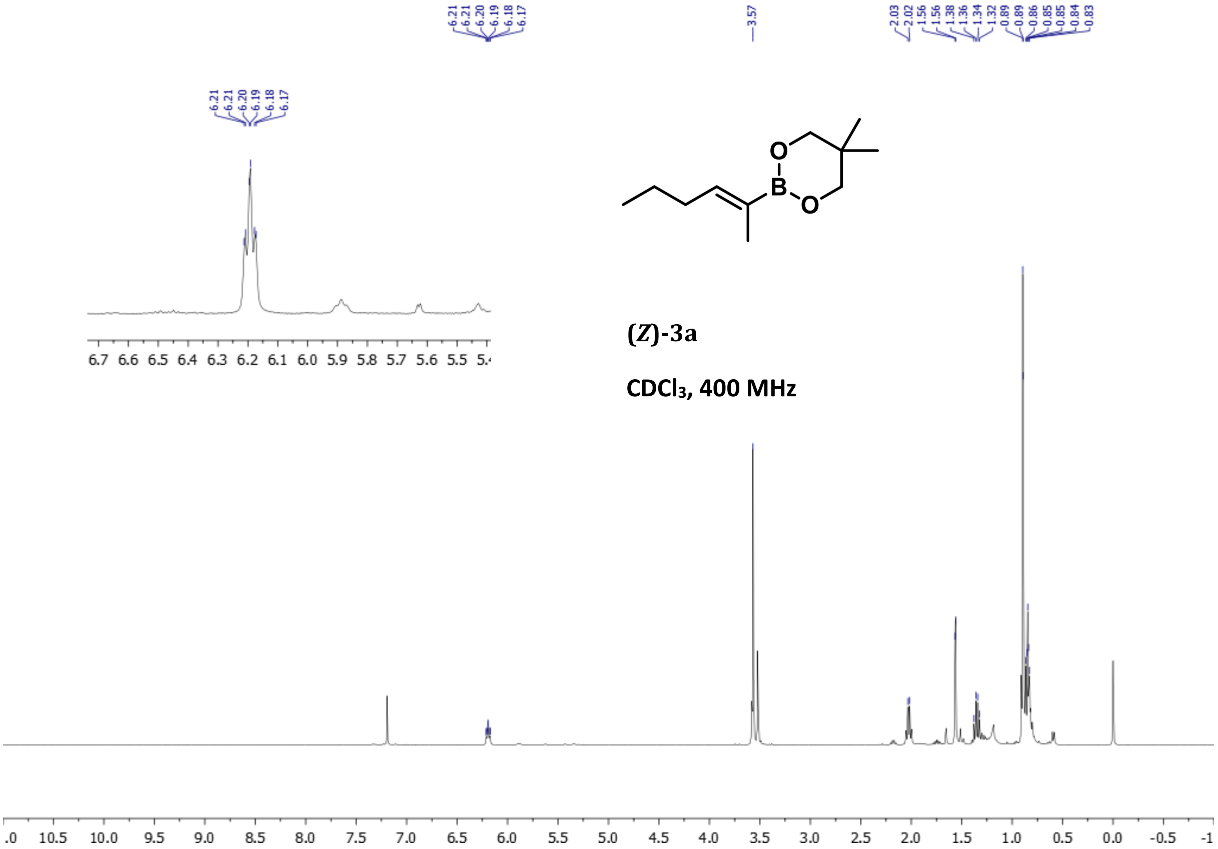
6.21
6.21
6.20
6.19
6.18
6.17
3.57
2.03
2.02
1.56
1.56
1.38
1.36
1.34
1.32
0.89
0.89
0.86
0.85
0.85
0.84
0.83
(Z)-3a
CDCl3, 400 MHz

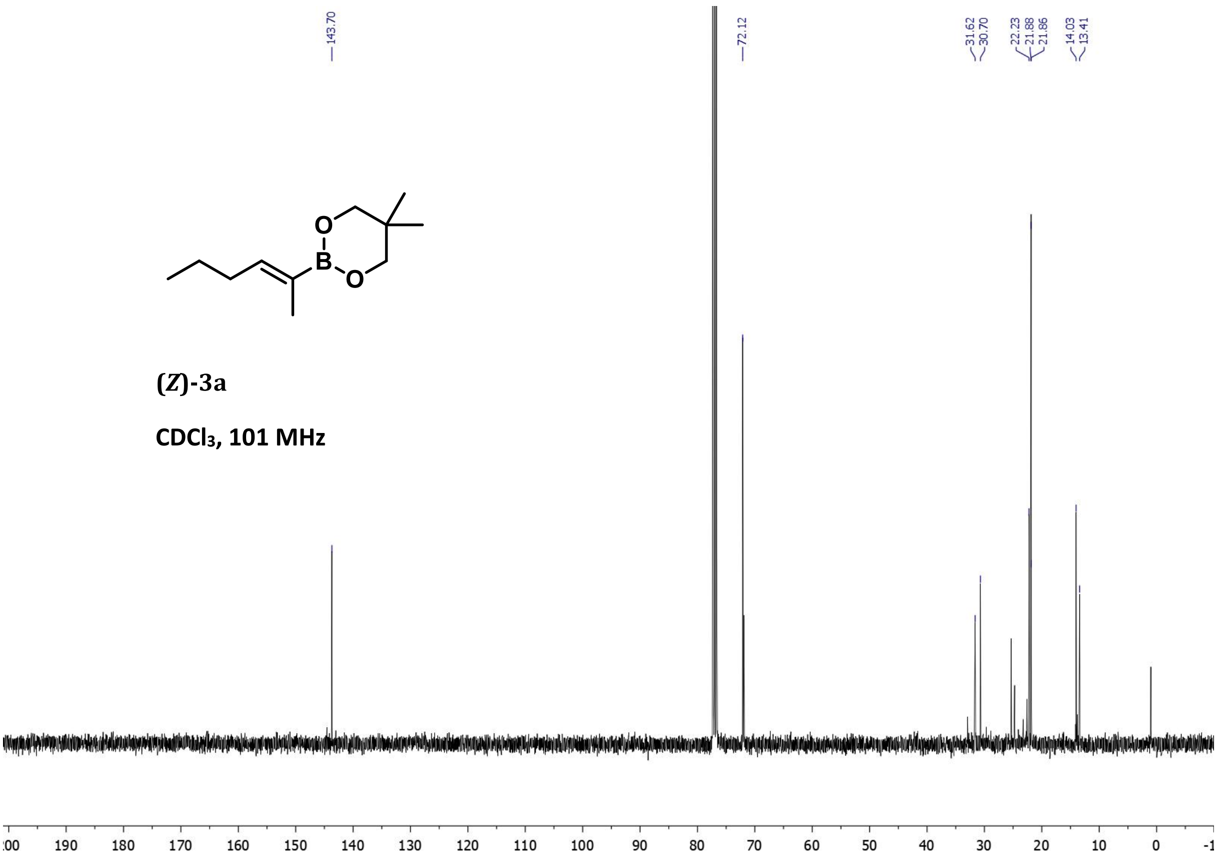
143.70
72.12
31.62
30.70
22.23
21.88
21.86
14.03
13.41
(Z)-3a
CDCl3, 101 MHz

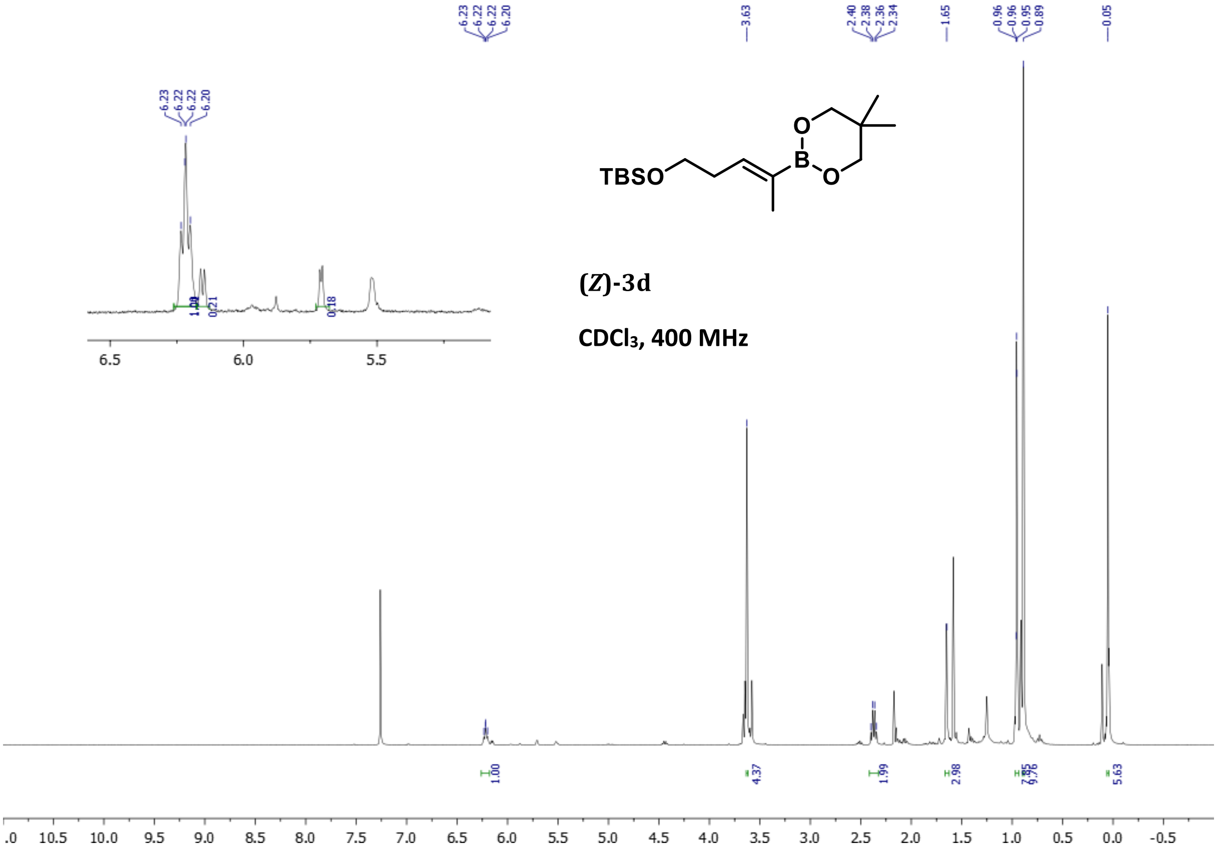
6.23 6.22 6.22 6.20
3.63
2.40 2.38 2.36 2.34
1.65
0.96 0.96 0.95 0.89
0.05
TBSO
O
B
O
(Z)-3d
CDCl₃, 400 MHz
1.00
4.37
1.99
2.98
5.63
6.5 6.0 5.5
10.5 10.0 9.5 9.0 8.5 8.0 7.5 7.0 6.5 6.0 5.5 5.0 4.5 4.0 3.5 3.0 2.5 2.0 1.5 1.0 0.5 0.0 -0.5

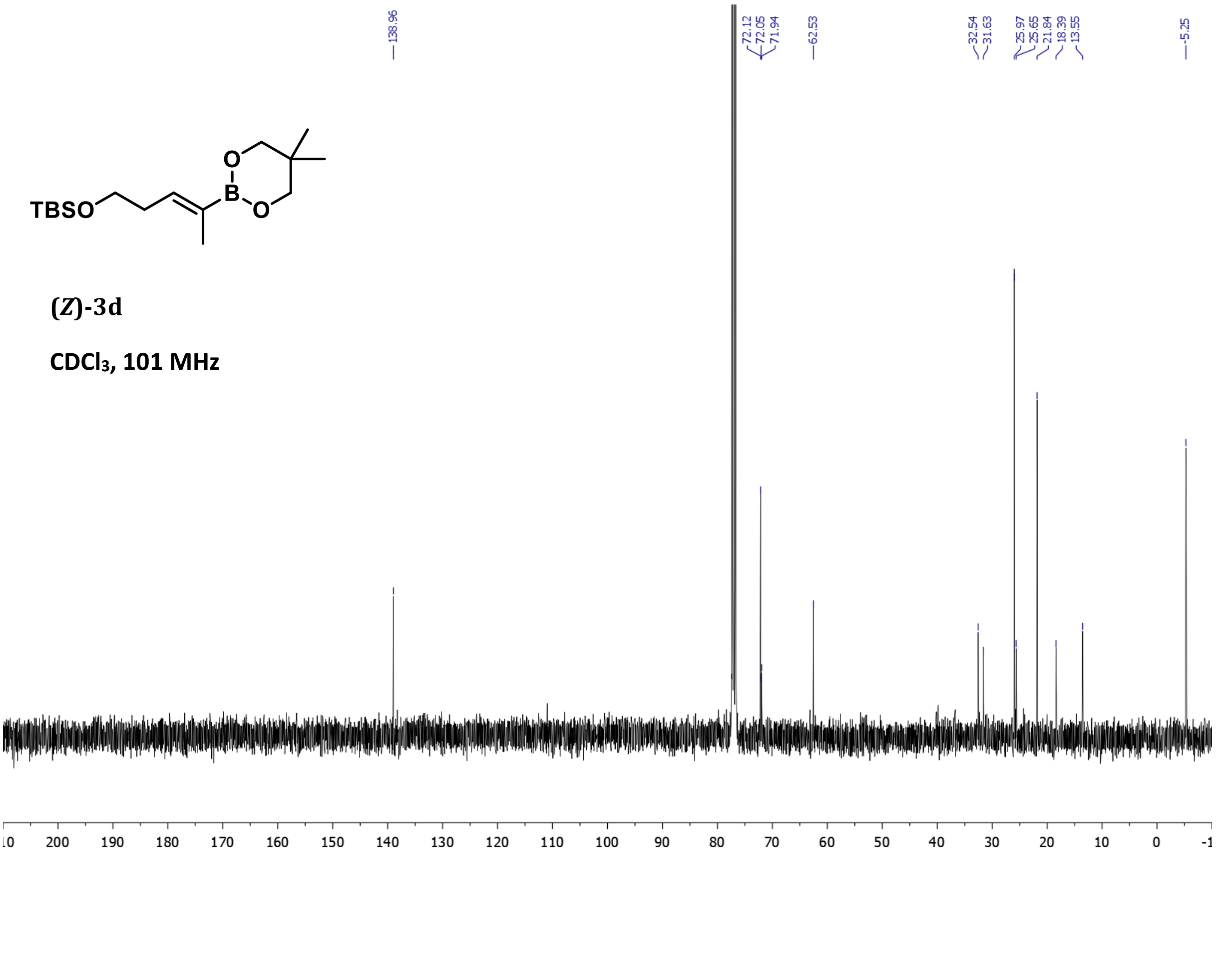
138.96
72.12 72.05 71.94
62.53
32.54 31.63
25.97 25.65 21.84 18.39 13.55
-5.25
TBSO
O
B
O
(Z)-3d
CDCl₃, 101 MHz
200 190 180 170 160 150 140 130 120 110 100 90 80 70 60 50 40 30 20 10 0

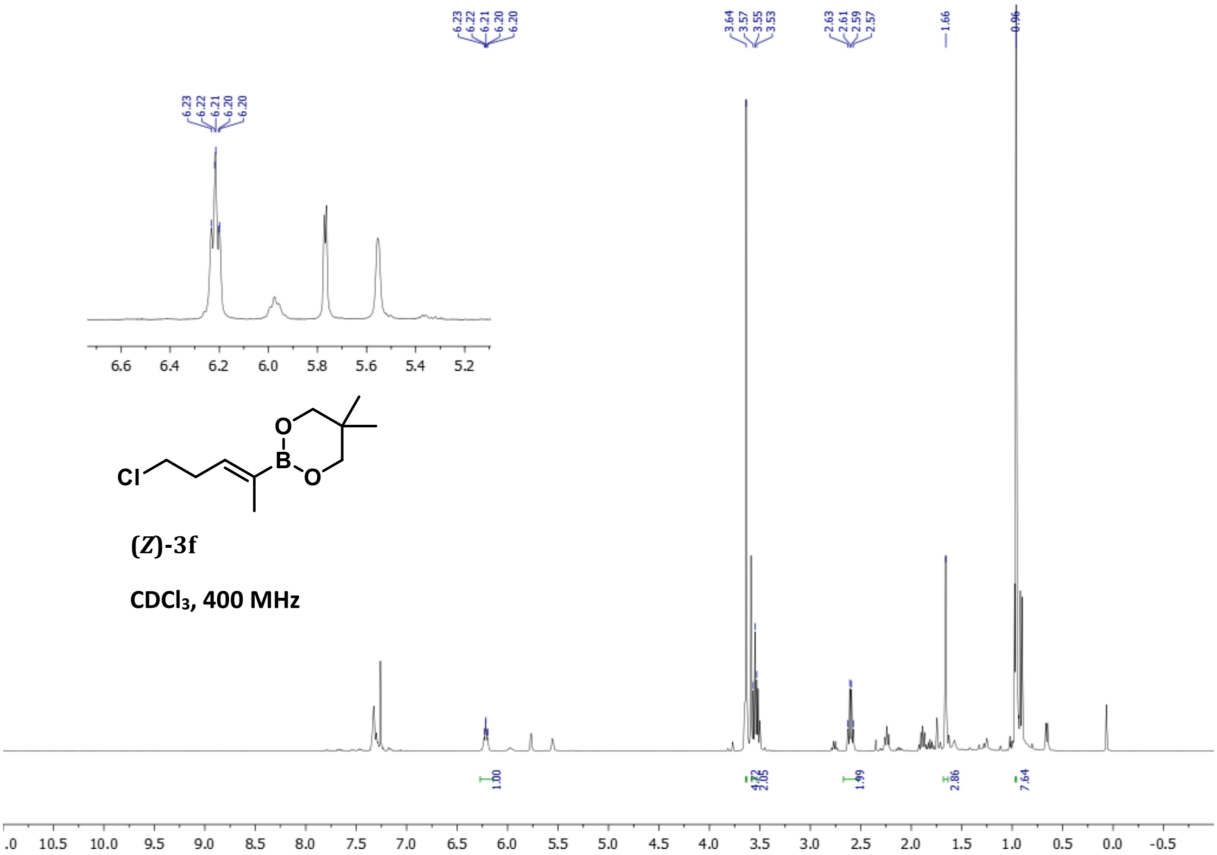
6.23
6.22
6.21
6.20
6.20
3.64
3.57
3.55
3.53
2.63
2.61
2.59
2.57
1.66
0.96
1.00
4.72
2.05
1.99
2.86
7.64
Cl
B
O
O
(Z)-3f
CDCl3, 400 MHz

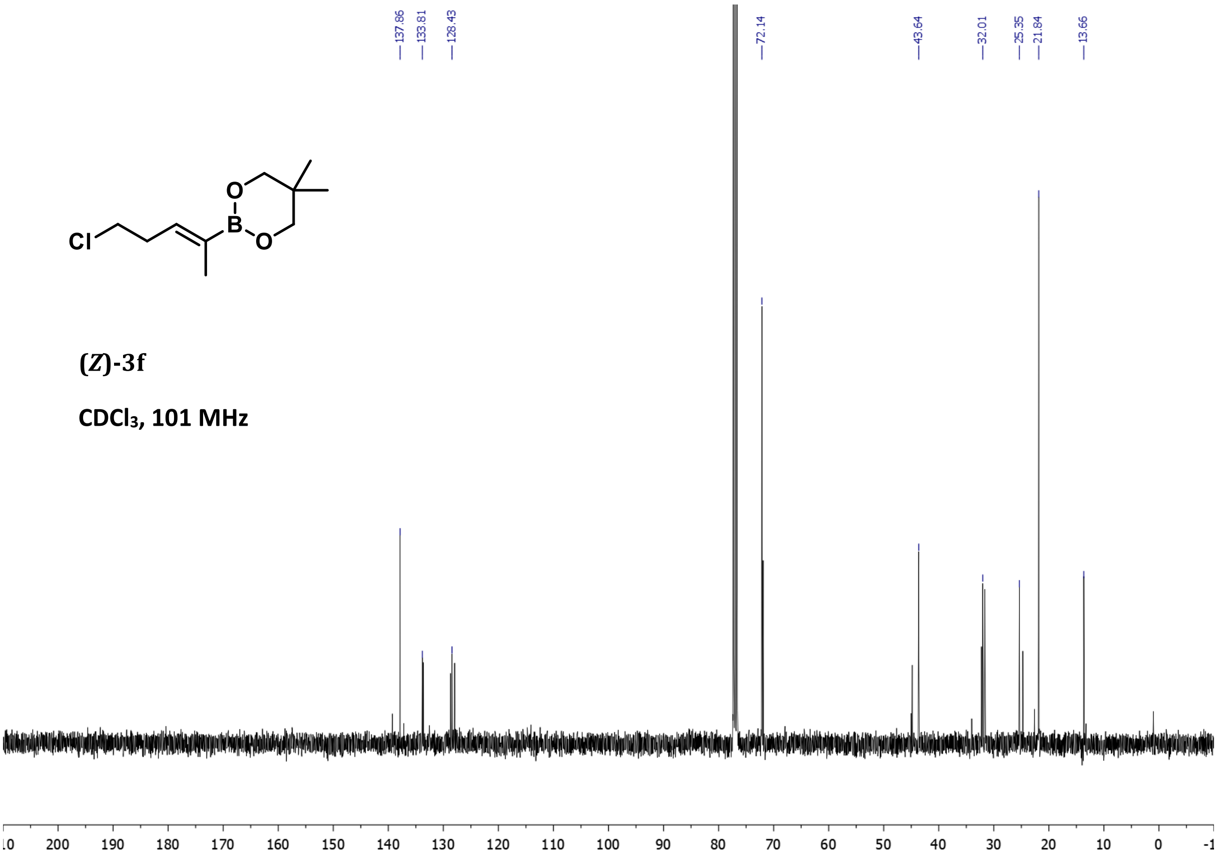
137.86
133.81
128.43
72.14
43.64
32.01
25.35
21.84
13.66
Cl
B
O
O
(Z)-3f
CDCl3, 101 MHz

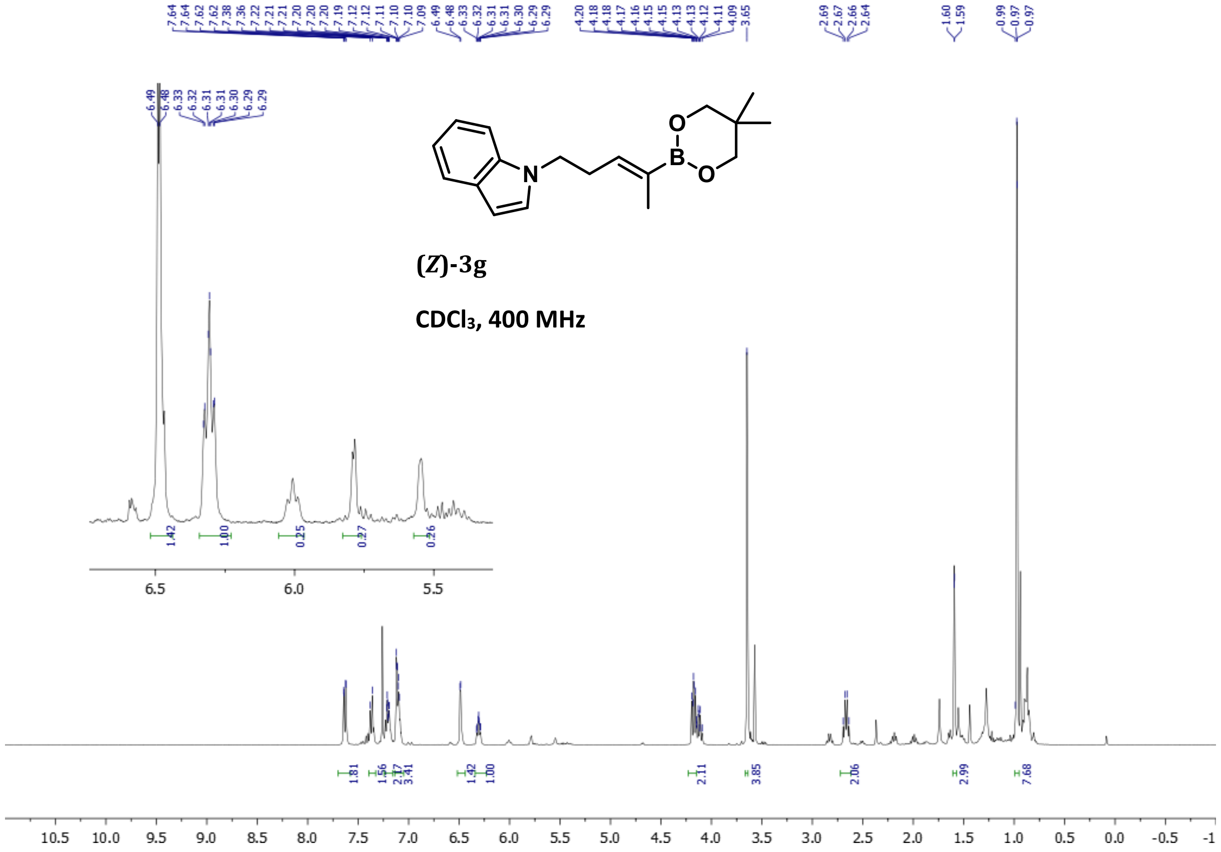
(Z)-3g
CDCl₃, 400 MHz

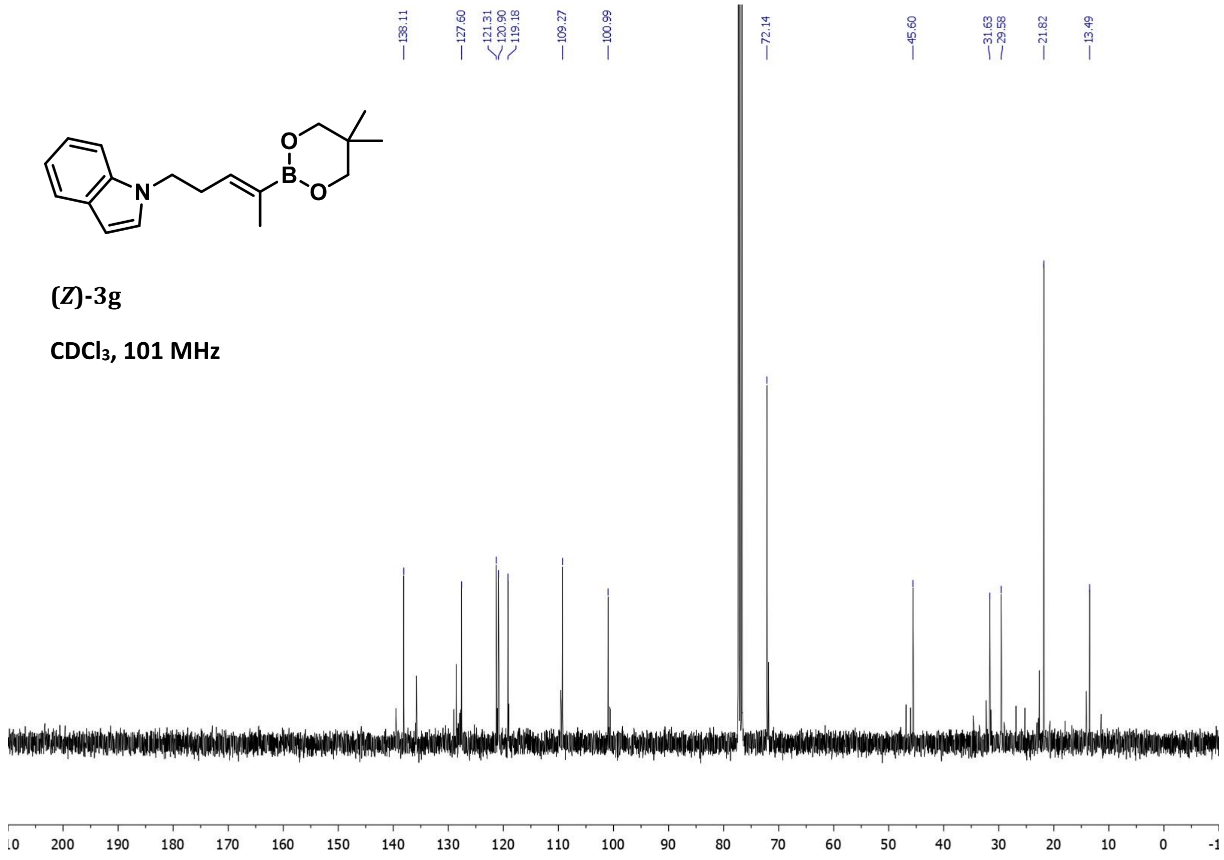
(Z)-3g
CDCl₃, 101 MHz

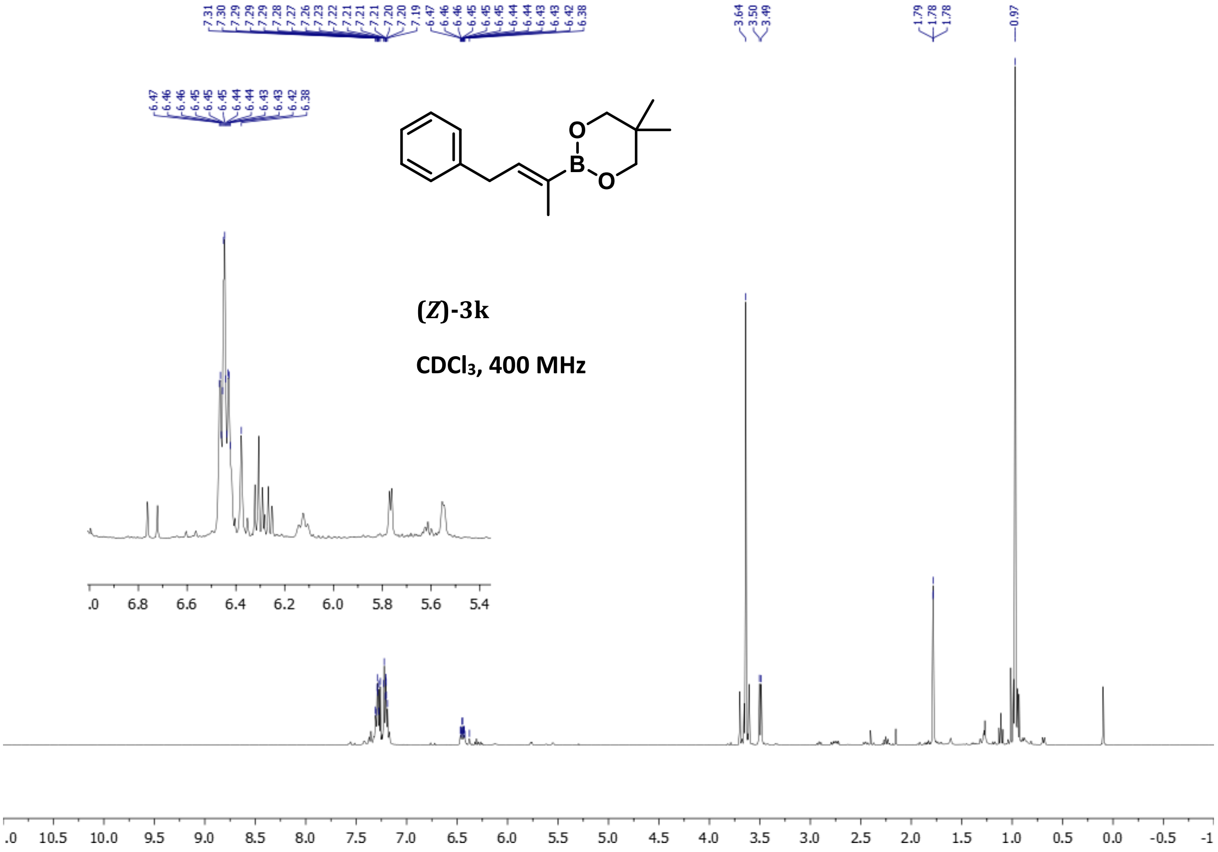
(Z)-3k
CDCl₃, 400 MHz
3.64
3.50
3.49
1.79
1.78
1.78
0.97

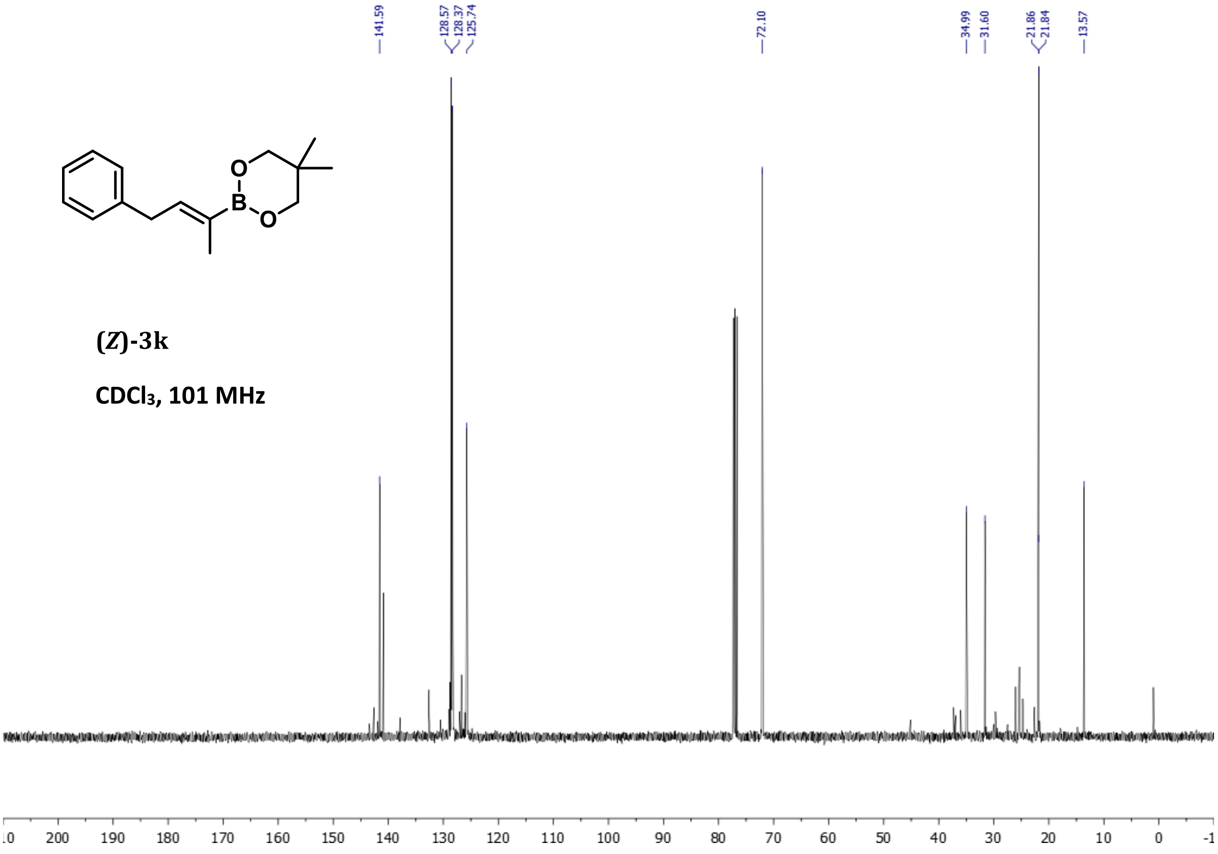
(Z)-3k
CDCl₃, 101 MHz
141.59
128.57
128.37
125.74
72.10
34.99
31.60
21.86
21.84
13.57

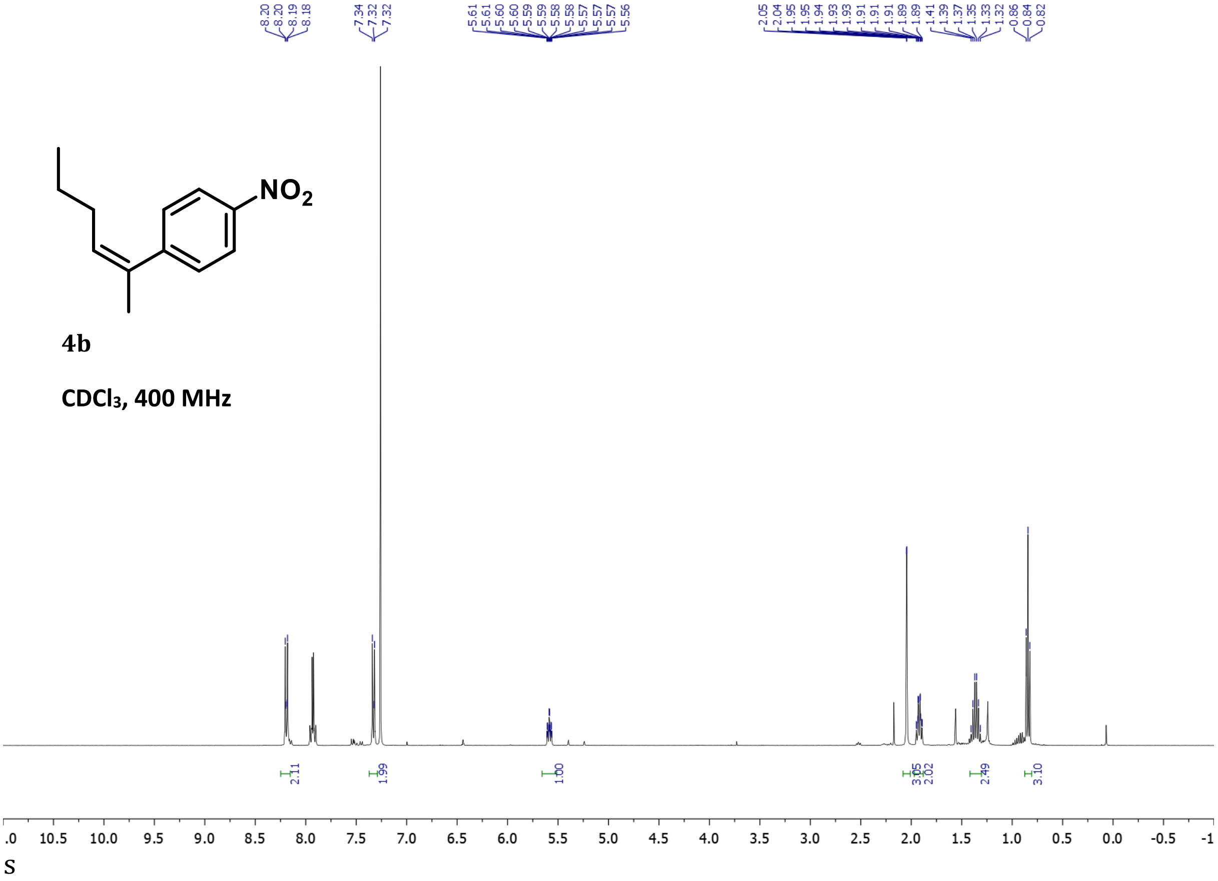
8.20
8.20
8.19
8.18
7.34
7.32
7.32
5.61
5.61
5.60
5.60
5.59
5.59
5.58
5.58
5.57
5.57
5.57
5.56
2.05
2.04
1.95
1.95
1.94
1.93
1.93
1.91
1.91
1.91
1.89
1.89
1.41
1.39
1.37
1.35
1.33
1.32
0.86
0.84
0.82
NO2
4b
CDCl3, 400 MHz
2.11
1.99
1.00
3.05
2.02
2.49
3.10

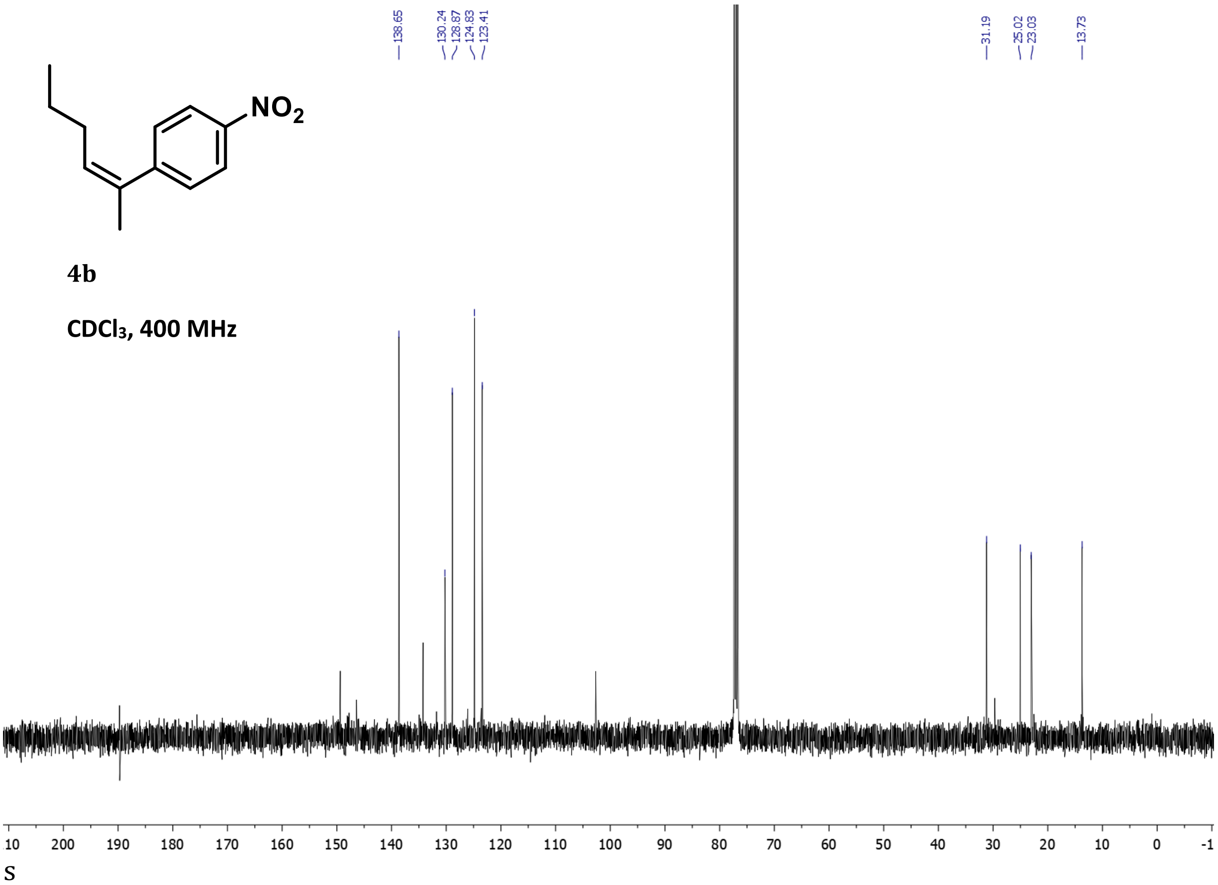
138.65
130.24
128.87
124.83
123.41
31.19
25.02
23.03
13.73
NO2
4b
CDCl3, 400 MHz
